# Supplementary material for: Why choose Random Forest to predict rare species distribution with few samples in large undersampled areas? Three Asian crane species models provide supporting evidence
Source: PeerJ. 2017 Jan 12;5:e2849. doi: 10.7717/peerj.2849 (PMC5237372; doi:10.7717/peerj.2849)
Supplement: Table S1 [file peerj-05-2849-s001.docx]

# Supporting Information

Supplement S1 Tracking cranes information in this study

| Species | Ring number | Track time | Track location |
| --- | --- | --- | --- |
| Hooded Crane | 385 | 2014/4/7 | Lindian |
| Hooded Crane | 386 | 2014/4/7 | Lindian |
| Hooded Crane | 387 | 2014/4/7 | Lindian |
| Hooded Crane | 388 | 2014/4/9 | Lindian |
| White-naped Crane | 478 | 2014/4/7 | Lindian |
| White-naped Crane | 480 | 2014/4/7 | Lindian |
| White-naped Crane | 481 | 2014/4/7 | Lindian |
| White-naped Crane | 483 | 2014/4/8 | Lindian |
| White-naped Crane | 484 | 2014/4/11 | Lindian |
| White-naped Crane | 485 | 2014/4/15 | Lindian |
| White-naped Crane | 486 | 2014/4/15 | Lindian |
| White-naped Crane | 438 | 2014/4/20 | Lindian |
