# Supplementary material for: Why choose Random Forest to predict rare species distribution with few samples in large undersampled areas? Three Asian crane species models provide supporting evidence
Source: PeerJ. 2017 Jan 12;5:e2849. doi: 10.7717/peerj.2849 (PMC5237372; doi:10.7717/peerj.2849)
Supplement: Figure S1 [file peerj-05-2849-s002.pdf]

Supplement S2 Prediction of three crane species with different predictor groups

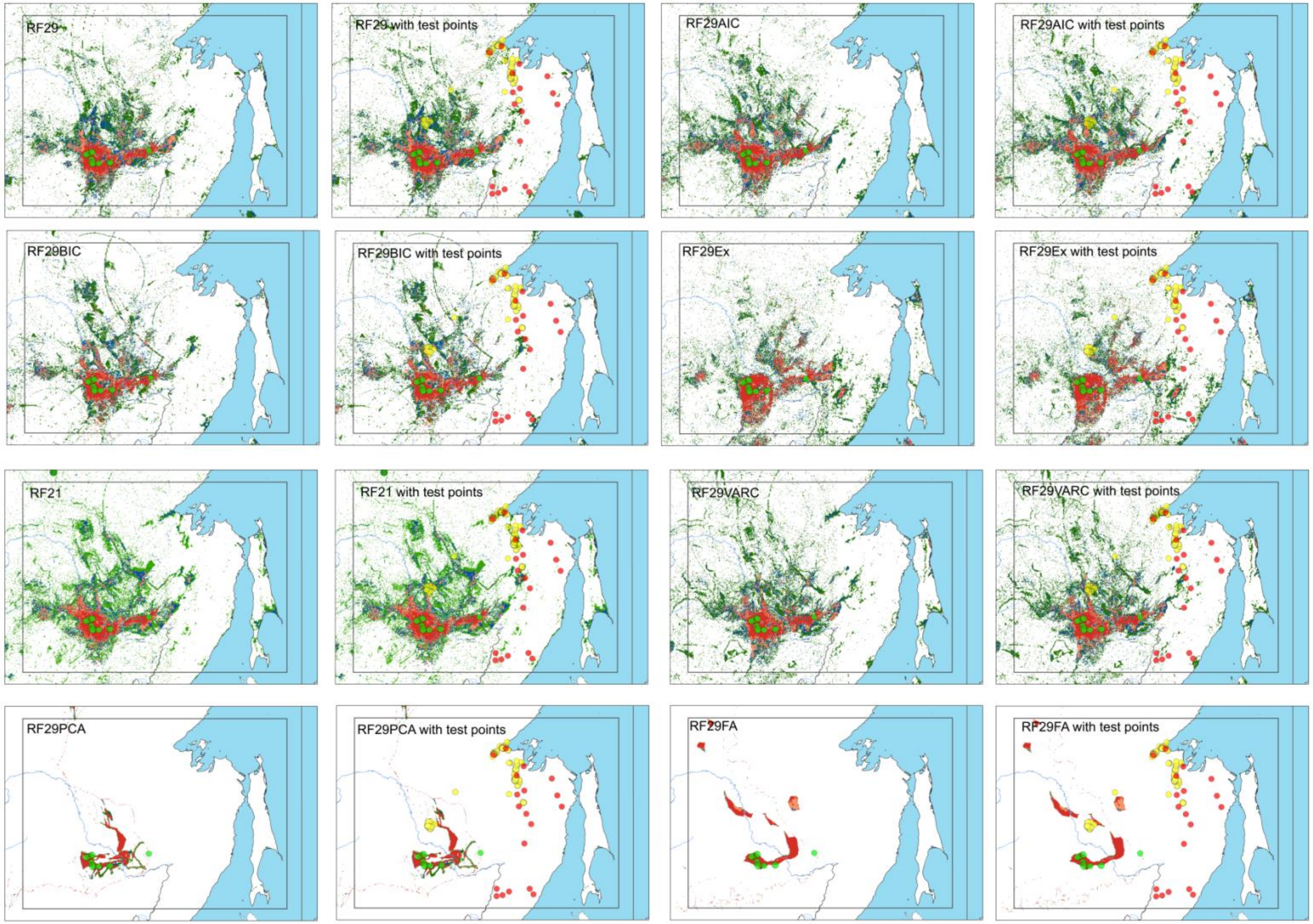

For Hooded Cranes, RF21 and RF29VARC are the best, RF21 a little bit better than RF29VARC. PCA and Factor Analysis(FA) are bad

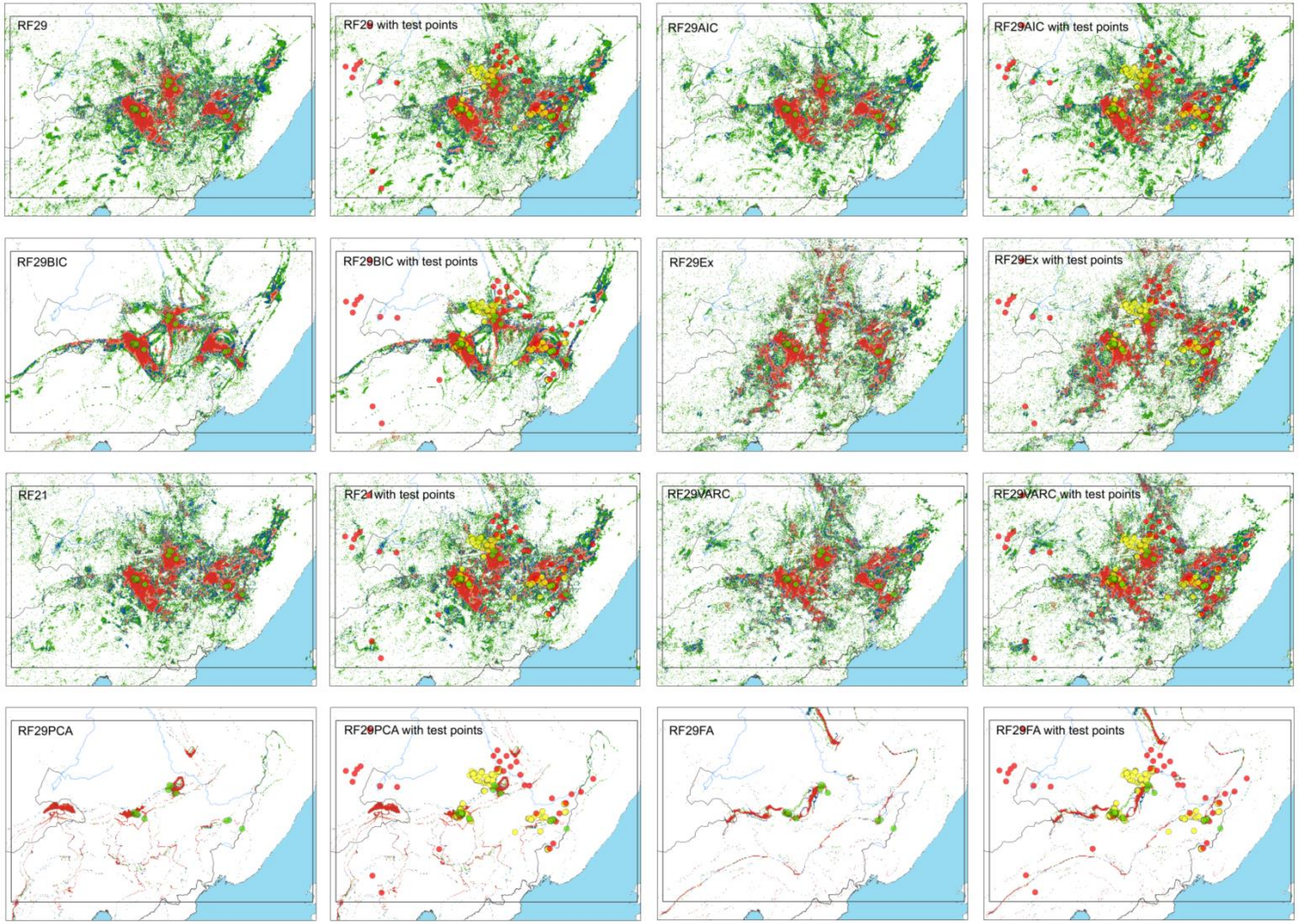

For White-naped Cranes, RF21 was in the first level group, PCA and Factor Analysis(FA) are bad.

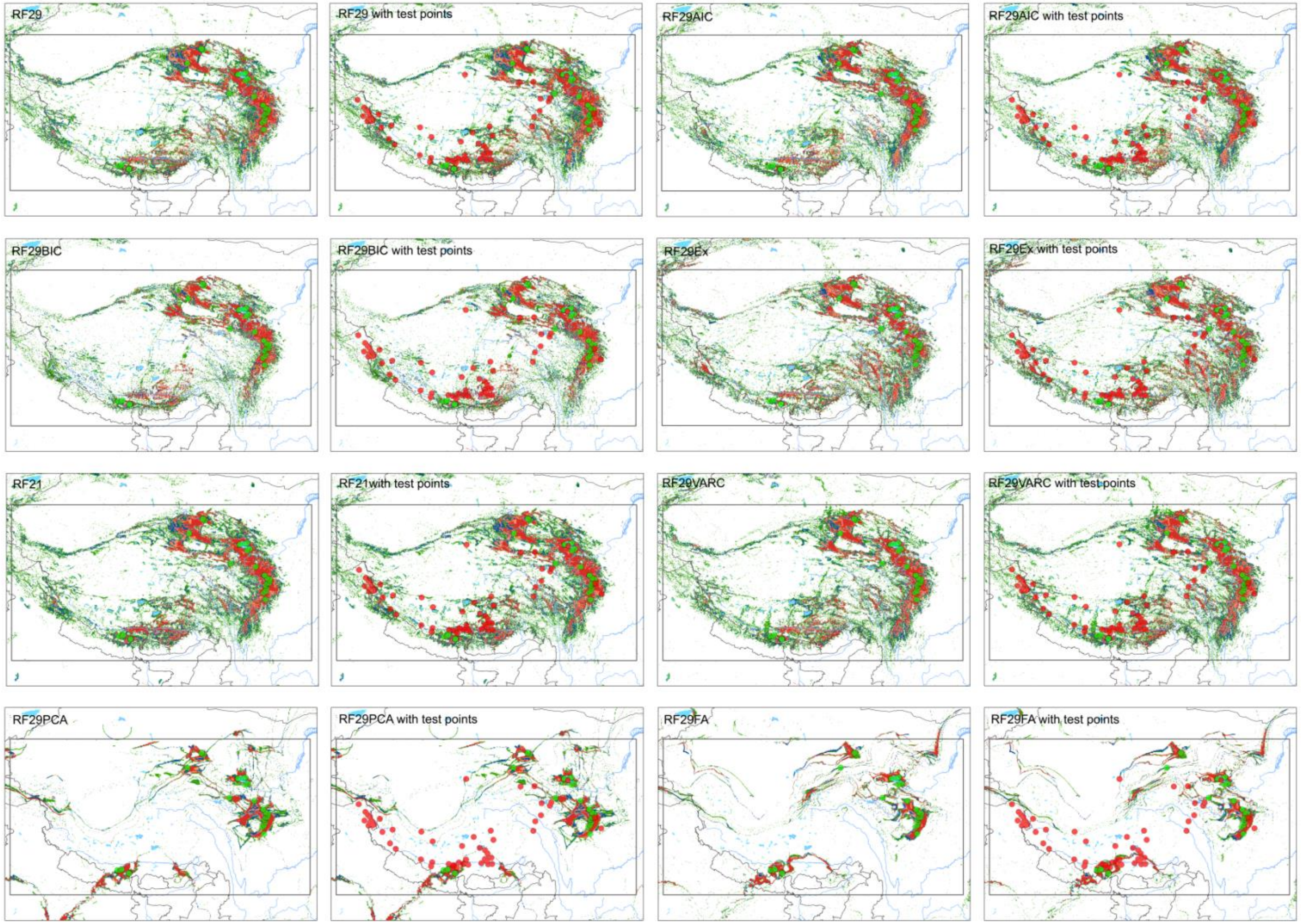

For Black-necked Cranes, RF21 was in the first level group, PCA and Factor Analysis (FA) are bad
