# Supplementary material for: Why choose Random Forest to predict rare species distribution with few samples in large undersampled areas? Three Asian crane species models provide supporting evidence
Source: PeerJ. 2017 Jan 12;5:e2849. doi: 10.7717/peerj.2849 (PMC5237372; doi:10.7717/peerj.2849)
Supplement: Supplemental Information 2 [file peerj-05-2849-s004.pdf]

Species	X	Y
Hooded Crane	-5829746.073	6207759.267
Hooded Crane	-5829585.328	6208657.913
Hooded Crane	-5797488.134	6227852.033
Hooded Crane	-5829987.302	6208030.760
Hooded Crane	-5781068.509	6142620.139
Hooded Crane	-5842485.921	6203908.415
Hooded Crane	-5611417.605	6123943.226
Hooded Crane	-5617728.863	6130609.536
Hooded Crane	-5619284.219	6128828.836
Hooded Crane	-5720284.950	6120514.903
Hooded Crane	-5784340.077	6224291.890
Hooded Crane	-5819619.116	6221408.445
Hooded Crane	-5792042.829	6160425.921
Hooded Crane	-5611176.487	6124017.462
Hooded Crane	-5787840.519	6132186.387
Hooded Crane	-5819498.446	6221338.071
Hooded Crane	-5791838.670	6235291.265
Hooded Crane	-5794303.172	6134867.908
Hooded Crane	-5774111.041	6120769.541
Hooded Crane	-5774114.158	6120769.541
Hooded Crane	-5783474.234	6228203.841
Hooded Crane	-5793313.653	6234544.645
Hooded Crane	-5607765.769	6127433.351
Hooded Crane	-5786009.870	6134566.244
Hooded Crane	-5789717.477	6234258.151
Hooded Crane	-5788919.650	6235516.600
Hooded Crane	-5783165.100	6229325.230
Hooded Crane	-5780978.897	6116184.908
Hooded Crane	-5782546.609	6134213.649
Hooded Crane	-5846901.631	6208573.661
Hooded Crane	-5780480.965	6114421.236
Hooded Crane	-5781247.844	6116092.407
Hooded Crane	-5231700.699	6246003.976
White-naped Crane	-6185993.179	5944318.022
White-naped Crane	-6205239.707	5946496.282
White-naped Crane	-6215239.904	5937819.195
White-naped Crane	-6206191.488	5947738.136
White-naped Crane	-6215093.329	5938208.226
White-naped Crane	-6197792.433	5939035.311
White-naped Crane	-6209653.524	5936589.651
White-naped Crane	-5273235.226	5872610.731
White-naped Crane	-5276358.294	5870316.158
White-naped Crane	-5301411.413	5865971.317
White-naped Crane	-5299800.398	5865903.978
White-naped Crane	-5301157.828	5864829.365
White-naped Crane	-6205505.014	5946854.004
White-naped Crane	-6204818.507	5944094.837
White-naped Crane	-6206559.432	5943718.173
White-naped Crane	-6203621.822	5944557.604
White-naped Crane	-6208513.757	5950631.097
White-naped Crane	-6204286.734	5948501.692
White-naped Crane	-5860111.581	6183532.074
White-naped Crane	-5839928.690	6208568.943
White-naped Crane	-6167699.691	5923617.166
White-naped Crane	-6176614.156	5926442.160
White-naped Crane	-6178343.949	5924248.158
White-naped Crane	-6180828.266	5926565.723

White-naped Crane	-6182434.718	5930175.904
White-naped Crane	-6181183.598	5931920.231
White-naped Crane	-6188471.240	5925776.615
White-naped Crane	-6196568.508	5930435.533
White-naped Crane	-6195872.205	5926712.925
White-naped Crane	-6189023.496	5934325.517
White-naped Crane	-6185243.976	5938793.163
White-naped Crane	-6179814.925	5941651.713
White-naped Crane	-6104709.779	5885088.115
White-naped Crane	-6102307.950	5884734.293
White-naped Crane	-5144481.878	5786307.907
White-naped Crane	-5869258.258	6170018.688
White-naped Crane	-5869258.258	6170857.398
White-naped Crane	-5791705.754	6136538.443
White-naped Crane	-5840810.006	6210043.479
White-naped Crane	-5858002.633	6216365.850
Black-necked Crane	-9048070.166	4176962.818
Black-necked Crane	-8650487.037	3825222.745
Black-necked Crane	-8629573.199	3844535.335
Black-necked Crane	-8616037.061	3880253.350
Black-necked Crane	-8622692.730	3906065.919
Black-necked Crane	-8625944.184	3913251.051
Black-necked Crane	-8625865.336	3913848.788
Black-necked Crane	-8612547.506	3932729.107
Black-necked Crane	-8602935.680	3939477.357
Black-necked Crane	-8584012.291	3944250.817
Black-necked Crane	-8577697.392	3967727.105
Black-necked Crane	-8580713.527	3986422.522
Black-necked Crane	-8609259.551	3999901.077
Black-necked Crane	-8609466.428	3999658.007
Black-necked Crane	-8630198.447	3742674.402
Black-necked Crane	-8646049.719	4033972.417
Black-necked Crane	-8644825.828	4033639.276
Black-necked Crane	-8644313.135	4036081.677
Black-necked Crane	-8641422.235	4035069.808
Black-necked Crane	-8747599.377	4138414.916
Black-necked Crane	-8947225.530	4405316.168
Black-necked Crane	-8945432.359	4403884.742
Black-necked Crane	-8946953.726	4401514.231
Black-necked Crane	-8944786.706	4405367.403
Black-necked Crane	-8941867.352	4407725.300
Black-necked Crane	-8851827.204	4419699.048
Black-necked Crane	-8853454.940	4420316.672
Black-necked Crane	-8855256.769	4421335.579
Black-necked Crane	-8856292.964	4421935.549
Black-necked Crane	-8856023.326	4422073.683
Black-necked Crane	-8855714.102	4422033.554
Black-necked Crane	-8854385.070	4423849.126
Black-necked Crane	-8852620.968	4423267.182
Black-necked Crane	-8874711.397	4417280.988
Black-necked Crane	-8853125.924	4424535.306
Black-necked Crane	-8820745.867	4353153.313
Black-necked Crane	-8820618.162	4353337.695
Black-necked Crane	-8820100.526	4352165.908
Black-necked Crane	-8853641.089	4333664.909
Black-necked Crane	-9373216.774	4707686.881
Black-necked Crane	-9375929.567	4718866.788
Black-necked Crane	-9371217.970	4716238.225

Black-necked Crane	-9369514.164	4715945.146
Black-necked Crane	-9375349.160	4719047.135
Black-necked Crane	-9377646.670	4719039.189
Black-necked Crane	-9385390.178	4723755.578
Black-necked Crane	-9372666.669	4716586.913
Black-necked Crane	-9371076.656	4716436.793
Black-necked Crane	-9370208.982	4715091.773
Black-necked Crane	-9371029.346	4716430.042
Black-necked Crane	-9370872.261	4716480.875
Black-necked Crane	-9373019.491	4716625.437
Black-necked Crane	-9376384.740	4719184.979
Black-necked Crane	-9377286.428	4719130.953
Black-necked Crane	-9361240.343	4698441.153
Black-necked Crane	-9378217.183	4708132.489
Black-necked Crane	-9384186.382	4716910.592
Black-necked Crane	-9552412.087	4541148.039
Black-necked Crane	-10261304.808	3270358.041
Black-necked Crane	-10251421.493	3270987.453
Black-necked Crane	-10253612.941	3267494.090
Black-necked Crane	-10255471.667	3264042.866
Black-necked Crane	-10255883.240	3264520.373
Black-necked Crane	-10257171.145	3263673.307
Black-necked Crane	-10256152.571	3264151.849
Black-necked Crane	-10256902.432	3265490.111
Black-necked Crane	-10257269.786	3264664.292
Black-necked Crane	-10259174.278	3261814.493
Black-necked Crane	-10262642.188	3269873.334
Black-necked Crane	-10258587.685	3266834.448
Black-necked Crane	-10397579.037	3298745.989
Black-necked Crane	-10397521.522	3300125.944
Black-necked Crane	-10397973.603	3303748.839
Black-necked Crane	-10363436.731	3303429.317
Black-necked Crane	-10347018.652	3314738.899
