# Supplementary material for: Why choose Random Forest to predict rare species distribution with few samples in large undersampled areas? Three Asian crane species models provide supporting evidence
Source: PeerJ. 2017 Jan 12;5:e2849. doi: 10.7717/peerj.2849 (PMC5237372; doi:10.7717/peerj.2849)
Supplement: Supplemental Information 3 [file peerj-05-2849-s005.pdf]

Species	X	Y
Hooded Crane	-5149269.912	8337727.926
Hooded Crane	-5215490.008	8264306.159
Hooded Crane	-5350579.006	8163245.022
Hooded Crane	-5420772.309	8142684.375
Hooded Crane	-5493614.415	8117063.617
Hooded Crane	-5557185.708	8050860.992
Hooded Crane	-6305119.629	8648728.475
Hooded Crane	-6312536.280	8688219.834
Hooded Crane	-5104416.833	7202863.745
Hooded Crane	-4985220.658	7266611.894
Hooded Crane	-4536248.401	6972732.579
Hooded Crane	-4481947.922	6807122.344
Hooded Crane	-4438242.658	6697881.302
Hooded Crane	-4803777.593	6337890.620
Hooded Crane	-4737557.496	6527358.147
Hooded Crane	-4810399.602	7092542.512
Hooded Crane	-4879268.503	7001409.844
Hooded Crane	-4805101.995	6854709.193
Hooded Crane	-4856753.670	6700009.098
Hooded Crane	-4785235.966	6627976.248
Hooded Crane	-4873970.895	6815753.465
Hooded Crane	-4715042.663	5841740.168
Hooded Crane	-4752125.917	5895681.711
Hooded Crane	-4953435.012	5868669.017
Hooded Crane	-5075279.990	5824472.696
Hooded Crane	-5017006.305	5834061.508
Hooded Crane	-5073955.588	5895681.711
Hooded Crane	-9320771.797	8723557.370
Hooded Crane	-9875431.328	6983634.516
White-naped Crane	-4883415.148	6278093.693
White-naped Crane	-5003141.083	6149709.288
White-naped Crane	-5096378.980	6127441.880
White-naped Crane	-5250440.035	5857165.063
White-naped Crane	-5216005.585	5938349.090
White-naped Crane	-5153758.694	6041114.012
White-naped Crane	-5289642.333	6086440.394
White-naped Crane	-5443537.838	5939125.951
White-naped Crane	-5824762.243	6559340.609
White-naped Crane	-5864118.018	6504579.965
White-naped Crane	-5830791.473	6376030.929
White-naped Crane	-5765693.917	6355258.695
White-naped Crane	-5736027.314	6503064.064
White-naped Crane	-5803571.812	6449234.596
White-naped Crane	-5696560.136	6399035.881
White-naped Crane	-5638021.570	6445108.429
White-naped Crane	-5600143.675	6318614.798
White-naped Crane	-5557762.813	6215255.128
White-naped Crane	-5512203.386	6211243.417
White-naped Crane	-5310894.292	5592426.269
White-naped Crane	-5276459.841	5642976.553
White-naped Crane	-6197175.239	5903598.655
White-naped Crane	-6398484.334	5586404.328
White-naped Crane	-6811697.738	6197564.516
White-naped Crane	-6979102.143	6207180.170
White-naped Crane	-7083041.207	6761906.950
White-naped Crane	-7316301.498	6356591.778
White-naped Crane	-7250346.282	6249539.777

White-naped Crane	-7170882.166	6402017.325
White-naped Crane	-7205051.736	6372523.730
White-naped Crane	-7229685.612	6329699.494
White-naped Crane	-7730786.329	5963399.951
White-naped Crane	-7804687.957	6036163.121
White-naped Crane	-7847598.580	6134553.815
White-naped Crane	-7646554.365	6293431.025
White-naped Crane	-7679134.653	6152457.706
White-naped Crane	-7722354.303	6173713.093
White-naped Crane	-7778641.385	6184700.746
White-naped Crane	-7811089.233	6154768.081
White-naped Crane	-8609703.601	6029866.460
White-naped Crane	-6964399.074	5158944.876
White-naped Crane	-7055120.607	5326024.927
Black-necked Crane	-11422278.870	4100547.792
Black-necked Crane	-11167993.700	3924871.144
Black-necked Crane	-11341556.570	4007036.776
Black-necked Crane	-11311095.330	3971704.647
Black-necked Crane	-11285600.590	3944783.464
Black-necked Crane	-11353476.190	3902944.557
Black-necked Crane	-11303855.260	3843070.070
Black-necked Crane	-11236575.650	3858130.640
Black-necked Crane	-11263063.680	3893978.310
Black-necked Crane	-11136419.960	3765495.328
Black-necked Crane	-11012601.620	3792918.654
Black-necked Crane	-10666575.130	3769081.872
Black-necked Crane	-10530294.170	3651081.117
Black-necked Crane	-10174493.590	3595674.406
Black-necked Crane	-10671059.330	3433693.030
Black-necked Crane	-10535016.760	3399543.080
Black-necked Crane	-10548154.830	3351499.788
Black-necked Crane	-10335862.030	3378326.021
Black-necked Crane	-10281296.670	3365051.685
Black-necked Crane	-10237326.520	3336738.417
Black-necked Crane	-10213752.170	3396751.745
Black-necked Crane	-10145677.910	3383458.405
Black-necked Crane	-10100913.130	3369877.136
Black-necked Crane	-10126341.640	3431864.134
Black-necked Crane	-10034587.080	3705425.351
Black-necked Crane	-9796724.488	3669787.694
Black-necked Crane	-9828355.621	3780774.340
Black-necked Crane	-9961597.078	3573147.363
Black-necked Crane	-9897694.684	3544441.394
Black-necked Crane	-9867895.641	3505888.765
Black-necked Crane	-9821541.573	3454922.446
Black-necked Crane	-9861273.631	3448471.278
Black-necked Crane	-9899681.287	3438232.052
Black-necked Crane	-9941399.948	3389046.981
Black-necked Crane	-9860942.531	3375082.604
Black-necked Crane	-9875179.851	3412102.547
Black-necked Crane	-9812270.759	3379232.593
Black-necked Crane	-10124984.130	4405538.119
Black-necked Crane	-11284555.420	3878983.419
Black-necked Crane	-11253862.400	3845142.092
Black-necked Crane	-8897923.528	4391451.129
Black-necked Crane	-9468343.442	4313764.970
Black-necked Crane	-9299879.516	4318182.315
Black-necked Crane	-9214381.093	4113144.946

Black-necked Crane	-9115713.149	4082679.017
Black-necked Crane	-9097171.522	4121979.747
Black-necked Crane	-9222989.706	3967186.666
Black-necked Crane	-9291196.406	3893728.721
Black-necked Crane	-9315697.841	3779343.624
Black-necked Crane	-8569671.059	3938363.210
Black-necked Crane	-8495749.565	3809158.271
Black-necked Crane	-10975194.990	3561501.916
Black-necked Crane	-11044394.990	3627525.901
