# Supplementary material for: Why choose Random Forest to predict rare species distribution with few samples in large undersampled areas? Three Asian crane species models provide supporting evidence
Source: PeerJ. 2017 Jan 12;5:e2849. doi: 10.7717/peerj.2849 (PMC5237372; doi:10.7717/peerj.2849)
Supplement: Supplemental Information 4 [file peerj-05-2849-s006.pdf]

Species	X	Y
Hooded Crane	-4812704.489	6737836.289
Hooded Crane	-5103804.957	7213595.239
Hooded Crane	-4812165.702	6737025.829
Hooded Crane	-5100340.694	7214352.933
Hooded Crane	-5101133.289	7211522.932
Hooded Crane	-5101881.356	7212459.791
Hooded Crane	-5101204.534	7208270.56
Hooded Crane	-5018796.941	7273043.067
Hooded Crane	-5100262.771	7214162.071
Hooded Crane	-5098840.108	7213461.648
Hooded Crane	-4812185.74	6737158.804
Hooded Crane	-4812541.962	6735002.731
Hooded Crane	-4812154.57	6736928.795
Hooded Crane	-5101001.932	7212828.078
Hooded Crane	-4812722.3	6736679.028
Hooded Crane	-4985982.182	7283255.694
Hooded Crane	-4812159.023	6736989.89
Hooded Crane	-4812179.061	6737085.128
Hooded Crane	-5105730.784	7227969.625
Hooded Crane	-4813094.107	6735252.447
Hooded Crane	-4812653.282	6736274.744
Hooded Crane	-5100347.374	7209542.734
Hooded Crane	-4810386.817	6737581.102
Hooded Crane	-5015443.998	7273689.257
Hooded Crane	-4812225.815	6737076.143
Hooded Crane	-4812196.872	6737110.286
Hooded Crane	-4812141.212	6737024.032
Hooded Crane	-4812450.68	6736290.915
Hooded Crane	-4812744.564	6736849.731
Hooded Crane	-5020687.146	7268951.782
Hooded Crane	-5099982.246	7209609.495
Hooded Crane	-4810771.982	6737155.21
Hooded Crane	-5016018.407	7268494.338
Hooded Crane	-4810932.282	6737277.404
Hooded Crane	-4814116.02	6735176.993
Hooded Crane	-4817553.566	6741183.219
Hooded Crane	-5093507.904	7222910.253
Hooded Crane	-4812435.095	6735288.378
Hooded Crane	-5102268.748	7215011.442
Hooded Crane	-4814614.731	6734612.903
Hooded Crane	-5093527.942	7223273.302
Hooded Crane	-4978975.733	7271195.185
Hooded Crane	-5020286.396	7269799.47
Hooded Crane	-5101847.96	7213478.824
Hooded Crane	-4812132.306	6737040.205
Hooded Crane	-5017904.159	7270260.836
Hooded Crane	-4812234.72	6737092.316
Hooded Crane	-5016661.833	7269069.031
Hooded Crane	-4812156.797	6737031.22
Hooded Crane	-4810124.103	6736970.124
Hooded Crane	-4812063.288	6737216.307
Hooded Crane	-4812156.797	6737056.377
Hooded Crane	-5019139.805	7269628.387
Hooded Crane	-4812058.835	6735383.596
Hooded Crane	-4812116.722	6737038.408
Hooded Crane	-5093242.964	7222877.771
Hooded Crane	-5004964.381	7268811.471

Hooded Crane	-4973888.432	7259291.605
Hooded Crane	-5017213.978	7269755.257
Hooded Crane	-5004968.834	7268805.705
Hooded Crane	-5005402.98	7268840.302
Hooded Crane	-5019257.804	7270539.591
Hooded Crane	-4809968.255	6737561.334
Hooded Crane	-4812181.287	6737036.611
Hooded Crane	-4812167.929	6737106.692
Hooded Crane	-4812163.476	6737074.346
Hooded Crane	-4812216.909	6735804.004
Hooded Crane	-5018565.396	7269526.508
Hooded Crane	-5102506.972	7211732.809
Hooded Crane	-4812154.57	6737013.25
Hooded Crane	-4812156.797	6737092.316
Hooded Crane	-4812154.57	6737074.346
Hooded Crane	-5100351.826	7212305.23
Hooded Crane	-4809921.501	6737487.655
Hooded Crane	-4812147.891	6737013.25
Hooded Crane	-5017429.938	7269357.354
Hooded Crane	-4812078.873	6735895.635
Hooded Crane	-5019870.061	7272233.483
Hooded Crane	-5100325.11	7214341.481
Hooded Crane	-5005774.787	7263258.718
Hooded Crane	-5016748.662	7266639.852
Hooded Crane	-4809859.162	6737489.452
Hooded Crane	-5094565.439	7220506.914
Hooded Crane	-4812550.868	6734986.563
Hooded Crane	-5004944.344	7268824.925
Hooded Crane	-5016394.666	7268921.029
Hooded Crane	-5017830.688	7270033.994
Hooded Crane	-4812150.117	6735715.968
Hooded Crane	-4812181.287	6737047.392
Hooded Crane	-4812161.249	6737059.971
Hooded Crane	-5016842.171	7272654.61
Hooded Crane	-4813062.937	6734857.217
Hooded Crane	-4812161.249	6737083.331
Hooded Crane	-4812123.401	6737058.174
Hooded Crane	-4812562	6735534.509
Hooded Crane	-5016332.327	7272271.941
Hooded Crane	-5017423.258	7265953.901
Hooded Crane	-5004953.249	7268803.783
Hooded Crane	-5004951.023	7268807.627
Hooded Crane	-5016254.404	7270158.948
Hooded Crane	-5102190.824	7215074.433
Hooded Crane	-5016606.173	7275495.411
Hooded Crane	-4812150.117	6737088.722
Hooded Crane	-5016715.267	7268603.892
Hooded Crane	-5016434.741	7272233.483
Hooded Crane	-5017621.407	7265519.689
Hooded Crane	-5016630.664	7269197.814
Hooded Crane	-5103379.717	7215578.377
Hooded Crane	-4812167.929	6737065.362
Hooded Crane	-5017178.356	7269718.734
Hooded Crane	-5101925.884	7212467.424
Hooded Crane	-4810627.267	6737295.374
Hooded Crane	-5100278.355	7208968.609
Hooded Crane	-5016690.776	7272104.65
Hooded Crane	-4810616.135	6737257.637

Hooded Crane	-5015711.165	7274866.383
Hooded Crane	-5016254.404	7269962.867
Hooded Crane	-5016457.005	7268676.928
Hooded Crane	-4812023.213	6735489.594
Hooded Crane	-4812105.59	6736984.5
Hooded Crane	-5020517.94	7273291.152
Hooded Crane	-5016042.897	7268705.759
Hooded Crane	-4813461.461	6734744.042
Hooded Crane	-4812372.756	6735694.408
Hooded Crane	-5015510.79	7268573.14
Hooded Crane	-4811958.648	6735629.73
Hooded Crane	-5019469.311	7270360.802
Hooded Crane	-5016632.89	7273756.571
Hooded Crane	-5016813.228	7270007.081
Hooded Crane	-4813074.069	6735259.633
Hooded Crane	-4812192.419	6737088.722
Hooded Crane	-5023414.473	7269664.91
Hooded Crane	-5018937.204	7273087.299
Hooded Crane	-5016748.662	7269094.018
Hooded Crane	-5015584.261	7273898.895
Hooded Crane	-4963025.876	7249511.133
Hooded Crane	-4812134.533	6737126.458
Hooded Crane	-5018458.53	7273468.085
Hooded Crane	-4812626.565	6735671.052
Hooded Crane	-5016136.405	7273820.04
Hooded Crane	-5015492.979	7266197.916
Hooded Crane	-5106173.836	7214702.222
Hooded Crane	-5015150.114	7269440.008
Hooded Crane	-5017830.688	7270255.068
Hooded Crane	-5019507.159	7275162.616
Hooded Crane	-5102146.297	7215311.13
Hooded Crane	-5018235.891	7269499.597
Hooded Crane	-5012939.309	7279738.354
Hooded Crane	-4811255.109	6737002.469
Hooded Crane	-4814140.51	6734842.846
Hooded Crane	-5009152.22	7266368.923
Hooded Crane	-4812165.702	6737076.143
Hooded Crane	-4812172.381	6737067.159
Hooded Crane	-5019440.368	7270360.802
Hooded Crane	-5004975.513	7268815.315
Hooded Crane	-5005910.597	7269119.006
Hooded Crane	-5017810.65	7270095.51
Hooded Crane	-4963166.139	7249361.586
Hooded Crane	-5018389.512	7268628.878
Hooded Crane	-5019280.068	7272969.99
Hooded Crane	-5015942.709	7273887.356
Hooded Crane	-5013520.397	7288468.218
Hooded Crane	-4813332.33	6735115.911
Hooded Crane	-4811308.542	6736700.59
Hooded Crane	-5102083.958	7212818.536
Hooded Crane	-5004964.381	7268650.02
Hooded Crane	-4812165.702	6737094.113
Hooded Crane	-5016666.286	7266238.266
Hooded Crane	-4814222.886	6734431.469
Hooded Crane	-4812069.967	6735883.058
Hooded Crane	-4810228.743	6736238.809
Hooded Crane	-5017959.818	7275772.431
Hooded Crane	-4810371.232	6737523.596

Hooded Crane	-4814307.489	6735556.068
Hooded Crane	-4812152.344	6737090.519
Hooded Crane	-5016174.254	7266169.095
Hooded Crane	-5021199.216	7271512.428
Hooded Crane	-4812159.023	6737095.91
Hooded Crane	-5017162.771	7269749.49
Hooded Crane	-5016615.079	7271962.359
Hooded Crane	-5018084.496	7271870.063
Hooded Crane	-5018013.252	7268213.735
Hooded Crane	-5016209.876	7272594.997
Hooded Crane	-5018705.659	7270362.724
Hooded Crane	-5018373.927	7273339.231
Hooded Crane	-5018607.698	7269386.187
Hooded Crane	-4810898.886	6736982.703
Hooded Crane	-5017768.349	7266199.837
Hooded Crane	-5016318.969	7269332.365
Hooded Crane	-5018609.924	7269370.809
Hooded Crane	-5018721.244	7272679.609
Hooded Crane	-4810471.42	6737588.29
Hooded Crane	-5015395.017	7277569.449
Hooded Crane	-4812495.208	6736154.361
Hooded Crane	-5099254.216	7212448.342
Hooded Crane	-5017278.543	7267358.533
Hooded Crane	-5016719.719	7266576.443
Hooded Crane	-5016027.312	7268671.162
Hooded Crane	-4812617.659	6736991.687
Hooded Crane	-4812147.891	6737068.956
Hooded Crane	-5014361.973	7283796.817
Hooded Crane	-5018897.129	7269451.542
Hooded Crane	-5016372.403	7271983.51
Hooded Crane	-4810458.061	6737636.811
Hooded Crane	-4812132.306	6737090.519
Hooded Crane	-4991519.213	7287208.107
Hooded Crane	-5103330.736	7215206.142
Hooded Crane	-4812236.947	6735926.178
Hooded Crane	-4871679.328	6903821.009
Hooded Crane	-4812163.476	6737056.377
Hooded Crane	-5019184.333	7273123.838
Hooded Crane	-4812147.891	6735811.191
Hooded Crane	-5019148.711	7274416.284
Hooded Crane	-4812147.891	6737104.895
Hooded Crane	-4812323.776	6736573.013
Hooded Crane	-5100785.972	7214664.048
Hooded Crane	-5018832.563	7269345.821
Hooded Crane	-5100109.15	7209346.268
Hooded Crane	-5021526.495	7272698.839
Hooded Crane	-4812159.023	6737095.91
Hooded Crane	-4814271.867	6734806.917
Hooded Crane	-5004935.438	7268798.016
Hooded Crane	-5017808.424	7268832.614
Hooded Crane	-5015232.491	7281047.304
Hooded Crane	-4812147.891	6737052.783
Hooded Crane	-5019119.768	7273104.607
Hooded Crane	-5018988.411	7272893.067
Hooded Crane	-4812833.619	6737006.063
Hooded Crane	-5019079.693	7273177.686
Hooded Crane	-5018344.984	7272589.228
Hooded Crane	-5015766.824	7273727.722

Hooded Crane	-5017144.96	7269566.875
Hooded Crane	-5019471.537	7275151.075
Hooded Crane	-5017167.224	7271222.102
Hooded Crane	-4812138.986	6737108.489
Hooded Crane	-5023806.318	7269388.109
Hooded Crane	-5099545.873	7211171.878
Hooded Crane	-5074047.031	7240451.49
Hooded Crane	-4812143.438	6737076.143
Hooded Crane	-5023394.436	7269620.698
Hooded Crane	-5018144.609	7268161.843
Hooded Crane	-5015833.616	7273681.564
Hooded Crane	-5017098.206	7269553.42
Hooded Crane	-4812138.986	6737058.174
Hooded Crane	-5018436.266	7273439.237
Hooded Crane	-5016027.312	7268594.282
Hooded Crane	-5019068.561	7273044.99
Hooded Crane	-5101208.986	7214973.266
Hooded Crane	-4812112.269	6736979.109
Hooded Crane	-4812147.891	6737113.879
Hooded Crane	-5019010.674	7273760.418
Hooded Crane	-4812156.797	6737054.58
Hooded Crane	-5019442.594	7270331.965
Hooded Crane	-4809966.029	6737451.715
Hooded Crane	-5019244.445	7273852.736
Hooded Crane	-5022154.337	7270478.071
Hooded Crane	-5020232.962	7273294.998
Hooded Crane	-4812163.476	6737063.565
Hooded Crane	-5016730.851	7266699.42
Hooded Crane	-5019070.787	7272943.067
Hooded Crane	-5016630.664	7271870.063
Hooded Crane	-4813042.9	6734945.244
Hooded Crane	-5015570.902	7273760.418
Hooded Crane	-4810355.647	6737557.74
Hooded Crane	-4812141.212	6737079.737
Hooded Crane	-4812154.57	6737076.143
Hooded Crane	-5019382.482	7272829.606
Hooded Crane	-5016009.501	7271131.738
Hooded Crane	-5019705.308	7270041.684
Hooded Crane	-5017149.413	7268156.078
Hooded Crane	-5015185.737	7268417.46
Hooded Crane	-4814069.265	6735135.673
Hooded Crane	-4814225.113	6734530.269
Hooded Crane	-5017465.56	7268926.795
Hooded Crane	-4870490.436	6904516.425
Hooded Crane	-5016160.895	7269270.856
Hooded Crane	-5015875.918	7272677.686
Hooded Crane	-4810188.668	6737473.279
Hooded Crane	-4813105.239	6734920.094
Hooded Crane	-5100267.224	7214297.582
Hooded Crane	-5017118.243	7269545.731
Hooded Crane	-5015806.899	7273685.41
Hooded Crane	-5100282.808	7208781.693
Hooded Crane	-5017291.901	7269826.382
Hooded Crane	-4812152.344	6737085.128
Hooded Crane	-5017365.372	7268907.574
Hooded Crane	-5016902.283	7271883.523
Hooded Crane	-5016123.047	7272391.163
Hooded Crane	-5020544.657	7272729.608

Hooded Crane	-5016817.68	7270066.674
Hooded Crane	-5016697.455	7275655.081
Hooded Crane	-4812156.797	6735841.734
Hooded Crane	-5018338.305	7268480.884
Hooded Crane	-5011648.003	7277242.338
Hooded Crane	-5015208.001	7269403.486
Hooded Crane	-5016174.254	7271852.758
Hooded Crane	-4812161.249	6737079.737
Hooded Crane	-4812159.023	6737068.956
Hooded Crane	-5101997.128	7212660.152
Hooded Crane	-5016180.933	7266169.095
Hooded Crane	-4813143.087	6735410.544
Hooded Crane	-5015831.39	7273954.672
Hooded Crane	-5020655.977	7273235.38
Hooded Crane	-5017051.451	7269737.957
Hooded Crane	-4812161.249	6737058.174
Hooded Crane	-5018545.359	7272756.53
Hooded Crane	-5018547.585	7271898.905
Hooded Crane	-4812138.986	6737086.925
Hooded Crane	-4809859.162	6737457.106
Hooded Crane	-5100445.335	7208835.097
Hooded Crane	-4814741.635	6734788.952
Hooded Crane	-5100966.31	7212875.785
Hooded Crane	-5019716.44	7270045.528
Hooded Crane	-4872017.74	6903894.401
Hooded Crane	-5006324.705	7270258.913
Hooded Crane	-5017405.447	7268223.344
Hooded Crane	-4814162.774	6734779.97
Hooded Crane	-5016908.962	7268377.099
Hooded Crane	-4812152.344	6737033.017
Hooded Crane	-5016485.948	7269332.365
Hooded Crane	-4812156.797	6737059.971
Hooded Crane	-5017877.442	7270203.163
Hooded Crane	-5015840.295	7271435.52
Hooded Crane	-5016296.705	7272425.776
Hooded Crane	-5015513.016	7273758.495
Hooded Crane	-5101155.553	7214938.908
Hooded Crane	-5016859.982	7269318.91
Hooded Crane	-5015804.673	7271429.751
Hooded Crane	-4812154.57	6737094.113
Hooded Crane	-4812145.665	6737090.519
Hooded Crane	-5020698.278	7269013.289
Hooded Crane	-5016492.628	7267252.841
Hooded Crane	-4812147.891	6737094.113
Hooded Crane	-5018438.492	7273464.238
Hooded Crane	-4812145.665	6737074.346
Hooded Crane	-5100405.26	7211889.267
Hooded Crane	-5015446.224	7273827.733
Hooded Crane	-4812156.797	6737099.504
Hooded Crane	-5018487.473	7269464.997
Hooded Crane	-5016730.851	7266687.891
Hooded Crane	-5015642.147	7273889.279
Hooded Crane	-5019738.704	7270133.957
Hooded Crane	-5015606.524	7273902.742
Hooded Crane	-4809634.297	6737397.804
Hooded Crane	-4812047.704	6735403.358
Hooded Crane	-4812372.756	6736163.345
Hooded Crane	-5018507.51	7269468.842

Hooded Crane	-4854680.842	6956939.289
Hooded Crane	-5106659.189	7212351.025
Hooded Crane	-4813710.817	6735018.9
Hooded Crane	-5015838.069	7273679.641
Hooded Crane	-4809892.558	6737415.774
Hooded Crane	-5015301.509	7287057.832
Hooded Crane	-4812239.173	6737103.098
Hooded Crane	-5023797.413	7269343.898
Hooded Crane	-5015610.977	7274064.302
Hooded Crane	-5018071.138	7268148.39
Hooded Crane	-5016739.757	7266691.734
Hooded Crane	-5017697.104	7270064.752
Hooded Crane	-4991668.381	7286021.401
Hooded Crane	-4812159.023	6737088.722
Hooded Crane	-5106543.416	7212102.97
Hooded Crane	-4812210.23	6737058.174
Hooded Crane	-4811969.78	6735412.341
Hooded Crane	-5017160.544	7268121.484
Hooded Crane	-5017405.447	7272123.878
Hooded Crane	-5015751.24	7273708.49
Hooded Crane	-4809819.087	6737708.694
Hooded Crane	-5013694.056	7278387.288
Hooded Crane	-4812691.13	6735189.568
Hooded Crane	-5014927.476	7281053.079
Hooded Crane	-5015742.334	7273702.72
Hooded Crane	-5018672.263	7274447.06
Hooded Crane	-4813298.935	6735133.876
Hooded Crane	-5020028.135	7272212.331
Hooded Crane	-5019280.068	7273002.682
Hooded Crane	-5019718.666	7270045.528
Hooded Crane	-5015573.129	7273898.895
Hooded Crane	-5019444.82	7275029.887
Hooded Crane	-5017202.846	7269743.724
Hooded Crane	-4810938.961	6736973.718
Hooded Crane	-5016713.04	7269372.731
Hooded Crane	-5100734.765	7207955.886
Hooded Crane	-5093483.414	7223173.94
Hooded Crane	-5099634.929	7210044.413
Hooded Crane	-5017826.235	7270062.83
Hooded Crane	-5104586.42	7213079.973
Hooded Crane	-4812150.117	6737067.159
Hooded Crane	-4812121.174	6737092.316
Hooded Crane	-5017156.092	7269674.522
Hooded Crane	-5017196.167	7269753.335
Hooded Crane	-4810638.399	6737252.246
Hooded Crane	-5101725.509	7212539.935
Hooded Crane	-5017921.97	7270205.086
Hooded Crane	-4812161.249	6737061.768
Hooded Crane	-5019266.709	7273237.303
Hooded Crane	-5016454.779	7269378.498
Hooded Crane	-5015619.883	7269807.159
Hooded Crane	-5018741.281	7271941.207
Hooded Crane	-5017138.281	7270316.586
Hooded Crane	-4812167.929	6737065.362
Hooded Crane	-5018654.452	7272971.913
Hooded Crane	-5019854.476	7270033.994
Hooded Crane	-5100151.451	7208598.597
Hooded Crane	-4812354.945	6737068.956

Hooded Crane	-5021880.491	7273785.421
Hooded Crane	-5016741.983	7266691.734
Hooded Crane	-5102132.938	7212793.729
Hooded Crane	-4812179.061	6737068.956
Hooded Crane	-5016775.379	7272764.222
Hooded Crane	-4812190.192	6737103.098
Hooded Crane	-4812154.57	6737054.58
Hooded Crane	-5015610.977	7271668.17
Hooded Crane	-5099652.74	7211137.536
Hooded Crane	-5018494.152	7269366.965
Hooded Crane	-4812163.476	6737090.519
Hooded Crane	-4812138.986	6737047.392
Hooded Crane	-5018877.091	7272485.387
Hooded Crane	-5018467.435	7273471.931
Hooded Crane	-5018881.544	7273854.659
Hooded Crane	-4812163.476	6737074.346
Hooded Crane	-5020684.92	7273231.534
Hooded Crane	-5017563.521	7269812.926
Hooded Crane	-4814245.15	6734528.473
Hooded Crane	-5016960.169	7268984.458
Hooded Crane	-4814345.338	6734790.749
Hooded Crane	-4812159.023	6737072.55
Hooded Crane	-5016367.95	7268901.808
Hooded Crane	-4813310.067	6735092.556
Hooded Crane	-4812012.081	6735437.493
Hooded Crane	-5018044.421	7272043.118
Hooded Crane	-4812020.987	6735439.289
Hooded Crane	-5014671.441	7280795.121
Hooded Crane	-5013084.025	7279143.625
Hooded Crane	-5023801.865	7269351.587
Hooded Crane	-4810397.949	6737505.626
Hooded Crane	-5019219.955	7270593.42
Hooded Crane	-5093394.358	7223001.969
Hooded Crane	-5074033.672	7240371.058
Hooded Crane	-5074323.103	7240485.961
Hooded Crane	-4812152.344	6737070.753
Hooded Crane	-5020684.92	7269113.239
Hooded Crane	-5021439.666	7268946.016
Hooded Crane	-5100647.936	7214059.008
Hooded Crane	-5015455.13	7273989.292
Hooded Crane	-4810958.999	6736928.795
Hooded Crane	-5017739.406	7268690.382
Hooded Crane	-5016844.397	7266883.889
Hooded Crane	-5023049.346	7272744.992
Hooded Crane	-4814229.566	6735457.255
Hooded Crane	-4816088.601	6734803.324
Hooded Crane	-4814291.904	6734727.874
Hooded Crane	-4813882.249	6734611.107
Hooded Crane	-4811831.744	6737570.32
Hooded Crane	-5020324.244	7269445.775
Hooded Crane	-5016739.757	7269020.978
Hooded Crane	-5020691.599	7272179.642
Hooded Crane	-4812145.665	6737099.504
Hooded Crane	-5016750.889	7268997.912
Hooded Crane	-4813786.514	6733281.882
Hooded Crane	-4812159.023	6737088.722
Hooded Crane	-4810571.607	6737239.667
Hooded Crane	-5016670.739	7269109.395

Hooded Crane	-4812147.891	6737072.55
Hooded Crane	-5019177.654	7274456.677
Hooded Crane	-5015960.52	7268640.41
Hooded Crane	-4812181.287	6737119.27
Hooded Crane	-4812755.696	6735112.318
Hooded Crane	-5014430.991	7290655.593
Hooded Crane	-5017009.15	7272771.914
Hooded Crane	-5016138.632	7272604.612
Hooded Crane	-5020740.579	7274750.971
Hooded Crane	-5100251.639	7214322.395
Hooded Crane	-4811183.864	6736959.343
Hooded Crane	-4812172.381	6737022.235
Hooded Crane	-5016766.474	7270049.373
Hooded Crane	-4810284.403	6737579.305
Hooded Crane	-5016824.36	7270070.519
Hooded Crane	-5016824.36	7270080.131
Hooded Crane	-5019431.462	7270358.879
Hooded Crane	-5107665.517	7211492.405
Hooded Crane	-5017055.904	7272741.146
Hooded Crane	-5015795.768	7273798.884
Hooded Crane	-5019716.44	7270064.752
Hooded Crane	-5106659.189	7212253.71
Hooded Crane	-5020700.504	7269074.797
Hooded Crane	-4810435.797	6737593.681
Hooded Crane	-5074055.936	7240428.51
Hooded Crane	-5014631.366	7280758.546
Hooded Crane	-5023786.281	7269365.042
Hooded Crane	-5018678.942	7272993.067
Hooded Crane	-5015766.824	7273866.199
Hooded Crane	-5015524.148	7273898.895
Hooded Crane	-5021134.65	7268753.809
Hooded Crane	-5017803.971	7270043.606
Hooded Crane	-5015844.748	7272581.536
Hooded Crane	-4814127.152	6734837.456
Hooded Crane	-4871674.876	6903841.191
Hooded Crane	-5073978.013	7223944.032
Hooded Crane	-4812288.154	6736546.061
Hooded Crane	-5099392.252	7212908.225
Hooded Crane	-5018503.058	7269530.353
Hooded Crane	-5023403.342	7269653.377
Hooded Crane	-5014364.199	7283798.743
Hooded Crane	-5103929.635	7214755.666
Hooded Crane	-4812172.381	6735706.985
Hooded Crane	-5017249.6	7268108.031
Hooded Crane	-5016735.304	7266712.87
Hooded Crane	-5101090.988	7214291.857
Hooded Crane	-4809629.844	6737421.165
Hooded Crane	-5097958.457	7217092.314
Hooded Crane	-4812444.001	6736339.428
Hooded Crane	-4810901.113	6737221.698
Hooded Crane	-5071310.798	7218150.145
Hooded Crane	-4809536.336	6737877.623
Hooded Crane	-5015446.224	7274008.525
Hooded Crane	-5015624.336	7273627.713
Hooded Crane	-5014680.346	7280804.746
Hooded Crane	-4810335.61	6737403.195
Hooded Crane	-5017797.292	7270041.684
Hooded Crane	-5019460.405	7275179.929

Hooded Crane	-5016608.4	7272877.682
Hooded Crane	-5019186.559	7270389.638
Hooded Crane	-5018053.327	7272039.272
Hooded Crane	-5015833.616	7273668.101
Hooded Crane	-5015608.751	7273885.432
Hooded Crane	-5017679.293	7270074.364
Hooded Crane	-5019119.768	7273925.822
Hooded Crane	-5016684.097	7269318.91
Hooded Crane	-5018304.909	7269559.186
Hooded Crane	-5017799.518	7270047.451
Hooded Crane	-5093436.66	7223212.156
Hooded Crane	-4866474.029	6905206.397
Hooded Crane	-4812421.737	6736350.209
Hooded Crane	-5014315.218	7283423.226
Hooded Crane	-5021911.66	7272414.238
Hooded Crane	-4813051.805	6735468.035
Hooded Crane	-5020239.642	7275024.116
Hooded Crane	-4812090.005	6737103.098
Hooded Crane	-5018534.227	7271835.452
Hooded Crane	-4813953.493	6734736.856
Hooded Crane	-4812165.702	6737092.316
Hooded Crane	-5019226.634	7273858.506
Hooded Crane	-4812143.438	6737040.205
Hooded Crane	-5019228.861	7274554.774
Hooded Crane	-5015993.916	7272621.919
Hooded Crane	-4812092.231	6735924.382
Hooded Crane	-4812156.797	6737067.159
Hooded Crane	-4814089.303	6735281.192
Hooded Crane	-5018667.81	7273035.375
Hooded Crane	-4812633.244	6735335.088
Hooded Crane	-4812887.053	6735139.266
Hooded Crane	-5015829.163	7273670.024
Hooded Crane	-5016508.212	7269332.365
Hooded Crane	-4814360.923	6734776.377
Hooded Crane	-5100979.668	7212826.17
Hooded Crane	-5100302.846	7214320.486
Hooded Crane	-5015853.654	7272575.767
Hooded Crane	-4810415.76	6737586.493
Hooded Crane	-5019206.597	7270601.11
Hooded Crane	-4812143.438	6737068.956
Hooded Crane	-5018356.116	7269436.164
Hooded Crane	-5023394.436	7269651.454
Hooded Crane	-5014375.331	7283798.743
Hooded Crane	-5018507.51	7269566.875
Hooded Crane	-5017881.895	7272762.299
Hooded Crane	-4814082.624	6734837.456
Hooded Crane	-5101006.385	7212906.317
Hooded Crane	-4810230.969	6736298.102
Hooded Crane	-4812147.891	6737108.489
Hooded Crane	-5016819.907	7270070.519
Hooded Crane	-5015691.127	7272406.546
Hooded Crane	-5090299.676	7230673.637
Hooded Crane	-5019297.879	7273043.067
Hooded Crane	-4811998.723	6735396.172
Hooded Crane	-5016815.454	7270066.674
Hooded Crane	-5016808.775	7270033.994
Hooded Crane	-5094594.382	7222560.596
Hooded Crane	-4811259.562	6735671.052

Hooded Crane	-4814265.188	6735539.899
Hooded Crane	-5017657.029	7270022.46
Hooded Crane	-4810892.207	6736659.262
Hooded Crane	-4812481.85	6736012.42
Hooded Crane	-5018151.288	7273673.871
Hooded Crane	-5020829.635	7274752.894
Hooded Crane	-4812633.244	6735114.114
Hooded Crane	-5101095.441	7214318.577
Hooded Crane	-5023799.639	7269347.743
Hooded Crane	-5018376.153	7272170.028
Hooded Crane	-5017808.424	7270055.14
Hooded Crane	-5017824.009	7270041.684
Hooded Crane	-5017913.064	7270295.439
Hooded Crane	-5019084.145	7273204.61
Hooded Crane	-4812154.57	6737056.377
Hooded Crane	-4814238.471	6734548.233
Hooded Crane	-5014368.652	7283791.04
Hooded Crane	-4812132.306	6737085.128
Hooded Crane	-4812272.569	6736714.965
Hooded Crane	-4810168.631	6737493.046
Hooded Crane	-4812145.665	6737095.91
Hooded Crane	-4812056.609	6735723.155
Hooded Crane	-5016419.157	7268655.786
Hooded Crane	-4809796.823	6738132.819
Hooded Crane	-5019950.211	7272270.018
Hooded Crane	-5022074.187	7270116.656
Hooded Crane	-4812172.381	6737090.519
Hooded Crane	-5020974.35	7273616.173
Hooded Crane	-5102624.97	7215828.452
Hooded Crane	-5100972.989	7212816.628
Hooded Crane	-4812145.665	6737058.174
Hooded Crane	-5018507.51	7269568.798
Hooded Crane	-4812590.943	6735432.103
Hooded Crane	-5018233.664	7270074.364
Hooded Crane	-5016641.796	7269111.317
Hooded Crane	-5018405.096	7275780.126
Hooded Crane	-5019297.879	7273048.837
Hooded Crane	-5019378.029	7270358.879
Hooded Crane	-4856637.839	6956342.695
Hooded Crane	-5015555.317	7268957.548
Hooded Crane	-5074018.088	7240394.039
Hooded Crane	-4814276.32	6734548.233
Hooded Crane	-4814245.15	6734539.251
Hooded Crane	-5084259.481	7231888.263
Hooded Crane	-4812159.023	6737079.737
Hooded Crane	-5016655.154	7270295.439
Hooded Crane	-5019302.332	7270337.733
Hooded Crane	-5018193.589	7272014.275
Hooded Crane	-4814269.641	6734717.095
Hooded Crane	-4849333.054	6937430.13
Hooded Crane	-4812143.438	6737086.925
Hooded Crane	-5018485.246	7269455.386
Hooded Crane	-5017830.688	7272977.682
Hooded Crane	-4812216.909	6737104.895
Hooded Crane	-4813330.104	6735112.318
Hooded Crane	-5018251.476	7270816.433
Hooded Crane	-5018231.438	7267281.666
Hooded Crane	-4812557.547	6735514.746

Hooded Crane	-4812161.249	6737068.956
Hooded Crane	-5101549.624	7213516.993
Hooded Crane	-5100300.619	7214291.857
Hooded Crane	-5019119.768	7271873.909
Hooded Crane	-4812826.94	6736855.122
Hooded Crane	-5019375.802	7273081.529
Hooded Crane	-5017737.179	7268701.915
Hooded Crane	-5016425.836	7275256.875
Hooded Crane	-5019707.534	7270070.519
Hooded Crane	-5020802.918	7269036.355
Hooded Crane	-4813463.687	6734576.976
Hooded Crane	-4812533.057	6735848.921
Hooded Crane	-4812121.174	6737079.737
Hooded Crane	-5018434.039	7273464.238
Hooded Crane	-4871672.649	6903835.687
Hooded Crane	-5100405.26	7212211.732
Hooded Crane	-5101013.064	7212780.371
Hooded Crane	-5019291.2	7270455.002
Hooded Crane	-4812154.57	6737059.971
Hooded Crane	-4809574.184	6737719.477
Hooded Crane	-5016706.361	7273385.387
Hooded Crane	-5019716.44	7270068.597
Hooded Crane	-5101102.12	7212847.16
Hooded Crane	-5016085.198	7268436.679
Hooded Crane	-5016045.123	7266434.253
Hooded Crane	-4812141.212	6737009.657
Hooded Crane	-5019794.364	7268425.147
Hooded Crane	-4809894.785	6737372.645
Hooded Crane	-5101124.384	7214589.608
Hooded Crane	-4812170.155	6735879.464
Hooded Crane	-4810910.018	6736463.407
Hooded Crane	-5015751.24	7273718.106
Hooded Crane	-4812757.922	6736896.451
Hooded Crane	-5016724.172	7270091.665
Hooded Crane	-4855486.795	6956139.531
Hooded Crane	-4812174.608	6737094.113
Hooded Crane	-5019865.608	7270035.917
Hooded Crane	-5015461.809	7273995.062
Hooded Crane	-5103290.661	7215206.142
Hooded Crane	-5075093.434	7240384.464
Hooded Crane	-5011627.966	7273152.685
Hooded Crane	-4810943.414	6736984.5
Hooded Crane	-4811858.46	6735730.341
Hooded Crane	-4814180.585	6734887.757
Hooded Crane	-4812141.212	6737050.986
Hooded Crane	-5023804.092	7269347.743
Hooded Crane	-5019126.447	7270643.405
Hooded Crane	-4962863.35	7249334.744
Hooded Crane	-4809870.294	6737505.626
Hooded Crane	-5016579.457	7269180.514
Hooded Crane	-5016312.29	7272606.535
Hooded Crane	-5016837.718	7269774.48
Hooded Crane	-4813085.201	6735349.461
Hooded Crane	-5093423.301	7223259.927
Hooded Crane	-4812152.344	6737018.641
Hooded Crane	-5014916.344	7280968.376
Hooded Crane	-5100440.882	7208131.339
Hooded Crane	-4810297.761	6736546.061

Hooded Crane	-5020635.939	7269007.523
Hooded Crane	-5099828.625	7211782.417
Hooded Crane	-5018743.508	7272837.298
Hooded Crane	-4812150.117	6737079.737
Hooded Crane	-4812176.834	6737097.707
Hooded Crane	-5021335.025	7273560.399
Hooded Crane	-5019462.632	7270422.32
Hooded Crane	-5100273.903	7214303.308
Hooded Crane	-5019671.912	7270124.345
Hooded Crane	-4814256.282	6734562.604
Hooded Crane	-5015105.587	7269786.014
Hooded Crane	-5023396.662	7269670.677
Hooded Crane	-5019273.388	7273069.991
Hooded Crane	-5097101.297	7217812.159
Hooded Crane	-4812450.68	6736316.07
Hooded Crane	-5018117.892	7271422.061
Hooded Crane	-4811967.554	6737027.626
Hooded Crane	-5017038.093	7269845.605
Hooded Crane	-5018652.226	7274448.983
Hooded Crane	-4812150.117	6737079.737
Hooded Crane	-4812145.665	6737079.737
Hooded Crane	-5019578.404	7270237.767
Hooded Crane	-5019489.348	7275187.624
Hooded Crane	-5015617.656	7273912.359
Hooded Crane	-4813312.293	6735121.3
Hooded Crane	-4814178.359	6734866.2
Hooded Crane	-4810081.801	6736826.371
Hooded Crane	-5019854.476	7270032.072
Hooded Crane	-5018202.495	7272008.507
Hooded Crane	-4809876.973	6737061.768
Hooded Crane	-4812138.986	6737079.737
Hooded Crane	-5018788.035	7272833.452
Hooded Crane	-5014666.988	7280795.121
Hooded Crane	-4813492.631	6734830.271
Hooded Crane	-5018447.398	7273468.085
Hooded Crane	-4814303.036	6734589.55
Hooded Crane	-4814251.829	6734523.084
Hooded Crane	-5018480.794	7269459.23
Hooded Crane	-4811337.485	6735726.748
Hooded Crane	-5099953.303	7209285.231
Hooded Crane	-4814365.375	6734760.21
Hooded Crane	-4810854.359	6736705.98
Hooded Crane	-4812154.57	6737077.94
Hooded Crane	-4810052.858	6736871.294
Hooded Crane	-5019433.689	7270353.112
Hooded Crane	-5019398.066	7270368.492
Hooded Crane	-5023383.304	7269662.988
Hooded Crane	-5019433.689	7275104.908
Hooded Crane	-5015568.676	7273921.975
Hooded Crane	-4814242.924	6734523.084
Hooded Crane	-4812706.715	6737011.454
Hooded Crane	-5018220.306	7269491.908
Hooded Crane	-5018474.114	7269468.842
Hooded Crane	-4812143.438	6737061.768
Hooded Crane	-5099051.615	7213104.781
Hooded Crane	-5104252.461	7215061.071
Hooded Crane	-5019286.747	7270305.051
Hooded Crane	-4812167.929	6737067.159

Hooded Crane	-5019233.313	7274214.326
Hooded Crane	-5018338.305	7273621.943
Hooded Crane	-4814249.603	6734546.437
Hooded Crane	-4809881.426	6737273.81
Hooded Crane	-5103682.506	7215440.934
Hooded Crane	-4812176.834	6737099.504
Hooded Crane	-5019685.271	7270120.501
Hooded Crane	-4810642.852	6737241.464
Hooded Crane	-5020103.832	7274725.965
Hooded Crane	-5017819.556	7270049.373
Hooded Crane	-4855615.926	6955781.236
Hooded Crane	-5018198.042	7269265.089
Hooded Crane	-5019061.881	7273246.919
Hooded Crane	-4810662.889	6737228.886
Hooded Crane	-4810063.99	6736725.746
Hooded Crane	-5093358.736	7221301.577
Hooded Crane	-5019340.18	7270539.591
Hooded Crane	-5016436.968	7272139.261
Hooded Crane	-4812159.023	6737074.346
Hooded Crane	-5018489.699	7269588.02
Hooded Crane	-4813183.162	6735220.109
Hooded Crane	-4811851.781	6735494.984
Hooded Crane	-5020415.526	7272043.118
Hooded Crane	-4814276.32	6734664.999
Hooded Crane	-5018209.174	7268011.937
Hooded Crane	-4871672.649	6903833.852
Hooded Crane	-5018496.378	7269541.886
Hooded Crane	-5019306.784	7273035.375
Hooded Crane	-5019246.672	7273183.455
Hooded Crane	-5019440.368	7270112.811
Hooded Crane	-4811021.338	6736693.402
Hooded Crane	-5018485.246	7269457.308
Hooded Crane	-5019255.577	7273025.759
Hooded Crane	-5019858.929	7270018.615
Hooded Crane	-5019444.82	7270358.879
Hooded Crane	-5016307.837	7272418.084
Hooded Crane	-4810771.982	6737200.134
Hooded Crane	-5103609.035	7215395.121
Hooded Crane	-5016505.986	7272048.887
Hooded Crane	-5075040.001	7240378.718
Hooded Crane	-4811255.109	6737043.798
Hooded Crane	-4810215.385	6736871.294
Hooded Crane	-4812528.604	6735484.204
Hooded Crane	-4812666.64	6734961.412
Hooded Crane	-4812161.249	6737056.377
Hooded Crane	-5015829.163	7273668.101
Hooded Crane	-4812130.08	6736898.247
Hooded Crane	-4810676.247	6737266.622
Hooded Crane	-4814249.603	6734668.592
Hooded Crane	-5100409.712	7214207.877
Hooded Crane	-5100322.883	7214345.298
Hooded Crane	-5018594.34	7269276.622
Hooded Crane	-5023383.304	7269655.299
Hooded Crane	-5019288.973	7270595.343
Hooded Crane	-5099797.455	7209344.36
Hooded Crane	-5018503.058	7269553.42
Hooded Crane	-5018324.946	7273539.244
Hooded Crane	-4809567.505	6737591.884

Hooded Crane	-5018803.62	7274050.839
Hooded Crane	-5019711.987	7270072.441
Hooded Crane	-5019718.666	7270053.218
Hooded Crane	-5018467.435	7270572.273
Hooded Crane	-5075044.453	7240361.483
Hooded Crane	-5099639.382	7210656.772
Hooded Crane	-4812152.344	6737061.768
Hooded Crane	-4812595.395	6736511.921
Hooded Crane	-4812170.155	6737103.098
Hooded Crane	-5017821.782	7270049.373
Hooded Crane	-4810333.383	6737626.029
Hooded Crane	-4814245.15	6734519.491
Hooded Crane	-4811958.648	6735480.611
Hooded Crane	-4812141.212	6737040.205
Hooded Crane	-4812517.472	6735935.162
Hooded Crane	-5020413.3	7272050.809
Hooded Crane	-5019300.105	7274383.586
Hooded Crane	-4814236.245	6734747.634
Hooded Crane	-4812147.891	6737063.565
Hooded Crane	-5023423.379	7269680.288
Hooded Crane	-4812141.212	6737115.676
Hooded Crane	-5095663.049	7225683.23
Hooded Crane	-4812136.759	6737079.737
Hooded Crane	-5017899.706	7275776.278
Hooded Crane	-5016559.419	7269195.891
Hooded Crane	-4810230.969	6737579.305
Hooded Crane	-5017033.64	7272748.838
Hooded Crane	-5020350.961	7274850.995
Hooded Crane	-5016051.802	7268473.197
Hooded Crane	-5099801.908	7211915.979
Hooded Crane	-4849473.316	6937638.343
Hooded Crane	-5019324.595	7274337.424
Hooded Crane	-5016539.382	7272033.504
Hooded Crane	-5021319.441	7272245.02
Hooded Crane	-4810642.852	6737263.028
Hooded Crane	-5014399.821	7283787.188
Hooded Crane	-4812143.438	6737090.519
Hooded Crane	-5019195.465	7273110.376
Hooded Crane	-5023392.21	7269647.61
Hooded Crane	-4963822.924	7250326.022
Hooded Crane	-4811998.723	6735415.934
Hooded Crane	-5016554.966	7271956.59
Hooded Crane	-4810756.397	6736961.14
Hooded Crane	-5017799.518	7270035.917
Hooded Crane	-5021562.117	7271039.452
Hooded Crane	-5100349.6	7214299.491
Hooded Crane	-5020785.107	7273681.564
Hooded Crane	-5014680.346	7280804.746
Hooded Crane	-5019286.747	7273114.222
Hooded Crane	-5101859.092	7212969.291
Hooded Crane	-5021357.289	7270997.155
Hooded Crane	-4813111.918	6735229.092
Hooded Crane	-4812791.318	6736826.371
Hooded Crane	-5019838.891	7272966.144
Hooded Crane	-5017810.65	7270047.451
Hooded Crane	-5102092.863	7215311.13
Hooded Crane	-5019981.38	7275002.957
Hooded Crane	-5102326.634	7215261.499

Hooded Crane	-5100307.299	7207902.488
Hooded Crane	-5015829.163	7273677.717
Hooded Crane	-4812472.944	6736289.118
Hooded Crane	-4812179.061	6735326.105
Hooded Crane	-5102575.99	7212549.476
Hooded Crane	-5100993.027	7212805.179
Hooded Crane	-5019226.634	7272771.914
Hooded Crane	-5019852.25	7270020.538
Hooded Crane	-5019424.783	7269937.877
Hooded Crane	-4810682.927	6737223.495
Hooded Crane	-5019402.519	7275158.769
Hooded Crane	-4809852.483	6736979.109
Hooded Crane	-5022432.636	7272712.301
Hooded Crane	-4812804.676	6735311.733
Hooded Crane	-4871668.196	6903826.513
Hooded Crane	-5019090.824	7274212.402
Hooded Crane	-4812165.702	6737083.331
Hooded Crane	-5019251.125	7270410.785
Hooded Crane	-4812125.627	6735723.155
Hooded Crane	-4810716.322	6736003.436
Hooded Crane	-5016726.398	7266685.969
Hooded Crane	-5016539.382	7271798.919
Hooded Crane	-4814276.32	6735638.713
Hooded Crane	-5022163.242	7271877.754
Hooded Crane	-5016336.78	7273750.802
Hooded Crane	-4871917.552	6901474.657
Hooded Crane	-5017857.405	7270764.524
Hooded Crane	-5015457.356	7273998.908
Hooded Crane	-4809556.373	6737724.868
Hooded Crane	-5019050.749	7273233.457
Hooded Crane	-4810366.779	6736213.654
Hooded Crane	-4856150.259	6956640.063
Hooded Crane	-5018240.344	7269495.753
Hooded Crane	-5015818.031	7273818.117
Hooded Crane	-5017185.035	7268150.312
Hooded Crane	-5097159.183	7217873.262
Hooded Crane	-4812150.117	6737047.392
Hooded Crane	-5020789.56	7269119.006
Hooded Crane	-4810633.946	6737228.886
Hooded Crane	-4812479.623	6736779.652
Hooded Crane	-5019211.05	7270591.498
Hooded Crane	-5017338.656	7267990.797
Hooded Crane	-5019683.044	7270158.948
Hooded Crane	-4814067.039	6735062.016
Hooded Crane	-4814167.227	6734763.802
Hooded Crane	-5018155.741	7275595.445
Hooded Crane	-5019921.268	7274570.162
Hooded Crane	-5019246.672	7270543.436
Hooded Crane	-5074527.931	7240309.778
Hooded Crane	-4812539.736	6736114.833
Hooded Crane	-5014675.893	7280814.372
Hooded Crane	-4812049.93	6735315.326
Hooded Crane	-5015746.787	7268863.367
Hooded Crane	-5020535.751	7270526.133
Hooded Crane	-4809872.521	6736975.515
Hooded Crane	-4812141.212	6737077.94
Hooded Crane	-5019542.782	7275160.693
Hooded Crane	-5019805.496	7273033.452

Hooded Crane	-4810159.725	6736833.559
Hooded Crane	-4812152.344	6737081.534
Hooded Crane	-4813995.795	6734729.67
Hooded Crane	-4812143.438	6737074.346
Hooded Crane	-4814249.603	6734569.79
Hooded Crane	-5023064.93	7269799.47
Hooded Crane	-5021054.5	7270224.31
Hooded Crane	-5020477.865	7274652.872
Hooded Crane	-4812170.155	6737076.143
Hooded Crane	-4812150.117	6737058.174
Hooded Crane	-5095709.804	7225096.446
Hooded Crane	-5018409.549	7271583.569
Hooded Crane	-4814271.867	6734812.306
Hooded Crane	-5018487.473	7269468.842
Hooded Crane	-5102671.725	7212560.925
Hooded Crane	-4812150.117	6737077.94
Hooded Crane	-5016089.651	7268594.282
Hooded Crane	-4812521.925	6736008.827
Hooded Crane	-4812152.344	6737068.956
Hooded Crane	-5102665.045	7215843.724
Hooded Crane	-4813229.917	6734370.393
Hooded Crane	-4814225.113	6734501.527
Hooded Crane	-5018173.552	7271927.748
Hooded Crane	-4849186.112	6937444.871
Hooded Crane	-5015602.072	7273891.202
Hooded Crane	-4814614.731	6734747.634
Hooded Crane	-4812686.677	6736901.841
Hooded Crane	-4814165	6735051.237
Hooded Crane	-4812735.658	6735457.255
Hooded Crane	-4814282.999	6735626.136
Hooded Crane	-5023389.983	7269726.423
Hooded Crane	-5018627.735	7269370.809
Hooded Crane	-5093485.64	7223279.035
Hooded Crane	-5021893.849	7271985.433
Hooded Crane	-5014678.12	7280822.072
Hooded Crane	-4812163.476	6737090.519
Hooded Crane	-5023374.398	7269724.501
Hooded Crane	-5019865.608	7275066.436
Hooded Crane	-4813396.896	6734479.971
Hooded Crane	-4810691.832	6737464.294
Hooded Crane	-5018507.51	7269564.953
Hooded Crane	-5019805.496	7272929.605
Hooded Crane	-5104564.156	7213030.357
Hooded Crane	-5016452.553	7267325.864
Hooded Crane	-4812145.665	6737052.783
Hooded Crane	-5100307.299	7214318.577
Hooded Crane	-4811994.27	6735013.51
Hooded Crane	-5018483.02	7269451.542
Hooded Crane	-5018705.659	7268846.068
Hooded Crane	-5019438.141	7275037.582
Hooded Crane	-5018414.002	7275662.776
Hooded Crane	-4813040.673	6734911.111
Hooded Crane	-5020495.676	7270574.195
Hooded Crane	-5075193.621	7240106.788
Hooded Crane	-4812147.891	6737077.94
Hooded Crane	-5023000.365	7269991.702
Hooded Crane	-4812150.117	6737070.753
Hooded Crane	-4810391.27	6737541.567

Hooded Crane	-5018505.284	7269574.564
Hooded Crane	-4811989.817	6735394.375
Hooded Crane	-5017280.77	7269809.082
Hooded Crane	-4810665.115	6737245.058
Hooded Crane	-4814189.491	6734815.899
Hooded Crane	-4814220.66	6734530.269
Hooded Crane	-5097199.258	7217932.457
Hooded Crane	-4855595.888	6955860.65
Hooded Crane	-4811947.516	6735453.662
Hooded Crane	-5018647.773	7273810.423
Hooded Crane	-4813022.862	6734947.041
Hooded Crane	-5016412.478	7271943.13
Hooded Crane	-4812127.854	6737068.956
Hooded Crane	-4812127.854	6737085.128
Hooded Crane	-4810173.083	6736224.435
Hooded Crane	-5017035.867	7272748.838
Hooded Crane	-5018645.547	7269353.509
Hooded Crane	-5017817.329	7270033.994
Hooded Crane	-4810914.471	6736682.621
Hooded Crane	-5019309.011	7273108.453
Hooded Crane	-4811250.656	6735879.464
Hooded Crane	-4812152.344	6737072.55
Hooded Crane	-4963346.476	7249401.848
Hooded Crane	-5017766.123	7273481.547
Hooded Crane	-5019211.05	7274348.964
Hooded Crane	-5103991.974	7214990.445
Hooded Crane	-4810642.852	6737257.637
Hooded Crane	-4810760.85	6737502.032
Hooded Crane	-4809863.615	6737401.398
Hooded Crane	-4811028.017	6736817.387
Hooded Crane	-4812181.287	6737079.737
Hooded Crane	-5018491.926	7269589.942
Hooded Crane	-5016871.114	7270889.491
Hooded Crane	-4814280.773	6734724.281
Hooded Crane	-4964628.877	7252460.477
Hooded Crane	-5019841.118	7275035.658
Hooded Crane	-5014640.271	7280556.422
Hooded Crane	-5014361.973	7283823.778
Hooded Crane	-5104708.871	7215458.114
Hooded Crane	-4812410.605	6735451.866
Hooded Crane	-5100329.562	7214186.883
Hooded Crane	-4812138.986	6735694.408
Hooded Crane	-4812294.833	6736903.638
Hooded Crane	-5018514.19	7269570.72
Hooded Crane	-4812145.665	6737050.986
Hooded Crane	-4963335.344	7249328.993
Hooded Crane	-4810199.8	6737027.626
Hooded Crane	-4814318.621	6734767.395
Hooded Crane	-4812417.284	6735500.374
Hooded Crane	-4812154.57	6737074.346
Hooded Crane	-5018229.212	7269516.897
Hooded Crane	-5020640.392	7274895.237
Hooded Crane	-4813312.293	6735119.504
Hooded Crane	-5100739.218	7214251.775
Hooded Crane	-5016806.549	7273550.783
Hooded Crane	-4812751.243	6735017.103
Hooded Crane	-5020722.768	7273208.456
Hooded Crane	-4810823.189	6737207.322

Hooded Crane	-4809565.279	6737636.811
Hooded Crane	-4813292.255	6735121.3
Hooded Crane	-4812076.647	6735356.647
Hooded Crane	-4810133.008	6736912.623
Hooded Crane	-5018509.737	7269534.197
Hooded Crane	-4963252.968	7249353.917
Hooded Crane	-4963339.797	7249357.751
Hooded Crane	-4812392.794	6736175.922
Hooded Crane	-4810709.643	6737297.171
Hooded Crane	-4814100.435	6734833.864
Hooded Crane	-5103413.112	7214347.207
Hooded Crane	-5100670.2	7213549.436
Hooded Crane	-4810674.021	6737081.534
Hooded Crane	-5093476.735	7223129.992
Hooded Crane	-4813312.293	6735112.318
Hooded Crane	-4814209.528	6734503.324
Hooded Crane	-5099118.406	7210208.467
Hooded Crane	-5018489.699	7269470.764
Hooded Crane	-4812130.08	6737092.316
Hooded Crane	-5019186.559	7274270.104
Hooded Crane	-4813303.387	6735103.335
Hooded Crane	-4813314.519	6734476.378
Hooded Crane	-4813385.764	6734824.881
Hooded Crane	-4810852.132	6737225.292
Hooded Crane	-5097132.467	7217863.715
Hooded Crane	-5019233.313	7274398.974
Hooded Crane	-5021987.358	7273227.687
Hooded Crane	-4810930.056	6737056.377
Hooded Crane	-4812152.344	6737068.956
Hooded Crane	-4812145.665	6737097.707
Hooded Crane	-5018307.135	7272216.177
Hooded Crane	-5020328.697	7271939.285
Hooded Crane	-4814231.792	6734747.634
Hooded Crane	-4814227.339	6734539.251
Hooded Crane	-4814169.453	6734830.271
Hooded Crane	-5106449.908	7225488.268
Hooded Crane	-4813459.235	6734602.125
Hooded Crane	-5106928.582	7225018.085
Hooded Crane	-4812147.891	6737067.159
Hooded Crane	-5103019.041	7211589.711
Hooded Crane	-5104570.835	7213026.54
Hooded Crane	-5016632.89	7269355.432
Hooded Crane	-5100716.954	7213910.141
Hooded Crane	-4811958.648	6735487.797
Hooded Crane	-5099786.323	7210803.669
Hooded Crane	-4812161.249	6737068.956
Hooded Crane	-5020448.922	7274674.03
Hooded Crane	-5093625.903	7223410.883
Hooded Crane	-4814262.961	6734715.299
Hooded Crane	-4813595.044	6734616.496
Hooded Crane	-5093389.905	7222908.343
Hooded Crane	-5016049.576	7273246.919
Hooded Crane	-5018572.076	7276068.698
Hooded Crane	-5018997.316	7273952.748
Hooded Crane	-4810313.346	6737527.19
Hooded Crane	-5019705.308	7270066.674
Hooded Crane	-5098995.955	7212894.867
Hooded Crane	-4812824.714	6736862.309

Hooded Crane	-5017806.198	7270053.218
Hooded Crane	-4809897.011	6737489.452
Hooded Crane	-5023000.365	7270003.236
Hooded Crane	-5023799.639	7269359.276
Hooded Crane	-4812826.94	6735088.963
Hooded Crane	-4813572.781	6734623.682
Hooded Crane	-5095696.445	7225211.124
Hooded Crane	-4812731.205	6735094.353
Hooded Crane	-4810889.981	6737148.022
Hooded Crane	-5018044.421	7272041.195
Hooded Crane	-4812688.904	6735024.289
Hooded Crane	-5020170.624	7273370.002
Hooded Crane	-5014673.667	7280814.372
Hooded Crane	-5101166.685	7214505.626
Hooded Crane	-5023799.639	7269353.509
Hooded Crane	-4812539.736	6735989.063
Hooded Crane	-4812143.438	6737063.565
Hooded Crane	-4812352.719	6735647.696
Hooded Crane	-5020411.074	7272083.498
Hooded Crane	-4812604.301	6735182.382
Hooded Crane	-4811348.617	6735715.968
Hooded Crane	-5019211.05	7274414.361
Hooded Crane	-4812141.212	6737065.362
Hooded Crane	-4814018.059	6734726.077
Hooded Crane	-4810945.641	6736882.075
Hooded Crane	-4812733.432	6735410.544
Hooded Crane	-4812214.683	6737042.001
Hooded Crane	-4811114.846	6737094.113
Hooded Crane	-4812156.797	6735804.004
Hooded Crane	-4812161.249	6737052.783
Hooded Crane	-5014484.424	7283789.114
Hooded Crane	-4812134.533	6737083.331
Hooded Crane	-4812381.662	6736192.093
Hooded Crane	-5019422.557	7275031.811
Hooded Crane	-4812163.476	6737070.753
Hooded Crane	-5020457.828	7274658.642
Hooded Crane	-4812141.212	6737068.956
Hooded Crane	-5018503.058	7269549.575
Hooded Crane	-5015751.24	7273718.106
Hooded Crane	-5101807.885	7212526.577
Hooded Crane	-4810861.038	6737228.886
Hooded Crane	-4810633.946	6737254.043
Hooded Crane	-4813425.839	6734939.855
Hooded Crane	-5095631.88	7225656.471
Hooded Crane	-4849847.35	6937623.602
Hooded Crane	-5075035.548	7240371.058
Hooded Crane	-4812163.476	6737050.986
Hooded Crane	-4812815.808	6735417.73
Hooded Crane	-4812138.986	6737081.534
Hooded Crane	-4812976.108	6735211.127
Hooded Crane	-4812194.645	6736634.106
Hooded Crane	-5106636.925	7212223.18
Hooded Crane	-4814238.471	6734559.012
Hooded Crane	-5101870.224	7212667.785
Hooded Crane	-4818326.123	6742175.702
Hooded Crane	-5093452.244	7222994.326
Hooded Crane	-5021368.421	7270949.09
Hooded Crane	-4812715.62	6735035.068

Hooded Crane	-4871677.102	6903813.67
Hooded Crane	-4812156.797	6737068.956
Hooded Crane	-4812147.891	6737076.143
Hooded Crane	-5020408.847	7272054.655
Hooded Crane	-4810625.04	6737239.667
Hooded Crane	-4812713.394	6735376.409
Hooded Crane	-4812041.024	6735358.444
Hooded Crane	-4814238.471	6734542.844
Hooded Crane	-4812134.533	6737059.971
Hooded Crane	-5014671.441	7280808.596
Hooded Crane	-4962932.368	7248797.935
Hooded Crane	-4857094.249	6958987.998
Hooded Crane	-4812617.659	6734814.103
Hooded Crane	-5015826.937	7273677.717
Hooded Crane	-5019340.18	7275104.908
Hooded Crane	-4814238.471	6734544.641
Hooded Crane	-4813283.35	6735085.37
Hooded Crane	-5015430.64	7274933.708
Hooded Crane	-4857316.888	6956104.439
Hooded Crane	-5017031.414	7272754.607
Hooded Crane	-5017038.093	7272739.223
Hooded Crane	-5104279.178	7214978.992
Hooded Crane	-5018418.455	7273441.16
Hooded Crane	-5101932.563	7213001.732
Hooded Crane	-4812147.891	6737079.737
Hooded Crane	-4810255.46	6736475.985
Hooded Crane	-5019863.382	7272521.924
Hooded Crane	-4809785.691	6737412.18
Hooded Crane	-4849239.545	6937511.204
Hooded Crane	-5023398.889	7269661.066
Hooded Crane	-5019228.861	7273087.299
Hooded Crane	-4810914.471	6736998.875
Hooded Crane	-4814245.15	6734866.2
Hooded Crane	-5103649.11	7213410.121
Hooded Crane	-4812290.38	6736576.607
Hooded Crane	-4812147.891	6737061.768
Hooded Crane	-4813301.161	6735114.114
Hooded Crane	-4813853.306	6734727.874
Hooded Crane	-5023385.53	7269741.801
Hooded Crane	-4810745.266	6737446.323
Hooded Crane	-5017815.103	7270043.606
Hooded Crane	-4813303.387	6735108.725
Hooded Crane	-4812156.797	6737115.676
Hooded Crane	-4812141.212	6737077.94
Hooded Crane	-4812172.381	6737050.986
Hooded Crane	-4812101.137	6735719.561
Hooded Crane	-4810451.382	6737599.073
Hooded Crane	-4809854.71	6736977.312
Hooded Crane	-4814191.717	6734519.491
Hooded Crane	-5044600.799	7287127.19
Hooded Crane	-4810682.927	6737221.698
Hooded Crane	-4856412.973	6956326.072
Hooded Crane	-5023806.318	7269349.665
Hooded Crane	-5022114.262	7274046.992
Hooded Crane	-4812094.458	6735354.85
Hooded Crane	-5020264.132	7270781.827
Hooded Crane	-4812232.494	6736556.842
Hooded Crane	-4812141.212	6737079.737

Hooded Crane	-4812134.533	6737076.143
Hooded Crane	-5101360.381	7215101.156
Hooded Crane	-5019206.597	7270595.343
Hooded Crane	-4813312.293	6735133.876
Hooded Crane	-4809868.068	6736964.733
Hooded Crane	-4812163.476	6737059.971
Hooded Crane	-5019211.05	7274470.141
Hooded Crane	-5101035.328	7212772.738
Hooded Crane	-4811916.347	6735482.408
Hooded Crane	-4810317.799	6736603.56
Hooded Crane	-4812138.986	6737040.205
Hooded Crane	-5019832.212	7270579.963
Hooded Crane	-5106772.735	7212452.158
Hooded Crane	-4812159.023	6737085.128
Hooded Crane	-5020460.054	7274666.336
Hooded Crane	-5019500.48	7274366.275
Hooded Crane	-5017812.877	7270043.606
Hooded Crane	-4809580.864	6737622.434
Hooded Crane	-5086594.964	7221853.686
Hooded Crane	-4812018.76	6735349.461
Hooded Crane	-5023788.507	7269401.564
Hooded Crane	-4813603.95	6734672.185
Hooded Crane	-5020787.334	7273212.302
Hooded Crane	-4812152.344	6737081.534
Hooded Crane	-4812152.344	6737088.722
Hooded Crane	-5106492.209	7212144.948
Hooded Crane	-4814278.546	6734745.838
Hooded Crane	-5093525.715	7223204.513
Hooded Crane	-5074020.314	7240405.529
Hooded Crane	-4812415.058	6736423.877
Hooded Crane	-5015450.677	7274006.602
Hooded Crane	-4811909.667	6735435.696
Hooded Crane	-4810967.904	6737148.022
Hooded Crane	-4812408.379	6735268.616
Hooded Crane	-5097152.504	7217873.262
Hooded Crane	-5018336.078	7272089.267
Hooded Crane	-5015757.919	7273712.336
Hooded Crane	-5100970.763	7214085.728
Hooded Crane	-5020399.942	7272077.73
Hooded Crane	-5022931.347	7273085.376
Hooded Crane	-5101269.099	7213564.704
Hooded Crane	-4991991.208	7298172.535
Hooded Crane	-5018743.508	7272914.22
Hooded Crane	-5016839.944	7272754.607
Hooded Crane	-4809529.657	6737568.522
Hooded Crane	-4809632.071	6737631.42
Hooded Crane	-4810727.454	6736975.515
Hooded Crane	-5103085.833	7214665.956
Hooded Crane	-5093180.625	7222669.504
Hooded Crane	-4812159.023	6737094.113
Hooded Crane	-4811923.026	6735494.984
Hooded Crane	-4812147.891	6737074.346
Hooded Crane	-4812722.3	6734920.094
Hooded Crane	-5097876.081	7221112.455
Hooded Crane	-4811802.801	6735814.784
Hooded Crane	-5103506.621	7215183.236
Hooded Crane	-4814225.113	6734541.048
Hooded Crane	-5019304.558	7273041.144

Hooded Crane	-5018694.527	7272948.836
Hooded Crane	-4812116.722	6737083.331
Hooded Crane	-4814352.017	6734769.192
Hooded Crane	-4809514.072	6737854.26
Hooded Crane	-4812150.117	6737077.94
Hooded Crane	-4810235.422	6737557.74
Hooded Crane	-5020411.074	7272077.73
Hooded Crane	-4810642.852	6737252.246
Hooded Crane	-4814225.113	6734544.641
Hooded Crane	-5018937.204	7274348.964
Hooded Crane	-4812147.891	6737110.286
Hooded Crane	-4812150.117	6737056.377
Hooded Crane	-5094471.931	7220501.183
Hooded Crane	-5101191.175	7214757.575
Hooded Crane	-5100037.905	7209687.702
Hooded Crane	-5101028.649	7213709.747
Hooded Crane	-4812150.117	6737061.768
Hooded Crane	-4811983.138	6735410.544
Hooded Crane	-4810647.304	6737255.84
Hooded Crane	-4813960.172	6734799.731
Hooded Crane	-4849319.695	6937430.13
Hooded Crane	-4812150.117	6737056.377
Hooded Crane	-5019246.672	7273112.299
Hooded Crane	-4813136.408	6735130.283
Hooded Crane	-4871686.008	6903837.522
Hooded Crane	-5018398.417	7275776.278
Hooded Crane	-4812152.344	6737059.971
Hooded Crane	-5014373.104	7283794.891
Hooded Crane	-4812152.344	6737081.534
Hooded Crane	-4809910.369	6737239.667
Hooded Crane	-5020678.24	7274485.529
Hooded Crane	-5104566.382	7213030.357
Hooded Crane	-4809861.389	6737354.675
Hooded Crane	-4809988.293	6736765.277
Hooded Crane	-5073793.222	7240734.923
Hooded Crane	-5097299.446	7217936.276
Hooded Crane	-5109455.534	7208079.847
Hooded Crane	-5101890.262	7213024.632
Hooded Crane	-5101876.903	7213318.518
Hooded Crane	-5020310.886	7269297.766
Hooded Crane	-5102798.629	7214929.364
Hooded Crane	-4810941.188	6736501.14
Hooded Crane	-4849488.901	6933786.362
Hooded Crane	-4811923.026	6735428.51
Hooded Crane	-5022646.369	7271950.822
Hooded Crane	-4814229.566	6734533.862
Hooded Crane	-4812448.454	6735390.782
Hooded Crane	-5100006.736	7213784.178
Hooded Crane	-4812130.08	6737052.783
Hooded Crane	-4810718.549	6736984.5
Hooded Crane	-4812130.08	6736966.53
Hooded Crane	-4814245.15	6735511.153
Hooded Crane	-4812136.759	6737059.971
Hooded Crane	-5100046.811	7209758.28
Hooded Crane	-5103344.094	7215492.475
Hooded Crane	-5099813.04	7211723.269
Hooded Crane	-4856947.307	6956542.17
Hooded Crane	-4812481.85	6735514.746

Hooded Crane	-4812744.564	6735020.696
Hooded Crane	-4813595.044	6734783.563
Hooded Crane	-4810785.341	6737171.382
Hooded Crane	-4812096.684	6735706.985
Hooded Crane	-4812161.249	6737076.143
Hooded Crane	-4812165.702	6737054.58
Hooded Crane	-4810177.536	6736267.557
Hooded Crane	-4809469.544	6738425.765
Hooded Crane	-4812145.665	6737063.565
Hooded Crane	-5022866.782	7273025.759
Hooded Crane	-5086623.907	7221853.686
Hooded Crane	-5100979.668	7215047.709
Hooded Crane	-4812130.08	6735814.784
Hooded Crane	-5015991.69	7274006.602
Hooded Crane	-5023530.246	7273906.589
Hooded Crane	-4810916.698	6737031.22
Hooded Crane	-4812577.584	6735698.002
Hooded Crane	-5014417.632	7290672.941
Hooded Crane	-5100178.168	7208863.707
Hooded Crane	-4964312.729	7251467.01
Hooded Crane	-4812163.476	6737079.737
Hooded Crane	-5018186.91	7270810.666
Hooded Crane	-5100814.915	7213904.415
Hooded Crane	-4812150.117	6737079.737
Hooded Crane	-4810564.928	6737511.017
Hooded Crane	-4810881.075	6737049.189
Hooded Crane	-5101805.659	7212580.007
Hooded Crane	-4810694.059	6737227.089
Hooded Crane	-5075053.359	7240401.699
Hooded Crane	-4812330.455	6736555.045
Hooded Crane	-4856312.786	6955941.912
Hooded Crane	-4812141.212	6737065.362
Hooded Crane	-5020402.168	7272075.807
Hooded Crane	-4813868.89	6734639.849
Hooded Crane	-4810217.611	6736707.777
Hooded Crane	-4856370.672	6956954.066
Hooded Crane	-4812259.211	6736720.355
Hooded Crane	-5101583.02	7213131.498
Hooded Crane	-4813109.691	6735455.459
Hooded Crane	-4814222.886	6734532.066
Hooded Crane	-4812526.377	6734925.483
Hooded Crane	-4871672.649	6903833.852
Hooded Crane	-4812159.023	6737067.159
Hooded Crane	-5103092.512	7211721.361
Hooded Crane	-4814238.471	6734535.659
Hooded Crane	-5093398.811	7222763.128
Hooded Crane	-4812154.57	6737061.768
Hooded Crane	-4812780.186	6736878.481
Hooded Crane	-5016423.609	7267838.972
Hooded Crane	-5074069.295	7240426.594
Hooded Crane	-5074064.842	7240422.764
Hooded Crane	-4810262.139	6736483.172
Hooded Crane	-4810239.875	6736876.685
Hooded Crane	-4809407.205	6736698.793
Hooded Crane	-4812150.117	6737067.159
Hooded Crane	-5019306.784	7273054.606
Hooded Crane	-5100993.027	7212797.546
Hooded Crane	-4812138.986	6737065.362

Hooded Crane	-5016606.173	7272791.145
Hooded Crane	-4810954.546	6736918.013
Hooded Crane	-4809643.203	6737643.999
Hooded Crane	-4812163.476	6737074.346
Hooded Crane	-5023372.172	7269726.423
Hooded Crane	-4812130.08	6737090.519
Hooded Crane	-4814307.489	6734585.957
Hooded Crane	-5103413.112	7215618.465
Hooded Crane	-4813054.032	6734329.077
Hooded Crane	-4812167.929	6737103.098
Hooded Crane	-5097163.636	7217863.715
Hooded Crane	-4814087.077	6734823.085
Hooded Crane	-5093456.697	7222887.325
Hooded Crane	-4813229.917	6734373.986
Hooded Crane	-4813287.803	6735114.114
Hooded Crane	-4812130.08	6737094.113
Hooded Crane	-4814227.339	6735579.424
Hooded Crane	-4813766.476	6735063.812
Hooded Crane	-4810328.931	6737572.117
Hooded Crane	-5103021.268	7211902.623
Hooded Crane	-5100563.333	7213900.598
Hooded Crane	-4849471.09	6937656.769
Hooded Crane	-4812147.891	6737063.565
Hooded Crane	-4812150.117	6737070.753
Hooded Crane	-5101979.317	7212305.23
Hooded Crane	-5022403.693	7272712.301
Hooded Crane	-4810006.104	6737496.64
Hooded Crane	-5018445.171	7273471.931
Hooded Crane	-5099543.647	7209422.565
Hooded Crane	-4814360.923	6734744.042
Hooded Crane	-4813490.404	6734959.616
Hooded Crane	-4812152.344	6737077.94
Hooded Crane	-5100803.783	7213952.128
Hooded Crane	-5019845.571	7270028.227
Hooded Crane	-4810647.304	6737182.164
Hooded Crane	-5100574.465	7214024.653
Hooded Crane	-5099425.648	7211690.833
Hooded Crane	-4814165	6735396.172
Hooded Crane	-5099757.38	7209315.749
Hooded Crane	-5099919.907	7209331.008
Hooded Crane	-4812138.986	6737077.94
Hooded Crane	-4813557.196	6734587.754
Hooded Crane	-4811927.478	6735451.866
Hooded Crane	-4812746.79	6735405.154
Hooded Crane	-4810656.21	6737491.249
Hooded Crane	-4849911.915	6937542.528
Hooded Crane	-5017926.423	7275764.736
Hooded Crane	-4813394.669	6734772.785
Hooded Crane	-5097421.897	7222778.413
Hooded Crane	-5019952.437	7272021.967
Hooded Crane	-5099543.647	7211770.969
Hooded Crane	-4814229.566	6734544.641
Hooded Crane	-4814276.32	6734546.437
Hooded Crane	-4814229.566	6734713.502
Hooded Crane	-4812228.041	6735380.002
Hooded Crane	-5093416.622	7223179.672
Hooded Crane	-5101095.441	7214309.034
Hooded Crane	-4856205.919	6956510.771

Hooded Crane	-5015285.924	7282706.907
Hooded Crane	-5099686.136	7210071.119
Hooded Crane	-4812165.702	6737058.174
Hooded Crane	-4814205.075	6734848.235
Hooded Crane	-5101888.035	7212730.757
Hooded Crane	-4813247.728	6735157.231
Hooded Crane	-5102115.127	7215307.312
Hooded Crane	-4811179.412	6737200.134
Hooded Crane	-5013979.033	7282508.583
Hooded Crane	-5095614.069	7225616.331
Hooded Crane	-4812156.797	6737074.346
Hooded Crane	-5019660.78	7272539.231
Hooded Crane	-4855905.357	6956473.831
Hooded Crane	-5100636.804	7208100.825
Hooded Crane	-4810874.396	6737241.464
Hooded Crane	-5093323.114	7222870.128
Hooded Crane	-4812152.344	6737068.956
Hooded Crane	-4812143.438	6737070.753
Hooded Crane	-5099877.605	7209273.786
Hooded Crane	-4812399.473	6735556.068
Hooded Crane	-5015593.166	7273898.895
Hooded Crane	-4814109.341	6734772.785
Hooded Crane	-4814311.942	6734663.203
Hooded Crane	-5074069.295	7240418.934
Hooded Crane	-5101554.077	7213459.74
Hooded Crane	-4812535.283	6734907.518
Hooded Crane	-4812143.438	6737077.94
Hooded Crane	-4811108.167	6736686.215
Hooded Crane	-5018260.381	7270547.281
Hooded Crane	-5019447.047	7275052.97
Hooded Crane	-4810636.172	6737257.637
Hooded Crane	-4813265.539	6734458.415
Hooded Crane	-4855658.227	6955871.731
Hooded Crane	-5101983.77	7212986.466
Hooded Crane	-5021566.57	7272096.958
Hooded Crane	-5023389.983	7269659.143
Hooded Crane	-5023376.625	7269730.268
Hooded Crane	-4812163.476	6737124.661
Hooded Crane	-4856858.251	6953094.539
Hooded Crane	-4812557.547	6735534.509
Hooded Crane	-5019847.797	7270026.305
Hooded Crane	-4812152.344	6737090.519
Hooded Crane	-4812125.627	6737040.205
Hooded Crane	-4810694.059	6737268.419
Hooded Crane	-5105205.356	7212436.893
Hooded Crane	-5103566.733	7215337.854
Hooded Crane	-4856052.298	6956290.98
Hooded Crane	-4812147.891	6737061.768
Hooded Crane	-4812829.166	6735480.611
Hooded Crane	-4812152.344	6737085.128
Hooded Crane	-4812145.665	6737094.113
Hooded Crane	-5101890.262	7212406.362
Hooded Crane	-4813318.972	6735121.3
Hooded Crane	-4814320.848	6734670.388
Hooded Crane	-5093616.997	7223559.932
Hooded Crane	-4812145.665	6737067.159
Hooded Crane	-4810453.608	6737622.434
Hooded Crane	-4810224.29	6737568.522

Hooded Crane	-4810832.095	6737140.834
Hooded Crane	-4810346.742	6737379.834
Hooded Crane	-4812840.298	6735159.027
Hooded Crane	-4812132.306	6735751.901
Hooded Crane	-4812740.111	6735092.556
Hooded Crane	-4812154.57	6737068.956
Hooded Crane	-5019438.141	7275043.352
Hooded Crane	-4813103.012	6734853.625
Hooded Crane	-4855760.641	6955487.594
Hooded Crane	-5101113.252	7214330.029
Hooded Crane	-4810351.195	6737473.279
Hooded Crane	-5105240.978	7212984.557
Hooded Crane	-4812147.891	6737081.534
Hooded Crane	-4812196.872	6736912.623
Hooded Crane	-5020578.053	7271950.822
Hooded Crane	-5103640.204	7215467.659
Hooded Crane	-4814222.886	6734506.917
Hooded Crane	-4813454.782	6734569.79
Hooded Crane	-5101021.97	7212881.509
Hooded Crane	-4812130.08	6737095.91
Hooded Crane	-4812125.627	6737076.143
Hooded Crane	-4811157.148	6736781.449
Hooded Crane	-5019725.346	7270053.218
Hooded Crane	-5016054.029	7273745.032
Hooded Crane	-4813160.898	6734359.615
Hooded Crane	-4813374.632	6734679.37
Hooded Crane	-5099957.755	7212020.922
Hooded Crane	-5093149.455	7222726.825
Hooded Crane	-4813082.975	6734850.032
Hooded Crane	-5102003.808	7212471.24
Hooded Crane	-5097132.467	7217854.167
Hooded Crane	-5100316.204	7214232.689
Hooded Crane	-5016661.833	7272050.809
Hooded Crane	-5103615.714	7215087.794
Hooded Crane	-4856931.722	6956346.389
Hooded Crane	-4811934.158	6735500.374
Hooded Crane	-5105724.105	7213879.604
Hooded Crane	-4814256.282	6734659.61
Hooded Crane	-4814231.792	6734492.546
Hooded Crane	-4812156.797	6737058.174
Hooded Crane	-5102999.004	7215671.915
Hooded Crane	-4810863.264	6736657.465
Hooded Crane	-4814082.624	6735155.434
Hooded Crane	-5102527.009	7212574.282
Hooded Crane	-4810324.478	6736517.312
Hooded Crane	-5096420.022	7220359.834
Hooded Crane	-4810371.232	6737403.195
Hooded Crane	-4809856.936	6736977.312
Hooded Crane	-4810907.792	6737085.128
Hooded Crane	-5102072.826	7215280.588
Hooded Crane	-4811976.459	6735548.882
Hooded Crane	-4858766.267	6953048.384
Hooded Crane	-4812141.212	6736849.731
Hooded Crane	-4810239.875	6736436.455
Hooded Crane	-5100968.536	7212828.078
Hooded Crane	-4812034.345	6736803.012
Hooded Crane	-4810651.757	6737255.84
Hooded Crane	-5018371.701	7271598.951

Hooded Crane	-4809556.373	6737663.767
Hooded Crane	-4814225.113	6734751.227
Hooded Crane	-5099788.55	7211896.899
Hooded Crane	-4810291.082	6736547.858
Hooded Crane	-4812143.438	6737083.331
Hooded Crane	-4814245.15	6735518.34
Hooded Crane	-5020453.375	7274664.413
Hooded Crane	-4809890.332	6737372.645
Hooded Crane	-5101099.893	7213515.084
Hooded Crane	-4813419.16	6734699.131
Hooded Crane	-5101974.865	7215108.791
Hooded Crane	-5105561.579	7228023.161
Hooded Crane	-5021559.891	7274370.122
Hooded Crane	-4812147.891	6737072.55
Hooded Crane	-5017561.295	7273244.996
Hooded Crane	-4814236.245	6734555.419
Hooded Crane	-4812107.816	6735694.408
Hooded Crane	-5101928.11	7212728.848
Hooded Crane	-4810849.906	6737187.555
Hooded Crane	-5101489.512	7213349.052
Hooded Crane	-4812379.436	6736303.493
Hooded Crane	-5020620.354	7274800.983
Hooded Crane	-4810649.531	6737250.449
Hooded Crane	-5093345.378	7223152.921
Hooded Crane	-4814225.113	6734524.88
Hooded Crane	-4814136.057	6735277.599
Hooded Crane	-4812415.058	6736413.097
Hooded Crane	-5074069.295	7240417.019
Hooded Crane	-5104646.532	7213053.257
Hooded Crane	-5021593.287	7272746.915
Hooded Crane	-4810631.72	6737248.652
Hooded Crane	-4809649.882	6737437.338
Hooded Crane	-5101921.431	7212646.794
Hooded Crane	-5101131.063	7213415.846
Hooded Crane	-4812152.344	6737068.956
Hooded Crane	-4811054.734	6736610.747
Hooded Crane	-4813481.499	6734830.271
Hooded Crane	-5017104.885	7270653.018
Hooded Crane	-4812156.797	6737072.55
Hooded Crane	-4812147.891	6736903.638
Hooded Crane	-5101937.016	7213118.14
Hooded Crane	-4812147.891	6737074.346
Hooded Crane	-4855197.365	6955847.722
Hooded Crane	-4963344.25	7249352
Hooded Crane	-4813031.768	6734939.855
Hooded Crane	-4812517.472	6736001.64
Hooded Crane	-4812167.929	6735778.851
Hooded Crane	-5103544.469	7215402.756
Hooded Crane	-5099029.351	7213083.79
Hooded Crane	-4809556.373	6737566.725
Hooded Crane	-5103134.814	7214688.861
Hooded Crane	-5099565.911	7211076.485
Hooded Crane	-5022762.141	7272027.735
Hooded Crane	-4813305.614	6735114.114
Hooded Crane	-5093314.208	7222917.896
Hooded Crane	-5100271.676	7214272.77
Hooded Crane	-4963480.06	7249467.035
Hooded Crane	-4810658.436	6737245.058

Hooded Crane	-5104207.934	7211534.38
Hooded Crane	-5099521.383	7210969.647
Hooded Crane	-4813321.199	6735133.876
Hooded Crane	-4811918.573	6735460.848
Hooded Crane	-4813314.519	6735106.928
Hooded Crane	-4812132.306	6737065.362
Hooded Crane	-4963299.722	7249459.366
Hooded Crane	-5103324.057	7215475.295
Hooded Crane	-5100810.463	7213856.702
Hooded Crane	-5101079.856	7214255.593
Hooded Crane	-4814151.642	6735211.127
Hooded Crane	-4813828.815	6734496.138
Hooded Crane	-4855560.266	6955777.542
Hooded Crane	-4811243.977	6737054.58
Hooded Crane	-4813572.781	6734648.831
Hooded Crane	-5017803.971	7270047.451
Hooded Crane	-4812408.379	6735473.425
Hooded Crane	-4813287.803	6735114.114
Hooded Crane	-4810910.018	6737165.991
Hooded Crane	-5097132.467	7217865.624
Hooded Crane	-4812138.986	6737079.737
Hooded Crane	-5018997.316	7273821.963
Hooded Crane	-5100897.292	7214461.726
Hooded Crane	-5099608.212	7211047.868
Hooded Crane	-4812165.702	6737085.128
Hooded Crane	-4812152.344	6737054.58
Hooded Crane	-5024291.671	7272693.07
Hooded Crane	-4812141.212	6737065.362
Hooded Crane	-4810943.414	6736900.044
Hooded Crane	-4813969.078	6734815.899
Hooded Crane	-4812130.08	6737086.925
Hooded Crane	-5017806.198	7270060.907
Hooded Crane	-4810640.625	6737257.637
Hooded Crane	-4813775.382	6735062.016
Hooded Crane	-5018823.658	7273660.408
Hooded Crane	-4809543.015	6737584.696
Hooded Crane	-4814196.17	6734756.617
Hooded Crane	-4812190.192	6737110.286
Hooded Crane	-5099862.021	7211952.232
Hooded Crane	-4810676.247	6737245.058
Hooded Crane	-4812332.681	6735629.73
Hooded Crane	-5093432.207	7222984.773
Hooded Crane	-5019567.272	7274452.83
Hooded Crane	-4812147.891	6737074.346
Hooded Crane	-5015450.677	7273995.062
Hooded Crane	-5102947.797	7215376.032
Hooded Crane	-5100843.859	7214226.963
Hooded Crane	-5101908.073	7212662.06
Hooded Crane	-4809640.976	6738183.14
Hooded Crane	-4814305.263	6735604.577
Hooded Crane	-5097375.143	7217976.376
Hooded Crane	-5101925.884	7212207.916
Hooded Crane	-5101571.888	7213520.81
Hooded Crane	-5020170.624	7274700.959
Hooded Crane	-4814323.074	6734621.885
Hooded Crane	-4857141.003	6960107.715
Hooded Crane	-4811943.063	6735518.34
Hooded Crane	-5101222.345	7214475.087

Hooded Crane	-5020063.757	7272927.682
Hooded Crane	-4814245.15	6734526.677
Hooded Crane	-5101102.12	7214312.851
Hooded Crane	-5103163.757	7215481.021
Hooded Crane	-4812125.627	6736997.078
Hooded Crane	-5099548.1	7210313.387
Hooded Crane	-4812726.752	6736948.561
Hooded Crane	-4812167.929	6737068.956
Hooded Crane	-4810649.531	6737246.855
Hooded Crane	-4810642.852	6737241.464
Hooded Crane	-5102462.444	7212566.65
Hooded Crane	-4814314.168	6734693.742
Hooded Crane	-5017156.092	7268181.062
Hooded Crane	-5018525.321	7277167.296
Hooded Crane	-5075828.143	7240240.837
Hooded Crane	-4855647.095	6956381.481
Hooded Crane	-4812123.401	6737081.534
Hooded Crane	-4809881.426	6737101.301
Hooded Crane	-4813454.782	6734569.79
Hooded Crane	-4856343.955	6956728.721
Hooded Crane	-4812154.57	6737063.565
Hooded Crane	-4814227.339	6734533.862
Hooded Crane	-4813285.576	6735117.707
Hooded Crane	-4811940.837	6735401.561
Hooded Crane	-5103577.865	7215484.839
Hooded Crane	-4812134.533	6737077.94
Hooded Crane	-5102366.709	7212639.162
Hooded Crane	-4810941.188	6736959.343
Hooded Crane	-5100981.895	7213677.303
Hooded Crane	-5101600.831	7213312.793
Hooded Crane	-5075033.321	7240395.954
Hooded Crane	-5102092.863	7212822.353
Hooded Crane	-4812114.495	6735265.023
Hooded Crane	-4812757.922	6736885.669
Hooded Crane	-4812646.602	6735031.475
Hooded Crane	-4811840.649	6735746.511
Hooded Crane	-5101634.227	7212496.046
Hooded Crane	-4813056.258	6734354.226
Hooded Crane	-5027713.632	7270956.781
Hooded Crane	-4810629.493	6737232.48
Hooded Crane	-4814271.867	6734539.251
Hooded Crane	-4812018.76	6735358.444
Hooded Crane	-5099730.664	7209342.453
Hooded Crane	-5100754.803	7214106.722
Hooded Crane	-5101952.601	7212952.116
Hooded Crane	-4810869.943	6737042.001
Hooded Crane	-5103337.415	7215460.023
Hooded Crane	-5103366.358	7215412.301
Hooded Crane	-5109860.737	7207631.69
Hooded Crane	-4858641.59	6957783.463
Hooded Crane	-4812150.117	6737076.143
Hooded Crane	-5094630.005	7222379.086
Hooded Crane	-4809269.169	6737333.111
Hooded Crane	-4810636.172	6737279.201
Hooded Crane	-4813258.86	6734995.546
Hooded Crane	-5101088.761	7214236.507
Hooded Crane	-4812176.834	6737092.316
Hooded Crane	-5101093.214	7213335.693

Hooded Crane	-5099109.501	7212923.492
Hooded Crane	-4963337.571	7249359.669
Hooded Crane	-4812143.438	6737072.55
Hooded Crane	-4813575.007	6734612.903
Hooded Crane	-4813501.536	6734792.545
Hooded Crane	-4812996.146	6734921.89
Hooded Crane	-5019444.82	7275054.894
Hooded Crane	-5097123.561	7217892.358
Hooded Crane	-5075031.095	7240380.634
Hooded Crane	-5076520.55	7240459.15
Hooded Crane	-4812125.627	6737085.128
Hooded Crane	-4812152.344	6737086.925
Hooded Crane	-4812713.394	6737004.266
Hooded Crane	-5100296.167	7212270.884
Hooded Crane	-4812150.117	6737072.55
Hooded Crane	-5016977.981	7269787.936
Hooded Crane	-5100814.915	7213948.311
Hooded Crane	-4814567.977	6734765.599
Hooded Crane	-5099790.776	7212040.003
Hooded Crane	-4855388.834	6956459.055
Hooded Crane	-5101035.328	7213614.324
Hooded Crane	-5099890.964	7211477.141
Hooded Crane	-4812161.249	6737076.143
Hooded Crane	-4856301.654	6956272.51
Hooded Crane	-5099670.551	7210078.749
Hooded Crane	-4812187.966	6734673.981
Hooded Crane	-5103435.376	7215393.212
Hooded Crane	-5103108.097	7215074.433
Hooded Crane	-4810645.078	6737255.84
Hooded Crane	-4814171.679	6734762.006
Hooded Crane	-5093407.717	7222896.878
Hooded Crane	-5103337.415	7214757.575
Hooded Crane	-5022005.169	7271333.617
Hooded Crane	-5100120.282	7211866.37
Hooded Crane	-5101093.214	7214278.496
Hooded Crane	-4812682.225	6735085.37
Hooded Crane	-4812831.393	6735079.981
Hooded Crane	-4813494.857	6734656.017
Hooded Crane	-4813103.012	6735367.426
Hooded Crane	-5093289.718	7222736.378
Hooded Crane	-5100126.961	7211723.269
Hooded Crane	-4812163.476	6737086.925
Hooded Crane	-4813835.495	6734673.981
Hooded Crane	-5099510.251	7210914.32
Hooded Crane	-4964170.24	7251307.836
Hooded Crane	-4812374.983	6735730.341
Hooded Crane	-4810331.157	6737503.829
Hooded Crane	-4813312.293	6735110.521
Hooded Crane	-5086621.68	7221840.313
Hooded Crane	-4811410.956	6736377.161
Hooded Crane	-4812159.023	6737070.753
Hooded Crane	-5074071.521	7240409.359
Hooded Crane	-4856168.071	6956429.503
Hooded Crane	-5092561.688	7220942.44
Hooded Crane	-4812138.986	6737061.768
Hooded Crane	-4812049.93	6736785.043
Hooded Crane	-4812711.168	6736910.826
Hooded Crane	-4809859.162	6737379.834

Hooded Crane	-5016748.662	7275468.479
Hooded Crane	-5103689.185	7214780.48
Hooded Crane	-5100984.121	7212892.959
Hooded Crane	-5099565.911	7211118.458
Hooded Crane	-5104548.571	7213041.807
Hooded Crane	-5104553.024	7213037.99
Hooded Crane	-4809868.068	6736952.155
Hooded Crane	-4963515.682	7249127.685
Hooded Crane	-5101609.737	7213440.656
Hooded Crane	-5097535.443	7221104.814
Hooded Crane	-4812147.891	6737068.956
Hooded Crane	-4810943.414	6737047.392
Hooded Crane	-4814262.961	6734560.808
Hooded Crane	-4812555.32	6734984.767
Hooded Crane	-5103865.069	7214948.452
Hooded Crane	-5023806.318	7269372.731
Hooded Crane	-5099844.209	7209420.657
Hooded Crane	-4812150.117	6737083.331
Hooded Crane	-5100679.106	7214221.238
Hooded Crane	-4810674.021	6737372.645
Hooded Crane	-5099679.457	7210095.918
Hooded Crane	-4812147.891	6737092.316
Hooded Crane	-5101042.007	7212799.454
Hooded Crane	-4816137.582	6741524.821
Hooded Crane	-5101959.28	7212894.867
Hooded Crane	-5099485.761	7211938.876
Hooded Crane	-4811747.141	6735755.494
Hooded Crane	-5100336.242	7214291.857
Hooded Crane	-4812154.57	6737061.768
Hooded Crane	-4809672.146	6737430.15
Hooded Crane	-4812145.665	6737088.722
Hooded Crane	-4812141.212	6737052.783
Hooded Crane	-4812125.627	6735600.984
Hooded Crane	-5101367.06	7213505.542
Hooded Crane	-4810838.774	6737169.585
Hooded Crane	-4809870.294	6737480.467
Hooded Crane	-4856444.143	6956283.592
Hooded Crane	-5101066.497	7214299.491
Hooded Crane	-5100291.714	7214278.496
Hooded Crane	-5101006.385	7214184.974
Hooded Crane	-5099167.387	7213232.641
Hooded Crane	-4812147.891	6736898.247
Hooded Crane	-4810636.172	6737246.855
Hooded Crane	-4810631.72	6737250.449
Hooded Crane	-4814222.886	6734548.233
Hooded Crane	-4812020.987	6735365.63
Hooded Crane	-4813318.972	6735126.69
Hooded Crane	-5093238.511	7223011.523
Hooded Crane	-4812731.205	6736935.983
Hooded Crane	-4810867.717	6737173.179
Hooded Crane	-4812165.702	6737043.798
Hooded Crane	-5096898.696	7223099.419
Hooded Crane	-4809861.389	6736968.327
Hooded Crane	-4814240.698	6734562.604
Hooded Crane	-5100276.129	7214253.684
Hooded Crane	-4809990.519	6736497.547
Hooded Crane	-5101807.885	7212478.873
Hooded Crane	-5097116.882	7217871.353

Hooded Crane	-4813608.403	6734729.67
Hooded Crane	-5093220.7	7223365.023
Hooded Crane	-4810867.717	6737169.585
Hooded Crane	-5021724.644	7274375.892
Hooded Crane	-4812141.212	6737081.534
Hooded Crane	-4812159.023	6737068.956
Hooded Crane	-5100260.544	7214267.044
Hooded Crane	-4812199.098	6737113.879
Hooded Crane	-4812136.759	6737086.925
Hooded Crane	-4811028.017	6736887.466
Hooded Crane	-5100725.86	7213900.598
Hooded Crane	-4810845.453	6737435.541
Hooded Crane	-4814236.245	6734557.215
Hooded Crane	-5093267.454	7223068.846
Hooded Crane	-4812150.117	6737072.55
Hooded Crane	-4812234.72	6735830.954
Hooded Crane	-5103099.191	7215597.466
Hooded Crane	-4814180.585	6734855.421
Hooded Crane	-4813514.894	6734778.174
Hooded Crane	-5091775.773	7219605.375
Hooded Crane	-5020618.128	7275016.422
Hooded Crane	-5101391.55	7212898.684
Hooded Crane	-5099069.426	7210263.788
Hooded Crane	-5075946.141	7240556.819
Hooded Crane	-5075705.691	7240281.052
Hooded Crane	-4812156.797	6737045.595
Hooded Crane	-5021691.248	7272581.536
Hooded Crane	-4812130.08	6737083.331
Hooded Crane	-4812161.249	6737070.753
Hooded Crane	-4813082.975	6735663.866
Hooded Crane	-4812138.986	6737068.956
Hooded Crane	-4812366.077	6736177.719
Hooded Crane	-4810892.207	6737162.397
Hooded Crane	-5103704.769	7215601.284
Hooded Crane	-4812415.058	6735457.255
Hooded Crane	-4812831.393	6736794.027
Hooded Crane	-4809638.75	6737629.623
Hooded Crane	-5100959.631	7212833.803
Hooded Crane	-5104806.832	7215099.247
Hooded Crane	-5100229.375	7214303.308
Hooded Crane	-4812141.212	6737072.55
Hooded Crane	-4813479.272	6734779.97
Hooded Crane	-4812170.155	6735719.561
Hooded Crane	-5101836.828	7212518.945
Hooded Crane	-4812312.644	6735780.648
Hooded Crane	-5097121.335	7217844.62
Hooded Crane	-4809805.729	6737568.522
Hooded Crane	-5093274.133	7222873.949
Hooded Crane	-4849455.505	6937649.399
Hooded Crane	-4813343.462	6734481.767
Hooded Crane	-4812145.665	6737074.346
Hooded Crane	-4811947.516	6735444.679
Hooded Crane	-4812145.665	6735795.021
Hooded Crane	-4811117.073	6736919.81
Hooded Crane	-5099968.887	7211645.042
Hooded Crane	-4812159.023	6737076.143
Hooded Crane	-4812713.394	6735132.08
Hooded Crane	-5099191.877	7212383.464

Hooded Crane	-4814218.434	6734555.419
Hooded Crane	-5097123.561	7217856.077
Hooded Crane	-4812766.827	6735514.746
Hooded Crane	-5101010.838	7213812.806
Hooded Crane	-4810769.756	6737428.353
Hooded Crane	-4813321.199	6735117.707
Hooded Crane	-5101609.737	7213270.808
Hooded Crane	-4855268.609	6956514.465
Hooded Crane	-5101133.289	7207170.211
Hooded Crane	-5099572.59	7210378.247
Hooded Crane	-5100233.828	7208396.433
Hooded Crane	-5100367.411	7207959.7
Hooded Crane	-5019453.726	7275174.158
Hooded Crane	-4812159.023	6737088.722
Hooded Crane	-4812666.64	6736995.281
Hooded Crane	-4812176.834	6737047.392
Hooded Crane	-5021740.228	7272885.374
Hooded Crane	-5022940.252	7272183.488
Hooded Crane	-4813245.501	6734893.147
Hooded Crane	-4856662.329	6956416.574
Hooded Crane	-4811076.998	6736581.998
Hooded Crane	-4811114.846	6736608.95
Hooded Crane	-5103506.621	7215162.239
Hooded Crane	-4812014.308	6735347.664
Hooded Crane	-5101102.12	7213375.77
Hooded Crane	-4812161.249	6737072.55
Hooded Crane	-4812199.098	6735775.258
Hooded Crane	-4813942.361	6734799.731
Hooded Crane	-5099703.947	7210446.924
Hooded Crane	-5022457.126	7272718.07
Hooded Crane	-5101142.195	7213469.282
Hooded Crane	-4812708.941	6735017.103
Hooded Crane	-4813321.199	6735101.539
Hooded Crane	-4812143.438	6735775.258
Hooded Crane	-5075026.642	7240372.973
Hooded Crane	-4856838.214	6956575.417
Hooded Crane	-4812141.212	6737077.94
Hooded Crane	-4813263.312	6734463.804
Hooded Crane	-5102014.94	7213007.457
Hooded Crane	-5100986.347	7212812.812
Hooded Crane	-4812138.986	6737065.362
Hooded Crane	-4810834.321	6737176.773
Hooded Crane	-4812617.659	6735121.3
Hooded Crane	-5101979.317	7212797.546
Hooded Crane	-4813241.048	6735168.01
Hooded Crane	-4813359.047	6735099.742
Hooded Crane	-4964312.729	7251495.776
Hooded Crane	-4811825.065	6736980.906
Hooded Crane	-4855538.002	6956350.083
Hooded Crane	-4812138.986	6737067.159
Hooded Crane	-5101578.567	7213287.984
Hooded Crane	-5097132.467	7217856.077
Hooded Crane	-4812147.891	6737077.94
Hooded Crane	-4857412.622	6955792.317
Hooded Crane	-4812150.117	6737050.986
Hooded Crane	-4810912.245	6737077.94
Hooded Crane	-5103019.041	7215620.374
Hooded Crane	-5103388.622	7215578.377

Hooded Crane	-5093432.207	7223017.256
Hooded Crane	-5100302.846	7214280.405
Hooded Crane	-5101088.761	7214524.712
Hooded Crane	-5100269.45	7214291.857
Hooded Crane	-5103502.168	7215526.836
Hooded Crane	-4812134.533	6737043.798
Hooded Crane	-4810874.396	6737164.194
Hooded Crane	-4810573.833	6737514.611
Hooded Crane	-4813423.612	6734559.012
Hooded Crane	-4812094.458	6735306.343
Hooded Crane	-4812764.601	6735130.283
Hooded Crane	-4812152.344	6737081.534
Hooded Crane	-4812170.155	6737104.895
Hooded Crane	-4812147.891	6736901.841
Hooded Crane	-5003597.378	7286450.983
Hooded Crane	-5101794.527	7212394.913
Hooded Crane	-4812145.665	6737052.783
Hooded Crane	-4814307.489	6735660.272
Hooded Crane	-5100819.368	7214284.222
Hooded Crane	-5100964.084	7212801.362
Hooded Crane	-4964920.534	7250318.352
Hooded Crane	-5101919.205	7212976.924
Hooded Crane	-5016808.775	7271202.876
Hooded Crane	-5102119.58	7215072.524
Hooded Crane	-5101155.553	7215112.609
Hooded Crane	-4855607.02	6956002.86
Hooded Crane	-4812143.438	6737061.768
Hooded Crane	-5099178.519	7213261.267
Hooded Crane	-4811900.762	6735683.629
Hooded Crane	-5103355.226	7214790.024
Hooded Crane	-4814225.113	6734553.622
Hooded Crane	-4814198.396	6734533.862
Hooded Crane	-4814267.414	6734762.006
Hooded Crane	-5099541.42	7209401.583
Hooded Crane	-5101095.441	7214299.491
Hooded Crane	-5101104.346	7214505.626
Hooded Crane	-5097154.731	7217871.353
Hooded Crane	-5101819.017	7212375.831
Hooded Crane	-5102086.184	7215299.676
Hooded Crane	-5086594.964	7221861.328
Hooded Crane	-5102190.824	7212665.877
Hooded Crane	-5099534.741	7211248.192
Hooded Crane	-5103535.564	7215169.874
Hooded Crane	-4812152.344	6737061.768
Hooded Crane	-4814231.792	6734530.269
Hooded Crane	-5102874.326	7215471.477
Hooded Crane	-4812150.117	6737065.362
Hooded Crane	-4855898.677	6956237.418
Hooded Crane	-4812152.344	6737074.346
Hooded Crane	-4812118.948	6737077.94
Hooded Crane	-5097139.146	7217865.624
Hooded Crane	-5104397.177	7214770.936
Hooded Crane	-5101680.981	7213247.908
Hooded Crane	-5017138.281	7268182.984
Hooded Crane	-4813307.84	6735117.707
Hooded Crane	-5020010.323	7274789.442
Hooded Crane	-5097145.825	7217880.901
Hooded Crane	-4856335.05	6956470.137

Hooded Crane	-4812143.438	6737065.362
Hooded Crane	-5103488.81	7215177.509
Hooded Crane	-5102152.976	7215070.615
Hooded Crane	-5101598.605	7213448.289
Hooded Crane	-5101896.941	7212709.766
Hooded Crane	-5100242.733	7214297.582
Hooded Crane	-4855607.02	6955999.166
Hooded Crane	-5103054.664	7215612.738
Hooded Crane	-5103027.947	7215536.38
Hooded Crane	-5099552.552	7209624.755
Hooded Crane	-5019188.786	7270495.374
Hooded Crane	-5101865.771	7213060.89
Hooded Crane	-4814225.113	6734508.713
Hooded Crane	-5100269.45	7214253.684
Hooded Crane	-5103346.321	7215549.743
Hooded Crane	-5101396.003	7213141.04
Hooded Crane	-4814254.056	6734650.628
Hooded Crane	-5094471.931	7220518.375
Hooded Crane	-5093389.905	7223055.471
Hooded Crane	-5074964.303	7240353.823
Hooded Crane	-4812138.986	6735299.157
Hooded Crane	-4812619.886	6736188.5
Hooded Crane	-5092330.144	7220262.418
Hooded Crane	-4812154.57	6737092.316
Hooded Crane	-5101529.587	7213509.359
Hooded Crane	-4813748.665	6734460.211
Hooded Crane	-4812063.288	6735892.041
Hooded Crane	-4813105.239	6734803.324
Hooded Crane	-4809854.71	6737024.032
Hooded Crane	-5100705.822	7214156.345
Hooded Crane	-5097125.788	7217854.167
Hooded Crane	-4856317.239	6954398.074
Hooded Crane	-5097134.693	7217880.901
Hooded Crane	-4963324.212	7249359.669
Hooded Crane	-5100445.335	7209157.436
Hooded Crane	-5102090.637	7214698.405
Hooded Crane	-5100966.31	7212814.72
Hooded Crane	-4812161.249	6737049.189
Hooded Crane	-5103393.075	7215553.561
Hooded Crane	-5103399.754	7215557.378
Hooded Crane	-4812720.073	6736916.217
Hooded Crane	-5101689.887	7212454.066
Hooded Crane	-4814365.375	6734772.785
Hooded Crane	-4810847.679	6737185.758
Hooded Crane	-4812156.797	6737052.783
Hooded Crane	-5103304.019	7215545.925
Hooded Crane	-5101102.12	7214293.765
Hooded Crane	-5098172.191	7222547.221
Hooded Crane	-5101598.605	7213478.824
Hooded Crane	-4812136.759	6737068.956
Hooded Crane	-5102722.932	7212440.709
Hooded Crane	-5099746.248	7209399.675
Hooded Crane	-4812134.533	6737059.971
Hooded Crane	-5099118.406	7210223.728
Hooded Crane	-5103261.718	7215204.233
Hooded Crane	-4814222.886	6734541.048
Hooded Crane	-4812141.212	6737108.489
Hooded Crane	-5101856.866	7212452.158

Hooded Crane	-5101910.299	7213169.665
Hooded Crane	-4812143.438	6737081.534
Hooded Crane	-5097121.335	7217842.71
Hooded Crane	-5101896.941	7213028.448
Hooded Crane	-4813443.65	6734580.568
Hooded Crane	-4812259.211	6736689.809
Hooded Crane	-5100376.317	7212278.516
Hooded Crane	-4812156.797	6737077.94
Hooded Crane	-4810887.754	6737140.834
Hooded Crane	-5104573.061	7213015.09
Hooded Crane	-5103488.81	7215091.612
Hooded Crane	-5094458.573	7220522.195
Hooded Crane	-4856433.011	6956486.76
Hooded Crane	-4812159.023	6737074.346
Hooded Crane	-4812152.344	6737074.346
Hooded Crane	-5101678.755	7212717.399
Hooded Crane	-5100251.639	7214303.308
Hooded Crane	-5100984.121	7212818.536
Hooded Crane	-5100993.027	7212812.812
Hooded Crane	-5103032.4	7215626.1
Hooded Crane	-5101242.382	7214929.364
Hooded Crane	-4812679.998	6736973.718
Hooded Crane	-5101812.338	7212450.25
Hooded Crane	-5103344.094	7215534.471
Hooded Crane	-4812152.344	6737067.159
Hooded Crane	-5099158.481	7213057.073
Hooded Crane	-4812132.306	6737076.143
Hooded Crane	-5098837.881	7213480.733
Hooded Crane	-5099870.926	7209712.5
Hooded Crane	-4813949.04	6734781.767
Hooded Crane	-4810364.553	6736226.231
Hooded Crane	-4810769.756	6736959.343
Hooded Crane	-5100828.274	7213944.494
Hooded Crane	-4810878.849	6737165.991
Hooded Crane	-4811802.801	6737660.173
Hooded Crane	-5100289.487	7214312.851
Hooded Crane	-5100240.507	7214372.019
Hooded Crane	-5101004.159	7213751.734
Hooded Crane	-4856457.501	6956294.674
Hooded Crane	-4812136.759	6737067.159
Hooded Crane	-4964237.032	7251691.392
Hooded Crane	-5099532.515	7211177.601
Hooded Crane	-4812484.076	6735951.332
Hooded Crane	-5018574.302	7269293.922
Hooded Crane	-4812221.362	6735755.494
Hooded Crane	-4811036.923	6736682.621
Hooded Crane	-5102377.841	7212265.159
Hooded Crane	-5101073.177	7212891.051
Hooded Crane	-4811934.158	6735484.204
Hooded Crane	-4811063.639	6736608.95
Hooded Crane	-4812005.402	6735421.324
Hooded Crane	-5020642.618	7274797.136
Hooded Crane	-5102072.826	7212524.669
Hooded Crane	-5099995.604	7209941.404
Hooded Crane	-4812726.752	6736926.998
Hooded Crane	-5095896.82	7225801.739
Hooded Crane	-4813287.803	6735119.504
Hooded Crane	-4810858.811	6736680.824

Hooded Crane	-5100935.14	7214675.5
Hooded Crane	-4812145.665	6737090.519
Hooded Crane	-5102139.617	7215082.068
Hooded Crane	-5093418.848	7223345.914
Hooded Crane	-5101712.151	7212602.905
Hooded Crane	-4812150.117	6737079.737
Hooded Crane	-4812136.759	6737063.565
Hooded Crane	-5103749.297	7213400.579
Hooded Crane	-4813312.293	6735117.707
Hooded Crane	-5102079.505	7215299.676
Hooded Crane	-4813396.896	6734420.691
Hooded Crane	-5101921.431	7212316.679
Hooded Crane	-4856332.823	6956466.443
Hooded Crane	-5096268.627	7220115.343
Hooded Crane	-5100296.167	7214337.664
Hooded Crane	-5101620.869	7213278.442
Hooded Crane	-5103475.451	7213558.979
Hooded Crane	-5102317.729	7206089.084
Hooded Crane	-5015795.768	7272439.236
Hooded Crane	-5103353	7215536.38
Hooded Crane	-5101037.554	7214221.238
Hooded Crane	-4812757.922	6735168.01
Hooded Crane	-4812179.061	6737067.159
Hooded Crane	-4811310.769	6737176.773
Hooded Crane	-5100997.479	7212808.995
Hooded Crane	-4812147.891	6737076.143
Hooded Crane	-4810642.852	6737241.464
Hooded Crane	-5101485.059	7213266.992
Hooded Crane	-5106850.658	7212263.251
Hooded Crane	-5100273.903	7214265.136
Hooded Crane	-5101665.396	7213276.534
Hooded Crane	-4812150.117	6737086.925
Hooded Crane	-4814220.66	6734524.88
Hooded Crane	-5097125.788	7217865.624
Hooded Crane	-5099939.944	7211961.772
Hooded Crane	-4812415.058	6736432.861
Hooded Crane	-5103181.568	7215196.598
Hooded Crane	-5099799.682	7211954.14
Hooded Crane	-5101090.988	7208079.847
Hooded Crane	-5103662.468	7215215.686
Hooded Crane	-4809609.807	6737600.87
Hooded Crane	-5022679.765	7271818.147
Hooded Crane	-4813049.579	6734841.049
Hooded Crane	-5101142.195	7214100.996
Hooded Crane	-4812773.507	6736935.983
Hooded Crane	-5100311.751	7214293.765
Hooded Crane	-4812138.986	6737049.189
Hooded Crane	-4810139.687	6737595.478
Hooded Crane	-4816449.276	6737164.194
Hooded Crane	-5100247.186	7214244.141
Hooded Crane	-4812132.306	6737065.362
Hooded Crane	-5100630.125	7214083.819
Hooded Crane	-4812152.344	6737081.534
Hooded Crane	-4813036.221	6734927.279
Hooded Crane	-4814202.849	6734735.06
Hooded Crane	-4856277.164	6956289.133
Hooded Crane	-4812141.212	6737085.128
Hooded Crane	-5101583.02	7213448.289

Hooded Crane	-5100113.603	7211578.263
Hooded Crane	-4812715.62	6736934.186
Hooded Crane	-4813321.199	6735117.707
Hooded Crane	-5101709.924	7212709.766
Hooded Crane	-4810609.456	6737255.84
Hooded Crane	-5100347.374	7207919.652
Hooded Crane	-5093365.415	7221303.487
Hooded Crane	-5015844.748	7271199.03
Hooded Crane	-5099619.344	7211139.444
Hooded Crane	-5103515.526	7213923.5
Hooded Crane	-5100060.169	7211767.153
Hooded Crane	-4812130.08	6736878.481
Hooded Crane	-5016630.664	7272070.038
Hooded Crane	-4818660.081	6741708.213
Hooded Crane	-4814225.113	6734532.066
Hooded Crane	-4813303.387	6735121.3
Hooded Crane	-4964800.309	7252849.843
Hooded Crane	-5099879.832	7211784.325
Hooded Crane	-4812706.715	6735354.85
Hooded Crane	-5101683.207	7212866.243
Hooded Crane	-5093133.871	7222701.986
Hooded Crane	-4812159.023	6737083.331
Hooded Crane	-4855595.888	6956366.706
Hooded Crane	-5100970.763	7212828.078
Hooded Crane	-4812147.891	6737050.986
Hooded Crane	-5100972.989	7212841.436
Hooded Crane	-4812152.344	6737079.737
Hooded Crane	-4963355.382	7249305.986
Hooded Crane	-4814251.829	6734776.377
Hooded Crane	-5097123.561	7217863.715
Hooded Crane	-5101678.755	7213278.442
Hooded Crane	-5101195.628	7214944.634
Hooded Crane	-5101549.624	7213509.359
Hooded Crane	-4812829.166	6735076.388
Hooded Crane	-4810219.838	6737564.928
Hooded Crane	-5093815.146	7223496.873
Hooded Crane	-5099875.379	7210347.725
Hooded Crane	-5101193.402	7214919.82
Hooded Crane	-5099764.059	7209407.305
Hooded Crane	-5101538.492	7213513.176
Hooded Crane	-5102431.274	7212616.263
Hooded Crane	-4855529.097	6956344.542
Hooded Crane	-4814269.641	6734727.874
Hooded Crane	-4814643.674	6734690.149
Hooded Crane	-4812136.759	6736921.607
Hooded Crane	-4856323.918	6956483.066
Hooded Crane	-5101075.403	7214301.4
Hooded Crane	-4812152.344	6737067.159
Hooded Crane	-4812165.702	6737059.971
Hooded Crane	-5101418.267	7212944.483
Hooded Crane	-5093447.792	7223173.94
Hooded Crane	-4814238.471	6734850.032
Hooded Crane	-5020829.635	7272610.381
Hooded Crane	-5097303.899	7217970.648
Hooded Crane	-5093563.564	7223515.981
Hooded Crane	-5101525.134	7213337.602
Hooded Crane	-5101099.893	7214288.039
Hooded Crane	-5101493.964	7215072.524

Hooded Crane	-4812535.283	6736008.827
Hooded Crane	-5100305.072	7214297.582
Hooded Crane	-5101567.435	7213125.773
Hooded Crane	-5101059.818	7214650.687
Hooded Crane	-5100932.914	7213734.558
Hooded Crane	-5100716.954	7214217.42
Hooded Crane	-4812132.306	6737081.534
Hooded Crane	-4811965.327	6735798.614
Hooded Crane	-4963355.382	7249380.758
Hooded Crane	-5100298.393	7214280.405
Hooded Crane	-5101551.85	7212732.665
Hooded Crane	-4812212.456	6735812.987
Hooded Crane	-5099668.325	7210109.271
Hooded Crane	-5102139.617	7212768.922
Hooded Crane	-5101903.62	7212934.941
Hooded Crane	-5014373.104	7283819.926
Hooded Crane	-5100405.26	7208127.525
Hooded Crane	-4814231.792	6734521.288
Hooded Crane	-4812147.891	6737047.392
Hooded Crane	-5099327.687	7212272.792
Hooded Crane	-4858456.799	6956549.558
Hooded Crane	-5100895.065	7213839.525
Hooded Crane	-4816393.616	6734936.262
Hooded Crane	-5100979.668	7212906.317
Hooded Crane	-5100260.544	7214284.222
Hooded Crane	-5097123.561	7217867.534
Hooded Crane	-4855620.379	6956001.013
Hooded Crane	-4875504.266	6968580.935
Hooded Crane	-5099804.134	7209434.009
Hooded Crane	-5099338.819	7210124.532
Hooded Crane	-5101663.17	7213246
Hooded Crane	-4810856.585	6737169.585
Hooded Crane	-5099536.968	7211204.311
Hooded Crane	-5101113.252	7214165.888
Hooded Crane	-4812292.606	6735768.071
Hooded Crane	-5100269.45	7214301.4
Hooded Crane	-5103727.033	7215398.938
Hooded Crane	-4812548.641	6736042.964
Hooded Crane	-5017712.689	7272629.611
Hooded Crane	-5100964.084	7212763.197
Hooded Crane	-5101053.139	7212797.546
Hooded Crane	-5017131.601	7275420.387
Hooded Crane	-5100986.347	7212797.546
Hooded Crane	-5102295.465	7215269.134
Hooded Crane	-4812132.306	6737063.565
Hooded Crane	-4810874.396	6736414.893
Hooded Crane	-5018019.931	7271525.887
Hooded Crane	-4812686.677	6735176.993
Hooded Crane	-5096972.167	7223061.203
Hooded Crane	-5100247.186	7208302.981
Hooded Crane	-5103350.773	7214774.754
Hooded Crane	-4856348.408	6956723.18
Hooded Crane	-5101088.761	7214289.948
Hooded Crane	-5097130.24	7217859.896
Hooded Crane	-5100262.771	7214282.313
Hooded Crane	-4812401.7	6737088.722
Hooded Crane	-4810482.552	6737588.29
Hooded Crane	-5099815.266	7211793.865

Hooded Crane	-5100285.035	7214333.846
Hooded Crane	-4964539.821	7252600.493
Hooded Crane	-5100244.96	7214299.491
Hooded Crane	-4812141.212	6737033.017
Hooded Crane	-4812161.249	6735728.545
Hooded Crane	-5100964.084	7212816.628
Hooded Crane	-5101077.629	7214284.222
Hooded Crane	-4812165.702	6737083.331
Hooded Crane	-4856337.276	6956471.984
Hooded Crane	-5100739.218	7213976.94
Hooded Crane	-5103548.922	7214387.288
Hooded Crane	-5100378.543	7208718.753
Hooded Crane	-5100950.725	7213740.283
Hooded Crane	-5099695.041	7210158.869
Hooded Crane	-5092559.462	7220955.812
Hooded Crane	-5104288.084	7215070.615
Hooded Crane	-5101721.056	7213165.848
Hooded Crane	-5102081.731	7215286.314
Hooded Crane	-5093187.304	7223330.627
Hooded Crane	-4810858.811	6737151.616
Hooded Crane	-4875047.856	6968752.971
Hooded Crane	-5100986.347	7212786.096
Hooded Crane	-5102010.487	7212383.464
Hooded Crane	-5101921.431	7212923.492
Hooded Crane	-4811931.931	6735462.645
Hooded Crane	-4814140.51	6734823.085
Hooded Crane	-5100454.24	7214121.991
Hooded Crane	-5100285.035	7214274.679
Hooded Crane	-5075011.057	7240367.228
Hooded Crane	-4855546.908	6956049.032
Hooded Crane	-4812130.08	6737076.143
Hooded Crane	-5101070.95	7214265.136
Hooded Crane	-4811940.837	6735514.746
Hooded Crane	-4812374.983	6736238.809
Hooded Crane	-5101872.451	7212917.767
Hooded Crane	-5093425.528	7223210.245
Hooded Crane	-4856179.202	6956494.148
Hooded Crane	-5096268.627	7220082.872
Hooded Crane	-5100846.085	7214072.368
Hooded Crane	-4814641.448	6734731.467
Hooded Crane	-5103680.279	7215471.477
Hooded Crane	-5099648.287	7209561.808
Hooded Crane	-4814104.888	6734819.492
Hooded Crane	-5101001.932	7214797.659
Hooded Crane	-4812147.891	6737077.94
Hooded Crane	-5102925.533	7215666.189
Hooded Crane	-4856339.503	6956708.404
Hooded Crane	-4812123.401	6737079.737
Hooded Crane	-4812154.57	6737077.94
Hooded Crane	-4812136.759	6737077.94
Hooded Crane	-4814311.942	6734781.767
Hooded Crane	-5101770.037	7212591.456
Hooded Crane	-5100970.763	7212837.619
Hooded Crane	-5097125.788	7217863.715
Hooded Crane	-4812156.797	6737076.143
Hooded Crane	-5103531.111	7213505.542
Hooded Crane	-4855239.666	6955844.029
Hooded Crane	-4812161.249	6737059.971

Hooded Crane	-4812156.797	6737054.58
Hooded Crane	-5016632.89	7271973.896
Hooded Crane	-5093385.453	7223240.818
Hooded Crane	-4812130.08	6737058.174
Hooded Crane	-4812143.438	6737061.768
Hooded Crane	-5097128.014	7217856.077
Hooded Crane	-5100752.577	7214093.362
Hooded Crane	-4873774.361	6966735.024
Hooded Crane	-4812234.72	6735868.684
Hooded Crane	-4810233.196	6737577.508
Hooded Crane	-5100302.846	7214272.77
Hooded Crane	-5097145.825	7217808.34
Hooded Crane	-5099692.815	7209117.381
Hooded Crane	-4855533.549	6956346.389
Hooded Crane	-4855604.794	6955993.625
Hooded Crane	-5101028.649	7214165.888
Hooded Crane	-5099572.59	7211045.96
Hooded Crane	-5101090.988	7214205.969
Hooded Crane	-4812145.665	6737065.362
Hooded Crane	-5097136.919	7217865.624
Hooded Crane	-5101269.099	7214568.612
Hooded Crane	-5099601.533	7211648.858
Hooded Crane	-5097119.108	7217848.439
Hooded Crane	-5100289.487	7214267.044
Hooded Crane	-5016708.587	7272006.584
Hooded Crane	-4814296.357	6734609.311
Hooded Crane	-5101665.396	7213307.068
Hooded Crane	-5099866.473	7210349.632
Hooded Crane	-5097266.05	7217951.553
Hooded Crane	-5101896.941	7213020.815
Hooded Crane	-5100986.347	7212778.463
Hooded Crane	-5101925.884	7212656.336
Hooded Crane	-5103426.471	7215108.791
Hooded Crane	-5104089.935	7215502.02
Hooded Crane	-5099772.965	7209334.823
Hooded Crane	-4812138.986	6737052.783
Hooded Crane	-4810642.852	6737266.622
Hooded Crane	-4856337.276	6956473.831
Hooded Crane	-4856328.371	6956479.372
Hooded Crane	-5093349.83	7221309.218
Hooded Crane	-5101079.856	7214276.588
Hooded Crane	-5102956.702	7215649.008
Hooded Crane	-5100636.804	7214566.704
Hooded Crane	-4813274.444	6734483.564
Hooded Crane	-5094500.874	7220522.195
Hooded Crane	-5101894.715	7213058.982
Hooded Crane	-5101574.114	7213499.817
Hooded Crane	-5101447.21	7212996.007
Hooded Crane	-4827585.678	7132479.542
Hooded Crane	-5100975.216	7212799.454
Hooded Crane	-4812145.665	6737077.94
Hooded Crane	-5103397.528	7214809.111
Hooded Crane	-5100278.355	7214274.679
Hooded Crane	-5100808.236	7214870.192
Hooded Crane	-4812292.606	6735714.171
Hooded Crane	-4810215.385	6737584.696
Hooded Crane	-5101834.602	7212389.189
Hooded Crane	-4818668.987	6741672.254

Hooded Crane	-4812170.155	6737113.879
Hooded Crane	-4812415.058	6737288.186
Hooded Crane	-4812121.174	6737081.534
Hooded Crane	-4812163.476	6735750.104
Hooded Crane	-5091782.452	7219599.645
Hooded Crane	-4814122.699	6734907.518
Hooded Crane	-5093407.717	7223066.936
Hooded Crane	-4812159.023	6737088.722
Hooded Crane	-5100857.217	7213925.409
Hooded Crane	-4812098.911	6737052.783
Hooded Crane	-5097145.825	7217812.159
Hooded Crane	-4855549.134	6956050.879
Hooded Crane	-5101585.246	7214811.02
Hooded Crane	-4812145.665	6737079.737
Hooded Crane	-5099764.059	7209163.158
Hooded Crane	-4811123.752	6736689.809
Hooded Crane	-5100736.992	7214095.271
Hooded Crane	-5101039.781	7212784.188
Hooded Crane	-4812125.627	6737052.783
Hooded Crane	-5101465.021	7213345.235
Hooded Crane	-4856101.279	6956363.012
Hooded Crane	-4812141.212	6737079.737
Hooded Crane	-5099708.4	7209687.702
Hooded Crane	-4812637.697	6736930.592
Hooded Crane	-5100282.808	7214270.862
Hooded Crane	-4809966.029	6737505.626
Hooded Crane	-5103702.543	7215586.013
Hooded Crane	-5100993.027	7214184.974
Hooded Crane	-5097357.332	7217869.443
Hooded Crane	-4812771.28	6736851.528
Hooded Crane	-5103257.265	7215385.576
Hooded Crane	-4856343.955	6956710.251
Hooded Crane	-5099554.779	7211236.745
Hooded Crane	-5100639.031	7214038.013
Hooded Crane	-5023456.775	7269707.2
Hooded Crane	-5100334.015	7212265.159
Hooded Crane	-5101082.082	7214280.405
Hooded Crane	-4812092.231	6736988.093
Hooded Crane	-5103074.701	7211547.735
Hooded Crane	-5103317.378	7215507.746
Hooded Crane	-5101166.685	7213591.422
Hooded Crane	-5100111.376	7211721.361
Hooded Crane	-5099156.255	7210177.945
Hooded Crane	-4814242.924	6734548.233
Hooded Crane	-5099467.95	7212076.257
Hooded Crane	-5101896.941	7213030.357
Hooded Crane	-5102660.593	7212412.087
Hooded Crane	-5091789.131	7219605.375
Hooded Crane	-5093418.848	7223345.914
Hooded Crane	-4812141.212	6737076.143
Hooded Crane	-5100142.546	7211545.828
Hooded Crane	-5086599.416	7221863.238
Hooded Crane	-5101536.266	7213545.619
Hooded Crane	-5100968.536	7212826.17
Hooded Crane	-5100981.895	7212826.17
Hooded Crane	-5097132.467	7217863.715
Hooded Crane	-4812996.146	6734923.686
Hooded Crane	-4812143.438	6737074.346

Hooded Crane	-5103355.226	7215553.561
Hooded Crane	-4812156.797	6737042.001
Hooded Crane	-5097130.24	7217861.805
Hooded Crane	-4812143.438	6737054.58
Hooded Crane	-5102798.629	7215475.295
Hooded Crane	-5093140.55	7222875.86
Hooded Crane	-4814340.885	6734641.646
Hooded Crane	-4856328.371	6956488.607
Hooded Crane	-4965437.056	7252556.378
Hooded Crane	-4812147.891	6737085.128
Hooded Crane	-5100260.544	7214270.862
Hooded Crane	-4810542.664	6737530.784
Hooded Crane	-5100280.582	7214270.862
Hooded Crane	-5102763.007	7215853.269
Hooded Crane	-5102077.278	7215297.767
Hooded Crane	-4812114.495	6737031.22
Hooded Crane	-4812172.381	6737065.362
Hooded Crane	-5020698.278	7273875.816
Hooded Crane	-5100365.185	7212051.452
Hooded Crane	-5101663.17	7213286.075
Hooded Crane	-5093830.731	7223491.14
Hooded Crane	-5100260.544	7214293.765
Hooded Crane	-5101204.534	7213686.846
Hooded Crane	-5099868.7	7210342.002
Hooded Crane	-5018042.195	7272112.341
Hooded Crane	-5102139.617	7215085.885
Hooded Crane	-5097156.957	7217815.978
Hooded Crane	-5097132.467	7217865.624
Hooded Crane	-5100282.808	7214282.313
Hooded Crane	-5101449.437	7213446.381
Hooded Crane	-5100325.11	7212247.986
Hooded Crane	-4810255.46	6737575.711
Hooded Crane	-5097121.335	7217861.805
Hooded Crane	-5099154.029	7213301.343
Hooded Crane	-5100285.035	7214280.405
Hooded Crane	-4812789.091	6735493.187
Hooded Crane	-4813586.139	6734612.903
Hooded Crane	-4964544.274	7252592.821
Hooded Crane	-4812161.249	6737070.753
Hooded Crane	-5100251.639	7214276.588
Hooded Crane	-5100995.253	7212829.986
Hooded Crane	-5101567.435	7213282.259
Hooded Crane	-5099777.418	7211837.75
Hooded Crane	-4812595.395	6736914.42
Hooded Crane	-5101529.587	7212747.931
Hooded Crane	-5100837.179	7214068.55
Hooded Crane	-5101166.685	7215152.694
Hooded Crane	-4856326.144	6956704.709
Hooded Crane	-4812152.344	6737090.519
Hooded Crane	-5100129.187	7209691.517
Hooded Crane	-5098980.37	7213081.881
Hooded Crane	-5097125.788	7217861.805
Hooded Crane	-5100966.31	7214627.782
Hooded Crane	-5099151.802	7213333.785
Hooded Crane	-5100269.45	7214309.034
Hooded Crane	-5100968.536	7214393.014
Hooded Crane	-5099272.027	7212398.729
Hooded Crane	-5101126.61	7215076.341

Hooded Crane	-5097116.882	7217856.077
Hooded Crane	-4812138.986	6737072.55
Hooded Crane	-5101160.006	7214807.203
Hooded Crane	-4966080.483	7251986.742
Hooded Crane	-4812147.891	6737085.128
Hooded Crane	-4812688.904	6736966.53
Hooded Crane	-5101042.007	7214606.786
Hooded Crane	-5093527.942	7223454.833
Hooded Crane	-5101756.678	7212646.794
Hooded Crane	-5101195.628	7214933.181
Hooded Crane	-4813040.673	6734914.704
Hooded Crane	-5100313.978	7212255.619
Hooded Crane	-4814165	6735345.868
Hooded Crane	-5101393.777	7212999.824
Hooded Crane	-5096190.704	7221641.626
Hooded Crane	-4812130.08	6737067.159
Hooded Crane	-4810627.267	6737230.683
Hooded Crane	-5103241.68	7215545.925
Hooded Crane	-4812147.891	6737088.722
Hooded Crane	-5099138.444	7210187.483
Hooded Crane	-5097123.561	7217857.986
Hooded Crane	-4964562.085	7252673.379
Hooded Crane	-4812826.94	6735069.202
Hooded Crane	-5100289.487	7214282.313
Hooded Crane	-5106100.365	7214066.642
Hooded Crane	-5100305.072	7214289.948
Hooded Crane	-5101070.95	7212807.087
Hooded Crane	-4812121.174	6735827.361
Hooded Crane	-4812651.055	6734943.448
Hooded Crane	-4855246.345	6955238.283
Hooded Crane	-5101073.177	7214272.77
Hooded Crane	-5097134.693	7217854.167
Hooded Crane	-4964542.048	7252596.657
Hooded Crane	-4812145.665	6737074.346
Hooded Crane	-4812150.117	6737065.362
Hooded Crane	-5101215.666	7214524.712
Hooded Crane	-4855322.042	6956431.35
Hooded Crane	-4875047.856	6968754.821
Hooded Crane	-4875497.587	6968579.086
Hooded Crane	-5100988.574	7212801.362
Hooded Crane	-5101102.12	7214494.174
Hooded Crane	-4812136.759	6737070.753
Hooded Crane	-5100293.94	7208329.682
Hooded Crane	-5100267.224	7214270.862
Hooded Crane	-4812154.57	6737038.408
Hooded Crane	-4812014.308	6735353.054
Hooded Crane	-4812392.794	6736174.126
Hooded Crane	-4813312.293	6735128.486
Hooded Crane	-4812103.363	6737072.55
Hooded Crane	-4856339.503	6956708.404
Hooded Crane	-5100264.997	7214293.765
Hooded Crane	-5099534.741	7210906.689
Hooded Crane	-5097134.693	7217863.715
Hooded Crane	-5103704.769	7211469.51
Hooded Crane	-5103174.889	7215179.418
Hooded Crane	-5101678.755	7213301.343
Hooded Crane	-5098978.144	7212919.675
Hooded Crane	-4812161.249	6737090.519

Hooded Crane	-5099681.683	7210055.858
Hooded Crane	-4812076.647	6737077.94
Hooded Crane	-4812172.381	6737085.128
Hooded Crane	-5097134.693	7217831.254
Hooded Crane	-4812535.283	6735999.843
Hooded Crane	-4812145.665	6737056.377
Hooded Crane	-5097141.372	7217815.978
Hooded Crane	-4812815.808	6735035.068
Hooded Crane	-5097128.014	7217842.71
Hooded Crane	-5100924.009	7214667.865
Hooded Crane	-5101754.452	7212751.747
Hooded Crane	-5103317.378	7211370.298
Hooded Crane	-5100264.997	7214291.857
Hooded Crane	-5100305.072	7214341.481
Hooded Crane	-4814325.3	6734702.724
Hooded Crane	-4810885.528	6736682.621
Hooded Crane	-4855529.097	6956348.236
Hooded Crane	-5100750.35	7214087.636
Hooded Crane	-5100977.442	7212816.628
Hooded Crane	-5103346.321	7215549.743
Hooded Crane	-5099873.153	7210345.817
Hooded Crane	-4810992.395	6736997.078
Hooded Crane	-5101099.893	7213589.514
Hooded Crane	-5101471.7	7213328.06
Hooded Crane	-5101689.887	7213352.869
Hooded Crane	-5101010.838	7213417.755
Hooded Crane	-4810190.894	6737537.973
Hooded Crane	-5099726.211	7211950.324
Hooded Crane	-5097159.183	7217857.986
Hooded Crane	-5101531.813	7213513.176
Hooded Crane	-5099917.68	7208829.375
Hooded Crane	-5097125.788	7217850.348
Hooded Crane	-5101108.799	7214267.044
Hooded Crane	-5100267.224	7214278.496
Hooded Crane	-5101050.913	7212789.913
Hooded Crane	-5093812.919	7223491.14
Hooded Crane	-5101763.358	7215349.307
Hooded Crane	-5100273.903	7214244.141
Hooded Crane	-5100260.544	7214291.857
Hooded Crane	-4812152.344	6737072.55
Hooded Crane	-4812121.174	6737049.189
Hooded Crane	-4812143.438	6737056.377
Hooded Crane	-5102072.826	7215292.041
Hooded Crane	-4814287.452	6734645.239
Hooded Crane	-4811954.195	6737554.146
Hooded Crane	-4812165.702	6737079.737
Hooded Crane	-5100262.771	7214301.4
Hooded Crane	-5101981.544	7212667.785
Hooded Crane	-5099550.326	7211440.891
Hooded Crane	-4810208.706	6737564.928
Hooded Crane	-4812775.733	6735011.714
Hooded Crane	-5100743.671	7214120.082
Hooded Crane	-4964542.048	7252588.985
Hooded Crane	-4813831.042	6734666.796
Hooded Crane	-5103159.304	7215196.598
Hooded Crane	-5100273.903	7214270.862
Hooded Crane	-5101262.42	7214692.678
Hooded Crane	-5100008.962	7210162.684

Hooded Crane	-4856326.144	6956471.984
Hooded Crane	-4812167.929	6737067.159
Hooded Crane	-4812150.117	6737086.925
Hooded Crane	-5100262.771	7214289.948
Hooded Crane	-4811945.29	6735430.307
Hooded Crane	-5101019.743	7212776.555
Hooded Crane	-5101761.131	7212599.089
Hooded Crane	-4812167.929	6737068.956
Hooded Crane	-5100238.28	7211503.853
Hooded Crane	-5100080.207	7211908.347
Hooded Crane	-5100280.582	7214274.679
Hooded Crane	-4812150.117	6737095.91
Hooded Crane	-5097132.467	7217869.443
Hooded Crane	-5103368.585	7215551.652
Hooded Crane	-5099641.608	7210746.436
Hooded Crane	-5100133.64	7209760.188
Hooded Crane	-4814325.3	6734654.221
Hooded Crane	-5093271.907	7221177.405
Hooded Crane	-5099672.777	7211313.061
Hooded Crane	-5101716.603	7212562.833
Hooded Crane	-4812156.797	6737063.565
Hooded Crane	-5016590.589	7272087.344
Hooded Crane	-5101039.781	7212786.096
Hooded Crane	-5099145.123	7210187.483
Hooded Crane	-5099111.727	7212788.004
Hooded Crane	-5102139.617	7212639.162
Hooded Crane	-5102702.894	7215649.008
Hooded Crane	-5093334.246	7223122.348
Hooded Crane	-4849455.505	6937645.714
Hooded Crane	-4811996.497	6735415.934
Hooded Crane	-4856508.708	6956353.777
Hooded Crane	-5100002.283	7213761.277
Hooded Crane	-5101068.724	7214274.679
Hooded Crane	-4874117.225	6967328.702
Hooded Crane	-5100921.782	7214278.496
Hooded Crane	-5099207.462	7212393.005
Hooded Crane	-4814242.924	6734557.215
Hooded Crane	-5100340.694	7214289.948
Hooded Crane	-5100968.536	7214150.619
Hooded Crane	-5100282.808	7214270.862
Hooded Crane	-5101999.355	7212400.638
Hooded Crane	-5100739.218	7214089.545
Hooded Crane	-4812303.738	6736114.833
Hooded Crane	-5100282.808	7214270.862
Hooded Crane	-5099975.566	7209754.465
Hooded Crane	-5101632.001	7213364.319
Hooded Crane	-4812150.117	6737056.377
Hooded Crane	-5101937.016	7212856.702
Hooded Crane	-5091219.175	7227401.787
Hooded Crane	-4855524.644	6956348.236
Hooded Crane	-5100946.272	7212797.546
Hooded Crane	-4856330.597	6956479.372
Hooded Crane	-5099672.777	7209996.723
Hooded Crane	-5106198.326	7225966.126
Hooded Crane	-5097125.788	7217856.077
Hooded Crane	-4964539.821	7252600.493
Hooded Crane	-5101638.68	7212765.105
Hooded Crane	-5097121.335	7217859.896

Hooded Crane	-5103304.019	7214152.528
Hooded Crane	-5100287.261	7214257.501
Hooded Crane	-5086603.869	7221851.776
Hooded Crane	-5091775.773	7219588.186
Hooded Crane	-4812161.249	6737065.362
Hooded Crane	-5100968.536	7212801.362
Hooded Crane	-5099815.266	7209344.36
Hooded Crane	-5097128.014	7217857.986
Hooded Crane	-5103212.737	7215612.738
Hooded Crane	-5097119.108	7217859.896
Hooded Crane	-4812150.117	6737088.722
Hooded Crane	-5100256.092	7214240.324
Hooded Crane	-4812619.886	6736132.801
Hooded Crane	-4812154.57	6737077.94
Hooded Crane	-5100979.668	7214677.409
Hooded Crane	-5100895.065	7214148.711
Hooded Crane	-5100260.544	7214263.227
Hooded Crane	-5101580.794	7213440.656
Hooded Crane	-4856341.729	6956708.404
Hooded Crane	-5101104.346	7214505.626
Hooded Crane	-5101482.832	7213370.045
Hooded Crane	-5101772.263	7212671.601
Hooded Crane	-5101734.414	7213249.816
Hooded Crane	-5099405.611	7209954.757
Hooded Crane	-5100975.216	7212831.894
Hooded Crane	-4812152.344	6737067.159
Hooded Crane	-4892988.105	6953011.46
Hooded Crane	-5100260.544	7214274.679
Hooded Crane	-4809872.521	6737480.467
Hooded Crane	-5100264.997	7214265.136
Hooded Crane	-4818252.652	6742127.154
Hooded Crane	-4814282.999	6734754.82
Hooded Crane	-5100814.915	7214141.077
Hooded Crane	-4812165.702	6737133.646
Hooded Crane	-5101030.875	7214454.091
Hooded Crane	-5099946.623	7210387.786
Hooded Crane	-4810865.491	6737446.323
Hooded Crane	-5100993.027	7212822.353
Hooded Crane	-5099875.379	7210343.91
Hooded Crane	-4812379.436	6736181.313
Hooded Crane	-5099939.944	7211744.257
Hooded Crane	-4809591.996	6737604.464
Hooded Crane	-4810562.702	6737379.834
Hooded Crane	-4814303.036	6735654.882
Hooded Crane	-5100583.371	7214662.139
Hooded Crane	-4812145.665	6737086.925
Hooded Crane	-5100320.657	7214343.39
Hooded Crane	-5093830.731	7223481.585
Hooded Crane	-5101115.478	7214215.512
Hooded Crane	-4963337.571	7249373.089
Hooded Crane	-4812132.306	6737050.986
Hooded Crane	-4812130.08	6737063.565
Hooded Crane	-4812136.759	6737067.159
Hooded Crane	-4812150.117	6737054.58
Hooded Crane	-4873843.379	6966368.852
Hooded Crane	-5100146.998	7209487.418
Hooded Crane	-5100338.468	7214186.883
Hooded Crane	-4812174.608	6737115.676

Hooded Crane	-4856341.729	6956710.251
Hooded Crane	-5099603.759	7211030.697
Hooded Crane	-4812161.249	6737065.362
Hooded Crane	-5099922.133	7210092.103
Hooded Crane	-5097128.014	7217846.529
Hooded Crane	-5103201.605	7215150.786
Hooded Crane	-5100336.242	7214309.034
Hooded Crane	-4812145.665	6737068.956
Hooded Crane	-5101001.932	7214696.496
Hooded Crane	-5098911.352	7205465.645
Hooded Crane	-5100979.668	7212912.042
Hooded Crane	-5101763.358	7212637.253
Hooded Crane	-5097121.335	7217857.986
Hooded Crane	-5101803.433	7212749.839
Hooded Crane	-5093336.472	7222990.505
Hooded Crane	-5100977.442	7212812.812
Hooded Crane	-5098975.917	7213068.523
Hooded Crane	-4812477.397	6736244.199
Hooded Crane	-5100594.503	7212015.198
Hooded Crane	-5099280.933	7210162.684
Hooded Crane	-5097123.561	7217854.167
Hooded Crane	-5100242.733	7214146.802
Hooded Crane	-5100273.903	7214274.679
Hooded Crane	-4855520.191	6956357.471
Hooded Crane	-4875043.403	6968743.722
Hooded Crane	-4812141.212	6737056.377
Hooded Crane	-5100975.216	7212816.628
Hooded Crane	-5099033.804	7212837.619
Hooded Crane	-5101104.346	7214574.338
Hooded Crane	-5099777.418	7211582.079
Hooded Crane	-5099343.272	7210145.515
Hooded Crane	-5101037.554	7212767.013
Hooded Crane	-4816914.592	6739177.051
Hooded Crane	-4875041.177	6968745.572
Hooded Crane	-5101070.95	7214268.953
Hooded Crane	-5102313.276	7215217.595
Hooded Crane	-4812143.438	6737068.956
Hooded Crane	-4964553.18	7252717.495
Hooded Crane	-5100273.903	7214272.77
Hooded Crane	-5100006.736	7209695.332
Hooded Crane	-5103350.773	7213749.826
Hooded Crane	-5100509.9	7214793.841
Hooded Crane	-5091789.131	7219605.375
Hooded Crane	-5099928.812	7209752.558
Hooded Crane	-5099530.289	7211448.522
Hooded Crane	-4812179.061	6737099.504
Hooded Crane	-4812147.891	6737045.595
Hooded Crane	-5097130.24	7217850.348
Hooded Crane	-5099826.398	7209559.901
Hooded Crane	-4812156.797	6737068.956
Hooded Crane	-5099695.041	7210057.766
Hooded Crane	-5101001.932	7212808.995
Hooded Crane	-5101665.396	7213196.382
Hooded Crane	-5100269.45	7214282.313
Hooded Crane	-4812459.586	6735516.543
Hooded Crane	-5099559.232	7210956.292
Hooded Crane	-5097128.014	7217861.805
Hooded Crane	-4812508.566	6735863.294

Hooded Crane	-5101469.474	7213345.235
Hooded Crane	-5098815.617	7213473.099
Hooded Crane	-4817912.014	6740809.272
Hooded Crane	-5101587.473	7213448.289
Hooded Crane	-5099984.472	7211860.646
Hooded Crane	-5100278.355	7214278.496
Hooded Crane	-5099784.097	7212179.294
Hooded Crane	-5097141.372	7217808.34
Hooded Crane	-5093427.754	7223206.424
Hooded Crane	-5086594.964	7221868.97
Hooded Crane	-4965172.116	7252596.657
Hooded Crane	-5101088.761	7214288.039
Hooded Crane	-5100305.072	7214242.232
Hooded Crane	-5099623.797	7211139.444
Hooded Crane	-5101077.629	7213446.381
Hooded Crane	-5100431.976	7209170.787
Hooded Crane	-5099770.739	7209239.453
Hooded Crane	-5100325.11	7208003.563
Hooded Crane	-5100264.997	7214272.77
Hooded Crane	-5103068.022	7215715.822
Hooded Crane	-5097125.788	7217846.529
Hooded Crane	-5097132.467	7217859.896
Hooded Crane	-5100300.619	7214316.669
Hooded Crane	-5086606.096	7221859.417
Hooded Crane	-5101044.234	7214236.507
Hooded Crane	-4892843.39	6953053.923
Hooded Crane	-4812143.438	6737115.676
Hooded Crane	-5101580.794	7213375.77
Hooded Crane	-5101950.374	7212854.794
Hooded Crane	-5100803.783	7214728.944
Hooded Crane	-4809721.126	6737518.205
Hooded Crane	-5100592.276	7207738.483
Hooded Crane	-5101088.761	7214484.63
Hooded Crane	-5102003.808	7212398.729
Hooded Crane	-5093240.737	7223044.006
Hooded Crane	-5099728.437	7209304.305
Hooded Crane	-4812130.08	6737068.956
Hooded Crane	-4812285.927	6735780.648
Hooded Crane	-5101044.234	7212782.279
Hooded Crane	-5102749.648	7214599.151
Hooded Crane	-4812136.759	6737072.55
Hooded Crane	-5100431.976	7207988.307
Hooded Crane	-5097128.014	7217856.077
Hooded Crane	-5100296.167	7214286.131
Hooded Crane	-4813321.199	6734778.174
Hooded Crane	-5103470.998	7211662.214
Hooded Crane	-5100073.528	7211568.723
Hooded Crane	-5100999.706	7212810.903
Hooded Crane	-5097130.24	7217856.077
Hooded Crane	-5100262.771	7214265.136
Hooded Crane	-4811217.26	6736979.109
Hooded Crane	-5101037.554	7212786.096
Hooded Crane	-5100313.978	7212253.71
Hooded Crane	-4812187.966	6735852.514
Hooded Crane	-5100282.808	7214253.684
Hooded Crane	-4814278.546	6734803.324
Hooded Crane	-5100193.753	7211929.336
Hooded Crane	-5093766.165	7223305.787

Hooded Crane	-5099614.891	7211019.25
Hooded Crane	-4812152.344	6737054.58
Hooded Crane	-5101088.761	7214289.948
Hooded Crane	-5097969.589	7217101.861
Hooded Crane	-4964544.274	7252600.493
Hooded Crane	-5018438.492	7273489.24
Hooded Crane	-4811028.017	6736856.919
Hooded Crane	-5104243.556	7215041.983
Hooded Crane	-4812123.401	6735759.088
Hooded Crane	-5099038.256	7212997.915
Hooded Crane	-5101075.403	7214289.948
Hooded Crane	-5099091.69	7213247.908
Hooded Crane	-5100883.934	7214093.362
Hooded Crane	-5100445.335	7209138.362
Hooded Crane	-4813436.971	6734587.754
Hooded Crane	-4874313.148	6966385.496
Hooded Crane	-4811992.044	6735399.765
Hooded Crane	-5099801.908	7209350.082
Hooded Crane	-5097128.014	7217856.077
Hooded Crane	-5101079.856	7214284.222
Hooded Crane	-4812136.759	6735568.645
Hooded Crane	-5100518.806	7214662.139
Hooded Crane	-5094471.931	7220524.105
Hooded Crane	-4873658.589	6966526.045
Hooded Crane	-4812165.702	6737076.143
Hooded Crane	-4812533.057	6735577.628
Hooded Crane	-5102522.556	7214795.75
Hooded Crane	-5102326.634	7215162.239
Hooded Crane	-5100267.224	7214291.857
Hooded Crane	-4857087.57	6959006.474
Hooded Crane	-4812154.57	6737045.595
Hooded Crane	-5100285.035	7214335.755
Hooded Crane	-5101142.195	7213799.447
Hooded Crane	-5101097.667	7214435.005
Hooded Crane	-5100276.129	7214337.664
Hooded Crane	-4812132.306	6737056.377
Hooded Crane	-5099641.608	7211246.285
Hooded Crane	-5099534.741	7210280.957
Hooded Crane	-4856337.276	6956693.627
Hooded Crane	-4812170.155	6737040.205
Hooded Crane	-5097359.558	7217970.648
Hooded Crane	-5099590.401	7210263.788
Hooded Crane	-5103353	7215538.289
Hooded Crane	-5101872.451	7212560.925
Hooded Crane	-5099249.763	7212393.005
Hooded Crane	-5101843.508	7212259.435
Hooded Crane	-5102041.656	7215269.134
Hooded Crane	-5100113.603	7208779.786
Hooded Crane	-5100280.582	7214270.862
Hooded Crane	-5099632.702	7210998.264
Hooded Crane	-5102999.004	7214971.357
Hooded Crane	-4812125.627	6737058.174
Hooded Crane	-5100743.671	7214085.728
Hooded Crane	-5099775.191	7209346.268
Hooded Crane	-4812156.797	6737058.174
Hooded Crane	-5099695.041	7211452.338
Hooded Crane	-5101576.341	7213461.648
Hooded Crane	-5100924.009	7214673.591

Hooded Crane	-5100280.582	7214272.77
Hooded Crane	-5101972.638	7212679.234
Hooded Crane	-5099759.607	7209399.675
Hooded Crane	-5100981.895	7212831.894
Hooded Crane	-5100296.167	7214312.851
Hooded Crane	-5102041.656	7212938.758
Hooded Crane	-5101139.968	7214999.989
Hooded Crane	-5100262.771	7214280.405
Hooded Crane	-5101037.554	7212784.188
Hooded Crane	-5099919.907	7212110.602
Hooded Crane	-5100763.708	7214644.96
Hooded Crane	-5097105.75	7217844.62
Hooded Crane	-5103355.226	7215538.289
Hooded Crane	-5099804.134	7209321.471
Hooded Crane	-5099102.822	7213177.299
Hooded Crane	-5101527.36	7213518.901
Hooded Crane	-5100908.424	7213759.368
Hooded Crane	-5097148.051	7217798.792
Hooded Crane	-5099096.143	7210111.179
Hooded Crane	-5101075.403	7214265.136
Hooded Crane	-5102631.65	7215366.487
Hooded Crane	-5099953.303	7209365.342
Hooded Crane	-5100258.318	7208327.775
Hooded Crane	-5100282.808	7214268.953
Hooded Crane	-4813303.387	6735110.521
Hooded Crane	-5100972.989	7212822.353
Hooded Crane	-5097119.108	7217856.077
Hooded Crane	-5100298.393	7214331.938
Hooded Crane	-5102144.07	7215057.253
Hooded Crane	-5100300.619	7214349.116
Hooded Crane	-5100264.997	7214272.77
Hooded Crane	-5099719.532	7210183.668
Hooded Crane	-5099666.098	7209811.691
Hooded Crane	-5100264.997	7214286.131
Hooded Crane	-4873990.321	6967397.135
Hooded Crane	-5101638.68	7214427.37
Hooded Crane	-5099603.759	7210113.086
Hooded Crane	-5100984.121	7212904.409
Hooded Crane	-5100977.442	7207664.109
Hooded Crane	-5099191.877	7212396.821
Hooded Crane	-5094460.799	7220623.434
Hooded Crane	-5100993.027	7212818.536
Hooded Crane	-4812726.752	6736909.029
Hooded Crane	-4814287.452	6734774.581
Hooded Crane	-5100369.637	7212228.905
Hooded Crane	-5097103.524	7217829.344
Hooded Crane	-5101965.959	7212976.924
Hooded Crane	-5100966.31	7212816.628
Hooded Crane	-5097132.467	7217857.986
Hooded Crane	-4812190.192	6735459.052
Hooded Crane	-5099410.063	7211803.405
Hooded Crane	-5015490.752	7277457.845
Hooded Crane	-4811934.158	6735451.866
Hooded Crane	-5099027.124	7213099.056
Hooded Crane	-5100267.224	7214265.136
Hooded Crane	-5102751.875	7215450.479
Hooded Crane	-5099585.948	7211061.222
Hooded Crane	-5101001.932	7214448.365

Hooded Crane	-5101551.85	7213509.359
Hooded Crane	-5099594.854	7210962.016
Hooded Crane	-5097192.579	7217819.797
Hooded Crane	-5100298.393	7214307.126
Hooded Crane	-5102409.011	7206544.779
Hooded Crane	-5100761.482	7214118.173
Hooded Crane	-5101119.931	7213455.923
Hooded Crane	-5101400.456	7212988.374
Hooded Crane	-5102081.731	7215292.041
Hooded Crane	-5101224.571	7214656.413
Hooded Crane	-4812163.476	6737063.565
Hooded Crane	-5101135.516	7213478.824
Hooded Crane	-5101048.686	7208013.099
Hooded Crane	-5099577.043	7211087.932
Hooded Crane	-4817466.736	6740541.407
Hooded Crane	-5106289.608	7225977.595
Hooded Crane	-5100273.903	7214270.862
Hooded Crane	-5102645.008	7215851.36
Hooded Crane	-5099735.116	7209350.082
Hooded Crane	-4812136.759	6737067.159
Hooded Crane	-5099530.289	7209186.046
Hooded Crane	-5100839.406	7214230.781
Hooded Crane	-5094460.799	7220545.117
Hooded Crane	-5099663.872	7210172.222
Hooded Crane	-5099561.458	7209296.675
Hooded Crane	-4812136.759	6737059.971
Hooded Crane	-5100262.771	7214286.131
Hooded Crane	-5102072.826	7215282.496
Hooded Crane	-4814236.245	6734542.844
Hooded Crane	-5099570.364	7209355.805
Hooded Crane	-5098989.276	7213104.781
Hooded Crane	-5099541.42	7209187.954
Hooded Crane	-5100863.896	7214364.385
Hooded Crane	-5099203.009	7212591.456
Hooded Crane	-5101863.545	7212940.666
Hooded Crane	-5100302.846	7214320.486
Hooded Crane	-4812134.533	6737029.423
Hooded Crane	-5100345.147	7214259.41
Hooded Crane	-4814229.566	6734567.994
Hooded Crane	-5099801.908	7209321.471
Hooded Crane	-5101039.781	7212786.096
Hooded Crane	-5101591.925	7213473.099
Hooded Crane	-5100276.129	7214782.389
Hooded Crane	-4811934.158	6735631.526
Hooded Crane	-5102139.617	7215072.524
Hooded Crane	-5100298.393	7212259.435
Hooded Crane	-4809832.446	6738132.819
Hooded Crane	-5101251.288	7214482.721
Hooded Crane	-5101144.421	7213287.984
Hooded Crane	-5097246.013	7207919.652
Hooded Crane	-5100267.224	7214272.77
Hooded Crane	-5100975.216	7212808.995
Hooded Crane	-5097123.561	7217850.348
Hooded Crane	-4812154.57	6737070.753
Hooded Crane	-4812147.891	6737072.55
Hooded Crane	-4812134.533	6737056.377
Hooded Crane	-5100240.507	7214276.588
Hooded Crane	-5100846.085	7214228.872

Hooded Crane	-5102070.599	7215278.679
Hooded Crane	-5101473.927	7213328.06
Hooded Crane	-4812132.306	6737063.565
Hooded Crane	-5102063.92	7215290.132
Hooded Crane	-5100454.24	7209147.899
Hooded Crane	-5100975.216	7212826.17
Hooded Crane	-4812127.854	6737042.001
Hooded Crane	-5100020.094	7209248.99
Hooded Crane	-5099886.511	7210378.247
Hooded Crane	-4812138.986	6737061.768
Hooded Crane	-4818263.784	6742058.828
Hooded Crane	-4812145.665	6737160.6
Hooded Crane	-4812150.117	6737081.534
Hooded Crane	-4809805.729	6738122.036
Hooded Crane	-5100988.574	7212780.371
Hooded Crane	-5100403.033	7207866.254
Hooded Crane	-5100705.822	7213900.598
Hooded Crane	-5101754.452	7212671.601
Hooded Crane	-4812326.002	6737022.235
Hooded Crane	-5102653.913	7206643.929
Hooded Crane	-5100017.868	7211782.417
Hooded Crane	-4888818.077	6969788.964
Hooded Crane	-5100436.429	7207994.028
Hooded Crane	-5097119.108	7217856.077
Hooded Crane	-4809596.448	6737615.246
Hooded Crane	-5101073.177	7214293.765
Hooded Crane	-5100302.846	7214337.664
Hooded Crane	-5100271.676	7214284.222
Hooded Crane	-5100278.355	7214297.582
Hooded Crane	-4812181.287	6737079.737
Hooded Crane	-5097130.24	7217861.805
Hooded Crane	-5100271.676	7214268.953
Hooded Crane	-5097555.481	7206876.557
Hooded Crane	-5099895.416	7208924.74
Hooded Crane	-5101948.148	7213053.257
Hooded Crane	-4810758.624	6737485.858
Hooded Crane	-5092474.859	7220936.709
Hooded Crane	-5102061.694	7214717.492
Hooded Crane	-5100149.225	7208648.185
Hooded Crane	-5100447.561	7209155.528
Hooded Crane	-4812003.176	6737532.581
Hooded Crane	-5099775.191	7209317.656
Hooded Crane	-5100244.96	7214293.765
Hooded Crane	-5101304.721	7214507.534
Hooded Crane	-5100763.708	7214116.265
Hooded Crane	-5097145.825	7217812.159
Hooded Crane	-4812147.891	6737059.971
Hooded Crane	-4964535.368	7252729.003
Hooded Crane	-4888951.661	6969885.171
Hooded Crane	-4812755.696	6736932.389
Hooded Crane	-4812969.429	6735457.255
Hooded Crane	-5101088.761	7214288.039
Hooded Crane	-5099677.23	7209685.795
Hooded Crane	-5100267.224	7214288.039
Hooded Crane	-5101647.585	7214805.294
Hooded Crane	-5102117.353	7215047.709
Hooded Crane	-5100269.45	7214288.039
Hooded Crane	-4810645.078	6737223.495

Hooded Crane	-4893558.061	6950796.337
Hooded Crane	-5100347.374	7212030.463
Hooded Crane	-4811230.618	6738175.951
Hooded Crane	-5100207.111	7208297.26
Hooded Crane	-4812392.794	6737068.956
Hooded Crane	-4875128.006	6968826.966
Hooded Crane	-5099552.552	7209207.028
Hooded Crane	-4812769.054	6736853.325
Hooded Crane	-5101556.303	7213163.94
Hooded Crane	-4812143.438	6737050.986
Hooded Crane	-4873814.436	6967585.791
Hooded Crane	-5100311.751	7214291.857
Hooded Crane	-5100494.315	7214583.882
Hooded Crane	-5101208.986	7214936.999
Hooded Crane	-5100289.487	7214305.217
Hooded Crane	-5093305.303	7222938.914
Hooded Crane	-4887072.588	6950683.751
Hooded Crane	-5099795.229	7209361.527
Hooded Crane	-4816482.672	6738202.909
Hooded Crane	-4811994.27	6735448.272
Hooded Crane	-5102420.142	7212686.867
Hooded Crane	-4810925.603	6736416.69
Hooded Crane	-4812156.797	6737059.971
Hooded Crane	-5100289.487	7214270.862
Hooded Crane	-5100975.216	7212812.812
Hooded Crane	-5097128.014	7217871.353
Hooded Crane	-4813617.308	6734629.071
Hooded Crane	-4964501.973	7252021.264
Hooded Crane	-5097920.609	7221114.365
Hooded Crane	-5100264.997	7214289.948
Hooded Crane	-5099479.082	7212076.257
Hooded Crane	-5099793.003	7209338.638
Hooded Crane	-4810856.585	6736670.043
Hooded Crane	-5099559.232	7213669.669
Hooded Crane	-5103535.564	7215538.289
Hooded Crane	-5093407.717	7223053.56
Hooded Crane	-5093559.111	7223340.182
Hooded Crane	-5100253.865	7214274.679
Hooded Crane	-5101082.082	7214289.948
Hooded Crane	-4964326.088	7251560.981
Hooded Crane	-5099893.19	7211778.601
Hooded Crane	-5101542.945	7213505.542
Hooded Crane	-5100837.179	7214158.254
Hooded Crane	-4810567.154	6737216.307
Hooded Crane	-5100915.103	7213784.178
Hooded Crane	-5100269.45	7214284.222
Hooded Crane	-5100839.406	7214219.329
Hooded Crane	-5101698.792	7215360.761
Hooded Crane	-5100305.072	7214316.669
Hooded Crane	-5101458.342	7212694.5
Hooded Crane	-5100273.903	7214274.679
Hooded Crane	-4812702.262	6736930.592
Hooded Crane	-5099795.229	7209306.212
Hooded Crane	-5099570.364	7209336.731
Hooded Crane	-5102006.034	7212663.968
Hooded Crane	-5101079.856	7214288.039
Hooded Crane	-5099801.908	7209363.434
Hooded Crane	-5101021.97	7214608.695

Hooded Crane	-5100280.582	7214259.41
Hooded Crane	-5101273.552	7213125.773
Hooded Crane	-4810190.894	6737525.393
Hooded Crane	-5102157.428	7215051.527
Hooded Crane	-5101108.799	7208064.59
Hooded Crane	-5100316.204	7212236.537
Hooded Crane	-5100852.764	7212017.106
Hooded Crane	-4812165.702	6737076.143
Hooded Crane	-4812724.526	6736979.109
Hooded Crane	-5099739.569	7209380.601
Hooded Crane	-5100543.296	7214095.271
Hooded Crane	-5099516.93	7211229.114
Hooded Crane	-5100972.989	7212803.27
Hooded Crane	-5099746.248	7209273.786
Hooded Crane	-5099650.514	7210723.543
Hooded Crane	-4812130.08	6737049.189
Hooded Crane	-5099002.634	7213037.99
Hooded Crane	-5099706.173	7211494.313
Hooded Crane	-4812141.212	6737083.331
Hooded Crane	-4875506.492	6968564.287
Hooded Crane	-4812134.533	6737049.189
Hooded Crane	-4812909.316	6736788.637
Hooded Crane	-5099539.194	7209193.676
Hooded Crane	-5097119.108	7217861.805
Hooded Crane	-5102124.033	7215082.068
Hooded Crane	-5100394.128	7208158.039
Hooded Crane	-5100296.167	7214333.846
Hooded Crane	-5101551.85	7212971.199
Hooded Crane	-5099846.436	7211944.6
Hooded Crane	-5100118.055	7208690.145
Hooded Crane	-4810685.153	6737430.15
Hooded Crane	-5098942.522	7212963.566
Hooded Crane	-4812150.117	6737042.001
Hooded Crane	-5101070.95	7208062.683
Hooded Crane	-5100287.261	7214289.948
Hooded Crane	-5099238.631	7212391.097
Hooded Crane	-5100271.676	7214284.222
Hooded Crane	-5099131.765	7210147.423
Hooded Crane	-5101540.719	7213497.908
Hooded Crane	-5100262.771	7214274.679
Hooded Crane	-5100307.299	7214293.765
Hooded Crane	-5097148.051	7217806.43
Hooded Crane	-4812127.854	6737045.595
Hooded Crane	-5100549.975	7214310.943
Hooded Crane	-4812138.986	6737056.377
Hooded Crane	-5747006.748	6509301.793
Hooded Crane	-4812163.476	6737079.737
Hooded Crane	-4812132.306	6737076.143
Hooded Crane	-4812506.34	6736260.37
Hooded Crane	-5100409.712	7207896.767
Hooded Crane	-5100445.335	7209144.084
Hooded Crane	-5102014.94	7212600.997
Hooded Crane	-5099349.951	7210042.505
Hooded Crane	-5100977.442	7212784.188
Hooded Crane	-5099200.783	7213011.274
Hooded Crane	-5101184.496	7214448.365
Hooded Crane	-5101077.629	7214282.313
Hooded Crane	-5101727.735	7212885.326

Hooded Crane	-5097729.139	7207229.325
Hooded Crane	-4816560.596	6738945.187
Hooded Crane	-5102039.43	7212967.383
Hooded Crane	-5100400.807	7213975.031
Hooded Crane	-5101916.978	7213028.448
Hooded Crane	-5099706.173	7210153.146
Hooded Crane	-4813269.992	6735128.486
Hooded Crane	-4812691.13	6736883.872
Hooded Crane	-5101643.132	7213389.129
Hooded Crane	-4812141.212	6737050.986
Hooded Crane	-4812194.645	6737034.814
Hooded Crane	-5100458.693	7213975.031
Hooded Crane	-5100779.293	7213803.264
Hooded Crane	-5099748.475	7209626.663
Hooded Crane	-4813051.805	6734943.448
Hooded Crane	-5100271.676	7214299.491
Hooded Crane	-5099931.039	7209702.962
Hooded Crane	-4812154.57	6737050.986
Hooded Crane	-5099626.023	7210036.782
Hooded Crane	-4812154.57	6737061.768
Hooded Crane	-5101253.514	7213392.945
Hooded Crane	-4818662.308	6741697.425
Hooded Crane	-4811201.675	6736722.152
Hooded Crane	-4812136.759	6737086.925
Hooded Crane	-5097143.599	7217802.611
Hooded Crane	-5100460.919	7209226.101
Hooded Crane	-5100293.94	7214282.313
Hooded Crane	-4814229.566	6734541.048
Hooded Crane	-5099681.683	7210080.657
Hooded Crane	-5100278.355	7214280.405
Hooded Crane	-4812203.551	6737162.397
Hooded Crane	-5100249.412	7208289.631
Hooded Crane	-4815589.89	6740857.812
Hooded Crane	-5099552.552	7209107.844
Hooded Crane	-4875495.361	6968575.386
Hooded Crane	-5099545.873	7209191.769
Hooded Crane	-4812141.212	6737101.301
Hooded Crane	-4884710.388	6959595.878
Hooded Crane	-5099372.215	7210145.515
Hooded Crane	-5016036.218	7272729.608
Hooded Crane	-4812154.57	6737042.001
Hooded Crane	-4964566.538	7252581.312
Hooded Crane	-5100264.997	7214280.405
Hooded Crane	-5100269.45	7214286.131
Hooded Crane	-4812713.394	6736937.78
Hooded Crane	-5093267.454	7222698.164
Hooded Crane	-5100269.45	7212268.975
Hooded Crane	-5100946.272	7212786.096
Hooded Crane	-4812094.458	6735874.074
Hooded Crane	-5101467.248	7213335.693
Hooded Crane	-5098866.824	7213030.357
Hooded Crane	-5101876.903	7213053.257
Hooded Crane	-5103056.89	7215427.572
Hooded Crane	-5099307.65	7210198.929
Hooded Crane	-4812399.473	6736335.835
Hooded Crane	-5099766.286	7209336.731
Hooded Crane	-4893382.176	6952610.846
Hooded Crane	-5101516.228	7213454.015

Hooded Crane	-5017189.488	7269797.548
Hooded Crane	-4812170.155	6737061.768
Hooded Crane	-5100244.96	7214274.679
Hooded Crane	-4812165.702	6737049.189
Hooded Crane	-5102052.788	7212587.64
Hooded Crane	-4812101.137	6737050.986
Hooded Crane	-5097130.24	7217869.443
Hooded Crane	-5100999.706	7212812.812
Hooded Crane	-4817649.3	6741670.456
Hooded Crane	-5101066.497	7214923.637
Hooded Crane	-5102137.391	7215043.892
Hooded Crane	-5093227.379	7223003.88
Hooded Crane	-5100768.161	7214303.308
Hooded Crane	-5100318.431	7212253.71
Hooded Crane	-4892142.077	6952444.699
Hooded Crane	-5099821.946	7209361.527
Hooded Crane	-5100285.035	7214251.775
Hooded Crane	-5099102.822	7210267.604
Hooded Crane	-5101295.816	7212933.033
Hooded Crane	-5099868.7	7211931.244
Hooded Crane	-5098793.353	7213142.948
Hooded Crane	-4892493.847	6952738.228
Hooded Crane	-4812159.023	6737076.143
Hooded Crane	-5099955.529	7208487.978
Hooded Crane	-5097136.919	7217840.801
Hooded Crane	-5100313.978	7212276.608
Hooded Crane	-4812132.306	6737083.331
Hooded Crane	-5094491.968	7220529.836
Hooded Crane	-5100521.032	7214234.598
Hooded Crane	-4812150.117	6737040.205
Hooded Crane	-4812141.212	6737036.611
Hooded Crane	-4896672.78	6952758.536
Hooded Crane	-5098964.786	7213045.623
Hooded Crane	-5093383.226	7223021.077
Hooded Crane	-4812582.037	6735457.255
Hooded Crane	-4875497.587	6968567.987
Hooded Crane	-4894154.734	6953609.65
Hooded Crane	-4812125.627	6737040.205
Hooded Crane	-5101068.724	7208074.126
Hooded Crane	-4884895.178	6959557.076
Hooded Crane	-4814084.85	6734785.36
Hooded Crane	-5100258.318	7214274.679
Hooded Crane	-4812110.042	6737050.986
Hooded Crane	-5099550.326	7209187.954
Hooded Crane	-4812190.192	6737036.611
Hooded Crane	-5099748.475	7209323.379
Hooded Crane	-4812167.929	6737173.179
Hooded Crane	-4812159.023	6737099.504
Hooded Crane	-5099539.194	7209460.713
Hooded Crane	-5100229.375	7214314.76
Hooded Crane	-4855526.87	6956359.318
Hooded Crane	-5099973.34	7209224.194
Hooded Crane	-5099414.516	7210425.939
Hooded Crane	-5100979.668	7212852.885
Hooded Crane	-4812159.023	6737063.565
Hooded Crane	-4812228.041	6735275.802
Hooded Crane	-4812150.117	6737072.55
Hooded Crane	-5103640.204	7215406.574

Hooded Crane	-5101358.155	7213503.634
Hooded Crane	-4812700.036	6736891.06
Hooded Crane	-4812134.533	6737072.55
Hooded Crane	-4812143.438	6737077.94
Hooded Crane	-5100975.216	7213965.488
Hooded Crane	-4812159.023	6737097.707
Hooded Crane	-5100305.072	7214232.689
Hooded Crane	-4971900.266	7328687.296
Hooded Crane	-4892162.115	6952437.315
Hooded Crane	-4810449.156	6737613.449
Hooded Crane	-5100883.934	7214148.711
Hooded Crane	-5100489.863	7214448.365
Hooded Crane	-5100269.45	7214301.4
Hooded Crane	-4886097.429	6949316.241
Hooded Crane	-5018857.054	7273891.202
Hooded Crane	-5101509.549	7213497.908
Hooded Crane	-5097119.108	7217850.348
Hooded Crane	-5099877.605	7210324.833
Hooded Crane	-5099000.408	7212967.383
Hooded Crane	-5101075.403	7208060.776
Hooded Crane	-5101222.345	7214774.754
Hooded Crane	-4888439.591	6969731.611
Hooded Crane	-4812528.604	6736068.118
Hooded Crane	-5100258.318	7214270.862
Hooded Crane	-5099597.08	7209266.157
Hooded Crane	-5100841.632	7214301.4
Hooded Crane	-4892852.296	6952839.766
Hooded Crane	-4812361.625	6737286.389
Hooded Crane	-5100425.297	7209197.491
Hooded Crane	-4812152.344	6737042.001
Hooded Crane	-4812455.133	6735600.984
Hooded Crane	-4816553.917	6741364.806
Hooded Crane	-5100253.865	7214207.877
Hooded Crane	-4812127.854	6737027.626
Hooded Crane	-5102567.084	7212600.997
Hooded Crane	-4812150.117	6737108.489
Hooded Crane	-5099868.7	7210349.632
Hooded Crane	-4885897.054	6949364.219
Hooded Crane	-4812143.438	6737074.346
Hooded Crane	-4963845.188	7250324.104
Hooded Crane	-5101948.148	7212763.197
Hooded Crane	-4892006.267	6950919.998
Hooded Crane	-4812141.212	6737110.286
Hooded Crane	-5075563.202	7240296.372
Hooded Crane	-4895426.002	6947288.478
Hooded Crane	-4812141.212	6737068.956
Hooded Crane	-5100423.071	7209189.861
Hooded Crane	-5100280.582	7214284.222
Hooded Crane	-5091771.32	7219626.384
Hooded Crane	-5097459.746	7206949.016
Hooded Crane	-5102126.259	7215034.347
Hooded Crane	-4812167.929	6737126.458
Hooded Crane	-4883708.513	6960177.934
Hooded Crane	-5102736.29	7212316.679
Hooded Crane	-5099877.605	7208907.575
Hooded Crane	-4894194.809	6950953.221
Hooded Crane	-4893050.444	6953201.621
Hooded Crane	-4886524.896	6950521.335

Hooded Crane	-4889241.091	6969583.605
Hooded Crane	-5099790.776	7211801.497
Hooded Crane	-5100427.524	7209182.232
Hooded Crane	-5100249.412	7214268.953
Hooded Crane	-4888700.079	6969424.501
Hooded Crane	-4812150.117	6737054.58
Hooded Crane	-5102651.687	7205671.546
Hooded Crane	-4892041.89	6952387.471
Hooded Crane	-5099657.193	7210801.761
Hooded Crane	-5096940.997	7207995.935
Hooded Crane	-5100133.64	7209334.823
Hooded Crane	-5098290.189	7201232.535
Hooded Crane	-5100358.506	7212280.424
Hooded Crane	-5019674.139	7274458.6
Hooded Crane	-5099732.89	7209298.582
Hooded Crane	-4897040.135	6952680.998
Hooded Crane	-4812176.834	6737097.707
Hooded Crane	-4818666.76	6741702.819
Hooded Crane	-5099443.459	7205147.271
Hooded Crane	-4901421.67	6961800.525
Hooded Crane	-5100264.997	7214295.674
Hooded Crane	-4812161.249	6737077.94
Hooded Crane	-4888624.381	6969332.001
Hooded Crane	-5101119.931	7212835.711
Hooded Crane	-4888597.665	6968865.814
Hooded Crane	-4888379.478	6969165.502
Hooded Crane	-5101068.724	7208053.148
Hooded Crane	-5099530.289	7209199.398
Hooded Crane	-4887776.127	6987551.427
Hooded Crane	-4892286.793	6952893.305
Hooded Crane	-5100476.504	7211969.405
Hooded Crane	-4888533.099	6968930.56
Hooded Crane	-5100227.149	7208234.324
Hooded Crane	-5101663.17	7213272.717
Hooded Crane	-5019576.177	7275228.02
Hooded Crane	-4896931.042	6952439.161
Hooded Crane	-5100342.921	7209012.477
Hooded Crane	-4888065.557	6969511.453
Hooded Crane	-4891572.121	6952084.725
Hooded Crane	-5100834.953	7208367.825
Hooded Crane	-5100690.238	7208526.122
Hooded Crane	-4888521.967	6968801.068
Hooded Crane	-4889196.563	6969726.061
Hooded Crane	-5100970.763	7212768.922
Hooded Crane	-5100961.857	7212818.536
Hooded Crane	-4888840.341	6969644.657
Hooded Crane	-4818644.497	6741722.597
Hooded Crane	-4812069.967	6737018.641
Hooded Crane	-5099764.059	7209273.786
Hooded Crane	-4814222.886	6734548.233
Hooded Crane	-4812165.702	6737049.189
Hooded Crane	-4812145.665	6737103.098
Hooded Crane	-4896919.91	6952127.182
Hooded Crane	-4812624.339	6736919.81
Hooded Crane	-4812406.152	6737333.111
Hooded Crane	-4888415.101	6969581.755
Hooded Crane	-5096667.151	7207521.085
Hooded Crane	-4888488.571	6969232.101

Hooded Crane	-4889290.072	6970242.254
Hooded Crane	-4894648.992	6950595.16
Hooded Crane	-5097107.976	7217892.358
Hooded Crane	-4885856.979	6949174.151
Hooded Crane	-4812054.383	6737043.798
Hooded Crane	-4889011.773	6969990.629
Hooded Crane	-5101398.23	7213537.986
Hooded Crane	-4889080.791	6969973.977
Hooded Crane	-4888530.873	6969554.004
Hooded Crane	-5100320.657	7214257.501
Hooded Crane	-4891788.081	6952175.178
Hooded Crane	-5096709.453	7208569.988
Hooded Crane	-5099539.194	7210916.228
Hooded Crane	-4812094.458	6737007.86
Hooded Crane	-5100937.367	7212765.105
Hooded Crane	-4893880.888	6950285.1
Hooded Crane	-5099610.439	7211150.891
Hooded Crane	-4812472.944	6737139.037
Hooded Crane	-4812165.702	6737061.768
Hooded Crane	-4888170.198	6969441.152
Hooded Crane	-4813926.777	6734760.21
Hooded Crane	-5103279.529	7204853.693
Hooded Crane	-4812125.627	6737024.032
Hooded Crane	-5102558.179	7214604.878
Hooded Crane	-4812141.212	6737042.001
Hooded Crane	-5097103.524	7217835.072
Hooded Crane	-4888760.191	6969067.455
Hooded Crane	-5099991.151	7209233.731
Hooded Crane	-4815821.434	6736707.777
Hooded Crane	-4810825.416	6737500.235
Hooded Crane	-4891460.802	6952400.393
Hooded Crane	-4893767.342	6950759.423
Hooded Crane	-5100754.803	7214110.539
Hooded Crane	-4812114.495	6737063.565
Hooded Crane	-4891253.748	6952007.194
Hooded Crane	-5022521.691	7267977.344
Hooded Crane	-4898213.442	6958655.44
Hooded Crane	-4888268.159	6970429.128
Hooded Crane	-4889201.016	6969746.412
Hooded Crane	-4813256.633	6735108.725
Hooded Crane	-4809449.507	6737825.506
Hooded Crane	-4856165.844	6956370.4
Hooded Crane	-5100053.49	7208688.237
Hooded Crane	-4891235.937	6952103.185
Hooded Crane	-4892830.032	6952703.152
Hooded Crane	-4891596.612	6951975.813
Hooded Crane	-4892442.64	6950528.718
Hooded Crane	-4813312.293	6735112.318
Hooded Crane	-4812141.212	6737070.753
Hooded Crane	-4812143.438	6737036.611
Hooded Crane	-5100975.216	7214379.654
Hooded Crane	-4812090.005	6737061.768
Hooded Crane	-5102119.58	7215015.259
Hooded Crane	-4812161.249	6737081.534
Hooded Crane	-5096460.097	7208159.946
Hooded Crane	-4888720.116	6970121.991
Hooded Crane	-4888860.379	6968973.108
Hooded Crane	-4971918.077	7328869.373

Hooded Crane	-4888713.437	6968680.827
Hooded Crane	-4812132.306	6737038.408
Hooded Crane	-4812123.401	6737043.798
Hooded Crane	-4812087.779	6735699.798
Hooded Crane	-5099274.254	7210158.869
Hooded Crane	-5101551.85	7212683.051
Hooded Crane	-5100952.952	7212812.812
Hooded Crane	-5101916.978	7212629.62
Hooded Crane	-4888533.099	6968839.915
Hooded Crane	-4889049.622	6969187.702
Hooded Crane	-4888927.17	6968704.875
Hooded Crane	-4812132.306	6735760.884
Hooded Crane	-4885952.713	6949120.638
Hooded Crane	-4888457.402	6968801.068
Hooded Crane	-4890964.317	6945062.042
Hooded Crane	-4812123.401	6737083.331
Hooded Crane	-4812161.249	6737110.286
Hooded Crane	-5099719.532	7209649.553
Hooded Crane	-5100271.676	7214242.232
Hooded Crane	-5099864.247	7210378.247
Hooded Crane	-5100512.126	7209157.436
Hooded Crane	-5101812.338	7212755.564
Hooded Crane	-5097252.692	7208680.608
Hooded Crane	-4888038.841	6969559.554
Hooded Crane	-4895238.985	6950916.307
Hooded Crane	-4898046.463	6958624.032
Hooded Crane	-5017216.204	7269809.082
Hooded Crane	-4812179.061	6737065.362
Hooded Crane	-5102789.723	7205570.501
Hooded Crane	-4810155.272	6737848.869
Hooded Crane	-4811177.185	6736914.42
Hooded Crane	-4897915.106	6952516.696
Hooded Crane	-4888439.591	6969677.959
Hooded Crane	-4810834.321	6737250.449
Hooded Crane	-4857000.74	6955328.773
Hooded Crane	-4812127.854	6737056.377
Hooded Crane	-4888134.575	6969877.77
Hooded Crane	-4897763.711	6956241.112
Hooded Crane	-4812187.966	6737015.047
Hooded Crane	-4894646.766	6950609.925
Hooded Crane	-4891930.57	6952345.012
Hooded Crane	-4889290.072	6970223.752
Hooded Crane	-5100961.857	7212902.501
Hooded Crane	-4888181.33	6969417.101
Hooded Crane	-4812116.722	6736889.263
Hooded Crane	-5099327.687	7210031.06
Hooded Crane	-4812219.136	6735786.038
Hooded Crane	-4812085.552	6735726.748
Hooded Crane	-4812141.212	6737063.565
Hooded Crane	-4894154.734	6951178.401
Hooded Crane	-5097114.656	7217848.439
Hooded Crane	-4812125.627	6737040.205
Hooded Crane	-4901319.256	6962066.677
Hooded Crane	-4812134.533	6737049.189
Hooded Crane	-4897024.55	6952902.536
Hooded Crane	-4892389.206	6952656.999
Hooded Crane	-4810716.322	6737419.368
Hooded Crane	-4812132.306	6737025.829

Hooded Crane	-4888170.198	6969322.751
Hooded Crane	-4811793.895	6737622.434
Hooded Crane	-4812156.797	6737050.986
Hooded Crane	-4812114.495	6737042.001
Hooded Crane	-4812170.155	6737018.641
Hooded Crane	-4891785.855	6952178.87
Hooded Crane	-4814449.978	6735708.781
Hooded Crane	-5100449.788	7209140.27
Hooded Crane	-4813530.479	6734612.903
Hooded Crane	-5096054.894	7216462.267
Hooded Crane	-4812165.702	6737015.047
Hooded Crane	-4897204.888	6952119.799
Hooded Crane	-4889205.469	6969698.31
Hooded Crane	-4888826.983	6969866.669
Hooded Crane	-4809952.671	6737563.131
Hooded Crane	-4888584.306	6969459.652
Hooded Crane	-4812818.034	6735480.611
Hooded Crane	-5101066.497	7207646.946
Hooded Crane	-4812141.212	6737139.037
Hooded Crane	-4893854.171	6950277.718
Hooded Crane	-4818657.855	6741695.627
Hooded Crane	-4812127.854	6737121.067
Hooded Crane	-4814307.489	6735627.933
Hooded Crane	-4888243.669	6968989.758
Hooded Crane	-4891287.143	6951855.827
Hooded Crane	-4893702.776	6951187.63
Hooded Crane	-4812145.665	6736980.906
Hooded Crane	-4889047.395	6969811.166
Hooded Crane	-4892814.447	6952716.075
Hooded Crane	-4893992.207	6950597.006
Hooded Crane	-5015481.847	7273989.292
Hooded Crane	-5101315.853	7213404.396
Hooded Crane	-4887818.428	6970147.894
Hooded Crane	-4889103.055	6969996.179
Hooded Crane	-5101358.155	7207116.819
Hooded Crane	-5018781.356	7272943.067
Hooded Crane	-4894228.204	6950707.744
Hooded Crane	-4894631.181	6950633.919
Hooded Crane	-4888695.626	6969435.601
Hooded Crane	-5099755.154	7209273.786
Hooded Crane	-4888599.891	6969058.205
Hooded Crane	-5099703.947	7209632.385
Hooded Crane	-4888542.005	6968738.172
Hooded Crane	-4814256.282	6735647.696
Hooded Crane	-4885899.28	6949329.158
Hooded Crane	-4898725.512	6951202.396
Hooded Crane	-4889294.525	6970212.651
Hooded Crane	-5097105.75	7217852.258
Hooded Crane	-4888332.724	6969689.059
Hooded Crane	-4888639.966	6969533.653
Hooded Crane	-4812105.59	6737052.783
Hooded Crane	-4889083.018	6969979.528
Hooded Crane	-4889201.016	6969759.363
Hooded Crane	-4891790.308	6952171.486
Hooded Crane	-4888613.249	6969204.352
Hooded Crane	-4888110.085	6969446.702
Hooded Crane	-5101905.846	7212994.099
Hooded Crane	-4895621.924	6947341.979

Hooded Crane	-5102838.704	7205498.055
Hooded Crane	-5100409.712	7209159.343
Hooded Crane	-4888546.458	6968817.717
Hooded Crane	-4896786.326	6952669.922
Hooded Crane	-4893615.947	6950626.536
Hooded Crane	-4895909.129	6954311.288
Hooded Crane	-4888642.192	6969443.002
Hooded Crane	-4888559.816	6969454.102
Hooded Crane	-4888330.498	6969640.957
Hooded Crane	-4817259.682	6741433.126
Hooded Crane	-4889100.829	6969968.427
Hooded Crane	-4888573.174	6969457.802
Hooded Crane	-4885912.638	6949338.385
Hooded Crane	-4856363.993	6956525.547
Hooded Crane	-4812461.812	6735768.071
Hooded Crane	-4888090.048	6969518.853
Hooded Crane	-4891790.308	6952178.87
Hooded Crane	-5102253.163	7212669.693
Hooded Crane	-4897256.095	6952309.937
Hooded Crane	-4812381.662	6736150.768
Hooded Crane	-4894087.942	6953912.454
Hooded Crane	-5100140.319	7209517.937
Hooded Crane	-5099510.251	7211307.337
Hooded Crane	-4813929.003	6734744.042
Hooded Crane	-5097535.443	7207454.342
Hooded Crane	-4812568.679	6735985.469
Hooded Crane	-4888366.12	6969071.155
Hooded Crane	-5100454.24	7208024.541
Hooded Crane	-4894257.147	6951158.098
Hooded Crane	-4883648.4	7090781.577
Hooded Crane	-4883948.963	7098552.049
Hooded Crane	-4888506.383	6969578.055
Hooded Crane	-4898275.781	6952601.616
Hooded Crane	-4888435.138	6969555.854
Hooded Crane	-4891763.591	6952195.484
Hooded Crane	-4814285.225	6734769.192
Hooded Crane	-4892823.352	6952719.767
Hooded Crane	-4893863.076	6950218.66
Hooded Crane	-4896145.126	6946666.784
Hooded Crane	-4888773.549	6968658.629
Hooded Crane	-5102651.687	7212448.342
Hooded Crane	-5099966.661	7210069.211
Hooded Crane	-4893767.342	6950790.8
Hooded Crane	-5100327.336	7214162.071
Hooded Crane	-4888310.46	6969668.708
Hooded Crane	-4888236.989	6969302.401
Hooded Crane	-4888170.198	6969413.401
Hooded Crane	-4888121.217	6970212.651
Hooded Crane	-4812141.212	6737045.595
Hooded Crane	-4895702.074	6947196.235
Hooded Crane	-4812150.117	6737099.504
Hooded Crane	-4888793.587	6969794.515
Hooded Crane	-4888931.623	6968714.124
Hooded Crane	-4888090.048	6987508.779
Hooded Crane	-4812159.023	6737059.971
Hooded Crane	-4891578.801	6970725.175
Hooded Crane	-4891799.213	6952182.562
Hooded Crane	-4892823.352	6952732.69

Hooded Crane	-5100204.885	7209020.106
Hooded Crane	-5090511.183	7224360.64
Hooded Crane	-4891129.07	6952232.405
Hooded Crane	-4810282.176	6737809.332
Hooded Crane	-4888259.253	6969476.302
Hooded Crane	-5100986.347	7214116.265
Hooded Crane	-5100260.544	7214289.948
Hooded Crane	-5100663.521	7214083.819
Hooded Crane	-4888481.892	6969729.761
Hooded Crane	-4896919.91	6953161.003
Hooded Crane	-4894648.992	6950515.798
Hooded Crane	-5100382.996	7208844.634
Hooded Crane	-4888526.42	6969322.751
Hooded Crane	-5719615.474	6550509.266
Hooded Crane	-5099817.493	7210059.673
Hooded Crane	-4888506.383	6969574.355
Hooded Crane	-4888179.103	6969777.864
Hooded Crane	-4892961.389	6945030.688
Hooded Crane	-4886389.086	6949858.787
Hooded Crane	-5100995.253	7214994.263
Hooded Crane	-5103655.789	7215435.208
Hooded Crane	-4888185.782	6969518.853
Hooded Crane	-4888564.269	6968778.869
Hooded Crane	-4887241.793	6989261.246
Hooded Crane	-5096691.642	7208198.088
Hooded Crane	-4888530.873	6969640.957
Hooded Crane	-4894648.992	6950600.697
Hooded Crane	-5099988.925	7209329.101
Hooded Crane	-4812165.702	6737085.128
Hooded Crane	-4897122.511	6952282.247
Hooded Crane	-4888826.983	6969925.874
Hooded Crane	-5099479.082	7210923.859
Hooded Crane	-5100679.106	7214927.455
Hooded Crane	-4888499.703	6968913.911
Hooded Crane	-5100247.186	7214295.674
Hooded Crane	-5099988.925	7209540.826
Hooded Crane	-4896893.193	6952110.569
Hooded Crane	-4888642.192	6969359.751
Hooded Crane	-4885015.403	6984965.148
Hooded Crane	-4895902.45	6954307.595
Hooded Crane	-4893827.454	6951163.635
Hooded Crane	-4888312.687	6969698.31
Hooded Crane	-4888488.571	6969700.16
Hooded Crane	-5101122.157	7213381.495
Hooded Crane	-4818684.572	6741731.587
Hooded Crane	-4893914.283	6950369.996
Hooded Crane	-4812141.212	6737095.91
Hooded Crane	-4889229.959	6969652.057
Hooded Crane	-4896218.597	6954494.093
Hooded Crane	-4888341.63	6969624.306
Hooded Crane	-4894635.634	6950632.073
Hooded Crane	-4888319.366	6969583.605
Hooded Crane	-4895902.45	6954285.437
Hooded Crane	-4883913.34	6976883.61
Hooded Crane	-4889937.951	6987467.986
Hooded Crane	-4893589.231	6950436.437
Hooded Crane	-5101070.95	7213736.466
Hooded Crane	-5101024.196	7214165.888

Hooded Crane	-5099746.248	7210462.185
Hooded Crane	-4888147.934	6969981.378
Hooded Crane	-4888842.567	6968902.812
Hooded Crane	-4888960.566	6969539.204
Hooded Crane	-4886638.442	6949622.572
Hooded Crane	-4888301.555	6969372.701
Hooded Crane	-4898298.045	6958635.117
Hooded Crane	-4888593.212	6969455.952
Hooded Crane	-4888619.928	6969285.751
Hooded Crane	-4888054.425	6969559.554
Hooded Crane	-5016468.137	7267212.486
Hooded Crane	-5739457.061	6505105.989
Hooded Crane	-4889087.47	6969320.901
Hooded Crane	-4888586.533	6969463.352
Hooded Crane	-4894633.407	6950633.919
Hooded Crane	-4888693.399	6968582.785
Hooded Crane	-4889232.186	6969642.807
Hooded Crane	-4888619.928	6969459.652
Hooded Crane	-4888504.156	6968965.709
Hooded Crane	-4898427.176	6951198.705
Hooded Crane	-4888731.248	6968588.335
Hooded Crane	-4888708.984	6968893.562
Hooded Crane	-5099797.455	7210548.032
Hooded Crane	-4888985.056	6969816.716
Hooded Crane	-5100140.319	7209525.567
Hooded Crane	-5099855.341	7209605.68
Hooded Crane	-4895279.06	6950818.485
Hooded Crane	-4883868.813	6977083.603
Hooded Crane	-5102840.93	7205524.745
Hooded Crane	-5100347.374	7209079.234
Hooded Crane	-5103054.664	7214978.992
Hooded Crane	-4888510.835	6969557.704
Hooded Crane	-4888392.837	6969568.804
Hooded Crane	-4895882.412	6952466.852
Hooded Crane	-5099142.897	7210345.817
Hooded Crane	-5101841.281	7212984.557
Hooded Crane	-5100287.261	7214387.288
Hooded Crane	-5102248.71	7212705.95
Hooded Crane	-4894005.565	6948472.968
Hooded Crane	-4889232.186	6969648.357
Hooded Crane	-4812076.647	6737031.22
Hooded Crane	-4888486.345	6969772.313
Hooded Crane	-4897187.076	6952125.337
Hooded Crane	-4888521.967	6968812.167
Hooded Crane	-4888045.52	6969554.004
Hooded Crane	-5102914.401	7205336.006
Hooded Crane	-5747037.918	6508325.143
Hooded Crane	-4888332.724	6969578.055
Hooded Crane	-4888368.346	6969598.405
Hooded Crane	-4888608.797	6969759.363
Hooded Crane	-4888844.794	6969572.505
Hooded Crane	-4889107.508	6969957.326
Hooded Crane	-4813644.025	6734733.263
Hooded Crane	-4888660.003	6969502.203
Hooded Crane	-4895911.355	6954300.209
Hooded Crane	-4888811.398	6969078.554
Hooded Crane	-4898197.857	6951076.885
Hooded Crane	-4888377.252	6969161.802

Hooded Crane	-4888352.762	6969650.207
Hooded Crane	-4888751.285	6969241.351
Hooded Crane	-4899155.205	6951420.2
Hooded Crane	-4898082.085	6958575.997
Hooded Crane	-4812116.722	6737013.25
Hooded Crane	-4891794.76	6952180.716
Hooded Crane	-4888236.989	6969283.901
Hooded Crane	-4888555.363	6969753.812
Hooded Crane	-5100006.736	7212114.418
Hooded Crane	-4894637.86	6950606.234
Hooded Crane	-4888757.965	6970638.21
Hooded Crane	-4891523.141	6970477.235
Hooded Crane	-4888515.288	6969559.554
Hooded Crane	-4888461.855	6969598.405
Hooded Crane	-4888611.023	6969470.752
Hooded Crane	-4885019.856	6984979.977
Hooded Crane	-4888312.687	6969626.156
Hooded Crane	-4812159.023	6735737.528
Hooded Crane	-4888746.833	6969629.857
Hooded Crane	-4888651.098	6969576.205
Hooded Crane	-4888680.041	6969535.503
Hooded Crane	-5101142.195	7213223.099
Hooded Crane	-4888339.403	6969659.458
Hooded Crane	-4897278.358	6952396.701
Hooded Crane	-4889005.094	6969763.063
Hooded Crane	-4888833.662	6969579.905
Hooded Crane	-4888744.606	6969433.751
Hooded Crane	-4897017.871	6952848.997
Hooded Crane	-4893867.529	6950322.011
Hooded Crane	-4888775.776	6970105.339
Hooded Crane	-4888357.214	6969611.356
Hooded Crane	-4888105.632	6969507.753
Hooded Crane	-4888829.209	6968945.36
Hooded Crane	-4888326.045	6969563.254
Hooded Crane	-4887219.529	6989318.741
Hooded Crane	-4888637.74	6969544.754
Hooded Crane	-4888871.511	6969526.253
Hooded Crane	-4888553.137	6968780.719
Hooded Crane	-4895906.902	6954303.902
Hooded Crane	-4888504.156	6969602.106
Hooded Crane	-4888167.971	6969317.201
Hooded Crane	-4897129.19	6952273.017
Hooded Crane	-4812081.099	6737025.829
Hooded Crane	-4888844.794	6969578.055
Hooded Crane	-4812138.986	6737043.798
Hooded Crane	-5099581.495	7209508.4
Hooded Crane	-4888769.097	6969592.855
Hooded Crane	-4902180.869	6961780.195
Hooded Crane	-4887822.881	6970120.141
Hooded Crane	-4889047.395	6969811.166
Hooded Crane	-4899215.318	7002995.862
Hooded Crane	-4888617.702	6969420.801
Hooded Crane	-4888608.797	6969467.052
Hooded Crane	-4888795.813	6969609.506
Hooded Crane	-4887998.766	6969480.002
Hooded Crane	-4888831.436	6969596.555
Hooded Crane	-4888775.776	6969642.807
Hooded Crane	-4894640.087	6950624.69

Hooded Crane	-4888833.662	6969579.905
Hooded Crane	-4897249.415	6952348.704
Hooded Crane	-4810213.158	6737534.378
Hooded Crane	-4888528.646	6969483.702
Hooded Crane	-5100302.846	7214274.679
Hooded Crane	-4896049.391	6947111.373
Hooded Crane	-4889245.544	6968871.364
Hooded Crane	-4888470.76	6968863.964
Hooded Crane	-4888893.774	6968814.017
Hooded Crane	-4889056.301	6969951.776
Hooded Crane	-5099541.42	7209189.861
Hooded Crane	-4888613.249	6969476.302
Hooded Crane	-4888680.041	6969546.604
Hooded Crane	-4888392.837	6970553.096
Hooded Crane	-4888590.985	6969454.102
Hooded Crane	-4888637.74	6969085.954
Hooded Crane	-4888611.023	6969467.052
Hooded Crane	-4889388.033	6970035.033
Hooded Crane	-4898289.139	6952215.79
Hooded Crane	-4889205.469	6970186.748
Hooded Crane	-4888459.628	6969631.707
Hooded Crane	-4888642.192	6969552.154
Hooded Crane	-4895270.155	6953951.229
Hooded Crane	-4898750.002	6953755.512
Hooded Crane	-4898177.82	6951117.491
Hooded Crane	-4888677.815	6969161.802
Hooded Crane	-4888363.894	6969574.355
Hooded Crane	-4898353.705	6951171.018
Hooded Crane	-4888822.53	6968758.521
Hooded Crane	-5741901.637	6511078.933
Hooded Crane	-4888261.48	6969400.451
Hooded Crane	-4888450.723	6970453.181
Hooded Crane	-4897004.513	6952596.077
Hooded Crane	-4888319.366	6970432.828
Hooded Crane	-4812105.59	6737059.971
Hooded Crane	-4898596.381	6951065.81
Hooded Crane	-4889091.923	6970005.43
Hooded Crane	-4888889.322	6969785.264
Hooded Crane	-4888326.045	6969642.807
Hooded Crane	-4812150.117	6737024.032
Hooded Crane	-5747075.766	6508920.903
Hooded Crane	-5100298.393	7214320.486
Hooded Crane	-4888506.383	6969541.054
Hooded Crane	-4888633.287	6969461.502
Hooded Crane	-4887813.975	6970195.999
Hooded Crane	-4812535.283	6736059.135
Hooded Crane	-4888285.97	6969670.558
Hooded Crane	-4889083.018	6969988.779
Hooded Crane	-5099383.347	7211648.858
Hooded Crane	-4888947.208	6969759.363
Hooded Crane	-4888944.981	6969777.864
Hooded Crane	-4888301.555	6969668.708
Hooded Crane	-4888611.023	6969448.552
Hooded Crane	-4888853.699	6968900.962
Hooded Crane	-4902412.413	6961541.776
Hooded Crane	-4888513.062	6969552.154
Hooded Crane	-4894640.087	6950615.462
Hooded Crane	-4897469.828	6951686.004

Hooded Crane	-4888326.045	6969642.807
Hooded Crane	-4889047.395	6969911.072
Hooded Crane	-4888740.154	6969750.112
Hooded Crane	-4888481.892	6969526.253
Hooded Crane	-4888566.495	6969740.862
Hooded Crane	-4889049.622	6969894.421
Hooded Crane	-4812094.458	6735762.681
Hooded Crane	-4889020.679	6970073.886
Hooded Crane	-4888136.802	6969507.753
Hooded Crane	-4888292.649	6969674.258
Hooded Crane	-4895909.129	6946849.413
Hooded Crane	-4889376.901	6969811.166
Hooded Crane	-4888624.381	6969010.107
Hooded Crane	-4895169.967	6951110.108
Hooded Crane	-4888285.97	6969681.659
Hooded Crane	-4888112.312	6969396.751
Hooded Crane	-4891124.617	6970046.134
Hooded Crane	-4888410.648	6969267.251
Hooded Crane	-4888599.891	6969450.402
Hooded Crane	-4888339.403	6969581.755
Hooded Crane	-4898157.782	6951115.645
Hooded Crane	-4888811.398	6968902.812
Hooded Crane	-4888642.192	6968562.437
Hooded Crane	-4888501.93	6968780.719
Hooded Crane	-4888811.398	6969084.104
Hooded Crane	-4888753.512	6969648.357
Hooded Crane	-4888441.817	6969483.702
Hooded Crane	-4889708.633	6987647.85
Hooded Crane	-4889051.848	6969931.424
Hooded Crane	-4888181.33	6969302.401
Hooded Crane	-4888156.839	6969468.902
Hooded Crane	-4888493.024	6969737.161
Hooded Crane	-4898053.142	6958158.475
Hooded Crane	-4888675.588	6969021.206
Hooded Crane	-4889080.791	6970007.28
Hooded Crane	-4892974.747	6945111.839
Hooded Crane	-4883879.945	6977165.084
Hooded Crane	-4899121.809	6951086.113
Hooded Crane	-4888760.191	6969646.507
Hooded Crane	-4891529.82	6970782.535
Hooded Crane	-4896637.158	6955515.295
Hooded Crane	-4888811.398	6968928.71
Hooded Crane	-4888281.517	6969690.909
Hooded Crane	-4812132.306	6737050.986
Hooded Crane	-4888446.27	6969591.005
Hooded Crane	-4888782.455	6969646.507
Hooded Crane	-4888639.966	6968697.475
Hooded Crane	-4898166.688	6951111.954
Hooded Crane	-4894631.181	6950626.536
Hooded Crane	-4888838.115	6969579.905
Hooded Crane	-4888488.571	6968814.017
Hooded Crane	-4888544.231	6968799.218
Hooded Crane	-4888755.738	6969635.407
Hooded Crane	-4888490.798	6968991.608
Hooded Crane	-4888644.419	6969492.952
Hooded Crane	-4888281.517	6969729.761
Hooded Crane	-4891543.178	6970717.773
Hooded Crane	-4888321.592	6969659.458

Hooded Crane	-4898876.906	6956564.335
Hooded Crane	-4888101.18	6969528.103
Hooded Crane	-4888833.662	6969600.255
Hooded Crane	-4888784.681	6969724.211
Hooded Crane	-4888844.794	6969574.355
Hooded Crane	-4888499.703	6968812.167
Hooded Crane	-4888435.138	6968956.459
Hooded Crane	-4888615.476	6969470.752
Hooded Crane	-4897180.397	6952276.709
Hooded Crane	-4897189.303	6952413.316
Hooded Crane	-4889194.337	6970092.388
Hooded Crane	-4888495.251	6969507.753
Hooded Crane	-4888613.249	6969476.302
Hooded Crane	-4888835.888	6969585.455
Hooded Crane	-4901003.109	6958110.443
Hooded Crane	-4887709.335	6970164.545
Hooded Crane	-4888597.665	6969452.252
Hooded Crane	-4888751.285	6969642.807
Hooded Crane	-5097136.919	7217844.62
Hooded Crane	-4888513.062	6969576.205
Hooded Crane	-4888372.799	6969603.956
Hooded Crane	-4889080.791	6969977.678
Hooded Crane	-4888526.42	6969565.104
Hooded Crane	-4888319.366	6969672.408
Hooded Crane	-4888586.533	6968986.058
Hooded Crane	-4888192.462	6969006.407
Hooded Crane	-4888308.234	6969668.708
Hooded Crane	-4888390.61	6970493.887
Hooded Crane	-4888635.513	6969546.604
Hooded Crane	-4888314.913	6969824.116
Hooded Crane	-4895085.365	6953823.827
Hooded Crane	-4888334.951	6969615.056
Hooded Crane	-4896501.348	6953081.616
Hooded Crane	-4888840.341	6969912.923
Hooded Crane	-4888112.312	6969481.852
Hooded Crane	-4888390.61	6969541.054
Hooded Crane	-4888352.762	6969591.005
Hooded Crane	-4897921.785	6952627.461
Hooded Crane	-4898538.495	6951230.083
Hooded Crane	-4888766.87	6968873.213
Hooded Crane	-4888176.877	6969246.901
Hooded Crane	-4888613.249	6969470.752
Hooded Crane	-4898082.085	6958539.047
Hooded Crane	-4888635.513	6969465.202
Hooded Crane	-4888110.085	6969457.802
Hooded Crane	-4888441.817	6969555.854
Hooded Crane	-4888902.68	6969985.078
Hooded Crane	-4888590.985	6969461.502
Hooded Crane	-4898847.963	6951075.039
Hooded Crane	-4888686.72	6969596.555
Hooded Crane	-4888506.383	6969579.905
Hooded Crane	-4888319.366	6969565.104
Hooded Crane	-4894637.86	6950608.08
Hooded Crane	-4888631.06	6969063.755
Hooded Crane	-4888350.535	6969676.108
Hooded Crane	-4888606.57	6969461.502
Hooded Crane	-4884810.576	6984746.419
Hooded Crane	-4888065.557	6969522.553

Hooded Crane	-4816184.336	6739759.435
Hooded Crane	-4887800.617	6970168.246
Hooded Crane	-4888419.553	6969598.405
Hooded Crane	-4888132.349	6969507.753
Hooded Crane	-4888395.063	6968780.719
Hooded Crane	-4888595.438	6968937.96
Hooded Crane	-4898175.594	6951027.05
Hooded Crane	-4888833.662	6969579.905
Hooded Crane	-4888677.815	6969409.701
Hooded Crane	-4888484.119	6968814.017
Hooded Crane	-4888586.533	6969209.901
Hooded Crane	-4812132.306	6736971.921
Hooded Crane	-4888617.702	6969476.302
Hooded Crane	-4888775.776	6969650.207
Hooded Crane	-4898217.895	6952160.41
Hooded Crane	-4888294.876	6969594.705
Hooded Crane	-4889203.243	6969726.061
Hooded Crane	-4888655.551	6969389.351
Hooded Crane	-4902392.376	6962184.969
Hooded Crane	-4888847.02	6968956.459
Hooded Crane	-4899063.923	6951527.259
Hooded Crane	-4888606.57	6969444.852
Hooded Crane	-4888611.023	6969443.002
Hooded Crane	-4895911.355	6954300.209
Hooded Crane	-4902216.491	6961824.553
Hooded Crane	-4888806.945	6968784.419
Hooded Crane	-4888595.438	6968950.909
Hooded Crane	-4888751.285	6969640.957
Hooded Crane	-4888611.023	6969463.352
Hooded Crane	-4898643.135	6951158.098
Hooded Crane	-4888664.456	6968876.913
Hooded Crane	-4888208.046	6969202.502
Hooded Crane	-4898752.229	6953735.202
Hooded Crane	-4898876.906	6956579.111
Hooded Crane	-4888635.513	6969537.354
Hooded Crane	-4888613.249	6968976.808
Hooded Crane	-4884953.064	6984881.733
Hooded Crane	-4889056.301	6969944.375
Hooded Crane	-4888230.31	6969801.915
Hooded Crane	-4888230.31	6969130.353
Hooded Crane	-4898320.309	6951076.885
Hooded Crane	-4812081.099	6737033.017
Hooded Crane	-4889606.219	6987551.427
Hooded Crane	-4888626.608	6969389.351
Hooded Crane	-4889080.791	6970788.086
Hooded Crane	-4898124.387	6951333.447
Hooded Crane	-4888742.38	6969644.657
Hooded Crane	-4888760.191	6969692.759
Hooded Crane	-4888746.833	6969624.306
Hooded Crane	-4888771.323	6969653.908
Hooded Crane	-4894640.087	6950602.543
Hooded Crane	-4888533.099	6969069.305
Hooded Crane	-4889232.186	6969676.108
Hooded Crane	-4888862.605	6969505.903
Hooded Crane	-4888815.851	6969600.255
Hooded Crane	-4887215.077	6989287.212
Hooded Crane	-4888613.249	6969463.352
Hooded Crane	-4888323.819	6969609.506

Hooded Crane	-4898810.115	6951069.502
Hooded Crane	-4888611.023	6969439.301
Hooded Crane	-4888622.155	6969672.408
Hooded Crane	-4812132.306	6737040.205
Hooded Crane	-4894637.86	6950639.455
Hooded Crane	-4888826.983	6969598.405
Hooded Crane	-4888786.908	6969689.059
Hooded Crane	-4898480.609	6952500.081
Hooded Crane	-4888833.662	6969570.655
Hooded Crane	-4888851.473	6969568.804
Hooded Crane	-4888608.797	6969459.652
Hooded Crane	-4888506.383	6968945.36
Hooded Crane	-4889201.016	6969764.913
Hooded Crane	-4888617.702	6969010.107
Hooded Crane	-4895544.001	6950822.176
Hooded Crane	-4888749.059	6970682.617
Hooded Crane	-4888829.209	6969596.555
Hooded Crane	-4810204.253	6737608.058
Hooded Crane	-4892386.98	6952616.385
Hooded Crane	-4889002.868	6968949.059
Hooded Crane	-5099637.155	7208913.296
Hooded Crane	-4899219.77	6956569.876
Hooded Crane	-4888143.481	6969282.051
Hooded Crane	-4888622.155	6969446.702
Hooded Crane	-4891534.273	6970664.114
Hooded Crane	-4812138.986	6737024.032
Hooded Crane	-4888775.776	6969642.807
Hooded Crane	-4898905.849	6951224.546
Hooded Crane	-4898378.195	6951043.661
Hooded Crane	-4888279.291	6969657.608
Hooded Crane	-4888624.381	6968641.98
Hooded Crane	-4888613.249	6969478.152
Hooded Crane	-4898467.251	6952450.237
Hooded Crane	-4899155.205	6956592.04
Hooded Crane	-4888631.06	6969459.652
Hooded Crane	-4888595.438	6969446.702
Hooded Crane	-4888145.707	6969293.151
Hooded Crane	-4891106.806	6969918.473
Hooded Crane	-4888196.914	6970464.283
Hooded Crane	-4888613.249	6969467.052
Hooded Crane	-4888791.36	6969716.81
Hooded Crane	-4888613.249	6969470.752
Hooded Crane	-4888671.135	6969017.507
Hooded Crane	-4812134.533	6737029.423
Hooded Crane	-4888686.72	6969422.651
Hooded Crane	-4818651.176	6741692.031
Hooded Crane	-4888568.722	6969443.002
Hooded Crane	-4888662.23	6969394.901
Hooded Crane	-4898872.454	6956566.182
Hooded Crane	-4888461.855	6968732.623
Hooded Crane	-4902363.433	6961554.713
Hooded Crane	-4898153.33	6951119.337
Hooded Crane	-4888835.888	6969568.804
Hooded Crane	-4888468.534	6968791.818
Hooded Crane	-4888611.023	6969446.702
Hooded Crane	-4888236.989	6969407.851
Hooded Crane	-4888624.381	6969015.657
Hooded Crane	-4888504.156	6969002.707

Hooded Crane	-4897238.283	6949580.128
Hooded Crane	-4889078.565	6969972.127
Hooded Crane	-4889040.716	6969898.122
Hooded Crane	-4889049.622	6969911.072
Hooded Crane	-4889060.754	6969949.925
Hooded Crane	-4889060.754	6970865.801
Hooded Crane	-4888829.209	6969598.405
Hooded Crane	-4898872.454	6956562.487
Hooded Crane	-4888145.707	6969529.953
Hooded Crane	-4888691.173	6969433.751
Hooded Crane	-4809834.672	6738156.182
Hooded Crane	-4888824.756	6969596.555
Hooded Crane	-4889176.526	6969535.503
Hooded Crane	-4888277.064	6969546.604
Hooded Crane	-4888842.567	6969581.755
Hooded Crane	-4888740.154	6969485.552
Hooded Crane	-4888611.023	6969468.902
Hooded Crane	-4898752.229	6953744.433
Hooded Crane	-4888838.115	6969576.205
Hooded Crane	-4888809.172	6968947.21
Hooded Crane	-4888504.156	6969566.954
Hooded Crane	-4898754.455	6953737.048
Hooded Crane	-4894631.181	6950639.455
Hooded Crane	-4897151.454	6952369.011
Hooded Crane	-4889205.469	6968769.62
Hooded Crane	-4886934.551	6950098.701
Hooded Crane	-4888584.306	6969455.952
Hooded Crane	-4889067.433	6969994.329
Hooded Crane	-4889078.565	6969998.029
Hooded Crane	-4888575.401	6969452.252
Hooded Crane	-4889089.697	6969996.179
Hooded Crane	-5747093.578	6508906.926
Hooded Crane	-4898164.462	6951126.72
Hooded Crane	-4888824.756	6969603.956
Hooded Crane	-4898079.859	6951135.949
Hooded Crane	-4888584.306	6968987.908
Hooded Crane	-4812132.306	6737040.205
Hooded Crane	-4891200.314	6987644.141
Hooded Crane	-4888452.949	6969322.751
Hooded Crane	-4888294.876	6969642.807
Hooded Crane	-4898571.891	6950423.518
Hooded Crane	-5747064.635	6508887.707
Hooded Crane	-4888880.416	6969518.853
Hooded Crane	-4898830.152	6956592.04
Hooded Crane	-4888746.833	6969637.257
Hooded Crane	-4888757.965	6969644.657
Hooded Crane	-4888515.288	6969491.102
Hooded Crane	-4898173.367	6951121.183
Hooded Crane	-4884955.291	6984848.368
Hooded Crane	-5747387.461	6508795.108
Hooded Crane	-4888435.138	6969289.451
Hooded Crane	-4889294.525	6970192.298
Hooded Crane	-4888864.831	6968610.533
Hooded Crane	-4888611.023	6969446.702
Hooded Crane	-4888375.026	6987948.25
Hooded Crane	-4898847.963	6951076.885
Hooded Crane	-4888613.249	6969439.301
Hooded Crane	-4888813.624	6968895.412

Hooded Crane	-4888780.229	6969648.357
Hooded Crane	-4888849.247	6969565.104
Hooded Crane	-4888590.985	6968949.059
Hooded Crane	-4898830.152	6951231.929
Hooded Crane	-4898518.458	6952453.929
Hooded Crane	-4888472.987	6969531.803
Hooded Crane	-4888733.474	6969609.506
Hooded Crane	-4888757.965	6969648.357
Hooded Crane	-4898088.764	6951089.805
Hooded Crane	-4888778.002	6970123.841
Hooded Crane	-5747040.144	6509411.871
Hooded Crane	-4888606.57	6968949.059
Hooded Crane	-4898131.066	6951060.273
Hooded Crane	-4888936.076	6969670.558
Hooded Crane	-4888631.06	6969468.902
Hooded Crane	-4888134.575	6969504.053
Hooded Crane	-4888753.512	6969628.007
Hooded Crane	-4889203.243	6969755.662
Hooded Crane	-4888833.662	6969578.055
Hooded Crane	-4884692.577	6984646.325
Hooded Crane	-4888508.609	6969583.605
Hooded Crane	-4898763.36	6953744.433
Hooded Crane	-4898151.103	6951117.491
Hooded Crane	-4888793.587	6969613.206
Hooded Crane	-4888615.476	6969450.402
Hooded Crane	-4888599.891	6988074.348
Hooded Crane	-5099715.079	7209453.084
Hooded Crane	-4888517.515	6969648.357
Hooded Crane	-4889209.922	6969777.864
Hooded Crane	-4888562.042	6969293.151
Hooded Crane	-4891520.914	6970701.121
Hooded Crane	-4888688.947	6969261.701
Hooded Crane	-5719622.154	6550547.9
Hooded Crane	-4889236.638	6969653.908
Hooded Crane	-4898754.455	6953737.048
Hooded Crane	-4899736.293	6953137.002
Hooded Crane	-4888809.172	6969596.555
Hooded Crane	-4898876.906	6956577.264
Hooded Crane	-4889069.659	6970001.73
Hooded Crane	-4888831.436	6969587.305
Hooded Crane	-4889083.018	6970005.43
Hooded Crane	-4889196.563	6969763.063
Hooded Crane	-5101787.848	7212513.22
Hooded Crane	-4899090.64	6952197.33
Hooded Crane	-4888847.02	6969583.605
Hooded Crane	-4899157.431	6951420.2
Hooded Crane	-4884690.35	6984651.885
Hooded Crane	-4889078.565	6969981.378
Hooded Crane	-4891380.652	6970290.36
Hooded Crane	-4898168.914	6951121.183
Hooded Crane	-4888590.985	6969448.552
Hooded Crane	-4899208.638	6951521.721
Hooded Crane	-4884690.35	6984638.91
Hooded Crane	-4888254.801	6968980.508
Hooded Crane	-4900125.911	6998677.438
Hooded Crane	-4891024.429	6970717.773
Hooded Crane	-5748525.146	6510900.677
Hooded Crane	-4887842.918	6970281.109

Hooded Crane	-4888735.701	6969622.456
Hooded Crane	-4888615.476	6969463.352
Hooded Crane	-4899135.168	6951104.571
Hooded Crane	-4888499.703	6968814.017
Hooded Crane	-4888613.249	6969454.102
Hooded Crane	-4885151.213	6984705.639
Hooded Crane	-4889775.425	6972125.997
Hooded Crane	-4884690.35	6984638.91
Hooded Crane	-4890875.261	6970965.721
Hooded Crane	-4895904.676	6954302.055
Hooded Crane	-4898872.454	6956580.958
Hooded Crane	-4888622.155	6968643.83
Hooded Crane	-4887638.091	6970158.995
Hooded Crane	-4898792.304	6951128.566
Hooded Crane	-4889701.954	6987455.006
Hooded Crane	-4899607.162	6953122.233
Hooded Crane	-4898819.02	6951076.885
Hooded Crane	-4901762.308	6961358.808
Hooded Crane	-4889980.253	6987351.169
Hooded Crane	-4889984.705	6987419.775
Hooded Crane	-4888740.154	6969722.361
Hooded Crane	-4898010.841	6950956.912
Hooded Crane	-4898814.567	6951062.119
Hooded Crane	-4888829.209	6969568.804
Hooded Crane	-5748607.523	6511000.29
Hooded Crane	-4888606.57	6969468.902
Hooded Crane	-4898807.888	6951063.964
Hooded Crane	-4885186.835	6984720.468
Hooded Crane	-4898204.537	6950984.598
Hooded Crane	-4888684.494	6969570.655
Hooded Crane	-4888584.306	6969457.802
Hooded Crane	-4898756.681	6953733.355
Hooded Crane	-4885077.742	6984683.396
Hooded Crane	-5103152.625	7215402.756
Hooded Crane	-4898870.227	6956575.417
Hooded Crane	-4888593.212	6969444.852
Hooded Crane	-4888749.059	6969670.558
Hooded Crane	-4888452.949	6969330.151
Hooded Crane	-4888784.681	6969666.858
Hooded Crane	-4812123.401	6737094.113
Hooded Crane	-4888441.817	6969328.301
Hooded Crane	-4884957.517	6984739.004
Hooded Crane	-4890071.535	6987694.207
Hooded Crane	-4897126.964	6952276.709
Hooded Crane	-4898750.002	6953744.433
Hooded Crane	-4898725.512	6951191.322
Hooded Crane	-4888829.209	6969574.355
Hooded Crane	-4883993.49	7098610.347
Hooded Crane	-4889986.932	6987419.775
Hooded Crane	-4887807.296	6970140.493
Hooded Crane	-4902514.827	6962072.222
Hooded Crane	-4899159.658	6951429.43
Hooded Crane	-4888579.853	6968950.909
Hooded Crane	-4899099.545	6951547.563
Hooded Crane	-4899164.111	6951436.813
Hooded Crane	-4888611.023	6969147.003
Hooded Crane	-4901751.176	6961345.871
Hooded Crane	-5747251.651	6509174.246

Hooded Crane	-4898752.229	6953746.28
Hooded Crane	-4888301.555	6969631.707
Hooded Crane	-4902396.829	6961554.713
Hooded Crane	-4889984.705	6987419.775
Hooded Crane	-4888973.924	6969798.215
Hooded Crane	-4899150.752	6951423.892
Hooded Crane	-4812096.684	6737034.814
Hooded Crane	-4902125.209	6961741.382
Hooded Crane	-5100282.808	7214402.558
Hooded Crane	-4884697.03	6984646.325
Hooded Crane	-4884688.124	6984637.057
Hooded Crane	-5734852.886	6511196.024
Hooded Crane	-4902363.433	6961543.624
Hooded Crane	-4887651.449	6970205.25
Hooded Crane	-4899157.431	6951429.43
Hooded Crane	-4812118.948	6737040.205
Hooded Crane	-4888450.723	6969326.451
Hooded Crane	-4902957.879	6961889.242
Hooded Crane	-4898830.152	6951302.069
Hooded Crane	-4888495.251	6969565.104
Hooded Crane	-4888321.592	6968987.908
Hooded Crane	-5738388.393	6505741.685
Hooded Crane	-4889984.705	6987412.358
Hooded Crane	-5747810.475	6509768.321
Hooded Crane	-5720546.105	6552674.786
Hooded Crane	-4898229.027	6966731.325
Hooded Crane	-4888562.042	6969450.402
Hooded Crane	-4900112.553	6998675.581
Hooded Crane	-4897160.36	6952444.699
Hooded Crane	-4818746.91	6741857.447
Hooded Crane	-4818241.52	6743008.26
Hooded Crane	-5747244.972	6510082.848
Hooded Crane	-4899763.01	7003395.319
Hooded Crane	-4888524.194	6969517.003
Hooded Crane	-5747763.721	6545284.879
Hooded Crane	-4899776.368	6998408.195
Hooded Crane	-5747592.289	6508506.836
Hooded Crane	-5103413.112	7215477.204
Hooded Crane	-5739514.947	6503443.633
Hooded Crane	-4888450.723	6969278.351
Hooded Crane	-5739376.91	6504908.654
Hooded Crane	-4902245.434	6961798.677
Hooded Crane	-4889982.479	6987414.213
Hooded Crane	-4899270.977	7003036.735
Hooded Crane	-4900117.005	6998670.011
Hooded Crane	-5747035.691	6508889.454
Hooded Crane	-4888713.437	6969492.952
Hooded Crane	-4899157.431	6951427.584
Hooded Crane	-4889628.483	6987384.545
Hooded Crane	-4888441.817	6969500.353
Hooded Crane	-4889120.866	6968617.932
Hooded Crane	-4887800.617	6970168.246
Hooded Crane	-5747180.407	6508540.031
Hooded Crane	-4888608.797	6969470.752
Hooded Crane	-4903149.348	6962092.553
Hooded Crane	-4897438.659	6995853.63
Hooded Crane	-5748752.238	6511096.409
Hooded Crane	-5746349.963	6510643.786

Hooded Crane	-5747017.88	6508898.19
Hooded Crane	-5746298.756	6509939.562
Hooded Crane	-4902227.623	6961820.856
Hooded Crane	-5101131.063	7214289.948
Hooded Crane	-4898843.511	6951087.959
Hooded Crane	-5747037.918	6509429.343
Hooded Crane	-5750021.28	6511858.41
Hooded Crane	-5752815.399	6513298.714
Hooded Crane	-4888321.592	6969291.301
Hooded Crane	-4810598.324	6737298.968
Hooded Crane	-5747029.012	6508903.432
Hooded Crane	-4901782.345	7002707.893
Hooded Crane	-4898068.727	6951123.028
Hooded Crane	-5747380.782	6508478.883
Hooded Crane	-5742008.503	6511042.233
Hooded Crane	-4899462.447	7003070.178
Hooded Crane	-5719595.437	6550509.266
Hooded Crane	-5746868.712	6509649.502
Hooded Crane	-5751281.417	6510563.4
Hooded Crane	-5746817.505	6509574.368
Hooded Crane	-4889982.479	6987403.087
Hooded Crane	-5747728.099	6509750.847
Hooded Crane	-5746358.869	6509916.846
Hooded Crane	-5746470.188	6510432.338
Hooded Crane	-4812272.569	6736262.167
Hooded Crane	-5746118.419	6509745.605
Hooded Crane	-4888749.059	6969653.908
Hooded Crane	-4902149.699	6961785.739
Hooded Crane	-5746993.39	6510096.828
Hooded Crane	-4813908.965	6737099.504
Hooded Crane	-4888501.93	6969600.255
Hooded Crane	-5747819.381	6509846.952
Hooded Crane	-5747037.918	6508875.477
Hooded Crane	-5748560.768	6511106.895
Hooded Crane	-5746877.618	6509642.513
Hooded Crane	-5747719.193	6509881.899
Hooded Crane	-5747035.691	6508903.432
Hooded Crane	-5747385.235	6508475.389
Hooded Crane	-5016672.965	7268282.924
Hooded Crane	-5747901.757	6509944.804
Hooded Crane	-5747057.955	6509908.109
Hooded Crane	-4812096.684	6737104.895
Hooded Crane	-5747750.363	6510400.883
Hooded Crane	-5747325.122	6509037.964
Hooded Crane	-5747944.058	6510173.714
Hooded Crane	-5747051.276	6510102.07
Hooded Crane	-5726147.702	6554758.301
Hooded Crane	-5747387.461	6508520.813
Hooded Crane	-5747425.31	6510642.039
Hooded Crane	-5747763.721	6510669.999
Hooded Crane	-5747040.144	6508891.202
Hooded Crane	-5747378.555	6508484.124
Hooded Crane	-5099875.379	7210450.739
Hooded Crane	-5747725.872	6509770.068
Hooded Crane	-4884692.577	6984646.325
Hooded Crane	-5751297.002	6510542.43
Hooded Crane	-4900965.26	7001634.143
Hooded Crane	-5747213.803	6509909.857

Hooded Crane	-4812096.684	6736982.703
Hooded Crane	-4812366.077	6736308.883
Hooded Crane	-5103499.942	7211820.578
Hooded Crane	-5739359.099	6504892.937
Hooded Crane	-5100358.506	7207953.979
Hooded Crane	-5725851.592	6554361.232
Hooded Crane	-5747169.275	6508520.813
Hooded Crane	-5748518.467	6510465.54
Hooded Crane	-5747374.103	6508480.63
Hooded Crane	-5725994.081	6554742.488
Hooded Crane	-5747656.854	6510404.378
Hooded Crane	-5747298.405	6510374.671
Hooded Crane	-5747269.462	6508569.731
Hooded Crane	-5747180.407	6508541.778
Hooded Crane	-5741830.392	6510556.41
Hooded Crane	-5747022.333	6508875.477
Hooded Crane	-5747033.465	6509397.892
Hooded Crane	-5748436.091	6511056.214
Hooded Crane	-5739359.099	6504915.639
Hooded Crane	-5740450.03	6504484.312
Hooded Crane	-5747478.743	6510491.753
Hooded Crane	-5746663.884	6508494.607
Hooded Crane	-5747024.559	6508882.466
Hooded Crane	-5746726.223	6508973.318
Hooded Crane	-5739136.46	6503967.449
Hooded Crane	-5747044.597	6508892.949
Hooded Crane	-5734988.696	6511045.728
Hooded Crane	-5747024.559	6508891.202
Hooded Crane	-5751303.681	6510538.935
Hooded Crane	-5747031.239	6508880.719
Hooded Crane	-4897587.827	6996072.666
Hooded Crane	-5747135.879	6509866.173
Hooded Crane	-4900103.647	6998696.007
Hooded Crane	-5747509.912	6508784.626
Hooded Crane	-5747040.144	6508891.202
Hooded Crane	-5747031.239	6508884.213
Hooded Crane	-5741837.071	6510558.158
Hooded Crane	-5751470.66	6510556.41
Hooded Crane	-5746056.08	6509646.008
Hooded Crane	-5739147.592	6503920.304
Hooded Crane	-5749707.359	6504582.101
Hooded Crane	-4812651.055	6735784.241
Hooded Crane	-4899366.712	7003226.244
Hooded Crane	-4887778.353	6970155.294
Hooded Crane	-5747779.306	6509883.646
Hooded Crane	-5747142.558	6509632.029
Hooded Crane	-4884156.017	7098482.468
Hooded Crane	-5747387.461	6508470.148
Hooded Crane	-5747031.239	6508878.971
Hooded Crane	-5747819.381	6509845.204
Hooded Crane	-5739100.838	6504000.625
Hooded Crane	-5747184.86	6508538.284
Hooded Crane	-5738947.217	6490238.836
Hooded Crane	-5745063.11	6510180.704
Hooded Crane	-5747033.465	6508887.707
Hooded Crane	-5751294.775	6510551.168
Hooded Crane	-5734950.848	6511229.23
Hooded Crane	-4900103.647	6998690.436

Hooded Crane	-5725987.402	6554747.759
Hooded Crane	-5747485.422	6510362.439
Hooded Crane	-4889218.827	6970129.392
Hooded Crane	-5748050.925	6510636.796
Hooded Crane	-5738566.505	6489044.841
Hooded Crane	-5747055.729	6508891.202
Hooded Crane	-5745989.288	6509780.552
Hooded Crane	-5747443.121	6509518.454
Hooded Crane	-4814213.981	6734514.102
Hooded Crane	-5747035.691	6508898.19
Hooded Crane	-5725882.762	6554348.934
Hooded Crane	-5747026.786	6508877.224
Hooded Crane	-5751292.549	6510552.915
Hooded Crane	-4899219.77	7002999.577
Hooded Crane	-5747158.143	6508484.124
Hooded Crane	-5725807.065	6554540.438
Hooded Crane	-4900103.647	6998688.579
Hooded Crane	-5725994.081	6554746.002
Hooded Crane	-5747189.312	6508531.295
Hooded Crane	-5747031.239	6508891.202
Hooded Crane	-4812713.394	6736835.356
Hooded Crane	-5748024.209	6510565.148
Hooded Crane	-5747040.144	6508887.707
Hooded Crane	-5719602.116	6550532.095
Hooded Crane	-5747022.333	6509443.321
Hooded Crane	-5747182.633	6508543.525
Hooded Crane	-5747040.144	6508887.707
Hooded Crane	-5748640.919	6509979.752
Hooded Crane	-5739385.816	6504934.849
Hooded Crane	-5747033.465	6508894.696
Hooded Crane	-5099369.988	7210084.472
Hooded Crane	-5747487.649	6510098.575
Hooded Crane	-5727501.347	6500088.518
Hooded Crane	-5738775.785	6489412.608
Hooded Crane	-5747035.691	6508882.466
Hooded Crane	-5735075.525	6511229.23
Hooded Crane	-5751455.075	6510573.885
Hooded Crane	-5747184.86	6508541.778
Hooded Crane	-5747031.239	6508875.477
Hooded Crane	-5747383.008	6508482.377
Hooded Crane	-5747216.029	6508538.284
Hooded Crane	-5744087.951	6505097.257
Hooded Crane	-5735358.277	6510893.687
Hooded Crane	-5739430.344	6503424.427
Hooded Crane	-5747037.918	6508884.213
Hooded Crane	-5747249.425	6508377.554
Hooded Crane	-5747182.633	6508545.272
Hooded Crane	-5725978.497	6554067.836
Hooded Crane	-5747102.483	6509499.234
Hooded Crane	-5747581.157	6508964.582
Hooded Crane	-5748718.842	6509499.234
Hooded Crane	-5747035.691	6508884.213
Hooded Crane	-5740572.482	6504529.714
Hooded Crane	-5747389.687	6508457.918
Hooded Crane	-5749246.497	6511302.632
Hooded Crane	-5747031.239	6508889.454
Hooded Crane	-5718395.413	6550477.657
Hooded Crane	-5739374.684	6504945.327

Hooded Crane	-5745238.995	6510315.257
Hooded Crane	-5747193.765	6508543.525
Hooded Crane	-5747287.273	6508625.638
Hooded Crane	-5739152.045	6503943.003
Hooded Crane	-5747040.144	6508885.96
Hooded Crane	-5725867.177	6554322.581
Hooded Crane	-5747104.71	6510683.98
Hooded Crane	-5744203.724	6508938.375
Hooded Crane	-5746859.807	6509635.524
Hooded Crane	-5747031.239	6508889.454
Hooded Crane	-5739432.57	6510682.232
Hooded Crane	-5747730.325	6510378.166
Hooded Crane	-5747020.107	6509885.394
Hooded Crane	-5718675.938	6550653.265
Hooded Crane	-5747679.118	6509700.175
Hooded Crane	-5727761.835	6500395.693
Hooded Crane	-5739198.799	6503340.62
Hooded Crane	-4883626.136	7090526.075
Hooded Crane	-4888165.745	6969417.101
Hooded Crane	-5739372.458	6504926.117
Hooded Crane	-4901989.399	7006377.953
Hooded Crane	-5747721.42	6510337.974
Hooded Crane	-5747801.57	6510589.613
Hooded Crane	-4898932.566	6956641.91
Hooded Crane	-5738375.035	6505743.431
Hooded Crane	-5746973.353	6508894.696
Hooded Crane	-5747035.691	6508894.696
Hooded Crane	-5747917.342	6509960.531
Hooded Crane	-5734265.119	6503368.556
Hooded Crane	-5746309.888	6509953.541
Hooded Crane	-5720848.894	6482636.838
Hooded Crane	-5751508.509	6510376.419
Hooded Crane	-5747198.218	6508636.121
Hooded Crane	-5747035.691	6508903.432
Hooded Crane	-5738363.903	6505753.91
Hooded Crane	-5747843.871	6509728.132
Hooded Crane	-5721514.585	6483673.139
Hooded Crane	-5739356.873	6504919.132
Hooded Crane	-5721505.679	6483685.331
Hooded Crane	-5739336.835	6504891.191
Hooded Crane	-5721512.359	6483687.073
Hooded Crane	-5734855.113	6511241.463
Hooded Crane	-5744767	6506531.134
Hooded Crane	-4880787.489	7101416.696
Hooded Crane	-5718395.413	6550470.633
Hooded Crane	-5747040.144	6508899.937
Hooded Crane	-4888642.192	6969047.105
Hooded Crane	-5747051.276	6509452.058
Hooded Crane	-5747247.198	6508653.592
Hooded Crane	-5747318.443	6508560.996
Hooded Crane	-5721507.906	6483706.233
Hooded Crane	-5747610.1	6509099.116
Hooded Crane	-5747730.325	6509792.784
Hooded Crane	-5721521.264	6483685.331
Hooded Crane	-5742391.442	6547930.009
Hooded Crane	-5747022.333	6508898.19
Hooded Crane	-5739336.835	6504906.908
Hooded Crane	-5741616.659	6504386.525

Hooded Crane	-4812167.929	6737024.032
Hooded Crane	-5721859.675	6478811.495
Hooded Crane	-5734986.47	6511028.252
Hooded Crane	-5747846.097	6510958.348
Hooded Crane	-5734213.912	6503578.076
Hooded Crane	-5747652.401	6510224.39
Hooded Crane	-5720748.707	6481257.629
Hooded Crane	-4887825.107	6970244.104
Hooded Crane	-4901666.573	7005998.79
Hooded Crane	-5477800.597	6840510.607
Hooded Crane	-4881780.459	7054721.622
Hooded Crane	-5744295.006	6506272.633
Hooded Crane	-5747026.786	6508892.949
Hooded Crane	-5713789.012	6493490.522
Hooded Crane	-5721623.678	6480098.018
Hooded Crane	-5720717.537	6478684.421
Hooded Crane	-4887704.882	7037686.417
Hooded Crane	-5720579.501	6478687.902
Hooded Crane	-5722405.141	6478277.099
Hooded Crane	-4887393.188	7034462.488
Hooded Crane	-5721505.679	6483690.557
Hooded Crane	-5722365.066	6480205.963
Hooded Crane	-5714189.762	6500179.273
Hooded Crane	-5099895.416	7210397.324
Hooded Crane	-4898814.567	7022732.769
Hooded Crane	-4812637.697	6736749.105
Hooded Crane	-5099786.323	7209517.937
Hooded Crane	-4899927.762	7018494.754
Hooded Crane	-4881845.024	7094462.852
Hooded Crane	-5720715.311	6481628.53
Hooded Crane	-4884204.997	7098574.616
Hooded Crane	-4883098.482	7090343.847
Hooded Crane	-4893019.275	6950229.734
Hooded Crane	-5710447.201	6498941.949
Hooded Crane	-4811978.685	6736817.387
Hooded Crane	-4897171.492	6952385.625
Hooded Crane	-4889047.395	6969948.075
Hooded Crane	-5722247.067	6477977.713
Hooded Crane	-5712312.916	6498535.365
Hooded Crane	-4885391.663	7098104.484
Hooded Crane	-4882613.129	7096921.755
Hooded Crane	-5101761.131	7212358.658
Hooded Crane	-4812130.08	6736971.921
Hooded Crane	-4882719.995	7053646.375
Hooded Crane	-5710460.56	6498997.79
Hooded Crane	-5710438.296	6498931.479
Hooded Crane	-5710460.56	6498940.204
Hooded Crane	-4882606.45	7097122.938
Hooded Crane	-4812114.495	6736975.515
Hooded Crane	-4880498.058	7100927.582
Hooded Crane	-5710444.975	6498929.734
Hooded Crane	-5710427.164	6498945.439
Hooded Crane	-4881996.419	7096906.714
Hooded Crane	-5675421.637	6527838.297
Hooded Crane	-4882103.285	7096807.064
Hooded Crane	-5710458.333	6498940.204
Hooded Crane	-5712039.07	6497642
Hooded Crane	-4883833.19	7098584.019

Hooded Crane	-4884095.904	7098593.422
Hooded Crane	-4812123.401	6736989.89
Hooded Crane	-4883842.096	7092666.159
Hooded Crane	-5744419.683	6506345.991
Hooded Crane	-4883203.122	7090388.934
Hooded Crane	-4882098.833	7095953.517
Hooded Crane	-5705947.667	6501024.042
Hooded Crane	-4884454.353	7098978.953
Hooded Crane	-4883147.462	7090291.246
Hooded Crane	-4882644.298	7094186.555
Hooded Crane	-4883252.103	7090417.113
Hooded Crane	-4812130.08	6737203.728
Hooded Crane	-4882791.24	7090159.745
Hooded Crane	-4881580.084	7094101.977
Hooded Crane	-4882746.712	7097184.986
Hooded Crane	-4880691.754	7101313.227
Hooded Crane	-4894562.163	7063643.421
Hooded Crane	-4882608.676	7097134.22
Hooded Crane	-4883855.454	7090604.979
Hooded Crane	-5721552.434	6483662.688
Hooded Crane	-4882898.107	7090189.802
Hooded Crane	-4883822.058	7093402.829
Hooded Crane	-4882606.45	7097134.22
Hooded Crane	-4884367.524	7092474.486
Hooded Crane	-4811996.497	6736964.733
Hooded Crane	-4881575.631	7094126.411
Hooded Crane	-4882802.372	7096996.963
Hooded Crane	-4883096.255	7097248.915
Hooded Crane	-4881522.198	7094103.856
Hooded Crane	-4883989.038	7098862.351
Hooded Crane	-4881575.631	7094111.374
Hooded Crane	-5710578.558	6498575.499
Hooded Crane	-4883073.991	7090388.934
Hooded Crane	-4881339.634	7101053.62
Hooded Crane	-4883955.642	7098694.975
Hooded Crane	-4882608.676	7097115.417
Hooded Crane	-5710444.975	6498957.654
Hooded Crane	-4881043.524	7101128.867
Hooded Crane	-4882546.337	7097141.74
Hooded Crane	-4882584.186	7097224.472
Hooded Crane	-4884285.148	7098832.261
Hooded Crane	-4880653.906	7101143.917
Hooded Crane	-4883761.946	7090552.376
Hooded Crane	-4883232.065	7090435.9
Hooded Crane	-4880903.261	7101174.016
Hooded Crane	-4882546.337	7097143.621
Hooded Crane	-4885006.498	7098253.043
Hooded Crane	-4882829.089	7096812.705
Hooded Crane	-4883073.991	7090381.42
Hooded Crane	-4881230.541	7096008.036
Hooded Crane	-5739305.666	6504864.996
Hooded Crane	-4881551.141	7094090.7
Hooded Crane	-4884249.525	7090914.967
Hooded Crane	-4881515.519	7100982.135
Hooded Crane	-4881114.768	7096053.155
Hooded Crane	-4887731.599	6969531.803
Hooded Crane	-4884053.603	7098595.303
Hooded Crane	-4883991.264	7099037.254

Hooded Crane	-4881573.405	7094126.411
Hooded Crane	-4883078.444	7090349.483
Hooded Crane	-4881239.446	7100942.631
Hooded Crane	-4881531.103	7094120.772
Hooded Crane	-4883071.765	7090388.934
Hooded Crane	-5730720.707	6502935.565
Hooded Crane	-4881577.857	7094120.772
Hooded Crane	-4882319.245	7096500.604
Hooded Crane	-4880631.642	7101296.296
Hooded Crane	-4880491.379	7100918.176
Hooded Crane	-4883094.029	7090390.813
Hooded Crane	-4884347.486	7099086.152
Hooded Crane	-4881085.825	7095968.557
Hooded Crane	-4882875.843	7090067.696
Hooded Crane	-4880785.263	7101316.989
Hooded Crane	-4883067.312	7090396.449
Hooded Crane	-4881424.237	7095611.372
Hooded Crane	-4884449.9	7090958.178
Hooded Crane	-4884389.788	7094571.87
Hooded Crane	-4883082.897	7090392.691
Hooded Crane	-4884990.913	7098303.816
Hooded Crane	-4883942.283	7098950.742
Hooded Crane	-4881143.711	7096041.876
Hooded Crane	-4883080.671	7090383.298
Hooded Crane	-4881551.141	7095506.1
Hooded Crane	-4884178.281	7098552.049
Hooded Crane	-4883067.312	7090396.449
Hooded Crane	-4879834.594	7058900.585
Hooded Crane	-4881074.693	7101128.867
Hooded Crane	-4880493.606	7100912.533
Hooded Crane	-4880740.735	7101258.671
Hooded Crane	-4882060.984	7095838.84
Hooded Crane	-4883100.708	7090253.674
Hooded Crane	-4881515.519	7100967.086
Hooded Crane	-4881426.463	7095615.132
Hooded Crane	-4884017.981	7098576.497
Hooded Crane	-4881297.332	7101409.171
Hooded Crane	-4894709.105	7063579.765
Hooded Crane	-4881517.745	7100965.205
Hooded Crane	-4880789.715	7101463.728
Hooded Crane	-4880725.15	7101134.511
Hooded Crane	-4894555.484	7063656.527
Hooded Crane	-4881301.785	7096205.435
Hooded Crane	-4881575.631	7094118.892
Hooded Crane	-4883632.815	7090578.678
Hooded Crane	-4883989.038	7098657.362
Hooded Crane	-4881074.693	7101495.71
Hooded Crane	-4884015.754	7098555.81
Hooded Crane	-4882067.663	7095835.081
Hooded Crane	-4881577.857	7094115.133
Hooded Crane	-4880722.924	7101204.116
Hooded Crane	-4880495.832	7100938.869
Hooded Crane	-4883254.329	7090434.021
Hooded Crane	-4881295.106	7096203.555
Hooded Crane	-4881332.955	7096235.516
Hooded Crane	-4881043.524	7101140.155
Hooded Crane	-4884253.978	7098854.828
Hooded Crane	-4881524.424	7100963.324

Hooded Crane	-4881526.651	7100974.611
Hooded Crane	-4881566.726	7094120.772
Hooded Crane	-4881321.823	7096226.116
Hooded Crane	-4882363.773	7096244.916
Hooded Crane	-4884020.207	7098582.138
Hooded Crane	-4880811.979	7100910.651
Hooded Crane	-4894551.031	7063649.038
Hooded Crane	-4882165.624	7096765.701
Hooded Crane	-4881586.763	7094111.374
Hooded Crane	-4880785.263	7101469.372
Hooded Crane	-4883087.35	7090390.813
Hooded Crane	-4880629.415	7101285.008
Hooded Crane	-4883802.021	7090527.954
Hooded Crane	-4881330.728	7096231.756
Hooded Crane	-4884204.997	7092427.508
Hooded Crane	-4880845.375	7101379.071
Hooded Crane	-4881522.198	7100995.303
Hooded Crane	-5477798.371	6840514.247
Hooded Crane	-4884022.434	7098580.258
Hooded Crane	-4884456.58	7092769.514
Hooded Crane	-4881330.728	7096233.636
Hooded Crane	-4882063.21	7095852
Hooded Crane	-4884009.075	7098572.736
Hooded Crane	-4880685.075	7101273.721
Hooded Crane	-4881524.424	7100965.205
Hooded Crane	-4882063.21	7095836.961
Hooded Crane	-4884387.561	7090020.732
Hooded Crane	-4883643.947	7090569.284
Hooded Crane	-4894548.805	7063662.144
Hooded Crane	-4882123.323	7095964.797
Hooded Crane	-4882822.409	7096820.226
Hooded Crane	-4881330.728	7096235.516
Hooded Crane	-4884229.488	7098544.527
Hooded Crane	-4877049.381	7108418.133
Hooded Crane	-4884017.981	7098574.616
Hooded Crane	-4884425.41	7092827.769
Hooded Crane	-4881232.767	7100982.135
Hooded Crane	-4881299.559	7096203.555
Hooded Crane	-4880500.285	7100920.057
Hooded Crane	-4880498.058	7100925.701
Hooded Crane	-4880983.411	7101000.947
Hooded Crane	-4883087.35	7090396.449
Hooded Crane	-4883672.89	7098999.64
Hooded Crane	-4881528.877	7100965.205
Hooded Crane	-4881330.728	7096237.396
Hooded Crane	-4881092.505	7101497.591
Hooded Crane	-4884022.434	7098572.736
Hooded Crane	-4880814.206	7101183.422
Hooded Crane	-4881003.449	7101497.591
Hooded Crane	-5099561.458	7209334.823
Hooded Crane	-4883076.218	7090387.055
Hooded Crane	-4880665.038	7101298.177
Hooded Crane	-4880653.906	7101277.483
Hooded Crane	-4881034.618	7101441.153
Hooded Crane	-4894548.805	7063665.888
Hooded Crane	-4876922.476	7098234.237
Hooded Crane	-4880838.696	7101207.878
Hooded Crane	-4884017.981	7098576.497

Hooded Crane	-4880716.245	7101269.959
Hooded Crane	-5477798.371	6840496.044
Hooded Crane	-4884107.036	7098646.079
Hooded Crane	-5477782.786	6840494.224
Hooded Crane	-4894551.031	7063643.421
Hooded Crane	-4880482.474	7101121.343
Hooded Crane	-4876437.123	7105739.157
Hooded Crane	-4880498.058	7100920.057
Hooded Crane	-4881128.127	7101755.331
Hooded Crane	-4880578.208	7101110.055
Hooded Crane	-4894539.899	7063654.655
Hooded Crane	-4884093.678	7098715.661
Hooded Crane	-4880638.321	7101281.246
Hooded Crane	-5477785.013	6840505.146
Hooded Crane	-4890968.77	7062776.62
Hooded Crane	-4882054.305	7095844.48
Hooded Crane	-5477802.824	6840494.224
Hooded Crane	-4881043.524	7101121.343
Hooded Crane	-4880624.963	7101286.89
Hooded Crane	-4880531.454	7101145.798
Hooded Crane	-4880620.51	7101277.483
Hooded Crane	-5477789.465	6840514.247
Hooded Crane	-4812096.684	6737052.783
Hooded Crane	-5477800.597	6840503.326
Hooded Crane	-4882755.618	7096855.949
Hooded Crane	-4812045.477	6737070.753
Hooded Crane	-4874255.261	7109958.535
Hooded Crane	-4884026.886	7098584.019
Hooded Crane	-5477805.05	6840525.169
Hooded Crane	-5477800.597	6840557.934
Hooded Crane	-4880918.846	7101426.102
Hooded Crane	-5477780.56	6840494.224
Hooded Crane	-4876065.316	7111444.612
Hooded Crane	-5477809.503	6840525.169
Hooded Crane	-5477791.692	6840499.685
Hooded Crane	-4884026.886	7098535.124
Hooded Crane	-4889428.108	7064047.836
Hooded Crane	-5101928.11	7212225.089
Hooded Crane	-4812185.74	6735978.283
Hooded Crane	-4878926.227	7114560.828
Hooded Crane	-4882737.807	7097177.465
Hooded Crane	-4878037.898	7114564.597
Hooded Crane	-4953018.254	6820218.462
Hooded Crane	-4877519.149	7115045.139
Hooded Crane	-4947650.428	7122956.656
Hooded Crane	-5748122.17	6510631.554
Hooded Crane	-4947338.733	7127047.53
Hooded Crane	-4947345.413	7127138.127
Hooded Crane	-4944310.843	7123988.608
Hooded Crane	-4948932.829	7125662.281
Hooded Crane	-4950328.775	7125475.461
Hooded Crane	-4812110.042	6735791.428
Hooded Crane	-4950310.964	7125503.767
Hooded Crane	-4950322.096	7125484.896
Hooded Crane	-4950328.775	7125479.235
Hooded Crane	-4950317.643	7125481.122
Hooded Crane	-4950331.001	7125479.235
Hooded Crane	-4950346.586	7125496.218

Hooded Crane	-4818510.913	6741499.65
Hooded Crane	-4950368.85	7125530.185
Hooded Crane	-4949331.352	7125483.009
Hooded Crane	-4949959.194	7125290.532
Hooded Crane	-4953875.414	7124481.049
Hooded Crane	-4953884.32	7124492.37
Hooded Crane	-5100605.635	7209235.638
Hooded Crane	-4812390.568	6735250.65
Hooded Crane	-4814352.017	6734636.257
Hooded Crane	-5113338.358	7185500.033
Hooded Crane	-4879404.901	7143905.29
Hooded Crane	-4879395.995	7143924.205
Hooded Crane	-4879701.011	7143772.887
Hooded Crane	-4879398.222	7143950.686
Hooded Crane	-4879554.069	7143977.168
Hooded Crane	-4879393.769	7143937.446
Hooded Crane	-4879567.427	7143969.601
Hooded Crane	-4879567.427	7143969.601
Hooded Crane	-4879384.864	7143926.097
Hooded Crane	-4879551.843	7143948.795
Hooded Crane	-4879393.769	7143935.554
Hooded Crane	-4879393.769	7143935.554
Hooded Crane	-4879549.616	7143982.842
Hooded Crane	-4879549.616	7143982.842
Hooded Crane	-4879574.107	7143971.493
Hooded Crane	-4879574.107	7143971.493
Hooded Crane	-4879562.975	7143931.771
Hooded Crane	-4879540.711	7143975.276
Hooded Crane	-4879540.711	7143975.276
Hooded Crane	-4879551.843	7143963.927
Hooded Crane	-4879551.843	7143965.818
Hooded Crane	-4879551.843	7143965.818
Hooded Crane	-4879545.164	7143980.951
Hooded Crane	-4812432.869	6736431.065
White-naped Crane	-6171778.548	5943904.216
White-naped Crane	-6174405.688	5936263.253
White-naped Crane	-6174138.521	5934884.356
White-naped Crane	-6172977.459	5946070.388
White-naped Crane	-6177902.233	5933611.724
White-naped Crane	-6226232.703	5897547.106
White-naped Crane	-6174296.595	5934938.202
White-naped Crane	-5870166.18	6250270.512
White-naped Crane	-5855345.103	6304667.488
White-naped Crane	-5278009.941	5876327.459
White-naped Crane	-6177537.105	5933293.596
White-naped Crane	-5332655.566	5587847.904
White-naped Crane	-5859255.757	6301553.951
White-naped Crane	-6229213.839	5906856.54
White-naped Crane	-5858908.44	6301678.334
White-naped Crane	-6178868.487	5932326.231
White-naped Crane	-6227924.76	5907142.793
White-naped Crane	-6174404.575	5934877.829
White-naped Crane	-6228165.21	5907074.482
White-naped Crane	-6225794.105	5905275.814
White-naped Crane	-5858060.186	6303286.959
White-naped Crane	-5870264.141	6250172.294
White-naped Crane	-6171430.118	5937151.085
White-naped Crane	-6173128.854	5946161.883

White-naped Crane	-5844442.472	6332310.856
White-naped Crane	-6177165.298	5933260.968
White-naped Crane	-6170893.558	5936504.786
White-naped Crane	-6173064.288	5946220.701
White-naped Crane	-6226404.135	5905083.935
White-naped Crane	-5881706.672	6323960.876
White-naped Crane	-6227960.382	5904774.986
White-naped Crane	-6030982.769	6267909.27
White-naped Crane	-6226503.21	5904802.629
White-naped Crane	-5810734.93	6355068.428
White-naped Crane	-6173190.079	5946241.941
White-naped Crane	-6172515.483	5945967.458
White-naped Crane	-5332268.174	5591300.626
White-naped Crane	-5854977.749	6304843.053
White-naped Crane	-5867375.401	6258997.939
White-naped Crane	-6177026.149	5933535.046
White-naped Crane	-5813619.218	6359634.645
White-naped Crane	-6228852.051	5915318.012
White-naped Crane	-5871022.227	6246940.19
White-naped Crane	-5306620.164	5863493.042
White-naped Crane	-6172803.801	5938164.697
White-naped Crane	-6226323.985	5897577.974
White-naped Crane	-6172755.933	5945052.579
White-naped Crane	-5979018.831	6349491.859
White-naped Crane	-6228365.585	5907059.844
White-naped Crane	-6174544.837	5935231.915
White-naped Crane	-5817529.872	6358437.099
White-naped Crane	-6172782.65	5945268.22
White-naped Crane	-6227094.316	5903350.712
White-naped Crane	-6228992.314	5908208.192
White-naped Crane	-6171392.27	5937005.827
White-naped Crane	-5332361.683	5589596.844
White-naped Crane	-5962491.226	6238242.224
White-naped Crane	-6226505.436	5904703.442
White-naped Crane	-6048536.74	6304353.866
White-naped Crane	-5815627.422	6368237.005
White-naped Crane	-6179111.163	5932053.822
White-naped Crane	-6172269.467	5937743.568
White-naped Crane	-5870799.588	6248734.708
White-naped Crane	-6228267.624	5907006.171
White-naped Crane	-5814503.095	6375454.01
White-naped Crane	-5816827.446	6358061.401
White-naped Crane	-6228499.168	5907006.171
White-naped Crane	-5808681.086	6358793.943
White-naped Crane	-5952673.96	6159763.482
White-naped Crane	-6171721.775	5937392.643
White-naped Crane	-6174894.381	5931949.428
White-naped Crane	-6172981.912	5931889.076
White-naped Crane	-6225081.66	5898941.176
White-naped Crane	-6172133.657	5943923.817
White-naped Crane	-6172685.802	5945122.825
White-naped Crane	-6227875.779	5904781.49
White-naped Crane	-6066351.198	6310908.289
White-naped Crane	-6185242.641	5975232.799
White-naped Crane	-6227264.635	5904700.19
White-naped Crane	-6228112.89	5907128.155
White-naped Crane	-5277653.719	5876040.602
White-naped Crane	-6230113.301	5923359.209

White-naped Crane	-6173502.887	5947926.608
White-naped Crane	-6172926.252	5945454.46
White-naped Crane	-6227149.976	5904718.076
White-naped Crane	-6227323.634	5904661.166
White-naped Crane	-6226406.362	5904820.515
White-naped Crane	-5332257.042	5591286.505
White-naped Crane	-6228523.659	5907020.809
White-naped Crane	-5855296.123	6304827.712
White-naped Crane	-6055150.231	6294557.424
White-naped Crane	-6177187.562	5931646.041
White-naped Crane	-5816737.277	6358243.244
White-naped Crane	-5842977.508	6318608.308
White-naped Crane	-6225963.31	5905230.283
White-naped Crane	-5812962.433	6359816.522
White-naped Crane	-5307489.569	5861463.946
White-naped Crane	-6228209.738	5907045.206
White-naped Crane	-6233666.619	5925116.011
White-naped Crane	-6173541.849	5931760.217
White-naped Crane	-6227270.201	5904719.702
White-naped Crane	-6174322.199	5936227.35
White-naped Crane	-6176201.272	5919771.75
White-naped Crane	-6228170.776	5907045.206
White-naped Crane	-6173072.081	5930672.337
White-naped Crane	-6173490.642	5946336.705
White-naped Crane	-6174161.898	5936011.934
White-naped Crane	-6226457.569	5904734.336
White-naped Crane	-5859177.833	6301688.557
White-naped Crane	-5859092.117	6301710.708
White-naped Crane	-6226285.024	5897495.117
White-naped Crane	-6195116.679	5913931.065
White-naped Crane	-6227933.665	5904758.726
White-naped Crane	-6174808.665	5946307.295
White-naped Crane	-6178913.014	5933388.217
White-naped Crane	-5867407.683	6259036.929
White-naped Crane	-5329607.638	5580938.626
White-naped Crane	-5860039.446	6301804.423
White-naped Crane	-5879774.165	6262382.244
White-naped Crane	-5813628.124	6373613.076
White-naped Crane	-6177645.085	5933140.246
White-naped Crane	-5813767.273	6360036.153
White-naped Crane	-5871396.261	6248807.513
White-naped Crane	-5307182.327	5861277.889
White-naped Crane	-5882150.837	6323984.794
White-naped Crane	-6200524.58	5940522.09
White-naped Crane	-6172529.955	5945327.032
White-naped Crane	-6228470.225	5906991.533
White-naped Crane	-5859174.494	6301719.227
White-naped Crane	-5859824.6	6301729.451
White-naped Crane	-6178356.417	5932655.742
White-naped Crane	-5858903.987	6301840.205
White-naped Crane	-5151506.472	6032630.411
White-naped Crane	-6177183.109	5932810.715
White-naped Crane	-5277419.948	5876157.288
White-naped Crane	-6177788.687	5932982.004
White-naped Crane	-5281020.02	5874042.596
White-naped Crane	-6226978.544	5916446.292
White-naped Crane	-5818941.403	6374788.751
White-naped Crane	-6228012.702	5907111.89

White-naped Crane	-6225948.839	5905218.901
White-naped Crane	-6228127.361	5907017.556
White-naped Crane	-6173752.243	5931517.186
White-naped Crane	-6171795.246	5944049.59
White-naped Crane	-6172636.821	5945230.646
White-naped Crane	-6193918.882	5944534.735
White-naped Crane	-6173195.645	5934660.816
White-naped Crane	-6171557.022	5945209.408
White-naped Crane	-6172998.61	5930616.886
White-naped Crane	-6178861.807	5932562.76
White-naped Crane	-5929428.224	6253476.797
White-naped Crane	-5881308.148	6262199.091
White-naped Crane	-6189464.989	5979858.709
White-naped Crane	-6178001.308	5931908.65
White-naped Crane	-6225906.537	5905186.379
White-naped Crane	-6178255.116	5932644.324
White-naped Crane	-6228067.249	5907139.54
White-naped Crane	-5276996.934	5877056.797
White-naped Crane	-5280306.462	5874941.885
White-naped Crane	-5815297.916	6374972.68
White-naped Crane	-5272759.001	5877076.247
White-naped Crane	-5811546.45	6360827.215
White-naped Crane	-6177390.164	5932505.666
White-naped Crane	-6178178.306	5932704.681
White-naped Crane	-6177223.184	5932375.167
White-naped Crane	-6227271.314	5908341.58
White-naped Crane	-5870965.454	6246679.509
White-naped Crane	-5881856.953	6324210.314
White-naped Crane	-6177096.28	5933998.389
White-naped Crane	-6175646.9	5931039.298
White-naped Crane	-6173809.016	5931448.681
White-naped Crane	-6173469.491	5931432.371
White-naped Crane	-6174344.462	5946223.969
White-naped Crane	-6172423.088	5945697.885
White-naped Crane	-6173191.192	5946246.843
White-naped Crane	-6228088.399	5907027.315
White-naped Crane	-6174131.842	5936801.816
White-naped Crane	-6226406.362	5905041.657
White-naped Crane	-5329674.43	5585773.149
White-naped Crane	-6229231.651	5906843.529
White-naped Crane	-6229970.812	5909525.891
White-naped Crane	-6177706.311	5933115.775
White-naped Crane	-5815160.993	6374826.568
White-naped Crane	-6178701.507	5932794.402
White-naped Crane	-6178478.868	5934014.704
White-naped Crane	-6174248.728	5936289.364
White-naped Crane	-6226257.194	5897464.248
White-naped Crane	-6169283.878	5950098.696
White-naped Crane	-6178623.584	5933112.512
White-naped Crane	-6226426.399	5905165.24
White-naped Crane	-6173213.456	5946426.568
White-naped Crane	-6228531.451	5906835.397
White-naped Crane	-6171728.454	5943414.207
White-naped Crane	-6030951.6	6267892.3
White-naped Crane	-5332310.476	5591280.228
White-naped Crane	-6229194.915	5906893.948
White-naped Crane	-5868350.559	6317083.78
White-naped Crane	-6228345.547	5906989.907

White-naped Crane	-6178407.624	5932698.156
White-naped Crane	-6178363.096	5932665.53
White-naped Crane	-5274403.19	5877564.128
White-naped Crane	-6177668.462	5933102.724
White-naped Crane	-5871613.334	6252667.064
White-naped Crane	-6231462.493	5923722.599
White-naped Crane	-5810495.594	6376244.826
White-naped Crane	-5809155.307	6373771.198
White-naped Crane	-5881813.539	6324205.188
White-naped Crane	-5803349.995	6346092.556
White-naped Crane	-6173082.099	5935320.031
White-naped Crane	-6177414.654	5933673.72
White-naped Crane	-6175633.542	5930819.119
White-naped Crane	-6174206.426	5935204.175
White-naped Crane	-6174538.158	5944096.96
White-naped Crane	-6225310.978	5898978.549
White-naped Crane	-6180255.527	5933349.063
White-naped Crane	-6174296.595	5933378.429
White-naped Crane	-6179428.424	5931944.535
White-naped Crane	-6173188.966	5946282.787
White-naped Crane	-6229400.856	5906633.725
White-naped Crane	-6175545.6	5935586.015
White-naped Crane	-6228564.847	5906858.167
White-naped Crane	-6173445.001	5936166.967
White-naped Crane	-6172153.695	5937712.556
White-naped Crane	-6226346.249	5904999.38
White-naped Crane	-6178959.769	5932252.826
White-naped Crane	-6174095.107	5935514.215
White-naped Crane	-6189964.813	5980017.816
White-naped Crane	-6172216.034	5937575.449
White-naped Crane	-5869007.344	6317005.257
White-naped Crane	-6174284.35	5936349.747
White-naped Crane	-6171860.925	5937738.671
White-naped Crane	-6069710.82	6324164.184
White-naped Crane	-6168887.581	5940282.077
White-naped Crane	-6178363.096	5932636.167
White-naped Crane	-5273921.176	5877557.645
White-naped Crane	-6177645.085	5933055.415
White-naped Crane	-6177706.311	5933115.775
White-naped Crane	-6177273.278	5932094.601
White-naped Crane	-5877299.533	6253312.472
White-naped Crane	-5300771.438	5863659.727
White-naped Crane	-6062368.187	6328667.285
White-naped Crane	-6177770.876	5932123.962
White-naped Crane	-6225900.971	5905051.414
White-naped Crane	-6174480.272	5932022.83
White-naped Crane	-6175860.634	5930951.226
White-naped Crane	-6177276.618	5934053.861
White-naped Crane	-6172627.916	5945199.606
White-naped Crane	-6197009.111	5949613.238
White-naped Crane	-6174671.742	5933889.077
White-naped Crane	-6189782.249	5911200.146
White-naped Crane	-6174151.88	5945261.685
White-naped Crane	-6172848.328	5951618.994
White-naped Crane	-6178062.533	5933052.152
White-naped Crane	-6172745.915	5945951.12
White-naped Crane	-6228228.662	5907066.35
White-naped Crane	-6172861.687	5946155.347

White-naped Crane	-6227336.993	5904735.962
White-naped Crane	-6226464.248	5904731.084
White-naped Crane	-6174593.818	5934992.049
White-naped Crane	-6175370.828	5936625.555
White-naped Crane	-6175809.427	5918972.025
White-naped Crane	-6228194.153	5906887.442
White-naped Crane	-6178025.798	5933022.788
White-naped Crane	-6066558.252	6311401.273
White-naped Crane	-6065662.13	6313593.613
White-naped Crane	-6069777.612	6323257.023
White-naped Crane	-6069512.672	6323942.083
White-naped Crane	-6172120.299	5937049.894
White-naped Crane	-6174542.611	5935246.601
White-naped Crane	-6172611.218	5937258.806
White-naped Crane	-5272977.187	5876585.151
White-naped Crane	-6178365.322	5932641.061
White-naped Crane	-6227153.316	5904948.972
White-naped Crane	-6225617.107	5905171.744
White-naped Crane	-6178353.077	5932652.48
White-naped Crane	-5814799.205	6359396.151
White-naped Crane	-6177009.451	5932252.826
White-naped Crane	-6225250.866	5905145.726
White-naped Crane	-6174555.969	5933907.023
White-naped Crane	-6191118.083	5911904.764
White-naped Crane	-6174388.99	5951978.674
White-naped Crane	-6178792.789	5932875.968
White-naped Crane	-6174495.857	5952050.611
White-naped Crane	-6225368.864	5898281.483
White-naped Crane	-6178314.115	5933783.029
White-naped Crane	-6227123.259	5904511.575
White-naped Crane	-6226994.129	5903358.841
White-naped Crane	-6171844.227	5943660.841
White-naped Crane	-6178284.059	5932473.041
White-naped Crane	-6172792.669	5945952.754
White-naped Crane	-6226791.527	5904911.573
White-naped Crane	-6006381.162	6255543.833
White-naped Crane	-6226364.06	5905022.145
White-naped Crane	-6172931.818	5930437.49
White-naped Crane	-6178584.622	5932809.084
White-naped Crane	-6098853.15	6313194.342
White-naped Crane	-5331580.22	5584937.423
White-naped Crane	-5863101.845	6281173.425
White-naped Crane	-6227985.985	5907128.155
White-naped Crane	-5859426.076	6301889.619
White-naped Crane	-5871545.429	6252882.195
White-naped Crane	-6225308.752	5905119.709
White-naped Crane	-5169326.496	6034280.967
White-naped Crane	-6055588.83	6293791.364
White-naped Crane	-6177680.708	5933161.453
White-naped Crane	-5870772.871	6248904.022
White-naped Crane	-5811413.979	6377878.271
White-naped Crane	-5815294.577	6358299.856
White-naped Crane	-6177053.979	5933466.526
White-naped Crane	-5815354.689	6358325.589
White-naped Crane	-5279221.097	5876004.948
White-naped Crane	-5307294.76	5862910.478
White-naped Crane	-6226134.742	5905201.014
White-naped Crane	-6228737.392	5915853.642

White-naped Crane	-5306820.539	5862989.769
White-naped Crane	-5961490.464	6159912.585
White-naped Crane	-5952760.79	6159793.638
White-naped Crane	-5927676.056	6264682.183
White-naped Crane	-5927328.739	6265352.27
White-naped Crane	-5926182.148	6265527.01
White-naped Crane	-5811235.868	6354687.738
White-naped Crane	-6174023.862	5935140.537
White-naped Crane	-6225014.868	5898582.076
White-naped Crane	-6177243.222	5934008.178
White-naped Crane	-6173467.265	5931254.589
White-naped Crane	-6178441.02	5932549.71
White-naped Crane	-6173413.831	5932079.921
White-naped Crane	-6173331.455	5931619.943
White-naped Crane	-6228528.111	5906902.08
White-naped Crane	-6173886.939	5934807.667
White-naped Crane	-6228534.791	5906820.759
White-naped Crane	-6229778.229	5911914.528
White-naped Crane	-6171545.89	5937027.044
White-naped Crane	-6094564.01	5870655.301
White-naped Crane	-6092958.783	5885385.329
White-naped Crane	-6228441.282	5904958.728
White-naped Crane	-6227453.878	5908273.259
White-naped Crane	-5331459.995	5586124.396
White-naped Crane	-5859069.853	6301819.758
White-naped Crane	-6178207.249	5932505.666
White-naped Crane	-6178105.948	5932517.085
White-naped Crane	-6228318.831	5906936.235
White-naped Crane	-6224826.738	5929410.11
White-naped Crane	-6178471.076	5932773.195
White-naped Crane	-6178295.191	5933451.843
White-naped Crane	-5152071.975	6032188.116
White-naped Crane	-6054866.366	6293881.586
White-naped Crane	-6177681.821	5933140.246
White-naped Crane	-6178821.732	5930280.929
White-naped Crane	-6226208.213	5905001.006
White-naped Crane	-6227981.533	5907121.649
White-naped Crane	-5871775.86	6248890.477
White-naped Crane	-5277756.133	5876821.781
White-naped Crane	-6177459.182	5933169.61
White-naped Crane	-5280171.766	5873843.306
White-naped Crane	-6228509.187	5914912.649
White-naped Crane	-5307583.077	5861572.347
White-naped Crane	-5307209.044	5862860.314
White-naped Crane	-6177695.179	5931735.751
White-naped Crane	-6178339.719	5932645.955
White-naped Crane	-5871066.755	6248174.303
White-naped Crane	-5881315.94	6323988.211
White-naped Crane	-5881416.128	6324007.004
White-naped Crane	-6171496.91	5938087.978
White-naped Crane	-6174222.011	5934285.543
White-naped Crane	-6177016.13	5933936.391
White-naped Crane	-6174598.271	5934706.503
White-naped Crane	-6178311.889	5933017.894
White-naped Crane	-6176628.738	5918716.324
White-naped Crane	-6178298.531	5932952.64
White-naped Crane	-6172374.107	5945003.57
White-naped Crane	-6171151.819	5937350.207

White-naped Crane	-6175232.792	5945034.609
White-naped Crane	-6174520.347	5944243.972
White-naped Crane	-6173396.02	5931403.012
White-naped Crane	-6193950.051	5944490.629
White-naped Crane	-6227862.421	5904758.726
White-naped Crane	-6174139.635	5951169.416
White-naped Crane	-6170860.162	5936243.669
White-naped Crane	-6172910.667	5945451.193
White-naped Crane	-6175292.904	5945717.49
White-naped Crane	-6173309.191	5931566.118
White-naped Crane	-6179075.541	5933580.727
White-naped Crane	-6173102.137	5931199.134
White-naped Crane	-6172852.781	5946349.776
White-naped Crane	-6173202.324	5946258.28
White-naped Crane	-6170800.05	5944281.541
White-naped Crane	-6171494.683	5945246.982
White-naped Crane	-6172006.753	5943548.14
White-naped Crane	-6171806.378	5935883.014
White-naped Crane	-6173295.833	5931391.595
White-naped Crane	-6178518.943	5933477.946
White-naped Crane	-6178245.097	5932769.933
White-naped Crane	-6030958.279	6267897.391
White-naped Crane	-6173716.62	5935763.887
White-naped Crane	-6177205.373	5933903.76
White-naped Crane	-6228641.657	5906446.695
White-naped Crane	-5870660.439	6249776.044
White-naped Crane	-6178098.156	5932871.074
White-naped Crane	-6171809.718	5937738.671
White-naped Crane	-6172673.557	5937053.158
White-naped Crane	-6174092.88	5936282.836
White-naped Crane	-5929441.583	6253334.495
White-naped Crane	-6172121.412	5936460.722
White-naped Crane	-6228042.758	5907131.408
White-naped Crane	-5872729.868	6263988.403
White-naped Crane	-5854743.978	6307732.74
White-naped Crane	-5855336.198	6304667.488
White-naped Crane	-6228098.418	5907098.879
White-naped Crane	-5381538.181	5958105.346
White-naped Crane	-6178797.242	5933001.58
White-naped Crane	-5870690.495	6249522.048
White-naped Crane	-6225892.066	5905092.066
White-naped Crane	-6178281.833	5932982.004
White-naped Crane	-6054859.687	6294179.492
White-naped Crane	-6177255.467	5933220.183
White-naped Crane	-6189955.908	5979789.819
White-naped Crane	-6177717.443	5932024.461
White-naped Crane	-5281183.66	5873802.801
White-naped Crane	-6177789.801	5933396.374
White-naped Crane	-5858528.841	6302296.868
White-naped Crane	-6177417.994	5933401.269
White-naped Crane	-5302559.229	5871946.237
White-naped Crane	-6226557.756	5910563.923
White-naped Crane	-5811568.713	6360846.092
White-naped Crane	-5813632.577	6359684.403
White-naped Crane	-6177591.652	5932722.625
White-naped Crane	-5871181.414	6249130.909
White-naped Crane	-6228646.11	5915785.262
White-naped Crane	-5928542.121	6255418.441

White-naped Crane	-5278022.186	5876797.47
White-naped Crane	-6172104.714	5938461.784
White-naped Crane	-6173482.85	5934225.175
White-naped Crane	-6176938.207	5934027.756
White-naped Crane	-6177140.808	5934008.178
White-naped Crane	-6175718.145	5934050.598
White-naped Crane	-6173843.525	5931068.655
White-naped Crane	-6170697.636	5936147.384
White-naped Crane	-6177755.292	5934037.546
White-naped Crane	-6173825.714	5935481.579
White-naped Crane	-6165830.748	5933742.242
White-naped Crane	-6177508.162	5930646.242
White-naped Crane	-6178176.079	5931903.756
White-naped Crane	-6179229.162	5933448.58
White-naped Crane	-6191725.888	5989103.241
White-naped Crane	-6179359.405	5932073.396
White-naped Crane	-6172015.659	5943553.04
White-naped Crane	-6179250.312	5932022.83
White-naped Crane	-6173629.791	5931497.613
White-naped Crane	-6176909.263	5950239.272
White-naped Crane	-6174138.521	5935816.107
White-naped Crane	-6173042.024	5930698.431
White-naped Crane	-6228520.319	5906872.804
White-naped Crane	-6171711.756	5937297.978
White-naped Crane	-6175825.012	5918985.055
White-naped Crane	-6173945.939	5934856.617
White-naped Crane	-6174161.898	5935778.574
White-naped Crane	-6227279.107	5904822.141
White-naped Crane	-6226395.23	5905046.536
White-naped Crane	-5332147.949	5589625.081
White-naped Crane	-6178396.492	5932831.922
White-naped Crane	-5978994.341	6349517.565
White-naped Crane	-5978989.888	6349498.714
White-naped Crane	-6171585.965	5944497.164
White-naped Crane	-6172556.671	5945287.824
White-naped Crane	-6171752.945	5937459.563
White-naped Crane	-6178341.945	5932717.731
White-naped Crane	-6178542.32	5933218.552
White-naped Crane	-6229287.31	5906664.626
White-naped Crane	-6178674.791	5932931.433
White-naped Crane	-6178623.584	5933003.212
White-naped Crane	-5933844.269	6249237.581
White-naped Crane	-6174299.935	5936366.067
White-naped Crane	-6178374.228	5932639.43
White-naped Crane	-6066574.95	6311334.744
White-naped Crane	-6069753.122	6323333.898
White-naped Crane	-6171662.776	5937697.865
White-naped Crane	-6227175.58	5904493.69
White-naped Crane	-5859386.001	6301628.922
White-naped Crane	-5862533.003	6272762.425
White-naped Crane	-6178007.987	5932836.816
White-naped Crane	-5402455.113	5762904.055
White-naped Crane	-5858890.629	6301724.339
White-naped Crane	-6178551.226	5932600.279
White-naped Crane	-5884118.965	6259248.833
White-naped Crane	-5876357.77	6252848.316
White-naped Crane	-5381393.466	5958192.06
White-naped Crane	-6178235.079	5933438.791

White-naped Crane	-6189892.456	5979663.521
White-naped Crane	-6048173.838	6304684.533
White-naped Crane	-6001454.161	6303764.153
White-naped Crane	-6177175.317	5933252.811
White-naped Crane	-6228126.248	5907074.482
White-naped Crane	-6177255.467	5933229.971
White-naped Crane	-6228052.777	5907118.396
White-naped Crane	-5815127.598	6358099.141
White-naped Crane	-6228018.268	5907113.517
White-naped Crane	-5270830.947	5873585.695
White-naped Crane	-6225707.275	5905140.848
White-naped Crane	-5814359.493	6359516.255
White-naped Crane	-6177212.052	5932327.862
White-naped Crane	-5306591.221	5866966.582
White-naped Crane	-5307005.329	5861920.209
White-naped Crane	-6178166.061	5932231.621
White-naped Crane	-5871963.99	6249069.954
White-naped Crane	-5871758.049	6248858.307
White-naped Crane	-5881718.917	6323947.208
White-naped Crane	-5919251.396	6272674.129
White-naped Crane	-6175148.189	5935966.241
White-naped Crane	-6178906.335	5932539.923
White-naped Crane	-6174237.596	5935524.006
White-naped Crane	-6175152.642	5935964.609
White-naped Crane	-6178879.618	5932535.029
White-naped Crane	-6174237.596	5935522.374
White-naped Crane	-6178877.392	5932536.66
White-naped Crane	-6175152.642	5935969.505
White-naped Crane	-6174555.969	5935486.474
White-naped Crane	-6175150.416	5935967.873
White-naped Crane	-6178870.713	5932546.448
White-naped Crane	-6174237.596	5935525.638
White-naped Crane	-6178857.355	5932566.023
White-naped Crane	-6174244.275	5935524.006
White-naped Crane	-6175152.642	5935976.032
White-naped Crane	-6178763.846	5932446.941
White-naped Crane	-6171732.907	5937474.252
White-naped Crane	-6171744.039	5937448.137
White-naped Crane	-6171864.264	5937639.105
White-naped Crane	-6178699.281	5932490.985
White-naped Crane	-6171784.114	5937603.196
White-naped Crane	-6178643.621	5932399.636
White-naped Crane	-6174173.03	5934838.669
White-naped Crane	-6173560.773	5936039.677
White-naped Crane	-6178545.66	5932530.135
White-naped Crane	-6174090.654	5935072.004
White-naped Crane	-6178542.32	5932531.766
White-naped Crane	-6174177.483	5934739.136
White-naped Crane	-6178427.661	5932637.798
White-naped Crane	-6174173.03	5934636.341
White-naped Crane	-6174166.351	5934637.973
White-naped Crane	-6174753.005	5934368.754
White-naped Crane	-6174696.232	5933978.81
White-naped Crane	-6178750.488	5931998.363
White-naped Crane	-6174722.949	5934223.543
White-naped Crane	-6174531.479	5933908.655
White-naped Crane	-6178790.563	5932091.339
White-naped Crane	-6174720.722	5934220.28

White-naped Crane	-6178870.713	5932551.341
White-naped Crane	-6176408.326	5920595.984
White-naped Crane	-6174691.779	5934200.701
White-naped Crane	-6178855.128	5932574.179
White-naped Crane	-6176432.816	5920402.135
White-naped Crane	-6174720.722	5934218.648
White-naped Crane	-6173825.714	5934864.776
White-naped Crane	-6176423.911	5920410.28
White-naped Crane	-6175268.414	5933791.187
White-naped Crane	-6174132.955	5934892.515
White-naped Crane	-6174718.496	5934200.701
White-naped Crane	-6177145.261	5933135.351
White-naped Crane	-6171904.339	5937439.977
White-naped Crane	-6176519.645	5920146.392
White-naped Crane	-6174685.1	5934150.122
White-naped Crane	-6176519.645	5920144.763
White-naped Crane	-6177156.393	5933133.72
White-naped Crane	-6173758.922	5935112.797
White-naped Crane	-6174567.101	5934735.873
White-naped Crane	-6177238.769	5933008.106
White-naped Crane	-6174342.236	5933947.811
White-naped Crane	-6176368.251	5920149.65
White-naped Crane	-6171087.254	5937213.106
White-naped Crane	-6172149.242	5938698.482
White-naped Crane	-6177236.543	5933004.843
White-naped Crane	-6176274.742	5920056.802
White-naped Crane	-6171768.529	5938143.476
White-naped Crane	-6171797.472	5937348.575
White-naped Crane	-6171764.077	5938143.476
White-naped Crane	-6176268.063	5920043.771
White-naped Crane	-6178870.713	5932548.079
White-naped Crane	-6176417.231	5931884.183
White-naped Crane	-6177240.996	5934011.441
White-naped Crane	-6171764.077	5938143.476
White-naped Crane	-6176270.29	5920037.256
White-naped Crane	-6172701.387	5936888.316
White-naped Crane	-6178850.675	5932556.235
White-naped Crane	-6177205.373	5934044.072
White-naped Crane	-6175433.167	5932717.731
White-naped Crane	-6171766.303	5938145.109
White-naped Crane	-6175433.167	5932720.994
White-naped Crane	-6172581.162	5936682.677
White-naped Crane	-6178843.996	5932552.973
White-naped Crane	-6176272.516	5920048.658
White-naped Crane	-6177205.373	5934045.703
White-naped Crane	-6175433.167	5932729.15
White-naped Crane	-6178848.449	5932569.285
White-naped Crane	-6172585.614	5936700.629
White-naped Crane	-6176252.478	5920009.564
White-naped Crane	-6177212.052	5934045.703
White-naped Crane	-6175433.167	5932722.625
White-naped Crane	-6172587.841	5936687.573
White-naped Crane	-6171127.329	5937740.304
White-naped Crane	-6176270.29	5920043.771
White-naped Crane	-6178850.675	5932556.235
White-naped Crane	-6177205.373	5934035.914
White-naped Crane	-6171127.329	5937743.568
White-naped Crane	-6178855.128	5932561.129

White-naped Crane	-6172605.652	5936695.733
White-naped Crane	-6175442.073	5932717.731
White-naped Crane	-6177205.373	5934031.019
White-naped Crane	-6176265.837	5920037.256
White-naped Crane	-6171127.329	5937743.568
White-naped Crane	-6178843.996	5932559.498
White-naped Crane	-6172601.199	5936697.365
White-naped Crane	-6176263.61	5920047.029
White-naped Crane	-6175430.941	5932714.469
White-naped Crane	-6177198.694	5934034.282
White-naped Crane	-6171127.329	5937748.465
White-naped Crane	-6172601.199	5936692.469
White-naped Crane	-6176268.063	5920047.029
White-naped Crane	-6178846.223	5932559.498
White-naped Crane	-6175428.714	5932719.363
White-naped Crane	-6177203.147	5934021.23
White-naped Crane	-6171127.329	5937743.568
White-naped Crane	-6172601.199	5936702.261
White-naped Crane	-6178846.223	5932556.235
White-naped Crane	-6175428.714	5932720.994
White-naped Crane	-6176263.61	5920020.967
White-naped Crane	-6177198.694	5934022.862
White-naped Crane	-6171129.556	5937740.304
White-naped Crane	-6172601.199	5936697.365
White-naped Crane	-6178881.845	5932580.704
White-naped Crane	-6175435.393	5932720.994
White-naped Crane	-6176268.063	5920020.967
White-naped Crane	-6177203.147	5934037.546
White-naped Crane	-6170958.124	5937673.382
White-naped Crane	-6177060.658	5933680.246
White-naped Crane	-6176272.516	5920060.06
White-naped Crane	-6172552.219	5936692.469
White-naped Crane	-6178786.11	5932585.598
White-naped Crane	-6177200.921	5934040.809
White-naped Crane	-6170960.35	5937671.75
White-naped Crane	-6171601.55	5937188.624
White-naped Crane	-6173099.911	5935347.771
White-naped Crane	-6171594.871	5937198.417
White-naped Crane	-6176196.819	5920056.802
White-naped Crane	-6178939.731	5932030.986
White-naped Crane	-6171000.425	5937546.069
White-naped Crane	-6173102.137	5935337.981
White-naped Crane	-6176187.913	5920064.947
White-naped Crane	-6171541.438	5937325.724
White-naped Crane	-6171557.022	5937315.932
White-naped Crane	-6173097.684	5935339.612
White-naped Crane	-6171002.651	5937550.966
White-naped Crane	-6176112.216	5920340.235
White-naped Crane	-6177403.522	5933859.71
White-naped Crane	-6173093.231	5935349.403
White-naped Crane	-6171000.425	5937554.23
White-naped Crane	-6186099.801	5913649.47
White-naped Crane	-6176112.216	5920340.235
White-naped Crane	-6177276.618	5933786.292
White-naped Crane	-6176104.424	5920345.122
White-naped Crane	-6176103.31	5920353.267
White-naped Crane	-6178739.356	5932102.757
White-naped Crane	-6173211.23	5935120.956

White-naped Crane	-6176084.386	5920159.423
White-naped Crane	-6171044.953	5937516.69
White-naped Crane	-6177033.941	5933768.346
White-naped Crane	-6185436.336	5911144.82
White-naped Crane	-6171049.405	5937526.483
White-naped Crane	-6185436.336	5911185.501
White-naped Crane	-6176207.951	5920074.72
White-naped Crane	-6173727.752	5934220.28
White-naped Crane	-6178492.227	5931588.953
White-naped Crane	-6176993.866	5933783.029
White-naped Crane	-6178131.551	5931911.912
White-naped Crane	-6171056.085	5937534.644
White-naped Crane	-6176190.14	5920066.576
White-naped Crane	-6185437.45	5911154.583
White-naped Crane	-6173736.658	5934210.49
White-naped Crane	-6176993.866	5933796.081
White-naped Crane	-6178136.004	5933185.924
White-naped Crane	-6171056.085	5937536.276
White-naped Crane	-6176275.856	5920063.318
White-naped Crane	-6174001.598	5934021.23
White-naped Crane	-6177002.772	5933779.766
White-naped Crane	-6185461.94	5911162.719
White-naped Crane	-6178124.872	5933172.873
White-naped Crane	-6176281.422	5920060.06
White-naped Crane	-6185449.695	5911154.583
White-naped Crane	-6173990.466	5934027.756
White-naped Crane	-6171049.405	5937531.379
White-naped Crane	-6177002.772	5933781.398
White-naped Crane	-6185459.714	5911196.891
White-naped Crane	-6178138.231	5933161.453
White-naped Crane	-6171018.236	5937547.701
White-naped Crane	-6176278.082	5920066.576
White-naped Crane	-6177007.225	5933784.661
White-naped Crane	-6173979.334	5934016.336
White-naped Crane	-6176276.969	5920019.338
White-naped Crane	-6171002.651	5937570.552
White-naped Crane	-6185367.318	5925428.948
White-naped Crane	-6177004.998	5933774.872
White-naped Crane	-6178104.835	5933125.563
White-naped Crane	-6178866.26	5932551.341
White-naped Crane	-6170991.519	5937559.127
White-naped Crane	-6176274.742	5920064.947
White-naped Crane	-6174010.504	5934031.019
White-naped Crane	-6193853.203	5936658.196
White-naped Crane	-6177007.225	5933779.766
White-naped Crane	-6176201.272	5920066.576
White-naped Crane	-6178864.034	5932551.341
White-naped Crane	-6170987.067	5937549.334
White-naped Crane	-6201875.999	5941594.885
White-naped Crane	-6177136.355	5934001.652
White-naped Crane	-6170978.161	5937529.747
White-naped Crane	-6176550.815	5934970.837
White-naped Crane	-6176214.63	5920066.576
White-naped Crane	-6206230.817	5944698.089
White-naped Crane	-6175471.016	5934481.336
White-naped Crane	-6178781.657	5932428.998
White-naped Crane	-6170980.387	5937521.586
White-naped Crane	-6175479.921	5934531.916

White-naped Crane	-6176212.403	5920071.462
White-naped Crane	-6206204.101	5944781.401
White-naped Crane	-6176555.268	5934974.1
White-naped Crane	-6178768.299	5932471.41
White-naped Crane	-6175479.921	5934536.811
White-naped Crane	-6178768.299	5932477.935
White-naped Crane	-6170942.539	5937544.437
White-naped Crane	-6206208.553	5944794.47
White-naped Crane	-6176555.268	5934980.627
White-naped Crane	-6176214.63	5920069.834
White-naped Crane	-6176216.856	5920073.091
White-naped Crane	-6178752.714	5932458.36
White-naped Crane	-6206210.78	5944787.935
White-naped Crane	-6170958.124	5937559.127
White-naped Crane	-6176553.041	5934980.627
White-naped Crane	-6175493.28	5934551.496
White-naped Crane	-6176214.63	5920066.576
White-naped Crane	-6206192.969	5944763.432
White-naped Crane	-6170953.671	5937534.644
White-naped Crane	-6176561.947	5934992.049
White-naped Crane	-6175488.827	5934540.074
White-naped Crane	-6178710.413	5932473.041
White-naped Crane	-6170958.124	5937521.586
White-naped Crane	-6175479.921	5934538.443
White-naped Crane	-6176216.856	5920061.689
White-naped Crane	-6206201.874	5944781.401
White-naped Crane	-6176564.173	5934977.363
White-naped Crane	-6178705.96	5932484.46
White-naped Crane	-6176210.177	5920063.318
White-naped Crane	-6170953.671	5937533.012
White-naped Crane	-6176550.815	5934980.627
White-naped Crane	-6175444.299	5934523.758
White-naped Crane	-6206204.101	5944781.401
White-naped Crane	-6178705.96	5932492.616
White-naped Crane	-6178703.734	5932494.247
White-naped Crane	-6176216.856	5920066.576
White-naped Crane	-6170944.765	5937542.805
White-naped Crane	-6176555.268	5934982.259
White-naped Crane	-6175479.921	5934558.022
White-naped Crane	-6206201.874	5944787.935
White-naped Crane	-6206204.101	5944791.202
White-naped Crane	-6176555.268	5934977.363
White-naped Crane	-6170944.765	5937533.012
White-naped Crane	-6176214.63	5920066.576
White-naped Crane	-6178703.734	5932484.46
White-naped Crane	-6175486.6	5934554.759
White-naped Crane	-6178705.96	5932484.46
White-naped Crane	-6170951.444	5937537.908
White-naped Crane	-6176219.083	5920066.576
White-naped Crane	-6206206.327	5944784.668
White-naped Crane	-6176548.588	5934975.732
White-naped Crane	-6175482.148	5934544.969
White-naped Crane	-6203999.975	5947361.203
White-naped Crane	-6177461.408	5933109.25
White-naped Crane	-6170962.576	5937502
White-naped Crane	-6176216.856	5920061.689
White-naped Crane	-6176548.588	5934977.363
White-naped Crane	-6175486.6	5934549.864

White-naped Crane	-6171051.632	5937389.379
White-naped Crane	-6177692.953	5933176.136
White-naped Crane	-6176212.403	5920066.576
White-naped Crane	-6175517.77	5934546.601
White-naped Crane	-6171365.553	5936981.345
White-naped Crane	-6178006.874	5933352.326
White-naped Crane	-6171122.876	5937386.115
White-naped Crane	-6176029.84	5920537.34
White-naped Crane	-6171398.949	5937062.95
White-naped Crane	-6175491.053	5934533.548
White-naped Crane	-6177232.09	5934040.809
White-naped Crane	-6176575.305	5931194.241
White-naped Crane	-6176216.856	5920758.886
White-naped Crane	-6175495.506	5934531.916
White-naped Crane	-6197104.846	5940882.94
White-naped Crane	-6174442.424	5933882.551
White-naped Crane	-6199999.152	5945353.171
White-naped Crane	-6176223.535	5920765.402
White-naped Crane	-6175495.506	5934531.916
White-naped Crane	-6177232.09	5934058.756
White-naped Crane	-6175482.148	5934500.915
White-naped Crane	-6175455.431	5934455.23
White-naped Crane	-6205671.994	5945990.331
White-naped Crane	-6174469.14	5933880.919
White-naped Crane	-6176949.338	5934058.756
White-naped Crane	-6176176.781	5920077.978
White-naped Crane	-6175016.832	5933115.775
White-naped Crane	-6175373.055	5933287.07
White-naped Crane	-6173810.129	5934146.859
White-naped Crane	-6176973.829	5934084.86
White-naped Crane	-6176194.592	5920063.318
White-naped Crane	-6175375.281	5933283.807
White-naped Crane	-6175001.247	5933122.301
White-naped Crane	-6233555.3	5925301.816
White-naped Crane	-6173790.091	5934143.596
White-naped Crane	-6176973.829	5934091.387
White-naped Crane	-6176230.215	5920082.865
White-naped Crane	-6175377.507	5933290.333
White-naped Crane	-6233443.98	5924767.229
White-naped Crane	-6175019.059	5933107.618
White-naped Crane	-6173881.373	5934225.175
White-naped Crane	-6174874.343	5932714.469
White-naped Crane	-6176924.848	5934066.913
White-naped Crane	-6233492.961	5924847.089
White-naped Crane	-6176976.055	5934094.65
White-naped Crane	-6176969.376	5934037.546
White-naped Crane	-6233541.941	5924812.863
White-naped Crane	-6174896.607	5932703.05
White-naped Crane	-6176924.848	5920338.606
White-naped Crane	-6177025.036	5934605.34
White-naped Crane	-6174253.18	5934375.281
White-naped Crane	-6233590.922	5924840.57
White-naped Crane	-6176891.452	5920332.09
White-naped Crane	-6177067.337	5934587.392
White-naped Crane	-6174620.535	5932014.674
White-naped Crane	-6177058.432	5931522.079
White-naped Crane	-6176931.527	5920361.411
White-naped Crane	-6233684.43	5924876.426

White-naped Crane	-6174531.479	5931774.897
White-naped Crane	-6177045.073	5934585.76
White-naped Crane	-6177568.275	5932504.035
White-naped Crane	-6174849.853	5935080.162
White-naped Crane	-6177465.861	5919765.235
White-naped Crane	-6173006.402	5930806.072
White-naped Crane	-6233635.45	5925006.811
White-naped Crane	-6174863.211	5935106.27
White-naped Crane	-6176898.132	5920335.348
White-naped Crane	-6173006.402	5930788.132
White-naped Crane	-6177851.026	5932670.424
White-naped Crane	-6233588.695	5924992.143
White-naped Crane	-6178527.849	5933823.817
White-naped Crane	-6174860.985	5935106.27
White-naped Crane	-6175891.803	5930835.429
White-naped Crane	-6176895.905	5920336.977
White-naped Crane	-6177886.649	5932735.675
White-naped Crane	-6233579.79	5924992.143
White-naped Crane	-6178583.509	5933836.869
White-naped Crane	-6174860.985	5935112.797
White-naped Crane	-6233630.997	5925125.79
White-naped Crane	-6178029.138	5932827.028
White-naped Crane	-6175889.577	5930838.69
White-naped Crane	-6176900.358	5920340.235
White-naped Crane	-6178559.018	5933848.289
White-naped Crane	-6178033.59	5932838.448
White-naped Crane	-6176902.584	5920336.977
White-naped Crane	-6174860.985	5935111.165
White-naped Crane	-6233628.77	5925129.049
White-naped Crane	-6175896.256	5930845.214
White-naped Crane	-6178563.471	5933843.395
White-naped Crane	-6233633.223	5925129.049
White-naped Crane	-6174863.211	5935099.743
White-naped Crane	-6176900.358	5920338.606
White-naped Crane	-6178033.59	5932827.028
White-naped Crane	-6175891.803	5930830.536
White-naped Crane	-6178561.245	5933836.869
White-naped Crane	-6174856.532	5935106.27
White-naped Crane	-6176900.358	5920335.348
White-naped Crane	-6175891.803	5930830.536
White-naped Crane	-6178554.566	5933836.869
White-naped Crane	-6178026.911	5932827.028
White-naped Crane	-6174856.532	5935101.375
White-naped Crane	-6175889.577	5930830.536
White-naped Crane	-6178026.911	5932830.291
White-naped Crane	-6176900.358	5920335.348
White-naped Crane	-6178556.792	5933840.132
White-naped Crane	-6233630.997	5925117.64
White-naped Crane	-6178029.138	5932835.185
White-naped Crane	-6174860.985	5935109.534
White-naped Crane	-6233628.77	5925122.53
White-naped Crane	-6175845.049	5930566.329
White-naped Crane	-6176900.358	5920340.235
White-naped Crane	-6178559.018	5933843.395
White-naped Crane	-6178031.364	5932831.922
White-naped Crane	-6174887.702	5935094.848
White-naped Crane	-6175842.823	5930550.02
White-naped Crane	-6178559.018	5933840.132

White-naped Crane	-6176898.132	5920338.606
White-naped Crane	-6233633.223	5925125.79
White-naped Crane	-6178035.817	5932830.291
White-naped Crane	-6174889.928	5935094.848
White-naped Crane	-6233633.223	5925127.42
White-naped Crane	-6175838.37	5930553.282
White-naped Crane	-6176902.584	5920341.864
White-naped Crane	-6178570.15	5933838.5
White-naped Crane	-6174963.399	5932603.542
White-naped Crane	-6177149.714	5933990.231
White-naped Crane	-6174894.381	5935093.216
White-naped Crane	-6177345.636	5919722.885
White-naped Crane	-6233688.883	5924881.315
White-naped Crane	-6175232.792	5929785.171
White-naped Crane	-6174885.475	5935098.111
White-naped Crane	-6176984.961	5918703.295
White-naped Crane	-6233653.261	5924806.344
White-naped Crane	-6171410.081	5936747.959
White-naped Crane	-6177143.034	5933996.757
White-naped Crane	-6175241.698	5929773.756
White-naped Crane	-6177049.526	5934163.175
White-naped Crane	-6171528.079	5937348.575
White-naped Crane	-6233673.298	5924946.508
White-naped Crane	-6176956.018	5918662.579
White-naped Crane	-6177127.45	5933988.599
White-naped Crane	-6175237.245	5929772.125
White-naped Crane	-6174028.315	5934744.031
White-naped Crane	-6177009.451	5934220.28
White-naped Crane	-6231987.921	5923652.527
White-naped Crane	-6176962.697	5918670.722
White-naped Crane	-6177127.45	5933996.757
White-naped Crane	-6175241.698	5929778.648
White-naped Crane	-6174030.541	5934748.926
White-naped Crane	-6174242.048	5934815.825
White-naped Crane	-6176960.47	5918628.378
White-naped Crane	-6176911.49	5934073.439
White-naped Crane	-6175243.924	5929790.064
White-naped Crane	-6231963.431	5923579.197
White-naped Crane	-6176921.509	5934045.703
White-naped Crane	-6175241.698	5929786.802
White-naped Crane	-6176694.417	5918864.532
White-naped Crane	-6176717.794	5918898.734
White-naped Crane	-6174754.118	5934804.403
White-naped Crane	-6176913.716	5934287.175
White-naped Crane	-6175250.603	5929793.325
White-naped Crane	-6177122.997	5933986.968
White-naped Crane	-6174865.438	5934773.401
White-naped Crane	-6176922.622	5934288.806
White-naped Crane	-6176702.209	5918892.219
White-naped Crane	-6175246.15	5929850.401
White-naped Crane	-6177125.223	5933991.863
White-naped Crane	-6246141.081	5904615.638
White-naped Crane	-6177254.354	5933796.081
White-naped Crane	-6176951.565	5934287.175
White-naped Crane	-6176682.172	5918825.444
White-naped Crane	-6246147.76	5904631.898
White-naped Crane	-6175243.924	5929863.447
White-naped Crane	-6177149.714	5933384.954

White-naped Crane	-6176976.055	5934096.281
White-naped Crane	-6175310.716	5931293.733
White-naped Crane	-6176717.794	5918900.363
White-naped Crane	-6246145.534	5904636.776
White-naped Crane	-6176971.602	5934094.65
White-naped Crane	-6177232.09	5933004.843
White-naped Crane	-6245853.877	5904716.45
White-naped Crane	-6176982.734	5934119.123
White-naped Crane	-6176706.662	5918898.734
White-naped Crane	-6175308.489	5931243.172
White-naped Crane	-6177042.847	5934166.438
White-naped Crane	-6179028.787	5932233.252
White-naped Crane	-6176711.115	5918900.363
White-naped Crane	-6175315.168	5931243.172
White-naped Crane	-6176987.187	5934130.544
White-naped Crane	-6176757.869	5918869.418
White-naped Crane	-6177120.771	5934138.702
White-naped Crane	-6223690.166	5895774.783
White-naped Crane	-6177962.346	5930659.29
White-naped Crane	-6176980.508	5934109.334
White-naped Crane	-6175308.489	5931244.803
White-naped Crane	-6176842.472	5918727.725
White-naped Crane	-6220671.182	5897134.452
White-naped Crane	-6171706.19	5937056.422
White-naped Crane	-6176913.716	5934057.124
White-naped Crane	-6175359.696	5931200.765
White-naped Crane	-6175072.492	5932314.812
White-naped Crane	-6224551.779	5897191.312
White-naped Crane	-6176833.566	5918750.526
White-naped Crane	-6175724.824	5934070.176
White-naped Crane	-6171675.021	5937044.997
White-naped Crane	-6175319.621	5931182.824
White-naped Crane	-6175738.182	5934053.861
White-naped Crane	-6178648.074	5931729.226
White-naped Crane	-6224545.1	5897189.688
White-naped Crane	-6176964.923	5918623.492
White-naped Crane	-6174729.628	5935303.713
White-naped Crane	-6175731.503	5934045.703
White-naped Crane	-6178668.111	5931739.013
White-naped Crane	-6224529.515	5897155.571
White-naped Crane	-6174718.496	5935290.659
White-naped Crane	-6176967.15	5918625.12
White-naped Crane	-6175631.316	5930961.011
White-naped Crane	-6224520.61	5897163.694
White-naped Crane	-6178768.299	5931973.895
White-naped Crane	-6175733.73	5934057.124
White-naped Crane	-6176964.923	5918621.863
White-naped Crane	-6175807.201	5931057.238
White-naped Crane	-6174718.496	5935293.922
White-naped Crane	-6176962.697	5918630.006
White-naped Crane	-6224527.289	5897166.944
White-naped Crane	-6178770.525	5931978.789
White-naped Crane	-6175738.182	5934058.756
White-naped Crane	-6174727.401	5935298.818
White-naped Crane	-6175800.521	5931058.869
White-naped Crane	-6175735.956	5934058.756
White-naped Crane	-6176962.697	5918623.492
White-naped Crane	-6178770.525	5931972.264

White-naped Crane	-6175800.521	5931057.238
White-naped Crane	-6174707.364	5935289.027
White-naped Crane	-6224527.289	5897162.07
White-naped Crane	-6175802.748	5931055.607
White-naped Crane	-6176960.47	5918626.749
White-naped Crane	-6175740.409	5934052.229
White-naped Crane	-6178766.073	5931972.264
White-naped Crane	-6224527.289	5897158.821
White-naped Crane	-6174745.213	5935285.764
White-naped Crane	-6178768.299	5931967.371
White-naped Crane	-6176956.018	5918625.12
White-naped Crane	-6175798.295	5931053.976
White-naped Crane	-6175733.73	5934055.492
White-naped Crane	-6224520.61	5897163.694
White-naped Crane	-6174689.553	5935285.764
White-naped Crane	-6175800.521	5931055.607
White-naped Crane	-6178768.299	5931973.895
White-naped Crane	-6175735.956	5934062.019
White-naped Crane	-6176962.697	5918625.12
White-naped Crane	-6224520.61	5897162.07
White-naped Crane	-6174729.628	5935289.027
White-naped Crane	-6224522.836	5897162.07
White-naped Crane	-6178766.073	5931972.264
White-naped Crane	-6175800.521	5931057.238
White-naped Crane	-6176964.923	5918625.12
White-naped Crane	-6175735.956	5934058.756
White-naped Crane	-6174711.817	5935279.236
White-naped Crane	-6175811.653	5931062.131
White-naped Crane	-6175738.182	5934053.861
White-naped Crane	-6178768.299	5931970.633
White-naped Crane	-6176964.923	5918625.12
White-naped Crane	-6224527.289	5897162.07
White-naped Crane	-6174720.722	5935293.922
White-naped Crane	-6177719.669	5919411.783
White-naped Crane	-6171510.268	5937505.264
White-naped Crane	-6223440.811	5895836.508
White-naped Crane	-6178783.884	5932159.848
White-naped Crane	-6175555.618	5931084.965
White-naped Crane	-6177590.539	5919590.95
White-naped Crane	-6178113.74	5931336.14
White-naped Crane	-6175733.73	5934066.913
White-naped Crane	-6223567.715	5895917.725
White-naped Crane	-6175531.128	5931111.061
White-naped Crane	-6173701.036	5935222.125
White-naped Crane	-6176829.113	5920004.678
White-naped Crane	-6177951.214	5932295.238
White-naped Crane	-6175551.166	5931084.965
White-naped Crane	-6174224.237	5934531.916
White-naped Crane	-6171363.326	5936682.677
White-naped Crane	-6224262.348	5902242.021
White-naped Crane	-6174388.99	5931256.22
White-naped Crane	-6174179.71	5934548.232
White-naped Crane	-6171097.273	5936290.996
White-naped Crane	-6224513.931	5897179.94
White-naped Crane	-6174235.369	5934491.125
White-naped Crane	-6174346.689	5934407.913
White-naped Crane	-6173683.225	5931753.693
White-naped Crane	-6173643.15	5931750.431

White-naped Crane	-6173642.036	5931747.169
White-naped Crane	-6176353.779	5918970.396
White-naped Crane	-6174355.594	5934378.544
White-naped Crane	-6171270.931	5936266.516
White-naped Crane	-6224554.006	5897192.937
White-naped Crane	-6174232.03	5934491.125
White-naped Crane	-6178380.907	5932517.085
White-naped Crane	-6177098.507	5934053.861
White-naped Crane	-6224578.496	5897223.804
White-naped Crane	-6187925.44	5948201.153
White-naped Crane	-6176349.326	5918975.282
White-naped Crane	-6173545.188	5931696.604
White-naped Crane	-6178448.812	5932768.301
White-naped Crane	-6224549.553	5897186.439
White-naped Crane	-6187931.006	5948214.227
White-naped Crane	-6177107.412	5934063.65
White-naped Crane	-6173530.717	5931714.546
White-naped Crane	-6178171.627	5933450.211
White-naped Crane	-6176350.44	5918999.713
White-naped Crane	-6205797.785	5939064.152
White-naped Crane	-6177040.62	5933903.76
White-naped Crane	-6224542.874	5897199.435
White-naped Crane	-6173557.434	5931701.498
White-naped Crane	-6176605.361	5918771.698
White-naped Crane	-6178111.514	5933466.526
White-naped Crane	-6173549.641	5931699.867
White-naped Crane	-6177017.243	5933830.343
White-naped Crane	-6224564.024	5897228.678
White-naped Crane	-6178199.456	5933226.709
White-naped Crane	-6177030.602	5933853.184
White-naped Crane	-6203389.944	5942948.725
White-naped Crane	-6173531.83	5931724.333
White-naped Crane	-6177072.903	5933849.921
White-naped Crane	-6176599.795	5918776.584
White-naped Crane	-6203365.454	5942868.697
White-naped Crane	-6173554.094	5931709.653
White-naped Crane	-6178732.677	5932861.286
White-naped Crane	-6203392.17	5942700.478
White-naped Crane	-6176579.758	5918776.584
White-naped Crane	-6177124.11	5933931.496
White-naped Crane	-6226254.967	5897538.982
White-naped Crane	-6173522.925	5931724.333
White-naped Crane	-6178700.394	5932802.559
White-naped Crane	-6177033.941	5933949.443
White-naped Crane	-6176589.777	5918747.268
White-naped Crane	-6200949.821	5938576.051
White-naped Crane	-6226309.514	5897423.632
White-naped Crane	-6178294.078	5932696.524
White-naped Crane	-6226283.91	5897566.601
White-naped Crane	-6177234.316	5934024.493
White-naped Crane	-6201619.964	5938148.373
White-naped Crane	-6178354.19	5932654.111
White-naped Crane	-6175845.049	5930996.892
White-naped Crane	-6177249.901	5934003.283
White-naped Crane	-6226288.363	5897564.977
White-naped Crane	-6201633.322	5938150.006
White-naped Crane	-6178351.964	5932659.005
White-naped Crane	-6226283.91	5897568.226

White-naped Crane	-6201628.87	5938150.006
White-naped Crane	-6175833.917	5930980.583
White-naped Crane	-6177254.354	5934003.283
White-naped Crane	-6178365.322	5932645.955
White-naped Crane	-6226257.194	5897564.977
White-naped Crane	-6201628.87	5938161.432
White-naped Crane	-6175845.049	5931016.464
White-naped Crane	-6178372.002	5932637.798
White-naped Crane	-6177245.448	5934000.02
White-naped Crane	-6226210.44	5897490.243
White-naped Crane	-6201631.096	5938156.535
White-naped Crane	-6185362.866	5929930.308
White-naped Crane	-6177243.222	5934003.283
White-naped Crane	-6201626.643	5938148.373
White-naped Crane	-6226199.308	5897498.366
White-naped Crane	-6175856.181	5931009.94
White-naped Crane	-6178363.096	5932644.324
White-naped Crane	-6226201.534	5897498.366
White-naped Crane	-6201617.738	5938150.006
White-naped Crane	-6177247.675	5934000.02
White-naped Crane	-6185349.507	5929917.262
White-naped Crane	-6175825.012	5930987.107
White-naped Crane	-6178367.549	5932645.955
White-naped Crane	-6185356.186	5929920.523
White-naped Crane	-6177252.128	5934001.652
White-naped Crane	-6226201.534	5897498.366
White-naped Crane	-6178358.643	5932649.217
White-naped Crane	-6201628.87	5938151.638
White-naped Crane	-6175836.144	5931001.785
White-naped Crane	-6185353.96	5929928.677
White-naped Crane	-6226295.042	5897584.473
White-naped Crane	-6177245.448	5933995.126
White-naped Crane	-6178365.322	5932637.798
White-naped Crane	-6201631.096	5938146.741
White-naped Crane	-6175829.464	5931000.154
White-naped Crane	-6178363.096	5932642.692
White-naped Crane	-6185398.488	5929933.569
White-naped Crane	-6176624.286	5918750.526
White-naped Crane	-6226297.269	5897581.223
White-naped Crane	-6177245.448	5934000.02
White-naped Crane	-6201631.096	5938150.006
White-naped Crane	-6175836.144	5930936.547
White-naped Crane	-6185333.923	5929914
White-naped Crane	-6176597.569	5918753.783
White-naped Crane	-6178149.363	5932654.111
White-naped Crane	-6177138.582	5934016.336
White-naped Crane	-6226312.853	5897503.24
White-naped Crane	-6175802.748	5930975.69
White-naped Crane	-6201624.417	5938148.373
White-naped Crane	-6223425.226	5895862.497
White-naped Crane	-6177252.128	5934045.703
White-naped Crane	-6176481.797	5918840.102
White-naped Crane	-6172806.027	5936978.081
White-naped Crane	-6201622.19	5938146.741
White-naped Crane	-6176481.797	5918833.587
White-naped Crane	-6177261.033	5933991.863
White-naped Crane	-6172193.77	5937802.329
White-naped Crane	-6201628.87	5938141.844

White-naped Crane	-6223474.206	5895868.995
White-naped Crane	-6137838.349	5940074.724
White-naped Crane	-6173870.241	5931406.274
White-naped Crane	-6177421.333	5933880.919
White-naped Crane	-6176488.476	5918838.473
White-naped Crane	-6172015.659	5937684.808
White-naped Crane	-6137851.707	5940082.887
White-naped Crane	-6223431.905	5895745.546
White-naped Crane	-6173861.336	5931409.536
White-naped Crane	-6177386.824	5933805.87
White-naped Crane	-6201623.304	5938136.947
White-naped Crane	-6176490.702	5918846.617
White-naped Crane	-6177343.409	5933800.976
White-naped Crane	-6172011.206	5937696.233
White-naped Crane	-6223545.451	5895756.916
White-naped Crane	-6173805.676	5931398.119
White-naped Crane	-6172094.696	5937559.127
White-naped Crane	-6223700.185	5895817.016
White-naped Crane	-6176558.607	5918939.451
White-naped Crane	-6177252.128	5934050.598
White-naped Crane	-6174262.086	5936398.706
White-naped Crane	-6223723.562	5895739.048
White-naped Crane	-6176564.173	5918947.595
White-naped Crane	-6173801.223	5931430.74
White-naped Crane	-6177574.954	5919708.225
White-naped Crane	-6224531.742	5897175.066
White-naped Crane	-6177501.483	5933918.444
White-naped Crane	-6173798.997	5936899.741
White-naped Crane	-6173754.469	5931411.167
White-naped Crane	-6174337.783	5936586.387
White-naped Crane	-6177577.18	5919704.968
White-naped Crane	-6224605.213	5897272.542
White-naped Crane	-6177240.996	5934050.598
White-naped Crane	-6176846.925	5934355.702
White-naped Crane	-6173770.054	5931450.312
White-naped Crane	-6174413.48	5936393.81
White-naped Crane	-6170808.955	5936261.621
White-naped Crane	-6176855.83	5934368.754
White-naped Crane	-6177572.728	5919704.968
White-naped Crane	-6223449.716	5895893.36
White-naped Crane	-6173674.319	5931460.099
White-naped Crane	-6174440.197	5936377.491
White-naped Crane	-6177572.728	5919693.566
White-naped Crane	-6170742.164	5936106.586
White-naped Crane	-6223687.94	5895864.122
White-naped Crane	-6176846.925	5934329.596
White-naped Crane	-6173727.752	5931463.361
White-naped Crane	-6171134.008	5936284.468
White-naped Crane	-6176935.98	5934300.227
White-naped Crane	-6176604.248	5918722.839
White-naped Crane	-6223607.79	5895862.497
White-naped Crane	-6174446.876	5936390.546
White-naped Crane	-6174458.008	5936356.275
White-naped Crane	-6171220.838	5936153.912
White-naped Crane	-6223443.037	5895877.117
White-naped Crane	-6176602.022	5918719.581
White-naped Crane	-6176947.112	5934306.754
White-naped Crane	-6173511.793	5931755.324

White-naped Crane	-6176586.437	5918678.865
White-naped Crane	-6223739.147	5895709.811
White-naped Crane	-6174571.554	5936349.747
White-naped Crane	-6175027.964	5934509.073
White-naped Crane	-6173567.452	5931681.925
White-naped Crane	-6174814.231	5936555.378
White-naped Crane	-6176357.119	5918968.768
White-naped Crane	-6173516.245	5931714.546
White-naped Crane	-6175094.756	5934125.649
White-naped Crane	-6174631.667	5936816.505
White-naped Crane	-6176564.173	5918708.181
White-naped Crane	-6223995.182	5897758.314
White-naped Crane	-6179661.081	5933360.483
White-naped Crane	-6175036.87	5931024.619
White-naped Crane	-6175110.341	5934122.386
White-naped Crane	-6174613.856	5936826.297
White-naped Crane	-6175112.567	5934128.912
White-naped Crane	-6179442.895	5933198.975
White-naped Crane	-6175764.899	5930863.154
White-naped Crane	-6176622.059	5918719.581
White-naped Crane	-6224471.629	5897269.293
White-naped Crane	-6174618.308	5936854.043
White-naped Crane	-6175112.567	5934133.807
White-naped Crane	-6179440.669	5933202.238
White-naped Crane	-6176622.059	5918721.21
White-naped Crane	-6175767.126	5930866.416
White-naped Crane	-6224464.95	5897266.044
White-naped Crane	-6174645.025	5936860.571
White-naped Crane	-6179445.121	5933203.869
White-naped Crane	-6224469.403	5897272.542
White-naped Crane	-6175117.02	5934135.438
White-naped Crane	-6175753.767	5930864.785
White-naped Crane	-6174613.856	5936839.354
White-naped Crane	-6176628.738	5918709.809
White-naped Crane	-6175288.452	5933924.97
White-naped Crane	-6224473.856	5897261.17
White-naped Crane	-6179449.574	5933194.081
White-naped Crane	-6175760.446	5930859.893
White-naped Crane	-6174604.95	5936827.929
White-naped Crane	-6175281.773	5933934.759
White-naped Crane	-6176626.512	5918709.809
White-naped Crane	-6224516.157	5897173.442
White-naped Crane	-6179440.669	5933200.606
White-naped Crane	-6175749.314	5930869.678
White-naped Crane	-6174587.139	5936844.25
White-naped Crane	-6176626.512	5918716.324
White-naped Crane	-6179436.216	5933203.869
White-naped Crane	-6224480.535	5897223.804
White-naped Crane	-6175292.904	5933941.285
White-naped Crane	-6175758.22	5930846.845
White-naped Crane	-6174598.271	5936839.354
White-naped Crane	-6176626.512	5918722.839
White-naped Crane	-6179440.669	5933202.238
White-naped Crane	-6175281.773	5933936.391
White-naped Crane	-6224491.667	5897214.057
White-naped Crane	-6174620.535	5936842.618
White-naped Crane	-6176624.286	5918717.953
White-naped Crane	-6179442.895	5933203.869

White-naped Crane	-6224522.836	5897183.189
White-naped Crane	-6175290.678	5933942.917
White-naped Crane	-6175747.088	5930872.94
White-naped Crane	-6174611.629	5936836.09
White-naped Crane	-6179445.121	5933202.238
White-naped Crane	-6224520.61	5897170.193
White-naped Crane	-6176624.286	5918724.467
White-naped Crane	-6175290.678	5933938.022
White-naped Crane	-6175749.314	5930872.94
White-naped Crane	-6174616.082	5936831.194
White-naped Crane	-6179162.37	5933220.183
White-naped Crane	-6176626.512	5918719.581
White-naped Crane	-6226361.834	5900189.206
White-naped Crane	-6170679.825	5936597.811
White-naped Crane	-6173658.734	5930998.523
White-naped Crane	-6174618.308	5936836.09
White-naped Crane	-6176606.474	5918729.353
White-naped Crane	-6178485.547	5934109.334
White-naped Crane	-6226319.533	5900036.442
White-naped Crane	-6173522.925	5931057.238
White-naped Crane	-6170677.598	5936547.218
White-naped Crane	-6177501.483	5933838.5
White-naped Crane	-6174555.969	5936927.486
White-naped Crane	-6226250.515	5897529.234
White-naped Crane	-6176597.569	5918726.096
White-naped Crane	-6170232.32	5936849.146
White-naped Crane	-6173518.472	5931063.762
White-naped Crane	-6174551.517	5936924.222
White-naped Crane	-6176403.873	5919001.341
White-naped Crane	-6178345.285	5933396.374
White-naped Crane	-6170216.736	5936873.627
White-naped Crane	-6226257.194	5897537.358
White-naped Crane	-6173445.001	5931032.774
White-naped Crane	-6178405.397	5933234.866
White-naped Crane	-6172253.882	5945510.006
White-naped Crane	-6174558.196	5936942.175
White-naped Crane	-6226286.137	5897498.366
White-naped Crane	-6178717.092	5932970.585
White-naped Crane	-6170911.369	5936516.21
White-naped Crane	-6176408.326	5919006.228
White-naped Crane	-6173378.209	5931331.247
White-naped Crane	-6174580.46	5934179.491
White-naped Crane	-6226293.929	5897436.629
White-naped Crane	-6173357.059	5931318.198
White-naped Crane	-6176421.684	5919009.485
White-naped Crane	-6172231.618	5945470.797
White-naped Crane	-6174535.932	5936901.373
White-naped Crane	-6174604.95	5934151.754
White-naped Crane	-6176421.684	5919007.856
White-naped Crane	-6178774.978	5932851.498
White-naped Crane	-6173349.266	5931319.829
White-naped Crane	-6174544.837	5936934.015
White-naped Crane	-6177252.128	5934040.809
White-naped Crane	-6174527.026	5934182.754
White-naped Crane	-6226234.93	5897517.862
White-naped Crane	-6173331.455	5931319.829
White-naped Crane	-6172238.298	5945475.698
White-naped Crane	-6174542.611	5936891.58

White-naped Crane	-6172334.032	5945477.332
White-naped Crane	-6177122.997	5934143.596
White-naped Crane	-6226268.326	5897534.108
White-naped Crane	-6173371.53	5931329.616
White-naped Crane	-6174021.636	5939958.804
White-naped Crane	-6191405.288	5988971.88
White-naped Crane	-6225807.463	5898128.753
White-naped Crane	-6172365.202	5945459.361
White-naped Crane	-6173333.681	5931342.664
White-naped Crane	-6177042.847	5934166.438
White-naped Crane	-6177951.214	5932774.827
White-naped Crane	-6192135.543	5988929.188
White-naped Crane	-6177022.809	5933936.391
White-naped Crane	-6226050.139	5898432.59
White-naped Crane	-6174820.91	5940551.48
White-naped Crane	-6172371.881	5945464.262
White-naped Crane	-6173358.172	5931376.915
White-naped Crane	-6177786.461	5932600.279
White-naped Crane	-6174295.482	5943040.186
White-naped Crane	-6192151.128	5988935.756
White-naped Crane	-6177082.922	5933862.973
White-naped Crane	-6225985.574	5898257.111
White-naped Crane	-6173353.719	5931331.247
White-naped Crane	-6172044.602	5944663.784
White-naped Crane	-6177069.564	5933866.236
White-naped Crane	-6178792.789	5932110.913
White-naped Crane	-6192115.506	5988940.682
White-naped Crane	-6174627.214	5944226.003
White-naped Crane	-6191240.535	5989141.007
White-naped Crane	-6178872.939	5932559.498
White-naped Crane	-6215200.942	5941856.165
White-naped Crane	-6173485.076	5931282.316
White-naped Crane	-6171712.87	5944034.889
White-naped Crane	-6191937.395	5989067.116
White-naped Crane	-6178728.224	5932843.341
White-naped Crane	-6204549.893	5949240.582
White-naped Crane	-6171009.33	5937351.839
White-naped Crane	-6191169.29	5988916.052
White-naped Crane	-6178959.769	5932892.281
White-naped Crane	-6204812.607	5948277.962
White-naped Crane	-6172645.727	5945225.745
White-naped Crane	-6172627.916	5945224.111
White-naped Crane	-6191229.403	5988943.966
White-naped Crane	-6178162.721	5933926.602
White-naped Crane	-6178772.752	5932849.867
White-naped Crane	-6204935.058	5947844.899
White-naped Crane	-6174640.572	5944337.08
White-naped Crane	-6175733.73	5931011.571
White-naped Crane	-6172639.048	5945230.646
White-naped Crane	-6174640.572	5944324.012
White-naped Crane	-6178162.721	5933923.339
White-naped Crane	-6204937.285	5947848.168
White-naped Crane	-6191233.856	5988962.028
White-naped Crane	-6175738.182	5931008.309
White-naped Crane	-6178779.431	5932861.286
White-naped Crane	-6191229.403	5988953.818
White-naped Crane	-6174651.704	5944343.614
White-naped Crane	-6178164.947	5933913.549

White-naped Crane	-6204941.738	5947864.509
White-naped Crane	-6175724.824	5930996.892
White-naped Crane	-6178783.884	5932871.074
White-naped Crane	-6174616.082	5944309.311
White-naped Crane	-6191229.403	5988943.966
White-naped Crane	-6172683.576	5945204.507
White-naped Crane	-6178169.4	5933910.286
White-naped Crane	-6204928.379	5947812.216
White-naped Crane	-6178786.11	5932856.392
White-naped Crane	-6175740.409	5931013.202
White-naped Crane	-6172676.896	5945204.507
White-naped Crane	-6174607.176	5944294.609
White-naped Crane	-6178173.853	5933915.181
White-naped Crane	-6191229.403	5988947.25
White-naped Crane	-6178783.884	5932856.392
White-naped Crane	-6204928.379	5947624.291
White-naped Crane	-6175722.598	5931003.416
White-naped Crane	-6174618.308	5944337.08
White-naped Crane	-6191229.403	5988948.892
White-naped Crane	-6178164.947	5933916.812
White-naped Crane	-6178786.11	5932861.286
White-naped Crane	-6204937.285	5947625.925
White-naped Crane	-6175731.503	5931005.047
White-naped Crane	-6172665.764	5945229.012
White-naped Crane	-6178167.174	5933916.812
White-naped Crane	-6191222.724	5988940.682
White-naped Crane	-6178786.11	5932862.917
White-naped Crane	-6204930.606	5947632.462
White-naped Crane	-6174631.667	5944302.777
White-naped Crane	-6175735.956	5931009.94
White-naped Crane	-6174611.629	5944319.112
White-naped Crane	-6178169.4	5933916.812
White-naped Crane	-6191218.271	5988937.398
White-naped Crane	-6204930.606	5947630.827
White-naped Crane	-6178786.11	5932862.917
White-naped Crane	-6172679.123	5945202.874
White-naped Crane	-6175738.182	5931006.678
White-naped Crane	-6172676.896	5945199.606
White-naped Crane	-6174631.667	5944312.578
White-naped Crane	-6191222.724	5988945.608
White-naped Crane	-6178167.174	5933918.444
White-naped Crane	-6178714.866	5932858.023
White-naped Crane	-6204536.535	5946839.952
White-naped Crane	-6175731.503	5931006.678
White-naped Crane	-6174747.439	5944217.836
White-naped Crane	-6191222.724	5988939.04
White-naped Crane	-6178193.89	5934155.017
White-naped Crane	-6170764.428	5937619.519
White-naped Crane	-6199520.478	5951058.251
White-naped Crane	-6172527.728	5944993.768
White-naped Crane	-6173404.926	5931479.671
White-naped Crane	-6174740.76	5944212.936
White-naped Crane	-6191783.774	5988917.694
White-naped Crane	-6170715.447	5937573.817
White-naped Crane	-6172527.728	5944970.898
White-naped Crane	-6173391.568	5931458.468
White-naped Crane	-6196042.858	5946652.048
White-naped Crane	-6170724.353	5936217.558

White-naped Crane	-6170773.333	5937518.322
White-naped Crane	-6174742.986	5944222.736
White-naped Crane	-6173042.024	5946371.016
White-naped Crane	-6191966.338	5988705.88
White-naped Crane	-6173095.458	5946446.175
White-naped Crane	-6174827.589	5944333.813
White-naped Crane	-6178396.492	5932864.549
White-naped Crane	-6191984.149	5988687.819
White-naped Crane	-6193591.602	5944604.977
White-naped Crane	-6174669.515	5933822.185
White-naped Crane	-6173449.454	5931434.002
White-naped Crane	-6174799.759	5944307.677
White-naped Crane	-6192062.073	5988612.29
White-naped Crane	-6178396.492	5932871.074
White-naped Crane	-6173035.345	5946480.487
White-naped Crane	-6173433.869	5931509.03
White-naped Crane	-6173449.454	5931425.847
White-naped Crane	-6174801.986	5944306.044
White-naped Crane	-6178369.775	5932791.14
White-naped Crane	-6173025.326	5946483.755
White-naped Crane	-6173436.095	5931464.992
White-naped Crane	-6194096.993	5944459.593
White-naped Crane	-6173020.874	5946488.656
White-naped Crane	-6174816.457	5944412.221
White-naped Crane	-6175025.738	5933766.714
White-naped Crane	-6178287.399	5932693.262
White-naped Crane	-6192229.052	5988653.338
White-naped Crane	-6174842.061	5944456.326
White-naped Crane	-6192351.503	5988817.533
White-naped Crane	-6173024.213	5946482.121
White-naped Crane	-6178768.299	5932858.023
White-naped Crane	-6175604.599	5933722.664
White-naped Crane	-6173445.001	5931491.089
White-naped Crane	-6172980.799	5946472.317
White-naped Crane	-6174860.985	5944459.593
White-naped Crane	-6178694.828	5932771.564
White-naped Crane	-6192353.73	5988843.805
White-naped Crane	-6177062.884	5933866.236
White-naped Crane	-6192215.693	5943646.141
White-naped Crane	-6173442.775	5931438.895
White-naped Crane	-6174823.136	5944451.425
White-naped Crane	-6177229.864	5934022.862
White-naped Crane	-6173000.836	5946475.585
White-naped Crane	-6193894.391	5944632.747
White-naped Crane	-6192320.334	5988835.595
White-naped Crane	-6173391.568	5931349.188
White-naped Crane	-6174817.57	5944451.425
White-naped Crane	-6192331.466	5988881.57
White-naped Crane	-6178249.55	5932593.754
White-naped Crane	-6177243.222	5934008.178
White-naped Crane	-6173005.289	5946464.148
White-naped Crane	-6196552.701	5942089.69
White-naped Crane	-6173344.813	5931316.567
White-naped Crane	-6173003.063	5946455.978
White-naped Crane	-6174819.797	5944451.425
White-naped Crane	-6192322.56	5988879.928
White-naped Crane	-6177138.582	5934009.809
White-naped Crane	-6197265.146	5942037.432

White-naped Crane	-6178058.081	5932747.094
White-naped Crane	-6173367.077	5931334.509
White-naped Crane	-6171863.151	5944255.406
White-naped Crane	-6174815.344	5944461.226
White-naped Crane	-6177031.715	5933983.705
White-naped Crane	-6177977.931	5933088.042
White-naped Crane	-6192253.542	5988139.427
White-naped Crane	-6197372.012	5941848
White-naped Crane	-6173369.304	5931314.936
White-naped Crane	-6174889.928	5944513.499
White-naped Crane	-6197490.011	5941772.881
White-naped Crane	-6192262.448	5988183.757
White-naped Crane	-6171510.268	5937244.117
White-naped Crane	-6171692.832	5943809.479
White-naped Crane	-6174146.314	5931385.071
White-naped Crane	-6174866.551	5944485.729
White-naped Crane	-6171621.588	5937299.61
White-naped Crane	-6177272.165	5933985.336
White-naped Crane	-6197349.748	5941813.706
White-naped Crane	-6172745.915	5945124.459
White-naped Crane	-6192244.637	5988157.488
White-naped Crane	-6175842.823	5931314.936
White-naped Crane	-6174850.966	5944482.462
White-naped Crane	-6177450.276	5933799.344
White-naped Crane	-6197572.387	5941560.593
White-naped Crane	-6192162.26	5988764.991
White-naped Crane	-6172620.124	5945171.834
White-naped Crane	-6175321.848	5934194.175
White-naped Crane	-6175849.502	5931252.958
White-naped Crane	-6178265.135	5933342.538
White-naped Crane	-6192166.713	5988771.558
White-naped Crane	-6197592.425	5941565.492
White-naped Crane	-6175326.3	5934192.543
White-naped Crane	-6172634.595	5945181.636
White-naped Crane	-6174845.4	5944469.394
White-naped Crane	-6175849.502	5931266.006
White-naped Crane	-6174856.532	5944474.294
White-naped Crane	-6175332.979	5934202.333
White-naped Crane	-6178265.135	5933347.432
White-naped Crane	-6192166.713	5988769.916
White-naped Crane	-6197594.651	5941570.391
White-naped Crane	-6172666.878	5945152.231
White-naped Crane	-6175840.596	5931288.84
White-naped Crane	-6172680.236	5945142.429
White-naped Crane	-6175332.979	5934190.912
White-naped Crane	-6178267.361	5933342.538
White-naped Crane	-6174955.606	5944515.132
White-naped Crane	-6192164.486	5988766.633
White-naped Crane	-6197576.84	5941576.923
White-naped Crane	-6175809.427	5931248.065
White-naped Crane	-6178265.135	5933342.538
White-naped Crane	-6175324.074	5934190.912
White-naped Crane	-6197561.255	5941572.024
White-naped Crane	-6192204.562	5988764.991
White-naped Crane	-6172667.991	5945144.062
White-naped Crane	-6175802.748	5931230.123
White-naped Crane	-6174947.814	5944526.567
White-naped Crane	-6172672.444	5945135.894

White-naped Crane	-6174935.569	5944524.933
White-naped Crane	-6175319.621	5934200.701
White-naped Crane	-6178302.984	5933198.975
White-naped Crane	-6192206.788	5988774.842
White-naped Crane	-6175789.389	5931246.434
White-naped Crane	-6197532.312	5941496.908
White-naped Crane	-6172669.104	5945142.429
White-naped Crane	-6174935.569	5944533.101
White-naped Crane	-6192204.562	5988768.274
White-naped Crane	-6178302.984	5933205.501
White-naped Crane	-6197532.312	5941488.743
White-naped Crane	-6175321.848	5934199.069
White-naped Crane	-6175811.653	5931243.172
White-naped Crane	-6178302.984	5933205.501
White-naped Crane	-6174942.248	5944518.399
White-naped Crane	-6175317.395	5934197.438
White-naped Crane	-6192202.335	5988763.349
White-naped Crane	-6197541.218	5941501.807
White-naped Crane	-6172666.878	5945144.062
White-naped Crane	-6175818.332	5931249.696
White-naped Crane	-6178302.984	5933202.238
White-naped Crane	-6174915.531	5944498.797
White-naped Crane	-6192204.562	5988769.916
White-naped Crane	-6175312.942	5934190.912
White-naped Crane	-6197543.444	5941480.578
White-naped Crane	-6172665.764	5945137.528
White-naped Crane	-6175858.407	5931243.172
White-naped Crane	-6174878.796	5944484.095
White-naped Crane	-6192204.562	5988764.991
White-naped Crane	-6170797.823	5936273.044
White-naped Crane	-6201023.292	5937888.839
White-naped Crane	-6173525.151	5931952.69
White-naped Crane	-6169945.116	5939831.457
White-naped Crane	-6172675.783	5945106.489
White-naped Crane	-6172672.444	5945085.251
White-naped Crane	-6174840.947	5944451.425
White-naped Crane	-6192204.562	5988766.633
White-naped Crane	-6170791.144	5936255.093
White-naped Crane	-6201007.707	5937859.458
White-naped Crane	-6171926.603	5936770.807
White-naped Crane	-6173493.982	5931944.535
White-naped Crane	-6192082.11	5988264.208
White-naped Crane	-6170938.086	5936328.531
White-naped Crane	-6203944.315	5934110.965
White-naped Crane	-6172761.499	5945068.915
White-naped Crane	-6173969.316	5948555.787
White-naped Crane	-6192077.657	5988262.566
White-naped Crane	-6178768.299	5932861.286
White-naped Crane	-6175591.241	5932561.129
White-naped Crane	-6172762.612	5945060.747
White-naped Crane	-6204253.783	5933572.569
White-naped Crane	-6191155.932	5988871.719
White-naped Crane	-6174189.728	5950337.349
White-naped Crane	-6178770.525	5932861.286
White-naped Crane	-6173137.759	5931846.667
White-naped Crane	-6178766.073	5933079.885
White-naped Crane	-6174233.143	5950728.034
White-naped Crane	-6227546.273	5904905.069

White-naped Crane	-6187146.204	5914447.074
White-naped Crane	-6172752.594	5945060.747
White-naped Crane	-6191135.894	5988810.966
White-naped Crane	-6191131.442	5988804.398
White-naped Crane	-6178144.91	5932368.642
White-naped Crane	-6174205.313	5950597.259
White-naped Crane	-6227782.271	5904784.742
White-naped Crane	-6172708.066	5945454.46
White-naped Crane	-6185750.257	5912331.143
White-naped Crane	-6172709.179	5945441.391
White-naped Crane	-6174321.085	5950886.602
White-naped Crane	-6191133.668	5988814.249
White-naped Crane	-6178053.628	5932539.923
White-naped Crane	-6227875.779	5904757.1
White-naped Crane	-6185745.805	5912313.241
White-naped Crane	-6172715.858	5945408.717
White-naped Crane	-6175193.83	5951430.986
White-naped Crane	-6178164.947	5932075.027
White-naped Crane	-6185756.937	5912277.437
White-naped Crane	-6191124.762	5988852.015
White-naped Crane	-6178452.152	5932244.67
White-naped Crane	-6172712.519	5945398.915
White-naped Crane	-6185759.163	5912269.3
White-naped Crane	-6191138.121	5988825.743
White-naped Crane	-6175249.49	5951622.264
White-naped Crane	-6226544.398	5900346.847
White-naped Crane	-6191138.121	5988825.743
White-naped Crane	-6178336.379	5932443.679
White-naped Crane	-6185748.031	5912303.476
White-naped Crane	-6172689.142	5945400.548
White-naped Crane	-6175155.982	5951761.229
White-naped Crane	-6226564.436	5900374.475
White-naped Crane	-6175093.643	5951821.721
White-naped Crane	-6191135.894	5988825.743
White-naped Crane	-6171673.908	5943914.016
White-naped Crane	-6185754.71	5912301.849
White-naped Crane	-6178365.322	5932942.852
White-naped Crane	-6226504.323	5900265.588
White-naped Crane	-6178140.457	5933138.614
White-naped Crane	-6192104.374	5989242.814
White-naped Crane	-6186580.701	5912744.522
White-naped Crane	-6171648.304	5943900.949
White-naped Crane	-6175128.152	5951800.467
White-naped Crane	-6226428.626	5900168.079
White-naped Crane	-6226344.023	5897629.964
White-naped Crane	-6175101.435	5951821.721
White-naped Crane	-6192099.921	5989257.592
White-naped Crane	-6191247.214	5911722.501
White-naped Crane	-6177904.46	5933277.282
White-naped Crane	-6171604.89	5943842.146
White-naped Crane	-6175291.791	5951723.627
White-naped Crane	-6172841.649	5945070.549
White-naped Crane	-6178160.495	5933435.529
White-naped Crane	-6192086.563	5988989.942
White-naped Crane	-6192039.809	5912228.615
White-naped Crane	-6172678.01	5945268.22
White-naped Crane	-6192171.166	5989141.007
White-naped Crane	-6178151.589	5933422.477

White-naped Crane	-6226266.099	5897569.851
White-naped Crane	-6192019.771	5912215.595
White-naped Crane	-6175271.754	5951754.69
White-naped Crane	-6175272.867	5951753.055
White-naped Crane	-6172683.576	5945295.993
White-naped Crane	-6192024.224	5912225.36
White-naped Crane	-6192171.166	5989141.007
White-naped Crane	-6226270.552	5897581.223
White-naped Crane	-6178527.849	5932983.636
White-naped Crane	-6175262.848	5951736.706
White-naped Crane	-6191999.734	5912217.223
White-naped Crane	-6192171.166	5989136.081
White-naped Crane	-6172685.802	5945269.854
White-naped Crane	-6178534.528	5932985.267
White-naped Crane	-6226275.005	5897569.851
White-naped Crane	-6175245.037	5951731.801
White-naped Crane	-6178538.981	5932980.373
White-naped Crane	-6192006.413	5912210.713
White-naped Crane	-6172678.01	5945268.22
White-naped Crane	-6192173.392	5989142.649
White-naped Crane	-6226188.176	5897514.612
White-naped Crane	-6172676.896	5945268.22
White-naped Crane	-6175269.527	5951748.15
White-naped Crane	-6192166.713	5989137.723
White-naped Crane	-6178552.339	5933004.843
White-naped Crane	-6192006.413	5912217.223
White-naped Crane	-6192166.713	5989142.649
White-naped Crane	-6178538.981	5933001.58
White-naped Crane	-6175265.075	5951753.055
White-naped Crane	-6172674.67	5945268.22
White-naped Crane	-6191997.507	5912222.105
White-naped Crane	-6226177.044	5897522.736
White-naped Crane	-6172679.123	5945264.953
White-naped Crane	-6175247.263	5951746.515
White-naped Crane	-6178541.207	5933006.474
White-naped Crane	-6226179.27	5897521.111
White-naped Crane	-6192171.166	5989132.797
White-naped Crane	-6191999.734	5912215.595
White-naped Crane	-6172690.255	5945256.784
White-naped Crane	-6178541.207	5933003.212
White-naped Crane	-6192171.166	5989139.365
White-naped Crane	-6226230.477	5897460.999
White-naped Crane	-6175262.848	5951749.785
White-naped Crane	-6192004.186	5912215.595
White-naped Crane	-6175302.923	5951743.245
White-naped Crane	-6178543.434	5932991.792
White-naped Crane	-6226230.477	5897467.498
White-naped Crane	-6192004.186	5912207.458
White-naped Crane	-6192171.166	5989131.155
White-naped Crane	-6175053.568	5951834.8
White-naped Crane	-6170860.162	5936457.458
White-naped Crane	-6192197.882	5989070.4
White-naped Crane	-6226292.816	5897407.386
White-naped Crane	-6172739.235	5945278.022
White-naped Crane	-6172279.486	5945047.678
White-naped Crane	-6174905.513	5952029.357
White-naped Crane	-6192200.109	5989078.61
White-naped Crane	-6191233.856	5911296.152

White-naped Crane	-6170797.823	5936243.669
White-naped Crane	-6226315.08	5897493.492
White-naped Crane	-6174897.72	5951954.15
White-naped Crane	-6192204.562	5989081.894
White-naped Crane	-6170835.672	5936230.613
White-naped Crane	-6226246.062	5897519.486
White-naped Crane	-6173160.023	5946109.6
White-naped Crane	-6190554.807	5910064.415
White-naped Crane	-6178238.418	5933239.76
White-naped Crane	-6189639.76	5910825.891
White-naped Crane	-6192202.335	5989083.536
White-naped Crane	-6173400.473	5947451.077
White-naped Crane	-6175137.057	5951726.896
White-naped Crane	-6223696.845	5895859.249
White-naped Crane	-6173423.85	5947485.392
White-naped Crane	-6192206.788	5989080.252
White-naped Crane	-6226330.665	5897576.349
White-naped Crane	-6178092.59	5956665.693
White-naped Crane	-6189840.136	5911636.254
White-naped Crane	-6172994.157	5946506.629
White-naped Crane	-6175056.907	5953133.02
White-naped Crane	-6178383.134	5932544.816
White-naped Crane	-6192206.788	5989070.4
White-naped Crane	-6226321.759	5897566.601
White-naped Crane	-6189802.287	5911761.557
White-naped Crane	-6174580.46	5952053.881
White-naped Crane	-6178750.488	5932947.746
White-naped Crane	-6191723.661	5989050.696
White-naped Crane	-6189987.077	5911509.324
White-naped Crane	-6172946.29	5946521.334
White-naped Crane	-6226292.816	5897506.489
White-naped Crane	-6174453.556	5951820.086
White-naped Crane	-6192213.467	5989127.871
White-naped Crane	-6226283.91	5897504.864
White-naped Crane	-6178904.109	5932765.039
White-naped Crane	-6172946.29	5946519.7
White-naped Crane	-6195112.227	5913900.138
White-naped Crane	-6179010.975	5932689.999
White-naped Crane	-6174259.86	5951735.071
White-naped Crane	-6195003.134	5913882.233
White-naped Crane	-6192217.92	5989131.155
White-naped Crane	-6172940.724	5946531.138
White-naped Crane	-6226275.005	5897509.738
White-naped Crane	-6173065.401	5950981.417
White-naped Crane	-6192211.241	5989144.291
White-naped Crane	-6179042.145	5932732.413
White-naped Crane	-6172931.818	5946524.602
White-naped Crane	-6194994.228	5913878.977
White-naped Crane	-6226275.005	5897495.117
White-naped Crane	-6172745.915	5945776.306
White-naped Crane	-6173113.269	5951027.19
White-naped Crane	-6178930.825	5932528.504
White-naped Crane	-6226277.231	5897491.867
White-naped Crane	-6192211.241	5989147.575
White-naped Crane	-6173699.923	5951283.852
White-naped Crane	-6178759.393	5932628.011
White-naped Crane	-6172743.688	5945691.35
White-naped Crane	-6192211.241	5989142.649

White-naped Crane	-6196165.309	5914282.664
White-naped Crane	-6226479.833	5899041.922
White-naped Crane	-6172737.009	5945692.984
White-naped Crane	-6178710.413	5932796.033
White-naped Crane	-6192213.467	5989129.513
White-naped Crane	-6225270.903	5899007.798
White-naped Crane	-6174107.352	5951712.182
White-naped Crane	-6191162.611	5989047.412
White-naped Crane	-6178717.092	5932982.004
White-naped Crane	-6190926.614	5912200.948
White-naped Crane	-6225224.149	5899009.423
White-naped Crane	-6172612.331	5944889.218
White-naped Crane	-6175002.361	5951831.53
White-naped Crane	-6172133.657	5943579.174
White-naped Crane	-6178576.829	5933353.958
White-naped Crane	-6189793.381	5911561.398
White-naped Crane	-6225299.846	5899030.547
White-naped Crane	-6191171.517	5989037.56
White-naped Crane	-6175046.888	5951864.228
White-naped Crane	-6174275.444	5945243.715
White-naped Crane	-6189786.702	5911545.125
White-naped Crane	-6191171.517	5989029.35
White-naped Crane	-6178547.886	5933006.474
White-naped Crane	-6225275.356	5899002.923
White-naped Crane	-6175012.379	5951828.26
White-naped Crane	-6191173.743	5989022.782
White-naped Crane	-6189784.476	5911525.597
White-naped Crane	-6225282.035	5899006.173
White-naped Crane	-6178550.113	5933004.843
White-naped Crane	-6174267.652	5945230.646
White-naped Crane	-6174276.558	5945232.28
White-naped Crane	-6175041.322	5951802.102
White-naped Crane	-6178554.566	5933008.106
White-naped Crane	-6189786.702	5911532.106
White-naped Crane	-6191175.969	5989029.35
White-naped Crane	-6225282.035	5899001.298
White-naped Crane	-6175012.379	5951759.594
White-naped Crane	-6178550.113	5933001.58
White-naped Crane	-6191171.517	5989027.708
White-naped Crane	-6189784.476	5911530.479
White-naped Crane	-6174273.218	5945233.913
White-naped Crane	-6175054.681	5951818.451
White-naped Crane	-6189786.702	5911527.224
White-naped Crane	-6191193.781	5989012.93
White-naped Crane	-6178547.886	5933003.212
White-naped Crane	-6174270.992	5945230.646
White-naped Crane	-6225288.714	5898960.675
White-naped Crane	-6174267.652	5945235.547
White-naped Crane	-6175034.643	5951811.911
White-naped Crane	-6178550.113	5932999.949
White-naped Crane	-6189780.023	5911538.615
White-naped Crane	-6191191.554	5989003.078
White-naped Crane	-6225279.809	5898972.049
White-naped Crane	-6174474.706	5944913.722
White-naped Crane	-6175033.53	5951813.546
White-naped Crane	-6189784.476	5911530.479
White-naped Crane	-6178538.981	5933003.212
White-naped Crane	-6191191.554	5989016.214

White-naped Crane	-6225288.714	5898955.8
White-naped Crane	-6175015.719	5951798.832
White-naped Crane	-6178538.981	5933001.58
White-naped Crane	-6191196.007	5989014.572
White-naped Crane	-6225288.714	5898954.175
White-naped Crane	-6189786.702	5911532.106
White-naped Crane	-6174494.744	5944882.683
White-naped Crane	-6174510.328	5944867.981
White-naped Crane	-6175029.077	5951802.102
White-naped Crane	-6191196.007	5989016.214
White-naped Crane	-6189782.249	5911548.379
White-naped Crane	-6178536.754	5932998.318
White-naped Crane	-6225284.261	5898985.049
White-naped Crane	-6191966.338	5988707.522
White-naped Crane	-6178599.093	5933420.846
White-naped Crane	-6174657.27	5951857.689
White-naped Crane	-6186941.376	5912645.244
White-naped Crane	-6171590.418	5943802.945
White-naped Crane	-6171580.399	5943856.847
White-naped Crane	-6182435.163	5915168.237
White-naped Crane	-6191964.111	5988702.596
White-naped Crane	-6174394.556	5951574.853
White-naped Crane	-6170777.786	5936255.093
White-naped Crane	-6225179.621	5898611.323
White-naped Crane	-6173196.758	5944204.768
White-naped Crane	-6170864.615	5936253.461
White-naped Crane	-6191966.338	5988712.448
White-naped Crane	-6174155.219	5951551.964
White-naped Crane	-6225368.864	5898362.723
White-naped Crane	-6170826.767	5936251.829
White-naped Crane	-6191966.338	5988712.448
White-naped Crane	-6174176.37	5951529.076
White-naped Crane	-6225482.41	5898073.511
White-naped Crane	-6170751.069	5936276.308
White-naped Crane	-6174095.107	5945343.369
White-naped Crane	-6191532.192	5988671.399
White-naped Crane	-6174304.387	5951769.404
White-naped Crane	-6178227.286	5933287.07
White-naped Crane	-6173569.679	5935599.07
White-naped Crane	-6174112.918	5945292.725
White-naped Crane	-6226308.401	5897545.481
White-naped Crane	-6174523.687	5951887.117
White-naped Crane	-6178149.363	5933187.555
White-naped Crane	-6226283.91	5897506.489
White-naped Crane	-6173741.111	5934182.754
White-naped Crane	-6191961.885	5988699.313
White-naped Crane	-6174127.389	5945269.854
White-naped Crane	-6174419.046	5951854.419
White-naped Crane	-6179331.576	5932081.552
White-naped Crane	-6177281.071	5934047.335
White-naped Crane	-6174126.276	5945271.487
White-naped Crane	-6226277.231	5897548.73
White-naped Crane	-6191966.338	5988702.596
White-naped Crane	-6174433.518	5951787.388
White-naped Crane	-6191961.885	5988700.954
White-naped Crane	-6178944.184	5932171.267
White-naped Crane	-6226283.91	5897486.993
White-naped Crane	-6177240.996	5934037.546

White-naped Crane	-6191293.968	5989323.275
White-naped Crane	-6178881.845	5932375.167
White-naped Crane	-6174386.764	5951792.292
White-naped Crane	-6226292.816	5897415.509
White-naped Crane	-6177214.279	5934029.388
White-naped Crane	-6174126.276	5945281.289
White-naped Crane	-6174393.443	5951803.737
White-naped Crane	-6191302.874	5989313.422
White-naped Crane	-6226270.552	5897547.106
White-naped Crane	-6178289.625	5932830.291
White-naped Crane	-6174126.276	5945276.388
White-naped Crane	-6177033.941	5933983.705
White-naped Crane	-6174130.729	5945278.022
White-naped Crane	-6178089.25	5933114.144
White-naped Crane	-6174384.537	5951787.388
White-naped Crane	-6177227.637	5934110.965
White-naped Crane	-6191912.904	5989316.706
White-naped Crane	-6226319.533	5897521.111
White-naped Crane	-6174380.085	5951784.118
White-naped Crane	-6178149.363	5933429.003
White-naped Crane	-6191890.641	5989257.592
White-naped Crane	-6226317.306	5897517.862
White-naped Crane	-6172000.074	5943530.173
White-naped Crane	-6174391.217	5935767.151
White-naped Crane	-6172073.545	5943569.373
White-naped Crane	-6173375.983	5951775.943
White-naped Crane	-6178156.042	5933432.266
White-naped Crane	-6191890.641	5989242.814
White-naped Crane	-6226272.778	5897522.736
White-naped Crane	-6171516.947	5938117.359
White-naped Crane	-6178133.778	5933872.762
White-naped Crane	-6225393.355	5898188.87
White-naped Crane	-6171619.361	5937508.529
White-naped Crane	-6191890.641	5989252.666
White-naped Crane	-6174159.672	5935705.14
White-naped Crane	-6191908.452	5989242.814
White-naped Crane	-6172830.517	5945291.092
White-naped Crane	-6178792.789	5932897.175
White-naped Crane	-6224767.739	5898404.968
White-naped Crane	-6172924.026	5945418.519
White-naped Crane	-6178795.016	5932859.655
White-naped Crane	-6175016.832	5951800.467
White-naped Crane	-6174567.101	5935819.371
White-naped Crane	-6191910.678	5989249.382
White-naped Crane	-6225413.392	5898320.478
White-naped Crane	-6175010.153	5951797.197
White-naped Crane	-6178797.242	5932862.917
White-naped Crane	-6174987.889	5935928.707
White-naped Crane	-6191915.131	5989251.024
White-naped Crane	-6172921.799	5945451.193
White-naped Crane	-6225420.071	5898322.103
White-naped Crane	-6175025.738	5951834.8
White-naped Crane	-6191912.904	5989252.666
White-naped Crane	-6175001.247	5935925.443
White-naped Crane	-6225415.618	5898326.977
White-naped Crane	-6175021.285	5951826.625
White-naped Crane	-6175001.247	5935931.971
White-naped Crane	-6191906.225	5989255.95

White-naped Crane	-6178795.016	5932895.544
White-naped Crane	-6172919.573	5945444.658
White-naped Crane	-6225420.071	5898323.728
White-naped Crane	-6172957.422	5945472.431
White-naped Crane	-6175019.059	5951824.99
White-naped Crane	-6174999.021	5935925.443
White-naped Crane	-6191912.904	5989269.087
White-naped Crane	-6225413.392	5898323.728
White-naped Crane	-6178795.016	5932897.175
White-naped Crane	-6175001.247	5935927.075
White-naped Crane	-6191910.678	5989278.939
White-naped Crane	-6178795.016	5932900.438
White-naped Crane	-6225413.392	5898320.478
White-naped Crane	-6175023.511	5951833.165
White-naped Crane	-6175021.285	5951824.99
White-naped Crane	-6191912.904	5989275.655
White-naped Crane	-6178792.789	5932898.806
White-naped Crane	-6225411.166	5898323.728
White-naped Crane	-6174999.021	5935923.811
White-naped Crane	-6172895.083	5945457.728
White-naped Crane	-6191912.904	5989272.371
White-naped Crane	-6175016.832	5951824.99
White-naped Crane	-6178797.242	5932897.175
White-naped Crane	-6225397.807	5898335.101
White-naped Crane	-6175005.7	5935930.339
White-naped Crane	-6175014.606	5951846.244
White-naped Crane	-6191912.904	5989275.655
White-naped Crane	-6178792.789	5932897.175
White-naped Crane	-6225397.807	5898339.976
White-naped Crane	-6175070.265	5935896.069
White-naped Crane	-6172921.799	5945436.49
White-naped Crane	-6178592.414	5933308.278
White-naped Crane	-6191912.904	5989274.013
White-naped Crane	-6225357.732	5898252.237
White-naped Crane	-6171568.154	5937461.195
White-naped Crane	-6171884.302	5943843.78
White-naped Crane	-6171864.264	5943833.979
White-naped Crane	-6172467.616	5951385.21
White-naped Crane	-6178196.117	5933412.689
White-naped Crane	-6192097.695	5988960.386
White-naped Crane	-6225179.621	5898614.573
White-naped Crane	-6171508.042	5937438.344
White-naped Crane	-6172340.712	5943958.118
White-naped Crane	-6169624.516	5950371.676
White-naped Crane	-6170860.162	5936568.435
White-naped Crane	-6226312.853	5897509.738
White-naped Crane	-6171300.988	5937247.381
White-naped Crane	-6191307.326	5989246.098
White-naped Crane	-6169070.145	5949642.659
White-naped Crane	-6191999.734	5988881.57
White-naped Crane	-6171906.566	5943961.385
White-naped Crane	-6173950.391	5934634.71
White-naped Crane	-6226261.647	5897537.358
White-naped Crane	-6172808.253	5945795.911
White-naped Crane	-6169214.86	5949982.641
White-naped Crane	-6192006.413	5988894.706
White-naped Crane	-6170606.354	5937066.215
White-naped Crane	-6178552.339	5932815.609

White-naped Crane	-6172712.519	5945478.966
White-naped Crane	-6178347.511	5932784.614
White-naped Crane	-6192001.96	5988891.422
White-naped Crane	-6169283.878	5949771.786
White-naped Crane	-6178331.927	5932822.134
White-naped Crane	-6174161.898	5935021.42
White-naped Crane	-6172721.424	5945457.728
White-naped Crane	-6192055.393	5988853.657
White-naped Crane	-6226288.363	5897469.122
White-naped Crane	-6172576.709	5945171.834
White-naped Crane	-6164087.485	5953425.719
White-naped Crane	-6177926.724	5932763.407
White-naped Crane	-6177060.658	5935308.608
White-naped Crane	-6192053.167	5988850.373
White-naped Crane	-6226319.533	5897435.005
White-naped Crane	-6163018.817	5954441.248
White-naped Crane	-6177911.139	5932773.195
White-naped Crane	-6192053.167	5988856.941
White-naped Crane	-6177227.637	5934001.652
White-naped Crane	-6172458.71	5944944.76
White-naped Crane	-6226283.91	5897456.125
White-naped Crane	-6162929.762	5954253.178
White-naped Crane	-6191180.422	5989045.77
White-naped Crane	-6173017.534	5944309.311
White-naped Crane	-6226317.306	5897597.47
White-naped Crane	-6177016.13	5933903.76
White-naped Crane	-6174344.462	5945104.855
White-naped Crane	-6178247.324	5933249.548
White-naped Crane	-6177218.732	5933923.339
White-naped Crane	-6191897.32	5989310.138
White-naped Crane	-6226335.117	5897603.969
White-naped Crane	-6162905.272	5954210.658
White-naped Crane	-6174342.236	5945090.152
White-naped Crane	-6165632.599	5959649.969
White-naped Crane	-6191859.471	5989280.581
White-naped Crane	-6177094.054	5934050.598
White-naped Crane	-6225270.903	5899037.047
White-naped Crane	-6165614.788	5959604.15
White-naped Crane	-6191892.867	5989257.592
White-naped Crane	-6178574.603	5933360.483
White-naped Crane	-6174342.236	5945081.984
White-naped Crane	-6225279.809	5899020.797
White-naped Crane	-6165739.466	5959643.423
White-naped Crane	-6171939.961	5943688.609
White-naped Crane	-6191910.678	5989267.444
White-naped Crane	-6178599.093	5933402.9
White-naped Crane	-6225295.393	5899027.297
White-naped Crane	-6171178.536	5936684.309
White-naped Crane	-6172670.217	5945109.756
White-naped Crane	-6165532.412	5959587.786
White-naped Crane	-6225239.734	5898575.576
White-naped Crane	-6178610.225	5933282.176
White-naped Crane	-6191910.678	5989265.802
White-naped Crane	-6170842.351	5937395.908
White-naped Crane	-6172672.444	5945114.657
White-naped Crane	-6174306.614	5936392.178
White-naped Crane	-6165572.487	5959555.058
White-naped Crane	-6178574.603	5933358.852

White-naped Crane	-6191917.357	5989274.013
White-naped Crane	-6225215.243	5898638.946
White-naped Crane	-6165561.355	5959556.694
White-naped Crane	-6178581.282	5933352.326
White-naped Crane	-6225213.017	5898651.945
White-naped Crane	-6191919.584	5989277.297
White-naped Crane	-6174302.161	5936388.914
White-naped Crane	-6165565.807	5959540.331
White-naped Crane	-6178583.509	5933347.432
White-naped Crane	-6191919.584	5989274.013
White-naped Crane	-6225213.017	5898629.197
White-naped Crane	-6174306.614	5936392.178
White-naped Crane	-6172672.444	5945114.657
White-naped Crane	-6178576.829	5933350.695
White-naped Crane	-6191919.584	5989254.308
White-naped Crane	-6165579.166	5959550.149
White-naped Crane	-6174311.067	5936382.387
White-naped Crane	-6172685.802	5945114.657
White-naped Crane	-6225213.017	5898648.695
White-naped Crane	-6165570.26	5959555.058
White-naped Crane	-6191915.131	5989265.802
White-naped Crane	-6172999.723	5945397.281
White-naped Crane	-6225213.017	5898648.695
White-naped Crane	-6178645.848	5933229.971
White-naped Crane	-6174306.614	5936385.65
White-naped Crane	-6178643.621	5933234.866
White-naped Crane	-6165565.807	5959571.422
White-naped Crane	-6174319.972	5936379.123
White-naped Crane	-6191915.131	5989282.223
White-naped Crane	-6173004.176	5945405.449
White-naped Crane	-6225210.791	5898647.07
White-naped Crane	-6173015.308	5945408.717
White-naped Crane	-6165576.939	5959555.058
White-naped Crane	-6191924.036	5989283.865
White-naped Crane	-6225208.564	5898648.695
White-naped Crane	-6178650.3	5933229.971
White-naped Crane	-6174322.199	5936384.019
White-naped Crane	-6173030.892	5945387.479
White-naped Crane	-6174322.199	5936382.387
White-naped Crane	-6225213.017	5898650.32
White-naped Crane	-6178648.074	5933231.603
White-naped Crane	-6191919.584	5989283.865
White-naped Crane	-6165579.166	5959577.967
White-naped Crane	-6191926.263	5989308.496
White-naped Crane	-6225217.47	5898630.821
White-naped Crane	-6178565.698	5933291.964
White-naped Crane	-6173010.855	5945420.153
White-naped Crane	-6174326.651	5936380.755
White-naped Crane	-6172033.47	5944013.655
White-naped Crane	-6192059.846	5988924.262
White-naped Crane	-6178178.306	5933136.983
White-naped Crane	-6225077.207	5899009.423
White-naped Crane	-6171488.004	5937492.207
White-naped Crane	-6171835.321	5943504.04
White-naped Crane	-6191852.792	5989021.14
White-naped Crane	-6226279.458	5897569.851
White-naped Crane	-6171510.268	5937258.806
White-naped Crane	-6170768.88	5936098.426

White-naped Crane	-6170813.408	5936292.628
White-naped Crane	-6172627.916	5945108.122
White-naped Crane	-6174226.464	5935151.959
White-naped Crane	-6192678.783	5988986.658
White-naped Crane	-6170800.05	5936268.148
White-naped Crane	-6192678.783	5988989.942
White-naped Crane	-6175179.359	5932583.967
White-naped Crane	-6225368.864	5898328.602
White-naped Crane	-6170860.162	5936349.747
White-naped Crane	-6192015.318	5988797.83
White-naped Crane	-6175139.284	5932659.005
White-naped Crane	-6225348.827	5898159.624
White-naped Crane	-6182078.941	5915555.704
White-naped Crane	-6178414.303	5932778.089
White-naped Crane	-6178227.286	5949140.883
White-naped Crane	-6225669.427	5898006.896
White-naped Crane	-6177989.063	5933158.191
White-naped Crane	-6191928.489	5989014.572
White-naped Crane	-6173462.812	5931691.711
White-naped Crane	-6175014.606	5943722.909
White-naped Crane	-6226283.91	5897456.125
White-naped Crane	-6174262.086	5944972.531
White-naped Crane	-6178066.986	5933229.971
White-naped Crane	-6226290.59	5897514.612
White-naped Crane	-6173467.265	5931660.72
White-naped Crane	-6191952.979	5989024.424
White-naped Crane	-6191959.659	5989024.424
White-naped Crane	-6178222.833	5933073.36
White-naped Crane	-6173502.887	5931631.361
White-naped Crane	-6174242.048	5945973.993
White-naped Crane	-6226272.778	5897558.478
White-naped Crane	-6168566.981	5938473.211
White-naped Crane	-6178365.322	5933283.807
White-naped Crane	-6226275.005	5897537.358
White-naped Crane	-6173469.491	5931670.507
White-naped Crane	-6191928.489	5989053.98
White-naped Crane	-6174375.632	5946235.406
White-naped Crane	-6174567.101	5946295.858
White-naped Crane	-6191941.848	5989037.56
White-naped Crane	-6168589.245	5938309.974
White-naped Crane	-6178164.947	5933442.054
White-naped Crane	-6173400.473	5931680.293
White-naped Crane	-6226335.117	5897621.84
White-naped Crane	-6168609.282	5938256.107
White-naped Crane	-6178133.778	5933460
White-naped Crane	-6225620.446	5898430.966
White-naped Crane	-6174569.328	5946300.76
White-naped Crane	-6191955.206	5989026.066
White-naped Crane	-6168571.434	5938492.799
White-naped Crane	-6191979.696	5989021.14
White-naped Crane	-6178182.758	5933326.224
White-naped Crane	-6180291.15	5944789.569
White-naped Crane	-6225290.941	5899093.92
White-naped Crane	-6180211	5944725.86
White-naped Crane	-6168629.32	5938313.239
White-naped Crane	-6178131.551	5933460
White-naped Crane	-6191997.507	5988968.596
White-naped Crane	-6169377.387	5937204.945

White-naped Crane	-6192124.411	5988945.608
White-naped Crane	-6176399.42	5947815.485
White-naped Crane	-6172472.069	5945253.517
White-naped Crane	-6191935.168	5988973.522
White-naped Crane	-6178416.529	5932854.761
White-naped Crane	-6179135.653	5933409.426
White-naped Crane	-6227951.476	5904727.832
White-naped Crane	-6172434.22	5945211.042
White-naped Crane	-6178418.756	5932846.604
White-naped Crane	-6179146.785	5933265.862
White-naped Crane	-6176403.873	5947815.485
White-naped Crane	-6227276.88	5904667.67
White-naped Crane	-6191926.263	5988976.806
White-naped Crane	-6172431.994	5945217.577
White-naped Crane	-6227279.107	5904748.97
White-naped Crane	-6191930.716	5988980.09
White-naped Crane	-6176401.647	5947810.582
White-naped Crane	-6179149.012	5933260.968
White-naped Crane	-6178418.756	5932843.341
White-naped Crane	-6191932.942	5988981.732
White-naped Crane	-6178423.209	5932849.867
White-naped Crane	-6179146.785	5933272.388
White-naped Crane	-6227281.333	5904752.222
White-naped Crane	-6176412.779	5947812.216
White-naped Crane	-6172436.446	5945237.181
White-naped Crane	-6179149.012	5933262.599
White-naped Crane	-6191928.489	5988957.102
White-naped Crane	-6176397.194	5947823.655
White-naped Crane	-6227316.955	5904657.914
White-naped Crane	-6178416.529	5932853.129
White-naped Crane	-6172454.257	5945238.814
White-naped Crane	-6176379.383	5947823.655
White-naped Crane	-6179146.785	5933269.125
White-naped Crane	-6191932.942	5988958.744
White-naped Crane	-6172454.257	5945235.547
White-naped Crane	-6178416.529	5932854.761
White-naped Crane	-6227301.371	5904651.41
White-naped Crane	-6176397.194	5947849.802
White-naped Crane	-6172476.521	5945225.745
White-naped Crane	-6179149.012	5933265.862
White-naped Crane	-6191930.716	5988957.102
White-naped Crane	-6178416.529	5932854.761
White-naped Crane	-6227301.371	5904653.036
White-naped Crane	-6172467.616	5945232.28
White-naped Crane	-6178418.756	5932856.392
White-naped Crane	-6191932.942	5988955.46
White-naped Crane	-6179151.238	5933259.336
White-naped Crane	-6227303.597	5904651.41
White-naped Crane	-6176403.873	5947807.314
White-naped Crane	-6176381.609	5947792.606
White-naped Crane	-6191937.395	5988958.744
White-naped Crane	-6227292.465	5904828.645
White-naped Crane	-6178102.608	5933716.138
White-naped Crane	-6179177.955	5933260.968
White-naped Crane	-6172512.144	5945137.528
White-naped Crane	-6171521.4	5944392.619
White-naped Crane	-6171799.699	5943567.74
White-naped Crane	-6178443.246	5932751.988

White-naped Crane	-6170270.169	5937084.168
White-naped Crane	-6227270.201	5904732.71
White-naped Crane	-6191932.942	5988955.46
White-naped Crane	-6178650.3	5932830.291
White-naped Crane	-6226744.773	5904537.591
White-naped Crane	-6171574.833	5944199.868
White-naped Crane	-6192478.407	5989559.735
White-naped Crane	-6171826.416	5943566.107
White-naped Crane	-6172492.106	5945426.687
White-naped Crane	-6172558.898	5945426.687
White-naped Crane	-6226444.21	5905090.44
White-naped Crane	-6192465.049	5989595.862
White-naped Crane	-6179776.854	5932463.254
White-naped Crane	-6173053.156	5934003.283
White-naped Crane	-6172757.046	5946067.121
White-naped Crane	-6172619.01	5945456.094
White-naped Crane	-6179741.231	5932875.968
White-naped Crane	-6191256.119	5989114.735
White-naped Crane	-6174262.086	5932711.206
White-naped Crane	-6226303.948	5905166.866
White-naped Crane	-6191291.742	5989361.042
White-naped Crane	-6174812.004	5932394.742
White-naped Crane	-6172828.291	5946132.474
White-naped Crane	-6179269.237	5933322.961
White-naped Crane	-6178358.643	5930631.564
White-naped Crane	-6191590.078	5989548.24
White-naped Crane	-6227107.675	5904509.949
White-naped Crane	-6173160.023	5946158.615
White-naped Crane	-6174201.973	5945686.449
White-naped Crane	-6173093.231	5946240.307
White-naped Crane	-6178360.87	5931197.503
White-naped Crane	-6191545.55	5989528.535
White-naped Crane	-6227129.939	5904644.906
White-naped Crane	-6173068.741	5946233.772
White-naped Crane	-6227118.807	5904612.386
White-naped Crane	-6191541.097	5989513.756
White-naped Crane	-6173338.134	5931588.953
White-naped Crane	-6174315.519	5947232.112
White-naped Crane	-6173086.552	5946238.673
White-naped Crane	-6178294.078	5932481.197
White-naped Crane	-6173476.17	5931672.138
White-naped Crane	-6227308.05	5904755.474
White-naped Crane	-6191797.132	5989280.581
White-naped Crane	-6191790.453	5989214.899
White-naped Crane	-6173086.552	5946212.532
White-naped Crane	-6173489.529	5931701.498
White-naped Crane	-6174302.161	5946642.244
White-naped Crane	-6227308.05	5904664.418
White-naped Crane	-6191803.811	5989191.91
White-naped Crane	-6178612.452	5933419.214
White-naped Crane	-6173467.265	5931665.614
White-naped Crane	-6173168.929	5946282.787
White-naped Crane	-6227185.598	5904649.784
White-naped Crane	-6174353.368	5946521.334
White-naped Crane	-6172834.97	5945310.696
White-naped Crane	-6191846.113	5989095.031
White-naped Crane	-6179509.687	5933357.22
White-naped Crane	-6173171.155	5931367.129

White-naped Crane	-6227156.655	5904480.682
White-naped Crane	-6172400.824	5944595.175
White-naped Crane	-6173117.722	5931345.926
White-naped Crane	-6191725.888	5989177.132
White-naped Crane	-6174255.407	5946382.453
White-naped Crane	-6227103.222	5904568.485
White-naped Crane	-6172661.312	5945487.134
White-naped Crane	-6172701.387	5945652.141
White-naped Crane	-6168865.317	5938620.126
White-naped Crane	-6226326.212	5905025.397
White-naped Crane	-6179033.239	5933448.58
White-naped Crane	-6192191.203	5989134.439
White-naped Crane	-6179661.081	5932706.312
White-naped Crane	-6227154.429	5904558.729
White-naped Crane	-6172788.216	5945969.092
White-naped Crane	-6173068.741	5946119.403
White-naped Crane	-6173062.062	5946166.784
White-naped Crane	-6191730.34	5989098.315
White-naped Crane	-6179698.93	5932740.569
White-naped Crane	-6175702.56	5939393.922
White-naped Crane	-6227112.127	5904535.965
White-naped Crane	-6172696.934	5945954.388
White-naped Crane	-6173037.572	5946215.799
White-naped Crane	-6175691.428	5939375.964
White-naped Crane	-6179696.704	5932740.569
White-naped Crane	-6191732.567	5989104.883
White-naped Crane	-6227125.486	5904516.453
White-naped Crane	-6191730.34	5989095.031
White-naped Crane	-6179698.93	5932737.307
White-naped Crane	-6175693.655	5939371.066
White-naped Crane	-6173050.93	5946134.107
White-naped Crane	-6172701.387	5945952.754
White-naped Crane	-6172701.387	5945957.655
White-naped Crane	-6179696.704	5932734.044
White-naped Crane	-6175689.202	5939367.801
White-naped Crane	-6191725.888	5989099.957
White-naped Crane	-6173066.515	5946127.572
White-naped Crane	-6227121.033	5904514.827
White-naped Crane	-6173148.891	5945983.796
White-naped Crane	-6175689.202	5939371.066
White-naped Crane	-6191725.888	5989098.315
White-naped Crane	-6179692.251	5932732.413
White-naped Crane	-6172681.349	5945949.486
White-naped Crane	-6227127.712	5904480.682
White-naped Crane	-6172690.255	5945957.655
White-naped Crane	-6173133.306	5945993.599
White-naped Crane	-6191721.435	5989106.525
White-naped Crane	-6175686.975	5939374.331
White-naped Crane	-6179696.704	5932735.675
White-naped Crane	-6227134.391	5904516.453
White-naped Crane	-6191725.888	5989090.104
White-naped Crane	-6227132.165	5904519.705
White-naped Crane	-6179698.93	5932747.094
White-naped Crane	-6173155.57	5945996.867
White-naped Crane	-6175689.202	5939377.596
White-naped Crane	-6172696.934	5945962.557
White-naped Crane	-6172696.934	5945964.191
White-naped Crane	-6173160.023	5946000.134

White-naped Crane	-6191725.888	5989091.746
White-naped Crane	-6179696.704	5932742.201
White-naped Crane	-6227134.391	5904521.331
White-naped Crane	-6175689.202	5939387.392
White-naped Crane	-6173146.665	5945991.965
White-naped Crane	-6179656.629	5932701.418
White-naped Crane	-6175691.428	5939372.699
White-naped Crane	-6191725.888	5989090.104
White-naped Crane	-6172696.934	5945954.388
White-naped Crane	-6227011.94	5904513.201
White-naped Crane	-6171873.17	5943722.909
White-naped Crane	-6174705.138	5938889.477
White-naped Crane	-6192674.33	5989548.24
White-naped Crane	-6227283.559	5904682.304
White-naped Crane	-6170860.162	5936331.795
White-naped Crane	-6172750.367	5945431.588
White-naped Crane	-6170840.125	5936315.475
White-naped Crane	-6168989.995	5939178.427
White-naped Crane	-6192640.934	5989556.451
White-naped Crane	-6226306.174	5905004.258
White-naped Crane	-6172305.089	5945405.449
White-naped Crane	-6172630.142	5945335.201
White-naped Crane	-6172703.613	5945475.698
White-naped Crane	-6178603.546	5931551.438
White-naped Crane	-6192647.613	5989558.093
White-naped Crane	-6169388.519	5938429.137
White-naped Crane	-6227087.637	5904500.194
White-naped Crane	-6172641.274	5945416.885
White-naped Crane	-6191739.246	5989131.155
White-naped Crane	-6178601.32	5931554.7
White-naped Crane	-6173151.117	5931893.969
White-naped Crane	-6172619.01	5945194.705
White-naped Crane	-6227285.786	5904646.532
White-naped Crane	-6172626.803	5945444.658
White-naped Crane	-6179265.897	5933052.152
White-naped Crane	-6191737.02	5988999.794
White-naped Crane	-6227272.428	5904670.922
White-naped Crane	-6172601.199	5945104.855
White-naped Crane	-6173102.137	5931378.546
White-naped Crane	-6172621.237	5945469.164
White-naped Crane	-6227320.295	5904677.426
White-naped Crane	-6178913.014	5933205.501
White-naped Crane	-6191767.076	5988989.942
White-naped Crane	-6172582.275	5945096.687
White-naped Crane	-6172608.992	5945439.757
White-naped Crane	-6178144.91	5933298.49
White-naped Crane	-6173064.288	5931266.006
White-naped Crane	-6191689.152	5988833.953
White-naped Crane	-6172716.971	5945449.559
White-naped Crane	-6178152.702	5933419.214
White-naped Crane	-6173262.437	5931437.264
White-naped Crane	-6174382.311	5946238.673
White-naped Crane	-6191810.491	5989180.416
White-naped Crane	-6227158.882	5904667.67
White-naped Crane	-6178172.74	5933339.275
White-naped Crane	-6191814.943	5989182.058
White-naped Crane	-6174378.971	5946248.476
White-naped Crane	-6173566.339	5931535.127

White-naped Crane	-6172607.878	5945269.854
White-naped Crane	-6228471.338	5906976.895
White-naped Crane	-6174450.216	5946375.918
White-naped Crane	-6172838.31	5946112.868
White-naped Crane	-6178257.343	5932809.084
White-naped Crane	-6191890.641	5989269.087
White-naped Crane	-6228466.886	5906918.344
White-naped Crane	-6173107.703	5931288.84
White-naped Crane	-6172794.895	5946197.827
White-naped Crane	-6178399.831	5932815.609
White-naped Crane	-6228502.508	5907011.05
White-naped Crane	-6191806.038	5989180.416
White-naped Crane	-6174804.212	5946284.421
White-naped Crane	-6173098.797	5931290.471
White-naped Crane	-6172504.351	5945343.369
White-naped Crane	-6178551.226	5932843.341
White-naped Crane	-6191818.283	5989162.354
White-naped Crane	-6172870.592	5930910.452
White-naped Crane	-6228499.168	5906991.533
White-naped Crane	-6172998.61	5930788.132
White-naped Crane	-6178729.337	5932980.373
White-naped Crane	-6172292.844	5944565.772
White-naped Crane	-6191830.528	5989167.28
White-naped Crane	-6172347.391	5944580.474
White-naped Crane	-6172046.828	5943424.007
White-naped Crane	-6178933.052	5933048.889
White-naped Crane	-6192455.03	5989459.567
White-naped Crane	-6172049.055	5943433.807
White-naped Crane	-6172994.157	5930639.719
White-naped Crane	-6172571.143	5945513.274
White-naped Crane	-6178886.298	5932960.797
White-naped Crane	-6172861.687	5930577.745
White-naped Crane	-6191725.888	5989119.661
White-naped Crane	-6172423.088	5945585.157
White-naped Crane	-6227289.125	5904700.19
White-naped Crane	-6172404.164	5945580.256
White-naped Crane	-6172566.69	5945652.141
White-naped Crane	-6176769.001	5933208.763
White-naped Crane	-6191816.056	5988853.657
White-naped Crane	-6227302.484	5904661.166
White-naped Crane	-6178789.45	5932889.018
White-naped Crane	-6172595.633	5945581.89
White-naped Crane	-6227298.031	5904661.166
White-naped Crane	-6176775.68	5933216.92
White-naped Crane	-6178789.45	5932903.7
White-naped Crane	-6191819.396	5988855.299
White-naped Crane	-6172611.218	5945585.157
White-naped Crane	-6172598.973	5945576.989
White-naped Crane	-6176775.68	5933210.395
White-naped Crane	-6178786.11	5932892.281
White-naped Crane	-6227303.597	5904657.914
White-naped Crane	-6172598.973	5945576.989
White-naped Crane	-6191822.736	5988860.225
White-naped Crane	-6178789.45	5932890.65
White-naped Crane	-6176771.227	5933218.552
White-naped Crane	-6191822.736	5988858.583
White-naped Crane	-6227301.371	5904656.288
White-naped Crane	-6172596.746	5945588.425

White-naped Crane	-6172605.652	5945570.454
White-naped Crane	-6172691.368	5945506.739
White-naped Crane	-6178783.884	5932887.387
White-naped Crane	-6176767.888	5933216.92
White-naped Crane	-6172699.16	5945514.907
White-naped Crane	-6191821.622	5988858.583
White-naped Crane	-6227300.257	5904659.54
White-naped Crane	-6172700.274	5945513.274
White-naped Crane	-6191818.283	5988858.583
White-naped Crane	-6176767.888	5933225.077
White-naped Crane	-6178786.11	5932889.018
White-naped Crane	-6172692.481	5945514.907
White-naped Crane	-6227291.352	5904674.174
White-naped Crane	-6172701.387	5945526.343
White-naped Crane	-6172696.934	5945511.64
White-naped Crane	-6191816.056	5988850.373
White-naped Crane	-6178789.45	5932895.544
White-naped Crane	-6176676.606	5933011.368
White-naped Crane	-6227302.484	5904662.792
White-naped Crane	-6172711.405	5945483.867
White-naped Crane	-6176671.04	5933001.58
White-naped Crane	-6227306.937	5904654.662
White-naped Crane	-6178789.45	5932892.281
White-naped Crane	-6191817.17	5988855.299
White-naped Crane	-6172699.16	5945514.907
White-naped Crane	-6227283.559	5904682.304
White-naped Crane	-6176605.361	5933065.203
White-naped Crane	-6191818.283	5988860.225
White-naped Crane	-6178789.45	5932898.806
White-naped Crane	-6172643.501	5945454.46
White-naped Crane	-6172643.501	5945454.46
White-naped Crane	-6172914.007	5946238.673
White-naped Crane	-6193779.732	5989502.261
White-naped Crane	-6174494.744	5946125.938
White-naped Crane	-6178926.373	5933050.521
White-naped Crane	-6170783.352	5936428.082
White-naped Crane	-6226468.701	5905061.17
White-naped Crane	-6170506.166	5945282.923
White-naped Crane	-6170517.298	5945302.527
White-naped Crane	-6191727.001	5989065.474
White-naped Crane	-6227289.125	5904680.678
White-naped Crane	-6170814.521	5936429.714
White-naped Crane	-6172254.996	5945918.444
White-naped Crane	-6177078.469	5933621.513
White-naped Crane	-6174008.278	5934592.287
White-naped Crane	-6191747.038	5988998.152
White-naped Crane	-6178934.165	5933060.309
White-naped Crane	-6174040.56	5934435.65
White-naped Crane	-6172534.407	5945434.856
White-naped Crane	-6172468.729	5945864.529
White-naped Crane	-6228945.559	5910484.195
White-naped Crane	-6191742.586	5988962.028
White-naped Crane	-6172366.315	5945825.319
White-naped Crane	-6174016.07	5934324.701
White-naped Crane	-6191728.114	5988986.658
White-naped Crane	-6172643.501	5945911.909
White-naped Crane	-6178926.373	5933052.152
White-naped Crane	-6228415.679	5909215.156

White-naped Crane	-6179912.663	5932694.893
White-naped Crane	-6172455.371	5945805.714
White-naped Crane	-6191712.529	5989101.599
White-naped Crane	-6172993.044	5947565.463
White-naped Crane	-6228495.829	5908779.17
White-naped Crane	-6172961.874	5946238.673
White-naped Crane	-6172725.877	5946011.571
White-naped Crane	-6175086.963	5932670.424
White-naped Crane	-6191741.472	5989052.338
White-naped Crane	-6178941.957	5932174.529
White-naped Crane	-6228494.716	5908777.544
White-naped Crane	-6172932.931	5946176.587
White-naped Crane	-6172957.422	5946269.717
White-naped Crane	-6191758.17	5989081.894
White-naped Crane	-6178944.184	5932171.267
White-naped Crane	-6175107.001	5932597.017
White-naped Crane	-6228505.847	5908949.983
White-naped Crane	-6172993.044	5946122.671
White-naped Crane	-6173091.005	5946353.044
White-naped Crane	-6178927.486	5932166.373
White-naped Crane	-6191578.946	5989535.104
White-naped Crane	-6173037.572	5930683.753
White-naped Crane	-6228543.696	5906915.091
White-naped Crane	-6172950.742	5946268.083
White-naped Crane	-6179304.859	5931920.068
White-naped Crane	-6173053.156	5930628.302
White-naped Crane	-6191574.493	5989558.093
White-naped Crane	-6172986.365	5946098.163
White-naped Crane	-6228490.263	5907006.171
White-naped Crane	-6191545.55	5989528.535
White-naped Crane	-6173001.949	5930644.611
White-naped Crane	-6179313.764	5931920.068
White-naped Crane	-6172930.705	5946148.812
White-naped Crane	-6228301.02	5906947.62
White-naped Crane	-6172983.025	5946094.896
White-naped Crane	-6172978.572	5946094.896
White-naped Crane	-6179366.085	5931825.462
White-naped Crane	-6173534.057	5930864.785
White-naped Crane	-6191723.661	5989034.276
White-naped Crane	-6228491.376	5906985.027
White-naped Crane	-6172942.95	5946081.825
White-naped Crane	-6173039.798	5946201.095
White-naped Crane	-6172984.138	5946098.163
White-naped Crane	-6179363.858	5931822.2
White-naped Crane	-6172978.572	5930581.007
White-naped Crane	-6228510.3	5907006.171
White-naped Crane	-6191783.774	5989003.078
White-naped Crane	-6172987.478	5946094.896
White-naped Crane	-6228395.641	5906924.85
White-naped Crane	-6192453.917	5989461.209
White-naped Crane	-6172998.61	5930626.672
White-naped Crane	-6172931.818	5946222.335
White-naped Crane	-6179420.631	5931911.912
White-naped Crane	-6191712.529	5989109.809
White-naped Crane	-6227308.05	5904657.914
White-naped Crane	-6173191.192	5946259.913
White-naped Crane	-6227273.541	5904662.792
White-naped Crane	-6179361.632	5931845.035

White-naped Crane	-6174235.369	5930028.154
White-naped Crane	-6191724.775	5989095.031
White-naped Crane	-6174238.709	5930033.047
White-naped Crane	-6179359.405	5932083.183
White-naped Crane	-6191730.34	5989101.599
White-naped Crane	-6227308.05	5904664.418
White-naped Crane	-6179361.632	5932075.027
White-naped Crane	-6173224.588	5946245.209
White-naped Crane	-6174253.18	5929928.677
White-naped Crane	-6173196.758	5946256.646
White-naped Crane	-6191727.001	5989111.451
White-naped Crane	-6227304.71	5904651.41
White-naped Crane	-6173201.211	5946245.209
White-naped Crane	-6173235.72	5946269.717
White-naped Crane	-6191730.34	5989101.599
White-naped Crane	-6174258.746	5929892.8
White-naped Crane	-6227316.955	5904679.052
White-naped Crane	-6179371.651	5932084.814
White-naped Crane	-6173223.475	5946269.717
White-naped Crane	-6191730.34	5989103.241
White-naped Crane	-6173194.532	5946256.646
White-naped Crane	-6174250.954	5929907.477
White-naped Crane	-6227277.993	5904670.922
White-naped Crane	-6179358.292	5932073.396
White-naped Crane	-6179358.292	5932083.183
White-naped Crane	-6191730.34	5989108.167
White-naped Crane	-6174250.954	5929904.216
White-naped Crane	-6173193.419	5946259.913
White-naped Crane	-6227282.446	5904664.418
White-naped Crane	-6173191.192	5946245.209
White-naped Crane	-6173190.079	5946256.646
White-naped Crane	-6191732.567	5989108.167
White-naped Crane	-6179364.971	5932083.183
White-naped Crane	-6227283.559	5904674.174
White-naped Crane	-6174258.746	5929873.231
White-naped Crane	-6173193.419	5946261.547
White-naped Crane	-6191729.227	5989103.241
White-naped Crane	-6227281.333	5904675.8
White-naped Crane	-6173182.287	5946235.406
White-naped Crane	-6174252.067	5929904.216
White-naped Crane	-6173193.419	5946276.252
White-naped Crane	-6191737.02	5989141.007
White-naped Crane	-6179322.67	5932076.658
White-naped Crane	-6173207.89	5946204.363
White-naped Crane	-6178317.455	5932654.111
White-naped Crane	-6191346.288	5989305.212
White-naped Crane	-6226954.054	5903314.945
White-naped Crane	-6170725.466	5936276.308
White-naped Crane	-6170786.691	5944286.442
White-naped Crane	-6172645.727	5944954.562
White-naped Crane	-6172456.484	5945124.459
White-naped Crane	-6170775.56	5936266.516
White-naped Crane	-6227090.977	5903303.565
White-naped Crane	-6191817.17	5989177.132
White-naped Crane	-6170767.767	5936266.516
White-naped Crane	-6174072.843	5934226.806
White-naped Crane	-6170873.521	5936542.322
White-naped Crane	-6172532.181	5945229.012

White-naped Crane	-6172336.259	5944255.406
White-naped Crane	-6192119.959	5988149.278
White-naped Crane	-6172507.691	5945501.838
White-naped Crane	-6172452.031	5945487.134
White-naped Crane	-6191823.849	5988605.722
White-naped Crane	-6178544.547	5933006.474
White-naped Crane	-6174321.085	5931706.391
White-naped Crane	-6227174.466	5903464.515
White-naped Crane	-6178544.547	5932996.686
White-naped Crane	-6172529.955	5945831.854
White-naped Crane	-6192122.185	5988155.846
White-naped Crane	-6227039.77	5903370.221
White-naped Crane	-6172970.78	5930801.179
White-naped Crane	-6172532.181	5945833.488
White-naped Crane	-6178462.17	5933074.991
White-naped Crane	-6191847.226	5988607.364
White-naped Crane	-6172988.591	5930797.917
White-naped Crane	-6229213.839	5906693.901
White-naped Crane	-6172545.539	5945951.12
White-naped Crane	-6172626.803	5945908.642
White-naped Crane	-6178300.757	5932928.17
White-naped Crane	-6191771.529	5988981.732
White-naped Crane	-6172965.214	5930762.037
White-naped Crane	-6229200.481	5906689.022
White-naped Crane	-6172706.953	5945952.754
White-naped Crane	-6191763.736	5988985.016
White-naped Crane	-6178210.588	5933078.254
White-naped Crane	-6172640.161	5946000.134
White-naped Crane	-6172572.256	5945835.121
White-naped Crane	-6191797.132	5988985.016
White-naped Crane	-6178221.72	5933280.545
White-naped Crane	-6228119.569	5907061.47
White-naped Crane	-6172826.065	5930737.573
White-naped Crane	-6174331.104	5945601.495
White-naped Crane	-6174402.349	5945621.1
White-naped Crane	-6191945.187	5989098.315
White-naped Crane	-6178406.511	5933221.814
White-naped Crane	-6172929.592	5946089.994
White-naped Crane	-6172988.591	5930773.453
White-naped Crane	-6228100.645	5907051.712
White-naped Crane	-6192015.318	5988876.644
White-naped Crane	-6172716.971	5946253.378
White-naped Crane	-6180463.695	5932019.568
White-naped Crane	-6172983.025	5930779.977
White-naped Crane	-6227997.117	5907129.781
White-naped Crane	-6172683.576	5946232.138
White-naped Crane	-6179417.292	5931898.863
White-naped Crane	-6228019.381	5907090.746
White-naped Crane	-6192032.016	5988884.854
White-naped Crane	-6172769.292	5945113.023
White-naped Crane	-6172642.387	5943714.742
White-naped Crane	-6179363.858	5931823.831
White-naped Crane	-6192237.957	5988829.027
White-naped Crane	-6172641.274	5943732.71
White-naped Crane	-6173026.44	5930846.845
White-naped Crane	-6228450.188	5907017.556
White-naped Crane	-6191721.435	5989113.093
White-naped Crane	-6179372.764	5931843.404

White-naped Crane	-6175708.126	5926261.873
White-naped Crane	-6177788.687	5947251.721
White-naped Crane	-6227286.899	5904679.052
White-naped Crane	-6179833.627	5932130.487
White-naped Crane	-6226285.024	5905113.205
White-naped Crane	-6175180.472	5929100.29
White-naped Crane	-6173230.154	5946310.563
White-naped Crane	-6173247.965	5946323.634
White-naped Crane	-6175171.566	5929101.92
White-naped Crane	-6179697.817	5932751.988
White-naped Crane	-6227318.068	5904677.426
White-naped Crane	-6192163.373	5988804.398
White-naped Crane	-6175174.906	5929064.417
White-naped Crane	-6179686.685	5932765.039
White-naped Crane	-6192160.034	5988806.04
White-naped Crane	-6227285.786	5904714.824
White-naped Crane	-6173482.85	5946341.607
White-naped Crane	-6173490.642	5946338.339
White-naped Crane	-6179691.138	5932765.039
White-naped Crane	-6175174.906	5929062.786
White-naped Crane	-6227299.144	5904714.824
White-naped Crane	-6173477.284	5946317.099
White-naped Crane	-6192186.75	5988799.472
White-naped Crane	-6173482.85	5946325.268
White-naped Crane	-6179688.911	5932763.407
White-naped Crane	-6175172.679	5929061.155
White-naped Crane	-6192186.75	5988806.04
White-naped Crane	-6173510.679	5946308.929
White-naped Crane	-6173476.17	5946320.366
White-naped Crane	-6173481.736	5946317.099
White-naped Crane	-6173481.736	5946323.634
White-naped Crane	-6175171.566	5929062.786
White-naped Crane	-6192183.411	5988797.83
White-naped Crane	-6227293.578	5904711.572
White-naped Crane	-6179688.911	5932742.201
White-naped Crane	-6175172.679	5929079.092
White-naped Crane	-6192182.298	5988794.546
White-naped Crane	-6179684.458	5932742.201
White-naped Crane	-6227292.465	5904718.076
White-naped Crane	-6173471.718	5946302.394
White-naped Crane	-6173480.623	5946322
White-naped Crane	-6192182.298	5988801.114
White-naped Crane	-6179691.138	5932745.463
White-naped Crane	-6173486.189	5946336.705
White-naped Crane	-6227310.276	5904656.288
White-naped Crane	-6175171.566	5929066.047
White-naped Crane	-6173486.189	5946341.607
White-naped Crane	-6175177.132	5929070.939
White-naped Crane	-6192185.637	5988799.472
White-naped Crane	-6179692.251	5932763.407
White-naped Crane	-6227304.71	5904653.036
White-naped Crane	-6173507.34	5946333.437
White-naped Crane	-6173500.661	5946341.607
White-naped Crane	-6173477.284	5946343.24
White-naped Crane	-6175171.566	5929075.831
White-naped Crane	-6192185.637	5988802.756
White-naped Crane	-6227288.012	5904688.808
White-naped Crane	-6179688.911	5932761.776

White-naped Crane	-6179344.934	5932045.666
White-naped Crane	-6194034.654	5988182.115
White-naped Crane	-6170682.051	5936284.468
White-naped Crane	-6171471.306	5945246.982
White-naped Crane	-6170678.712	5936459.09
White-naped Crane	-6194027.975	5988144.353
White-naped Crane	-6227210.089	5907611.227
White-naped Crane	-6179323.783	5932001.625
White-naped Crane	-6172053.507	5943571.007
White-naped Crane	-6172120.299	5943575.907
White-naped Crane	-6191863.924	5988998.152
White-naped Crane	-6179299.293	5931874.396
White-naped Crane	-6175025.738	5932414.317
White-naped Crane	-6173408.265	5945305.795
White-naped Crane	-6173409.379	5945295.993
White-naped Crane	-6191863.924	5988998.152
White-naped Crane	-6175090.303	5932355.593
White-naped Crane	-6227271.314	5904664.418
White-naped Crane	-6172759.273	5945949.486
White-naped Crane	-6172797.121	5946148.812
White-naped Crane	-6191789.34	5988980.09
White-naped Crane	-6174855.419	5932313.181
White-naped Crane	-6179367.198	5931871.133
White-naped Crane	-6191796.019	5988976.806
White-naped Crane	-6174805.325	5932311.55
White-naped Crane	-6172799.348	5946125.938
White-naped Crane	-6179258.105	5931910.281
White-naped Crane	-6228426.811	5906932.982
White-naped Crane	-6172798.235	5946165.15
White-naped Crane	-6191792.679	5988981.732
White-naped Crane	-6172897.309	5946238.673
White-naped Crane	-6178434.341	5932740.569
White-naped Crane	-6172957.422	5946228.87
White-naped Crane	-6178415.416	5932691.631
White-naped Crane	-6191753.718	5988998.152
White-naped Crane	-6172903.988	5946336.705
White-naped Crane	-6172964.101	5930815.857
White-naped Crane	-6229202.707	5906682.516
White-naped Crane	-6172905.101	5946361.213
White-naped Crane	-6172900.649	5946225.603
White-naped Crane	-6178956.429	5933032.576
White-naped Crane	-6172683.576	5946259.913
White-naped Crane	-6173096.571	5931554.7
White-naped Crane	-6191754.831	5989026.066
White-naped Crane	-6229198.255	5906661.373
White-naped Crane	-6178919.693	5933303.384
White-naped Crane	-6172856.121	5946214.166
White-naped Crane	-6172851.668	5946171.686
White-naped Crane	-6191715.869	5989109.809
White-naped Crane	-6228476.904	5906972.016
White-naped Crane	-6173027.553	5931060.5
White-naped Crane	-6172968.554	5946287.689
White-naped Crane	-6173046.477	5931088.227
White-naped Crane	-6191820.509	5989185.342
White-naped Crane	-6228439.056	5906944.367
White-naped Crane	-6179326.01	5931907.018
White-naped Crane	-6172670.217	5946132.474
White-naped Crane	-6171925.49	5943641.241

White-naped Crane	-6179344.934	5931890.707
White-naped Crane	-6173027.553	5931071.917
White-naped Crane	-6192321.447	5988809.324
White-naped Crane	-6171926.603	5943639.608
White-naped Crane	-6228257.605	5906950.872
White-naped Crane	-6179391.688	5931900.494
White-naped Crane	-6229427.573	5906719.923
White-naped Crane	-6173003.063	5931068.655
White-naped Crane	-6171850.906	5943639.608
White-naped Crane	-6192629.802	5989277.297
White-naped Crane	-6173774.507	5944456.326
White-naped Crane	-6179334.915	5932075.027
White-naped Crane	-6191243.874	5988699.313
White-naped Crane	-6173771.167	5944444.891
White-naped Crane	-6170748.843	5936584.755
White-naped Crane	-6228106.21	5906950.872
White-naped Crane	-6174542.611	5944859.813
White-naped Crane	-6179311.538	5931880.92
White-naped Crane	-6191242.761	5988689.461
White-naped Crane	-6174673.968	5935612.125
White-naped Crane	-6174539.272	5944863.08
White-naped Crane	-6175006.813	5944439.991
White-naped Crane	-6175004.587	5944441.624
White-naped Crane	-6174724.062	5935561.538
White-naped Crane	-6179312.651	5931920.068
White-naped Crane	-6191276.157	5988692.745
White-naped Crane	-6228127.361	5907067.976
White-naped Crane	-6175009.04	5944466.127
White-naped Crane	-6175009.04	5944474.294
White-naped Crane	-6191273.931	5988679.609
White-naped Crane	-6179318.217	5931931.485
White-naped Crane	-6228128.474	5907080.988
White-naped Crane	-6174727.401	5935559.906
White-naped Crane	-6191272.817	5988682.893
White-naped Crane	-6179318.217	5931921.699
White-naped Crane	-6228126.248	5907054.964
White-naped Crane	-6174991.229	5944448.158
White-naped Crane	-6175001.247	5944446.525
White-naped Crane	-6174726.288	5935568.065
White-naped Crane	-6174984.549	5944448.158
White-naped Crane	-6191278.383	5988687.819
White-naped Crane	-6174726.288	5935566.434
White-naped Crane	-6179317.104	5931928.223
White-naped Crane	-6228120.682	5907053.338
White-naped Crane	-6175004.587	5944457.959
White-naped Crane	-6175039.096	5944466.127
White-naped Crane	-6191272.817	5988681.251
White-naped Crane	-6179317.104	5931921.699
White-naped Crane	-6174729.628	5935563.17
White-naped Crane	-6175005.7	5944444.891
White-naped Crane	-6228126.248	5907061.47
White-naped Crane	-6191271.704	5988686.177
White-naped Crane	-6179318.217	5931920.068
White-naped Crane	-6174721.835	5935572.961
White-naped Crane	-6175007.927	5944466.127
White-naped Crane	-6228120.682	5907064.723
White-naped Crane	-6175006.813	5944451.425
White-naped Crane	-6175023.511	5944454.692

White-naped Crane	-6174999.021	5944454.692
White-naped Crane	-6179319.33	5931924.961
White-naped Crane	-6191270.591	5988684.535
White-naped Crane	-6174729.628	5935564.802
White-naped Crane	-6228121.795	5907064.723
White-naped Crane	-6174729.628	5935561.538
White-naped Crane	-6191277.27	5988687.819
White-naped Crane	-6228129.588	5907056.591
White-naped Crane	-6179315.991	5931920.068
White-naped Crane	-6174993.455	5944446.525
White-naped Crane	-6175009.04	5944451.425
White-naped Crane	-6175012.379	5944461.226
White-naped Crane	-6175026.851	5944457.959
White-naped Crane	-6174345.576	5934876.198
White-naped Crane	-6191291.742	5988697.671
White-naped Crane	-6228160.757	5907032.194
White-naped Crane	-6172140.336	5943580.807
White-naped Crane	-6192659.858	5989543.314
White-naped Crane	-6228206.398	5906923.223
White-naped Crane	-6180269.999	5932102.757
White-naped Crane	-6171941.075	5943348.875
White-naped Crane	-6171783.001	5943874.814
White-naped Crane	-6171747.379	5936055.996
White-naped Crane	-6192646.5	5989561.377
White-naped Crane	-6228335.529	5907006.171
White-naped Crane	-6171882.075	5937433.448
White-naped Crane	-6192655.405	5989564.661
White-naped Crane	-6227965.948	5907120.023
White-naped Crane	-6174517.008	5945421.786
White-naped Crane	-6178422.095	5932861.286
White-naped Crane	-6174488.065	5945436.49
White-naped Crane	-6173260.211	5946330.17
White-naped Crane	-6178298.531	5932960.797
White-naped Crane	-6191709.19	5989108.167
White-naped Crane	-6173260.211	5946282.787
White-naped Crane	-6174918.871	5933458.369
White-naped Crane	-6228042.758	5907124.902
White-naped Crane	-6173221.249	5946390.623
White-naped Crane	-6191738.133	5989067.116
White-naped Crane	-6178370.888	5933019.525
White-naped Crane	-6228150.738	5907032.194
White-naped Crane	-6173439.435	5931098.013
White-naped Crane	-6180449.223	5933179.399
White-naped Crane	-6173270.229	5931282.316
White-naped Crane	-6173222.362	5946447.809
White-naped Crane	-6191731.454	5989099.957
White-naped Crane	-6173223.475	5946460.88
White-naped Crane	-6228509.187	5906890.695
White-naped Crane	-6173144.438	5946372.65
White-naped Crane	-6191669.115	5989049.054
White-naped Crane	-6173013.081	5930700.062
White-naped Crane	-6180050.7	5932389.848
White-naped Crane	-6228434.603	5907001.292
White-naped Crane	-6173265.777	5946387.355
White-naped Crane	-6179775.74	5932277.294
White-naped Crane	-6191986.375	5989068.758
White-naped Crane	-6173011.968	5930696.8
White-naped Crane	-6173145.551	5946336.705

White-naped Crane	-6228419.018	5907048.459
White-naped Crane	-6173162.249	5946256.646
White-naped Crane	-6174396.783	5946532.772
White-naped Crane	-6174387.877	5946554.012
White-naped Crane	-6179408.386	5931908.65
White-naped Crane	-6191986.375	5989058.906
White-naped Crane	-6227392.653	5907585.202
White-naped Crane	-6176563.06	5945622.733
White-naped Crane	-6191777.095	5988980.09
White-naped Crane	-6179367.198	5931822.2
White-naped Crane	-6230089.924	5909363.201
White-naped Crane	-6173618.659	5931489.458
White-naped Crane	-6176617.606	5945658.676
White-naped Crane	-6191793.793	5988986.658
White-naped Crane	-6173627.565	5931499.244
White-naped Crane	-6179560.894	5931900.494
White-naped Crane	-6229340.744	5906732.934
White-naped Crane	-6179769.061	5932024.461
White-naped Crane	-6173224.588	5946089.994
White-naped Crane	-6191792.679	5988980.09
White-naped Crane	-6172789.329	5943345.608
White-naped Crane	-6191789.34	5988976.806
White-naped Crane	-6226785.961	5904537.591
White-naped Crane	-6170493.921	5936196.342
White-naped Crane	-6172967.44	5934856.617
White-naped Crane	-6171783.001	5936867.099
White-naped Crane	-6191782.661	5988955.46
White-naped Crane	-6172962.988	5934890.883
White-naped Crane	-6173047.59	5934817.457
White-naped Crane	-6224677.57	5903023.942
White-naped Crane	-6192355.956	5988799.472
White-naped Crane	-6227073.166	5904487.186
White-naped Crane	-6171662.776	5937048.261
White-naped Crane	-6174167.464	5934988.785
White-naped Crane	-6174213.105	5934975.732
White-naped Crane	-6174293.255	5934913.727
White-naped Crane	-6174297.708	5934916.99
White-naped Crane	-6180213.226	5932301.762
White-naped Crane	-6192304.749	5988784.694
White-naped Crane	-6174303.274	5934902.305
White-naped Crane	-6229161.519	5906721.549
White-naped Crane	-6174296.595	5934921.885
White-naped Crane	-6180216.566	5932309.918
White-naped Crane	-6174297.708	5934916.99
White-naped Crane	-6192301.409	5988787.978
White-naped Crane	-6229167.085	5906723.176
White-naped Crane	-6174316.633	5934908.832
White-naped Crane	-6192325.9	5988779.768
White-naped Crane	-6174399.009	5934869.671
White-naped Crane	-6180162.019	5932311.55
White-naped Crane	-6229245.009	5906775.22
White-naped Crane	-6174604.95	5934990.417
White-naped Crane	-6174395.669	5934876.198
White-naped Crane	-6174397.896	5934881.093
White-naped Crane	-6180162.019	5932318.075
White-naped Crane	-6174593.818	5934944.729
White-naped Crane	-6229196.028	5906745.945
White-naped Crane	-6174409.028	5934861.512

White-naped Crane	-6192327.013	5988779.768
White-naped Crane	-6192324.787	5988779.768
White-naped Crane	-6174395.669	5934877.829
White-naped Crane	-6180155.34	5932319.706
White-naped Crane	-6229171.538	5906757.33
White-naped Crane	-6174392.33	5934877.829
White-naped Crane	-6180154.227	5932319.706
White-naped Crane	-6229167.085	5906760.583
White-naped Crane	-6192328.126	5988781.41
White-naped Crane	-6174395.669	5934877.829
White-naped Crane	-6174589.365	5934965.941
White-naped Crane	-6174594.931	5934959.415
White-naped Crane	-6175436.507	5935762.255
White-naped Crane	-6229168.198	5906897.2
White-naped Crane	-6180149.774	5932316.443
White-naped Crane	-6192323.673	5988783.052
White-naped Crane	-6192328.126	5988779.768
White-naped Crane	-6175429.827	5935767.151
White-naped Crane	-6229159.293	5906893.948
White-naped Crane	-6180153.113	5932314.812
White-naped Crane	-6174593.818	5934954.519
White-naped Crane	-6175515.543	5935559.906
White-naped Crane	-6175489.94	5935559.906
White-naped Crane	-6175432.054	5935768.783
White-naped Crane	-6192285.825	5988784.694
White-naped Crane	-6229186.01	5906884.189
White-naped Crane	-6180152	5932311.55
White-naped Crane	-6174577.12	5934941.466
White-naped Crane	-6171638.286	5937211.474
White-naped Crane	-6170550.694	5936439.506
White-naped Crane	-6191572.267	5989535.104
White-naped Crane	-6179676.666	5932709.575
White-naped Crane	-6171632.72	5937234.324
White-naped Crane	-6228199.719	5907028.941
White-naped Crane	-6170501.714	5936375.859
White-naped Crane	-6171549.23	5937297.978
White-naped Crane	-6178916.354	5933163.085
White-naped Crane	-6191546.663	5989517.04
White-naped Crane	-6228022.721	5907131.408
White-naped Crane	-6171602.663	5937234.324
White-naped Crane	-6178875.166	5932365.38
White-naped Crane	-6191543.324	5989523.609
White-naped Crane	-6171571.494	5937170.67
White-naped Crane	-6171602.663	5937231.06
White-naped Crane	-6171528.079	5937089.064
White-naped Crane	-6179339.368	5931843.404
White-naped Crane	-6229150.387	5906877.684
White-naped Crane	-6174307.727	5935063.845
White-naped Crane	-6174304.387	5935081.794
White-naped Crane	-6174233.143	5935351.035
White-naped Crane	-6191699.171	5989104.883
White-naped Crane	-6173933.693	5934159.912
White-naped Crane	-6191782.661	5988999.794
White-naped Crane	-6179359.405	5931760.217
White-naped Crane	-6174019.41	5934239.859
White-naped Crane	-6173836.846	5934181.122
White-naped Crane	-6228787.486	5906731.308
White-naped Crane	-6173920.335	5934182.754

White-naped Crane	-6191796.019	5988976.806
White-naped Crane	-6173924.788	5934179.491
White-naped Crane	-6174008.278	5934372.018
White-naped Crane	-6174923.324	5934752.189
White-naped Crane	-6191708.077	5989108.167
White-naped Crane	-6180164.245	5933187.555
White-naped Crane	-6173961.523	5934388.334
White-naped Crane	-6173932.58	5934543.338
White-naped Crane	-6229514.402	5908346.46
White-naped Crane	-6174024.975	5934577.602
White-naped Crane	-6191570.04	5989518.682
White-naped Crane	-6179406.16	5931893.969
White-naped Crane	-6229202.707	5906680.89
White-naped Crane	-6174927.777	5934760.348
White-naped Crane	-6173940.373	5934850.09
White-naped Crane	-6171718.436	5937227.795
White-naped Crane	-6171709.53	5937240.852
White-naped Crane	-6178534.528	5933443.686
White-naped Crane	-6191536.645	5989554.809
White-naped Crane	-6228381.17	5906981.775
White-naped Crane	-6173990.466	5934572.707
White-naped Crane	-6173900.298	5934796.245
White-naped Crane	-6191737.02	5989116.377
White-naped Crane	-6178154.929	5933373.535
White-naped Crane	-6228411.226	5906911.838
White-naped Crane	-6173013.081	5930799.548
White-naped Crane	-6179482.97	5931791.209
White-naped Crane	-6192215.693	5989410.304
White-naped Crane	-6173886.939	5934820.72
White-naped Crane	-6173001.949	5930784.87
White-naped Crane	-6228505.847	5907004.545
White-naped Crane	-6170948.105	5936996.034
White-naped Crane	-6179411.726	5931902.125
White-naped Crane	-6173893.618	5934781.56
White-naped Crane	-6192247.976	5989482.556
White-naped Crane	-6173289.154	5931447.05
White-naped Crane	-6228085.06	5907038.7
White-naped Crane	-6179368.311	5932068.502
White-naped Crane	-6193033.892	5989829.045
White-naped Crane	-6173975.995	5935551.747
White-naped Crane	-6228342.208	5906983.401
White-naped Crane	-6179342.708	5932050.56
White-naped Crane	-6228278.756	5906932.982
White-naped Crane	-6191654.643	5989037.56
White-naped Crane	-6191734.793	5989090.104
White-naped Crane	-6179362.745	5931838.511
White-naped Crane	-6228538.13	5907014.303
White-naped Crane	-6175383.073	5936664.724
White-naped Crane	-6175353.017	5936658.196
White-naped Crane	-6175356.357	5936638.612
White-naped Crane	-6174488.065	5935509.32
White-naped Crane	-6179358.292	5932107.651
White-naped Crane	-6175350.791	5936654.932
White-naped Crane	-6228561.507	5907017.556
White-naped Crane	-6191729.227	5989101.599
White-naped Crane	-6175364.149	5936658.196
White-naped Crane	-6174482.499	5935509.32
White-naped Crane	-6179357.179	5932106.019

White-naped Crane	-6191732.567	5989103.241
White-naped Crane	-6175361.923	5936672.884
White-naped Crane	-6228560.394	5907025.688
White-naped Crane	-6228557.054	5907020.809
White-naped Crane	-6179357.179	5932099.495
White-naped Crane	-6191733.68	5989101.599
White-naped Crane	-6174482.499	5935496.265
White-naped Crane	-6175348.564	5936650.036
White-naped Crane	-6175398.658	5936654.932
White-naped Crane	-6174460.235	5935545.22
White-naped Crane	-6179354.953	5932102.757
White-naped Crane	-6175369.715	5936643.508
White-naped Crane	-6228557.054	5907025.688
White-naped Crane	-6175358.583	5936640.244
White-naped Crane	-6191731.454	5989099.957
White-naped Crane	-6179371.651	5932104.388
White-naped Crane	-6175357.47	5936646.772
White-naped Crane	-6174459.121	5935558.274
White-naped Crane	-6228554.828	5907024.062
White-naped Crane	-6191730.34	5989104.883
White-naped Crane	-6175340.772	5936648.404
White-naped Crane	-6174473.593	5935550.115
White-naped Crane	-6175361.923	5936645.14
White-naped Crane	-6191731.454	5989109.809
White-naped Crane	-6228555.941	5907020.809
White-naped Crane	-6175368.602	5936645.14
White-naped Crane	-6179367.198	5932115.806
White-naped Crane	-6174460.235	5935566.434
White-naped Crane	-6191733.68	5989111.451
White-naped Crane	-6228553.715	5907025.688
White-naped Crane	-6175365.262	5936643.508
White-naped Crane	-6179364.971	5932114.175
White-naped Crane	-6228554.828	5907024.062
White-naped Crane	-6191730.34	5989104.883
White-naped Crane	-6175364.149	5936648.404
White-naped Crane	-6179362.745	5932115.806
White-naped Crane	-6175367.489	5936645.14
White-naped Crane	-6174456.895	5935558.274
White-naped Crane	-6175364.149	5936638.612
White-naped Crane	-6174440.197	5935551.747
White-naped Crane	-6228533.677	5906905.333
White-naped Crane	-6179348.274	5931952.69
White-naped Crane	-6191737.02	5989108.167
White-naped Crane	-6175366.375	5936638.612
White-naped Crane	-6171662.776	5937412.229
White-naped Crane	-6178447.699	5932755.251
White-naped Crane	-6228411.226	5906919.97
White-naped Crane	-6193643.923	5989894.732
White-naped Crane	-6171902.113	5937541.173
White-naped Crane	-6171806.378	5937508.529
White-naped Crane	-6171911.018	5937531.379
White-naped Crane	-6172158.148	5937502
White-naped Crane	-6193708.488	5989965.346
White-naped Crane	-6178868.487	5932525.241
White-naped Crane	-6228306.586	5906960.631
White-naped Crane	-6171983.376	5937614.622
White-naped Crane	-6178605.773	5932916.751
White-naped Crane	-6191244.988	5989186.984

White-naped Crane	-6173908.09	5934820.72
White-naped Crane	-6173908.09	5934838.669
White-naped Crane	-6172886.177	5945972.36
White-naped Crane	-6178486.661	5933119.038
White-naped Crane	-6173901.411	5934866.407
White-naped Crane	-6173902.524	5934838.669
White-naped Crane	-6191299.534	5989270.729
White-naped Crane	-6173048.704	5946405.328
White-naped Crane	-6229226.085	5906760.583
White-naped Crane	-6179313.764	5931910.281
White-naped Crane	-6191303.987	5989265.802
White-naped Crane	-6229194.915	5906719.923
White-naped Crane	-6175206.075	5933955.969
White-naped Crane	-6173206.777	5946323.634
White-naped Crane	-6172960.761	5931226.861
White-naped Crane	-6173563	5931562.856
White-naped Crane	-6172750.367	5945328.666
White-naped Crane	-6191715.869	5989108.167
White-naped Crane	-6179418.405	5932079.921
White-naped Crane	-6175214.981	5934088.123
White-naped Crane	-6229230.537	5906739.439
White-naped Crane	-6179352.726	5932068.502
White-naped Crane	-6172629.029	5945351.538
White-naped Crane	-6191712.529	5989172.206
White-naped Crane	-6173564.113	5931530.234
White-naped Crane	-6173569.679	5931551.438
White-naped Crane	-6229224.971	5908204.939
White-naped Crane	-6172632.369	5945343.369
White-naped Crane	-6191714.756	5989159.07
White-naped Crane	-6173005.289	5930695.169
White-naped Crane	-6173116.608	5945977.261
White-naped Crane	-6192085.45	5989260.876
White-naped Crane	-6178177.192	5932983.636
White-naped Crane	-6173039.798	5930704.955
White-naped Crane	-6173023.1	5930727.788
White-naped Crane	-6229209.387	5906672.758
White-naped Crane	-6173049.817	5930690.277
White-naped Crane	-6178346.398	5932818.872
White-naped Crane	-6192305.862	5989505.546
White-naped Crane	-6173006.402	5930801.179
White-naped Crane	-6229206.047	5906729.681
White-naped Crane	-6171528.079	5943798.045
White-naped Crane	-6178536.754	5932637.798
White-naped Crane	-6173456.133	5931517.186
White-naped Crane	-6173466.152	5931515.555
White-naped Crane	-6191773.755	5988986.658
White-naped Crane	-6172507.691	5944923.523
White-naped Crane	-6191722.548	5989142.649
White-naped Crane	-6179354.953	5931882.551
White-naped Crane	-6173428.303	5931698.236
White-naped Crane	-6173473.944	5931678.662
White-naped Crane	-6229223.858	5906736.187
White-naped Crane	-6171592.645	5937371.425
White-naped Crane	-6171486.891	5943595.507
White-naped Crane	-6192537.407	5989387.315
White-naped Crane	-6179764.608	5932003.256
White-naped Crane	-6229374.139	5908172.406
White-naped Crane	-6171557.022	5937340.414

White-naped Crane	-6192097.695	5989104.883
White-naped Crane	-6173793.431	5943548.14
White-naped Crane	-6179770.174	5932003.256
White-naped Crane	-6229233.877	5908185.419
White-naped Crane	-6171923.263	5937516.69
White-naped Crane	-6179773.514	5932278.925
White-naped Crane	-6174106.239	5936785.496
White-naped Crane	-6192351.503	5988799.472
White-naped Crane	-6174109.578	5936752.855
White-naped Crane	-6173832.393	5943553.04
White-naped Crane	-6174108.465	5936759.383
White-naped Crane	-6192306.975	5988783.052
White-naped Crane	-6179641.044	5932952.64
White-naped Crane	-6174110.691	5936751.223
White-naped Crane	-6173500.661	5945970.726
White-naped Crane	-6173495.095	5945960.923
White-naped Crane	-6192341.484	5988787.978
White-naped Crane	-6179642.157	5932962.428
White-naped Crane	-6174115.144	5936749.591
White-naped Crane	-6228954.465	5908177.286
White-naped Crane	-6179661.081	5932947.746
White-naped Crane	-6173456.133	5945967.458
White-naped Crane	-6174911.079	5936669.62
White-naped Crane	-6192331.466	5988760.065
White-naped Crane	-6174891.041	5936654.932
White-naped Crane	-6228957.805	5908167.526
White-naped Crane	-6174902.173	5936658.196
White-naped Crane	-6174908.852	5936676.149
White-naped Crane	-6179657.742	5932949.377
White-naped Crane	-6228956.691	5908167.526
White-naped Crane	-6192324.787	5988781.41
White-naped Crane	-6173450.567	5945983.796
White-naped Crane	-6173427.19	5945985.43
White-naped Crane	-6192188.977	5988789.62
White-naped Crane	-6179657.742	5932954.271
White-naped Crane	-6228953.352	5908169.152
White-naped Crane	-6174908.852	5936685.941
White-naped Crane	-6174899.947	5936681.045
White-naped Crane	-6174902.173	5936676.149
White-naped Crane	-6192191.203	5988792.904
White-naped Crane	-6228957.805	5908170.779
White-naped Crane	-6179659.968	5932954.271
White-naped Crane	-6174903.286	5936681.045
White-naped Crane	-6173457.246	5945967.458
White-naped Crane	-6174902.173	5936689.205
White-naped Crane	-6179659.968	5932957.534
White-naped Crane	-6192192.316	5988796.188
White-naped Crane	-6228955.578	5908174.032
White-naped Crane	-6173514.019	5945991.965
White-naped Crane	-6174903.286	5936681.045
White-naped Crane	-6228958.918	5908174.032
White-naped Crane	-6173505.113	5945988.698
White-naped Crane	-6174904.399	5936677.781
White-naped Crane	-6174899.947	5936663.092
White-naped Crane	-6179658.855	5932951.009
White-naped Crane	-6192338.145	5988774.842
White-naped Crane	-6174253.18	5937133.132
White-naped Crane	-6173509.566	5945991.965

White-naped Crane	-6179656.629	5932951.009
White-naped Crane	-6229030.162	5908213.072
White-naped Crane	-6174255.407	5937129.867
White-naped Crane	-6192338.145	5988779.768
White-naped Crane	-6171997.848	5937640.737
White-naped Crane	-6171712.87	5943455.04
White-naped Crane	-6171983.376	5937634.209
White-naped Crane	-6193205.324	5989756.791
White-naped Crane	-6171663.889	5936989.506
White-naped Crane	-6229082.482	5908227.712
White-naped Crane	-6172139.223	5937678.279
White-naped Crane	-6171750.718	5943360.308
White-naped Crane	-6229448.724	5908050.407
White-naped Crane	-6193346.699	5989930.86
White-naped Crane	-6172132.544	5937879.045
White-naped Crane	-6229322.933	5907936.544
White-naped Crane	-6185201.452	5986724.279
White-naped Crane	-6173470.604	5933998.389
White-naped Crane	-6178448.812	5932753.62
White-naped Crane	-6172611.218	5945278.022
White-naped Crane	-6172446.465	5937647.266
White-naped Crane	-6173497.321	5933983.705
White-naped Crane	-6178399.831	5932871.074
White-naped Crane	-6173830.166	5935941.762
White-naped Crane	-6172703.613	5945209.408
White-naped Crane	-6188764.789	5987730.619
White-naped Crane	-6178311.889	5933058.678
White-naped Crane	-6228675.053	5907633.999
White-naped Crane	-6172839.423	5945460.995
White-naped Crane	-6173157.797	5934414.439
White-naped Crane	-6173646.489	5933587.252
White-naped Crane	-6172920.686	5945738.729
White-naped Crane	-6228125.135	5907080.988
White-naped Crane	-6172153.695	5937859.458
White-naped Crane	-6179772.401	5932004.887
White-naped Crane	-6172995.27	5946037.712
White-naped Crane	-6229240.556	5906693.901
White-naped Crane	-6174852.079	5935196.016
White-naped Crane	-6174835.381	5935213.966
White-naped Crane	-6174274.331	5936297.524
White-naped Crane	-6179772.401	5932014.674
White-naped Crane	-6174382.311	5935995.615
White-naped Crane	-6229209.387	5906731.308
White-naped Crane	-6173187.853	5946286.055
White-naped Crane	-6179838.079	5931988.576
White-naped Crane	-6229227.198	5906726.428
White-naped Crane	-6174340.01	5936276.308
White-naped Crane	-6175157.095	5919581.177
White-naped Crane	-6173186.74	5946284.421
White-naped Crane	-6175157.095	5919582.806
White-naped Crane	-6180088.548	5932482.829
White-naped Crane	-6174346.689	5936289.364
White-naped Crane	-6175842.823	5918965.51
White-naped Crane	-6179667.76	5931424.216
White-naped Crane	-6173974.882	5936457.458
White-naped Crane	-6227993.778	5907089.12
White-naped Crane	-6195390.525	5913818.751
White-naped Crane	-6173144.438	5946156.981

White-naped Crane	-6179364.971	5931840.142
White-naped Crane	-6175831.691	5918968.768
White-naped Crane	-6227991.551	5907142.793
White-naped Crane	-6173948.165	5936462.354
White-naped Crane	-6179184.634	5931999.994
White-naped Crane	-6172151.468	5937575.449
White-naped Crane	-6173171.155	5946158.615
White-naped Crane	-6227962.608	5907118.396
White-naped Crane	-6196946.772	5914723.812
White-naped Crane	-6171828.642	5937368.161
White-naped Crane	-6179654.402	5932009.781
White-naped Crane	-6171741.813	5943626.541
White-naped Crane	-6196966.809	5914725.439
White-naped Crane	-6225921.009	5905253.049
White-naped Crane	-6171786.341	5937482.413
White-naped Crane	-6174771.929	5943842.146
White-naped Crane	-6179776.854	5931980.42
White-naped Crane	-6196980.168	5914771.021
White-naped Crane	-6228218.643	5906908.585
White-naped Crane	-6176199.045	5919752.204
White-naped Crane	-6228243.133	5907022.436
White-naped Crane	-6179743.458	5931973.895
White-naped Crane	-6195900.369	5914482.887
White-naped Crane	-6176196.819	5919757.09
White-naped Crane	-6174938.908	5943739.243
White-naped Crane	-6174938.908	5943739.243
White-naped Crane	-6179745.684	5931980.42
White-naped Crane	-6195902.595	5914487.771
White-naped Crane	-6228252.039	5907054.964
White-naped Crane	-6174451.329	5935997.247
White-naped Crane	-6179752.363	5931978.789
White-naped Crane	-6174950.04	5943726.176
White-naped Crane	-6195900.369	5914487.771
White-naped Crane	-6174451.329	5936010.302
White-naped Crane	-6227116.58	5907980.462
White-naped Crane	-6176185.687	5919760.348
White-naped Crane	-6174440.197	5936010.302
White-naped Crane	-6174938.908	5943734.343
White-naped Crane	-6179747.91	5931975.526
White-naped Crane	-6195898.142	5914484.515
White-naped Crane	-6227116.58	5908008.115
White-naped Crane	-6176196.819	5919763.606
White-naped Crane	-6174446.876	5936002.143
White-naped Crane	-6174938.908	5943740.876
White-naped Crane	-6195900.369	5914484.515
White-naped Crane	-6179743.458	5931973.895
White-naped Crane	-6176190.14	5919765.235
White-naped Crane	-6227114.354	5907980.462
White-naped Crane	-6174941.135	5943732.71
White-naped Crane	-6179750.137	5931980.42
White-naped Crane	-6195911.501	5914478.003
White-naped Crane	-6174444.65	5936013.566
White-naped Crane	-6227105.448	5908004.862
White-naped Crane	-6176199.045	5919775.008
White-naped Crane	-6175132.604	5943642.874
White-naped Crane	-6195911.501	5914476.376
White-naped Crane	-6179750.137	5931985.313
White-naped Crane	-6176199.045	5919765.235

White-naped Crane	-6174442.424	5936007.039
White-naped Crane	-6227109.901	5908009.742
White-naped Crane	-6174458.008	5936029.885
White-naped Crane	-6175139.284	5943639.608
White-naped Crane	-6195911.501	5914479.631
White-naped Crane	-6179750.137	5931970.633
White-naped Crane	-6227105.448	5908012.995
White-naped Crane	-6176190.14	5919768.492
White-naped Crane	-6195902.595	5914491.026
White-naped Crane	-6174451.329	5936039.677
White-naped Crane	-6179714.515	5931985.313
White-naped Crane	-6227109.901	5908009.742
White-naped Crane	-6175148.189	5943636.341
White-naped Crane	-6176192.366	5919765.235
White-naped Crane	-6196581.644	5915259.404
White-naped Crane	-6225126.188	5905233.536
White-naped Crane	-6171557.022	5937203.313
White-naped Crane	-6176185.687	5919773.379
White-naped Crane	-6171418.986	5937071.111
White-naped Crane	-6225097.245	5904883.93
White-naped Crane	-6171450.156	5936999.298
White-naped Crane	-6196492.588	5915171.493
White-naped Crane	-6171755.171	5943484.44
White-naped Crane	-6176201.272	5919768.492
White-naped Crane	-6172452.031	5945065.648
White-naped Crane	-6171750.718	5937265.335
White-naped Crane	-6225157.357	5904787.995
White-naped Crane	-6178412.077	5932849.867
White-naped Crane	-6175838.37	5920700.241
White-naped Crane	-6178398.718	5932862.917
White-naped Crane	-6173852.43	5935696.981
White-naped Crane	-6196494.815	5915189.401
White-naped Crane	-6226629.001	5908460.329
White-naped Crane	-6178398.718	5932864.549
White-naped Crane	-6172535.521	5945367.874
White-naped Crane	-6173697.696	5935734.514
White-naped Crane	-6172576.709	5945465.896
White-naped Crane	-6178490	5933039.101
White-naped Crane	-6228512.527	5906884.189
White-naped Crane	-6175818.332	5920718.16
White-naped Crane	-6228514.753	5906989.907
White-naped Crane	-6172605.652	5945470.797
White-naped Crane	-6228483.584	5907019.183
White-naped Crane	-6178238.418	5933270.756
White-naped Crane	-6172730.33	5946060.585
White-naped Crane	-6178080.345	5933107.618
White-naped Crane	-6228461.32	5907048.459
White-naped Crane	-6178033.59	5933107.618
White-naped Crane	-6228521.432	5906851.661
White-naped Crane	-6178454.378	5932614.961
White-naped Crane	-6229227.198	5906676.011
White-naped Crane	-6173162.249	5936543.954
White-naped Crane	-6079731.801	5858945.227
White-naped Crane	-6178398.718	5932867.811
White-naped Crane	-6088944.602	5864883.262
White-naped Crane	-6227873.553	5904755.474
White-naped Crane	-6165815.163	6032777.297
White-naped Crane	-6178469.963	5932696.524

White-naped Crane	-6166149.122	6032838.363
White-naped Crane	-6172349.617	5936405.234
White-naped Crane	-6227134.391	5904696.938
White-naped Crane	-6092324.262	5870208.296
White-naped Crane	-6179892.626	5932101.126
White-naped Crane	-6092916.481	5885297.721
White-naped Crane	-6165955.426	6032889.527
White-naped Crane	-6172175.959	5937484.046
White-naped Crane	-6179977.229	5932287.082
White-naped Crane	-6165964.331	6032897.779
White-naped Crane	-6227310.276	5904661.166
White-naped Crane	-6165991.048	6032879.624
White-naped Crane	-6178930.825	5933047.258
White-naped Crane	-6226384.098	5905066.049
White-naped Crane	-6092858.595	5885550.813
White-naped Crane	-6202305.692	5891674.33
White-naped Crane	-6171390.043	5936707.157
White-naped Crane	-6202305.692	5891687.318
White-naped Crane	-6171418.986	5937049.894
White-naped Crane	-6092802.935	5885591.374
White-naped Crane	-6178405.397	5932851.498
White-naped Crane	-6166137.99	6032820.208
White-naped Crane	-6228503.621	5906867.925
White-naped Crane	-6202316.824	5891716.542
White-naped Crane	-6166135.763	6032877.974
White-naped Crane	-6229189.349	5906651.615
White-naped Crane	-6178398.718	5932864.549
White-naped Crane	-6202321.277	5891714.919
White-naped Crane	-6092809.615	5885591.374
White-naped Crane	-6174270.992	5935985.824
White-naped Crane	-6174266.539	5935987.456
White-naped Crane	-6229189.349	5906651.615
White-naped Crane	-6178387.586	5932880.862
White-naped Crane	-6166122.405	6032846.615
White-naped Crane	-6092809.615	5885597.863
White-naped Crane	-6202296.787	5891706.801
White-naped Crane	-6174255.407	5935967.873
White-naped Crane	-6092811.841	5885596.241
White-naped Crane	-6166122.405	6032846.615
White-naped Crane	-6229198.255	5906648.362
White-naped Crane	-6178387.586	5932875.968
White-naped Crane	-6202316.824	5891701.93
White-naped Crane	-6166122.405	6032841.664
White-naped Crane	-6092809.615	5885589.751
White-naped Crane	-6202319.05	5891703.554
White-naped Crane	-6178387.586	5932875.968
White-naped Crane	-6229196.028	5906646.736
White-naped Crane	-6178385.36	5932882.493
White-naped Crane	-6202319.05	5891701.93
White-naped Crane	-6166120.178	6032848.266
White-naped Crane	-6092807.388	5885599.486
White-naped Crane	-6229191.576	5906658.12
White-naped Crane	-6174150.766	5936016.83
White-naped Crane	-6166124.631	6032849.916
White-naped Crane	-6174157.446	5936015.198
White-naped Crane	-6202321.277	5891703.554
White-naped Crane	-6178385.36	5932882.493
White-naped Crane	-6092807.388	5885602.731

White-naped Crane	-6229198.255	5906654.868
White-naped Crane	-6166106.82	6032856.518
White-naped Crane	-6174155.219	5936005.407
White-naped Crane	-6202316.824	5891708.424
White-naped Crane	-6229193.802	5906654.868
White-naped Crane	-6178383.134	5932871.074
White-naped Crane	-6092811.841	5885592.996
White-naped Crane	-6174150.766	5936016.83
White-naped Crane	-6092814.067	5885586.506
White-naped Crane	-6178389.813	5932872.705
White-naped Crane	-6202321.277	5891698.683
White-naped Crane	-6166102.367	6032851.567
White-naped Crane	-6229202.707	5906646.736
White-naped Crane	-6166100.141	6032849.916
White-naped Crane	-6178398.718	5932862.917
White-naped Crane	-6229209.387	5906679.263
White-naped Crane	-6092816.294	5885756.862
White-naped Crane	-6202310.145	5891710.048
White-naped Crane	-6202307.919	5891710.048
White-naped Crane	-6202305.692	5891711.672
White-naped Crane	-6172069.092	5937865.987
White-naped Crane	-6167921.328	6036859.774
White-naped Crane	-6171470.193	5937315.932
White-naped Crane	-6094599.632	5887502.792
White-naped Crane	-6202301.239	5891718.166
White-naped Crane	-6202301.239	5891716.542
White-naped Crane	-6167729.858	6033992.096
White-naped Crane	-6174760.797	5935289.027
White-naped Crane	-6171514.721	5937281.656
White-naped Crane	-6101806.456	5895076.351
White-naped Crane	-6226168.138	5904913.199
White-naped Crane	-6202303.466	5891716.542
White-naped Crane	-6178469.963	5932655.742
White-naped Crane	-6173899.184	5934846.827
White-naped Crane	-6227283.559	5904687.182
White-naped Crane	-6102953.047	5895279.378
White-naped Crane	-6167433.748	6033599.248
White-naped Crane	-6202285.655	5891740.896
White-naped Crane	-6180487.072	5931967.371
White-naped Crane	-6166356.176	6035106.365
White-naped Crane	-6102933.009	5895243.645
White-naped Crane	-6202301.239	5891693.812
White-naped Crane	-6180654.051	5933306.647
White-naped Crane	-6228009.363	5904869.296
White-naped Crane	-6202229.995	5891633.741
White-naped Crane	-6202223.316	5891636.988
White-naped Crane	-6180651.825	5933308.278
White-naped Crane	-6231006.083	5916654.707
White-naped Crane	-6174210.879	5934863.144
White-naped Crane	-6202218.863	5891638.611
White-naped Crane	-6174319.972	5934863.144
White-naped Crane	-6180647.372	5933305.015
White-naped Crane	-6230217.941	5917293.006
White-naped Crane	-6179155.691	5933541.572
White-naped Crane	-6174440.197	5934724.451
White-naped Crane	-6225782.973	5916830.56
White-naped Crane	-6173696.583	5935832.426
White-naped Crane	-6178260.682	5933164.716

White-naped Crane	-6226223.798	5916483.741
White-naped Crane	-6178233.965	5933138.614
White-naped Crane	-6173750.016	5935791.629
White-naped Crane	-6229202.707	5906684.142
White-naped Crane	-6178205.022	5933112.512
White-naped Crane	-6229187.123	5906680.89
White-naped Crane	-6173761.148	5935791.629
White-naped Crane	-6173761.148	5935793.261
White-naped Crane	-6178176.079	5932975.479
White-naped Crane	-6229298.442	5906744.319
White-naped Crane	-6030962.732	6267921.149
White-naped Crane	-6187164.015	5975618.059
White-naped Crane	-6030880.355	6268024.667
White-naped Crane	-6178245.097	5932946.115
White-naped Crane	-6229231.651	5906765.462
White-naped Crane	-6189955.908	5979771.776
White-naped Crane	-6030524.133	6267612.299
White-naped Crane	-6178142.683	5933133.72
White-naped Crane	-6189960.361	5979788.179
White-naped Crane	-6172075.771	5937877.413
White-naped Crane	-6174086.201	5936694.101
White-naped Crane	-5652084.619	5754143.478
White-naped Crane	-6178129.325	5933045.627
White-naped Crane	-6226257.194	5905018.893
White-naped Crane	-6189470.555	5979914.478
White-naped Crane	-6030987.222	6267936.422
White-naped Crane	-6189459.423	5979922.68
White-naped Crane	-6189461.649	5979916.119
White-naped Crane	-6173903.637	5936674.516
White-naped Crane	-6178418.756	5932854.761
White-naped Crane	-6227276.88	5904679.052
White-naped Crane	-6030964.958	6267914.361
White-naped Crane	-5652075.713	5754185.03
White-naped Crane	-6030969.411	6267910.967
White-naped Crane	-6178423.209	5932861.286
White-naped Crane	-5652069.034	5754197.815
White-naped Crane	-6174186.389	5935770.415
White-naped Crane	-6227279.107	5904679.052
White-naped Crane	-6189461.649	5979912.838
White-naped Crane	-6174197.521	5935758.991
White-naped Crane	-6178420.982	5932846.604
White-naped Crane	-5652069.034	5754191.423
White-naped Crane	-6030962.732	6267900.785
White-naped Crane	-6227292.465	5904649.784
White-naped Crane	-6189463.876	5979912.838
White-naped Crane	-6189468.328	5979911.198
White-naped Crane	-6030969.411	6267900.785
White-naped Crane	-6178416.529	5932846.604
White-naped Crane	-6189466.102	5979914.478
White-naped Crane	-5652062.355	5754197.815
White-naped Crane	-6227281.333	5904666.044
White-naped Crane	-6174190.842	5935754.096
White-naped Crane	-6178416.529	5932849.867
White-naped Crane	-5652062.355	5754196.217
White-naped Crane	-6174190.842	5935752.464
White-naped Crane	-6227281.333	5904664.418
White-naped Crane	-6174193.068	5935752.464
White-naped Crane	-6178416.529	5932853.129

White-naped Crane	-5652062.355	5754188.226
White-naped Crane	-6227281.333	5904667.67
White-naped Crane	-6030973.864	6267910.967
White-naped Crane	-6174195.294	5935757.36
White-naped Crane	-6178423.209	5932856.392
White-naped Crane	-6227299.144	5904661.166
White-naped Crane	-5652060.128	5754188.226
White-naped Crane	-6178420.982	5932849.867
White-naped Crane	-6227299.144	5904661.166
White-naped Crane	-5652066.807	5754188.226
White-naped Crane	-6030962.732	6267893.997
White-naped Crane	-6189470.555	5979904.637
White-naped Crane	-6189475.008	5979906.277
White-naped Crane	-6178329.7	5932875.968
White-naped Crane	-5651968.846	5754010.834
White-naped Crane	-6174168.578	5935762.255
White-naped Crane	-6189301.349	5979944.003
White-naped Crane	-6189301.349	5979944.003
White-naped Crane	-6030960.505	6267899.088
White-naped Crane	-6172044.602	5937861.09
White-naped Crane	-6170895.785	5936550.482
White-naped Crane	-5652044.544	5753994.853
White-naped Crane	-6189312.481	5979950.564
White-naped Crane	-6189312.481	5979942.363
White-naped Crane	-6172147.016	5937771.316
White-naped Crane	-6170929.18	5936522.738
White-naped Crane	-5652075.713	5753945.311
White-naped Crane	-6226493.191	5905041.657
White-naped Crane	-6030922.657	6267900.785
White-naped Crane	-6189310.255	5979948.924
White-naped Crane	-6189312.481	5979955.485
White-naped Crane	-6170906.917	5936568.435
White-naped Crane	-6030951.6	6267936.422
White-naped Crane	-5652080.166	5753953.302
White-naped Crane	-6227288.012	5904685.556
White-naped Crane	-6174400.122	5935088.321
White-naped Crane	-6189314.708	5979950.564
White-naped Crane	-6030708.923	6268718.782
White-naped Crane	-6175288.452	5932988.53
White-naped Crane	-6178841.77	5932889.018
White-naped Crane	-6227310.276	5904770.108
White-naped Crane	-5652057.902	5754205.806
White-naped Crane	-6227893.59	5904726.206
White-naped Crane	-6189301.349	5979968.607
White-naped Crane	-6228223.096	5904935.964
White-naped Crane	-6178781.657	5932900.438
White-naped Crane	-6006634.97	6256111.51
White-naped Crane	-6189236.784	5979983.37
White-naped Crane	-6178701.507	5932954.271
White-naped Crane	-6006697.309	6256113.205
White-naped Crane	-6189274.633	5979968.607
White-naped Crane	-6173442.775	5947029.494
White-naped Crane	-6178536.754	5932818.872
White-naped Crane	-6189274.633	5979965.327
White-naped Crane	-6227283.559	5904687.182
White-naped Crane	-6178478.868	5932766.67
White-naped Crane	-6174466.914	5936085.371
White-naped Crane	-6189497.271	5979922.68

White-naped Crane	-6008262.461	6256760.573
White-naped Crane	-6173950.391	5935710.036
White-naped Crane	-6178607.999	5932851.498
White-naped Crane	-6227167.787	5904513.201
White-naped Crane	-6189381.499	5980096.55
White-naped Crane	-6178659.206	5932952.64
White-naped Crane	-5332285.985	5591244.141
White-naped Crane	-6174070.616	5935600.702
White-naped Crane	-6227118.807	5904518.079
White-naped Crane	-6189421.574	5980042.42
White-naped Crane	-6030312.626	6269665.858
White-naped Crane	-6227132.165	5904628.646
White-naped Crane	-6178705.96	5932753.62
White-naped Crane	-6174028.315	5935718.195
White-naped Crane	-5332279.306	5591247.279
White-naped Crane	-6189439.385	5979870.191
White-naped Crane	-6178868.487	5932548.079
White-naped Crane	-6029597.955	6267495.21
White-naped Crane	-6189434.933	5979875.112
White-naped Crane	-6227158.882	5904640.028
White-naped Crane	-5332653.34	5591211.193
White-naped Crane	-6179271.463	5932632.905
White-naped Crane	-6029613.54	6267493.513
White-naped Crane	-6226468.701	5904443.285
White-naped Crane	-6172158.148	5937888.839
White-naped Crane	-6187393.333	5976226.315
White-naped Crane	-6189089.842	5979834.106
White-naped Crane	-6031955.702	6265706.843
White-naped Crane	-6174010.504	5935546.852
White-naped Crane	-5332272.627	5591241.003
White-naped Crane	-6178607.999	5933396.374
White-naped Crane	-6226824.923	5904358.736
White-naped Crane	-6189114.332	5979817.703
White-naped Crane	-6188822.675	5979309.242
White-naped Crane	-6174504.762	5935430.993
White-naped Crane	-6032518.978	6264777.179
White-naped Crane	-5332263.722	5591278.659
White-naped Crane	-6226831.602	5904365.24
White-naped Crane	-6178407.624	5932864.549
White-naped Crane	-6188811.543	5979266.598
White-naped Crane	-6032516.752	6264782.269
White-naped Crane	-6174551.517	5935424.466
White-naped Crane	-5332263.722	5591278.659
White-naped Crane	-6178407.624	5932862.917
White-naped Crane	-6188811.543	5979266.598
White-naped Crane	-6226827.15	5904355.484
White-naped Crane	-6178400.945	5932862.917
White-naped Crane	-6032400.98	6264750.038
White-naped Crane	-5332263.722	5591272.383
White-naped Crane	-6188822.675	5979255.117
White-naped Crane	-6226831.602	5904352.232
White-naped Crane	-6174547.064	5935395.093
White-naped Crane	-6032525.658	6264775.483
White-naped Crane	-6174533.706	5935396.725
White-naped Crane	-5332263.722	5591273.952
White-naped Crane	-6178398.718	5932864.549
White-naped Crane	-6226824.923	5904350.606
White-naped Crane	-6188780.374	5979227.235

White-naped Crane	-6178396.492	5932867.811
White-naped Crane	-5332259.269	5591275.521
White-naped Crane	-6032521.205	6264772.09
White-naped Crane	-6188809.317	5979271.518
White-naped Crane	-6174538.158	5935399.989
White-naped Crane	-6226824.923	5904348.98
White-naped Crane	-6034142.017	6263184.437
White-naped Crane	-6174542.611	5935399.989
White-naped Crane	-6178400.945	5932862.917
White-naped Crane	-6226827.15	5904357.11
White-naped Crane	-5332259.269	5591270.814
White-naped Crane	-6188800.411	5979268.238
White-naped Crane	-6034139.79	6263196.31
White-naped Crane	-6174544.837	5935399.989
White-naped Crane	-6178392.039	5932859.655
White-naped Crane	-6226829.376	5904353.858
White-naped Crane	-5332257.042	5591286.505
White-naped Crane	-6188820.449	5979264.958
White-naped Crane	-6178394.265	5932856.392
White-naped Crane	-6226829.376	5904345.728
White-naped Crane	-6034135.337	6263177.653
White-naped Crane	-5332243.684	5591256.693
White-naped Crane	-6188811.543	5979256.757
White-naped Crane	-6174538.158	5935399.989
White-naped Crane	-6178467.736	5932879.23
White-naped Crane	-6174535.932	5935398.357
White-naped Crane	-6226831.602	5904358.736
White-naped Crane	-6031412.463	6266288.784
White-naped Crane	-5332297.117	5591273.952
White-naped Crane	-6032069.248	6265191.105
White-naped Crane	-6172082.45	5937888.839
White-naped Crane	-6178380.907	5932672.055
White-naped Crane	-5332308.249	5591253.555
White-naped Crane	-6172334.032	5937730.51
White-naped Crane	-6032075.927	6265208.069
White-naped Crane	-6228521.432	5907033.821
White-naped Crane	-6178944.184	5932174.529
White-naped Crane	-6030310.4	6267673.389
White-naped Crane	-6228519.206	5907015.93
White-naped Crane	-5332103.421	5589560.764
White-naped Crane	-6190082.812	5979697.966
White-naped Crane	-6178445.472	5932610.067
White-naped Crane	-6228530.338	5906876.057
White-naped Crane	-5332107.874	5589519.978
White-naped Crane	-6173999.372	5934766.874
White-naped Crane	-6189617.497	5980037.499
White-naped Crane	-6178944.184	5932203.89
White-naped Crane	-6229391.951	5908191.926
White-naped Crane	-6174435.744	5934530.285
White-naped Crane	-5332497.492	5592640.66
White-naped Crane	-6189619.723	5980004.693
White-naped Crane	-6178915.241	5932159.848
White-naped Crane	-5334116.078	5594500.408
White-naped Crane	-6228441.282	5906934.608
White-naped Crane	-6173493.982	5931730.858
White-naped Crane	-5976629.915	6356503.891
White-naped Crane	-5332312.702	5594324.618
White-naped Crane	-6227212.315	5903495.405

White-naped Crane	-6173465.038	5931659.089
White-naped Crane	-6189989.304	5979958.766
White-naped Crane	-5976623.236	6356533.049
White-naped Crane	-6173013.081	5930737.573
White-naped Crane	-6178242.871	5932791.14
White-naped Crane	-5332390.626	5594324.618
White-naped Crane	-6227207.862	5903396.233
White-naped Crane	-6189991.53	5979955.485
White-naped Crane	-5332390.626	5594324.618
White-naped Crane	-6189993.756	5979957.125
White-naped Crane	-6227058.694	5904787.995
White-naped Crane	-5976641.047	6356503.891
White-naped Crane	-6173004.176	5930641.35
White-naped Crane	-5976614.33	6356509.037
White-naped Crane	-6173021.987	5930607.101
White-naped Crane	-6178955.316	5932189.21
White-naped Crane	-5332370.588	5594566.33
White-naped Crane	-6178937.505	5932203.89
White-naped Crane	-6172910.667	5930559.805
White-naped Crane	-6229988.623	5909501.487
White-naped Crane	-5332388.399	5594561.622
White-naped Crane	-5976634.368	6356459.297
White-naped Crane	-5976634.368	6356557.061
White-naped Crane	-6173015.308	5930636.457
White-naped Crane	-6180055.152	5931264.375
White-naped Crane	-6228461.32	5907014.303
White-naped Crane	-5333855.59	5590268.277
White-naped Crane	-6189889.116	5979883.313
White-naped Crane	-5978818.456	6351128.613
White-naped Crane	-6179329.349	5931859.716
White-naped Crane	-6189831.23	5979857.069
White-naped Crane	-5333811.062	5590189.836
White-naped Crane	-6173010.855	5930662.551
White-naped Crane	-6171888.754	5937612.99
White-naped Crane	-6179358.292	5931884.183
White-naped Crane	-5333935.74	5590070.607
White-naped Crane	-6228301.02	5907027.315
White-naped Crane	-5978992.114	6350881.794
White-naped Crane	-6189216.746	5979986.65
White-naped Crane	-6179367.198	5931889.076
White-naped Crane	-6228223.096	5906919.97
White-naped Crane	-6189218.973	5979980.089
White-naped Crane	-6174386.764	5946357.945
White-naped Crane	-6189241.237	5979981.729
White-naped Crane	-5978983.209	6349500.428
White-naped Crane	-6173237.947	5946202.729
White-naped Crane	-6179153.464	5931986.944
White-naped Crane	-6189236.784	5979975.168
White-naped Crane	-5332259.269	5591294.35
White-naped Crane	-6228256.492	5906970.39
White-naped Crane	-6189243.463	5979996.492
White-naped Crane	-5978994.341	6349497
White-naped Crane	-6173226.815	5946189.658
White-naped Crane	-6228249.813	5906970.39
White-naped Crane	-5332261.495	5591294.35
White-naped Crane	-6179160.144	5931988.576
White-naped Crane	-6189270.18	5980060.463
White-naped Crane	-5978985.435	6349507.283

White-naped Crane	-5332263.722	5591284.936
White-naped Crane	-6179157.917	5931985.313
White-naped Crane	-6228252.039	5906973.642
White-naped Crane	-6173229.041	5946192.926
White-naped Crane	-6189214.52	5979945.643
White-naped Crane	-6173286.927	5945926.613
White-naped Crane	-5332254.816	5591286.505
White-naped Crane	-6179153.464	5931985.313
White-naped Crane	-6228252.039	5906965.51
White-naped Crane	-6173291.38	5945944.585
White-naped Crane	-5332245.91	5591278.659
White-naped Crane	-6179142.332	5931986.944
White-naped Crane	-5978963.171	6349507.283
White-naped Crane	-6228247.586	5906970.39
White-naped Crane	-6179153.464	5932003.256
White-naped Crane	-5332250.363	5591270.814
White-naped Crane	-5978978.756	6349507.283
White-naped Crane	-6173280.248	5945938.05
White-naped Crane	-6228252.039	5906965.51
White-naped Crane	-5978978.756	6349503.855
White-naped Crane	-6173286.927	5945931.515
White-naped Crane	-6228252.039	5906962.257
White-naped Crane	-6179164.596	5931986.944
White-naped Crane	-6179169.049	5931980.42
White-naped Crane	-5332254.816	5591269.245
White-naped Crane	-5978978.756	6349500.428
White-naped Crane	-6228203.058	5906975.269
White-naped Crane	-6173280.248	5945910.275
White-naped Crane	-5978905.285	6349503.855
White-naped Crane	-6171637.172	5944769.966
White-naped Crane	-6179017.655	5931903.756
White-naped Crane	-5332936.091	5589347.423
White-naped Crane	-6228209.738	5907001.292
White-naped Crane	-5978938.681	6349524.42
White-naped Crane	-6171692.832	5937454.666
White-naped Crane	-5332858.168	5589331.736
White-naped Crane	-6189960.361	5979804.581
White-naped Crane	-6190031.605	5979812.782
White-naped Crane	-6171583.739	5937374.689
White-naped Crane	-6190047.19	5979804.581
White-naped Crane	-5979001.02	6349526.134
White-naped Crane	-5332851.488	5589578.02
White-naped Crane	-6226361.834	5905001.006
White-naped Crane	-6178877.392	5932923.276
White-naped Crane	-6172690.255	5945204.507
White-naped Crane	-5332871.526	5589272.127
White-naped Crane	-6178340.832	5933456.737
White-naped Crane	-5332241.458	5591088.812
White-naped Crane	-5978983.209	6349464.44
White-naped Crane	-6173756.696	5936119.641
White-naped Crane	-6178337.493	5933402.9
White-naped Crane	-5979479.694	6347687.52
White-naped Crane	-6173880.26	5935718.195
White-naped Crane	-6178913.014	5932112.544
White-naped Crane	-5332624.397	5589491.741
White-naped Crane	-6229178.217	5906879.31
White-naped Crane	-6189196.709	5979962.046
White-naped Crane	-6229148.161	5906900.453

White-naped Crane	-6189236.784	5979942.363
White-naped Crane	-6178786.11	5931556.331
White-naped Crane	-6229084.709	5906879.31
White-naped Crane	-5332628.849	5589510.566
White-naped Crane	-6173931.467	5935458.733
White-naped Crane	-6178516.717	5931799.364
White-naped Crane	-6229246.122	5906728.055
White-naped Crane	-5330113.029	5585555.194
White-naped Crane	-6189245.689	5979937.442
White-naped Crane	-6172959.648	5930603.839
White-naped Crane	-6178915.241	5932146.799
White-naped Crane	-5329905.975	5585167.906
White-naped Crane	-5845121.521	6333171.116
White-naped Crane	-6189234.558	5979930.881
White-naped Crane	-6229312.914	5906734.56
White-naped Crane	-6178955.316	5932145.168
White-naped Crane	-5845219.482	6333183.088
White-naped Crane	-6189243.463	5979922.68
White-naped Crane	-5330044.011	5585442.299
White-naped Crane	-6229216.066	5906689.022
White-naped Crane	-6172932.931	5930395.088
White-naped Crane	-5845003.523	6333270.316
White-naped Crane	-6172966.327	5930417.92
White-naped Crane	-6178916.354	5932130.487
White-naped Crane	-6229206.047	5906906.959
White-naped Crane	-5330155.33	5585563.034
White-naped Crane	-6189203.388	5979950.564
White-naped Crane	-5845190.539	6333222.426
White-naped Crane	-5330050.69	5585363.9
White-naped Crane	-6172980.799	5930660.92
White-naped Crane	-6178938.618	5932197.366
White-naped Crane	-6189210.067	5979947.284
White-naped Crane	-6178963.108	5932213.677
White-naped Crane	-5845181.634	6333215.585
White-naped Crane	-5329892.616	5584978.189
White-naped Crane	-6228083.947	5907030.568
White-naped Crane	-6173028.666	5930625.041
White-naped Crane	-6172224.939	5937825.181
White-naped Crane	-5330411.365	5584854.325
White-naped Crane	-6178354.19	5932619.854
White-naped Crane	-6228305.472	5907040.326
White-naped Crane	-6189962.587	5980012.895
White-naped Crane	-6172100.261	5937968.82
White-naped Crane	-6178372.002	5932579.073
White-naped Crane	-6228464.659	5906872.804
White-naped Crane	-5330587.25	5585425.051
White-naped Crane	-5841118.472	6327742.541
White-naped Crane	-5841988.991	6321465.201
White-naped Crane	-6178366.436	5932642.692
White-naped Crane	-5330939.02	5585426.619
White-naped Crane	-6228194.153	5906937.861
White-naped Crane	-6173913.656	5936225.718
White-naped Crane	-6188054.571	5977178.952
White-naped Crane	-6173934.807	5936225.718
White-naped Crane	-6188054.571	5977175.673
White-naped Crane	-6228210.851	5906911.838
White-naped Crane	-6178366.436	5932649.217
White-naped Crane	-5330932.34	5585410.939

White-naped Crane	-6173914.769	5936212.662
White-naped Crane	-5330945.699	5585421.915
White-naped Crane	-5842211.63	6329865.675
White-naped Crane	-6188047.892	5977164.195
White-naped Crane	-6178366.436	5932647.586
White-naped Crane	-6228216.417	5906913.465
White-naped Crane	-6173921.448	5936207.766
White-naped Crane	-6188052.345	5977169.114
White-naped Crane	-5330947.925	5585407.803
White-naped Crane	-6228209.738	5906924.85
White-naped Crane	-6178363.096	5932645.955
White-naped Crane	-5842162.649	6329886.191
White-naped Crane	-6173924.788	5936204.502
White-naped Crane	-5330950.152	5585401.531
White-naped Crane	-6188054.571	5977167.474
White-naped Crane	-6178364.209	5932645.955
White-naped Crane	-5842209.403	6329870.804
White-naped Crane	-6228211.964	5906924.85
White-naped Crane	-5842220.535	6329865.675
White-naped Crane	-6173924.788	5936207.766
White-naped Crane	-5330947.925	5585406.235
White-naped Crane	-6178365.322	5932645.955
White-naped Crane	-6228209.738	5906928.102
White-naped Crane	-6173927.014	5936209.398
White-naped Crane	-5842191.592	6329874.223
White-naped Crane	-5330950.152	5585407.803
White-naped Crane	-6228228.662	5906981.775
White-naped Crane	-6178365.322	5932634.536
White-naped Crane	-6228249.813	5906965.51
White-naped Crane	-5330950.152	5585406.235
White-naped Crane	-5842193.819	6329872.513
White-naped Crane	-6173910.316	5936201.238
White-naped Crane	-6178363.096	5932654.111
White-naped Crane	-5842200.498	6329874.223
White-naped Crane	-5330950.152	5585403.099
White-naped Crane	-6228256.492	5906970.39
White-naped Crane	-6178363.096	5932649.217
White-naped Crane	-6173921.448	5936211.03
White-naped Crane	-6173923.675	5936212.662
White-naped Crane	-6178361.983	5932645.955
White-naped Crane	-5330954.604	5585530.106
White-naped Crane	-6228206.398	5907033.821
White-naped Crane	-5842196.045	6329874.223
White-naped Crane	-5842383.062	6329745.999
White-naped Crane	-6173924.788	5936214.294
White-naped Crane	-6227963.722	5907100.505
White-naped Crane	-6178321.908	5932900.438
White-naped Crane	-5331079.282	5585537.946
White-naped Crane	-5842567.852	6329453.655
White-naped Crane	-6177991.289	5932773.195
White-naped Crane	-6172012.319	5937921.484
White-naped Crane	-5331010.264	5585552.058
White-naped Crane	-6228020.494	5907123.275
White-naped Crane	-6171990.055	5937905.161
White-naped Crane	-6174626.101	5934416.071
White-naped Crane	-6228304.359	5907048.459
White-naped Crane	-5843244.675	6322006.636
White-naped Crane	-6171666.115	5937430.183

White-naped Crane	-6172071.318	5937784.374
White-naped Crane	-6228467.999	5906882.563
White-naped Crane	-5332179.119	5587896.525
White-naped Crane	-6172066.866	5937777.845
White-naped Crane	-5868360.578	6317065.003
White-naped Crane	-6228441.282	5906929.729
White-naped Crane	-6171682.813	5937549.334
White-naped Crane	-5332377.267	5587858.883
White-naped Crane	-6171652.757	5937554.23
White-naped Crane	-5330023.973	5584982.892
White-naped Crane	-6228441.282	5906945.993
White-naped Crane	-5868355.012	6317054.761
White-naped Crane	-5332800.281	5587515.409
White-naped Crane	-6172052.394	5937413.862
White-naped Crane	-6171656.097	5937544.437
White-naped Crane	-6228440.169	5906928.102
White-naped Crane	-5868349.446	6317076.952
White-naped Crane	-6172212.694	5937627.68
White-naped Crane	-5332775.791	5587455.812
White-naped Crane	-6228505.847	5906893.948
White-naped Crane	-5868344.993	6317056.468
White-naped Crane	-6172133.657	5937727.246
White-naped Crane	-5868349.446	6317044.518
White-naped Crane	-6178363.096	5932642.692
White-naped Crane	-5332715.679	5587407.194
White-naped Crane	-6172247.203	5937544.437
White-naped Crane	-6178359.756	5932645.955
White-naped Crane	-5868105.656	6317117.921
White-naped Crane	-5332833.677	5587397.784
White-naped Crane	-6229168.198	5906841.902
White-naped Crane	-6172404.164	5937072.743
White-naped Crane	-5332742.395	5587142.151
White-naped Crane	-6178358.643	5932647.586
White-naped Crane	-5868332.748	6317068.417
White-naped Crane	-6229180.444	5906877.684
White-naped Crane	-5868349.446	6317070.124
White-naped Crane	-6178355.304	5932621.486
White-naped Crane	-5332241.458	5591241.003
White-naped Crane	-6172355.183	5936994.402
White-naped Crane	-6229178.217	5906843.529
White-naped Crane	-5868332.748	6317056.468
White-naped Crane	-6171855.359	5937723.981
White-naped Crane	-6228211.964	5906926.476
White-naped Crane	-6178431.001	5932768.301
White-naped Crane	-5332330.513	5591269.245
White-naped Crane	-6172528.842	5937040.101
White-naped Crane	-6178366.436	5932637.798
White-naped Crane	-6228483.584	5906893.948
White-naped Crane	-5332201.383	5589576.451
White-naped Crane	-5868872.648	6318056.851
White-naped Crane	-5868338.314	6317071.831
White-naped Crane	-5332250.363	5591248.848
White-naped Crane	-6228482.47	5906835.397
White-naped Crane	-6178377.568	5932644.324
White-naped Crane	-6173849.091	5936579.859
White-naped Crane	-6174437.971	5936256.725
White-naped Crane	-5868357.238	6317097.437
White-naped Crane	-6178366.436	5932647.586

White-naped Crane	-5332245.91	5591248.848
White-naped Crane	-6228553.715	5907035.447
White-naped Crane	-6228553.715	5907033.821
White-naped Crane	-5332237.005	5591261.4
White-naped Crane	-6178365.322	5932639.43
White-naped Crane	-5868353.899	6317095.73
White-naped Crane	-5868362.804	6317073.538
White-naped Crane	-6174372.292	5936446.034
White-naped Crane	-5332234.778	5591258.262
White-naped Crane	-6178364.209	5932644.324
White-naped Crane	-6228550.375	5907030.568
White-naped Crane	-5868361.691	6317105.972
White-naped Crane	-6174367.84	5936457.458
White-naped Crane	-6178365.322	5932644.324
White-naped Crane	-6228553.715	5907040.326
White-naped Crane	-5330939.02	5585415.643
White-naped Crane	-6174368.953	5936462.354
White-naped Crane	-6178359.756	5932647.586
White-naped Crane	-6228519.206	5906916.718
White-naped Crane	-5330936.793	5585420.347
White-naped Crane	-5868341.654	6317095.73
White-naped Crane	-5868337.201	6317097.437
White-naped Crane	-6178361.983	5932642.692
White-naped Crane	-6174375.632	5936454.194
White-naped Crane	-5330939.02	5585415.643
White-naped Crane	-6228522.545	5906913.465
White-naped Crane	-6178361.983	5932639.43
White-naped Crane	-5330943.472	5585417.211
White-naped Crane	-6174371.179	5936457.458
White-naped Crane	-5868352.786	6317097.437
White-naped Crane	-6228519.206	5906905.333
White-naped Crane	-6174364.5	5936459.09
White-naped Crane	-6178361.983	5932641.061
White-naped Crane	-5330943.472	5585412.507
White-naped Crane	-6228515.866	5906908.585
White-naped Crane	-6174302.161	5936398.706
White-naped Crane	-6178364.209	5932662.268
White-naped Crane	-5332468.549	5587233.112
White-naped Crane	-5868367.257	6317073.538
White-naped Crane	-6228467.999	5906931.355
White-naped Crane	-5868468.558	6317053.054
White-naped Crane	-6171686.153	5937652.163
White-naped Crane	-6178363.096	5932637.798
White-naped Crane	-5332490.813	5587231.543
White-naped Crane	-6228442.395	5906911.838
White-naped Crane	-5869326.831	6316894.301
White-naped Crane	-6172527.728	5937028.676
White-naped Crane	-6228455.754	5907097.252
White-naped Crane	-5329819.146	5585992.677
White-naped Crane	-6178549	5932761.776
White-naped Crane	-5329848.089	5584750.846
White-naped Crane	-6172547.766	5937066.215
White-naped Crane	-6228519.206	5907102.132
White-naped Crane	-5879574.904	6312827.508
White-naped Crane	-6178476.642	5932913.488
White-naped Crane	-6173578.584	5936153.912
White-naped Crane	-6228362.245	5907113.517
White-naped Crane	-6189495.045	5980275.344

White-naped Crane	-5879873.24	6313184.104
White-naped Crane	-6173056.496	5934174.596
White-naped Crane	-6227993.778	5907090.746
White-naped Crane	-6178438.793	5932562.76
White-naped Crane	-5332657.793	5587694.202
White-naped Crane	-6178358.643	5932637.798
White-naped Crane	-5880384.196	6312991.301
White-naped Crane	-6173940.373	5934381.807
White-naped Crane	-6227574.103	5907606.348
White-naped Crane	-5332660.019	5587694.202
White-naped Crane	-5332715.679	5588197.665
White-naped Crane	-6173935.92	5934386.702
White-naped Crane	-5879833.165	6313250.648
White-naped Crane	-6227575.217	5907638.879
White-naped Crane	-6178364.209	5932642.692
White-naped Crane	-5880024.634	6313097.087
White-naped Crane	-5332708.999	5588029.841
White-naped Crane	-6173981.561	5934438.914
White-naped Crane	-6227756.667	5907669.783
White-naped Crane	-6172482.087	5937020.516
White-naped Crane	-6178176.079	5932840.079
White-naped Crane	-6228494.716	5907001.292
White-naped Crane	-5880405.347	6312940.115
White-naped Crane	-5332234.778	5591262.969
White-naped Crane	-6178360.87	5932647.586
White-naped Crane	-5332248.137	5591281.797
White-naped Crane	-6172459.823	5937043.365
White-naped Crane	-6174023.862	5935316.767
White-naped Crane	-6178351.964	5932675.318
White-naped Crane	-5874293.907	6283306.826
White-naped Crane	-5332228.099	5591226.882
White-naped Crane	-6177842.121	5932546.448
White-naped Crane	-6174080.635	5935383.671
White-naped Crane	-5874257.171	6283281.324
White-naped Crane	-6228840.919	5906568.67
White-naped Crane	-6174010.504	5933743.873
White-naped Crane	-6178364.209	5932639.43
White-naped Crane	-6229223.858	5906732.934
White-naped Crane	-5874741.411	6282458.5
White-naped Crane	-5332330.513	5589584.295
White-naped Crane	-6171892.094	5937774.581
White-naped Crane	-6229167.085	5906726.428
White-naped Crane	-5872048.593	6254249.334
White-naped Crane	-5330313.404	5585053.448
White-naped Crane	-5330478.157	5585075.399
White-naped Crane	-6178562.358	5933102.724
White-naped Crane	-6173975.995	5935943.394
White-naped Crane	-6174252.067	5935998.879
White-naped Crane	-5330544.949	5585141.251
White-naped Crane	-6229192.689	5906659.747
White-naped Crane	-6178364.209	5932645.955
White-naped Crane	-6178367.549	5932641.061
White-naped Crane	-6229160.406	5906724.802
White-naped Crane	-5330558.307	5585127.14
White-naped Crane	-5870653.76	6249804.831
White-naped Crane	-6178365.322	5932641.061
White-naped Crane	-6229248.348	5906854.914
White-naped Crane	-5330549.401	5585139.684

White-naped Crane	-6174265.426	5935997.247
White-naped Crane	-6174262.086	5936010.302
White-naped Crane	-6178368.662	5932655.742
White-naped Crane	-5870659.326	6249799.751
White-naped Crane	-5330562.76	5585128.708
White-naped Crane	-6229243.896	5906848.408
White-naped Crane	-5870653.76	6249818.378
White-naped Crane	-6174264.312	5935997.247
White-naped Crane	-5330556.081	5585134.98
White-naped Crane	-6178369.775	5932654.111
White-naped Crane	-6229240.556	5906853.287
White-naped Crane	-5330553.854	5585133.412
White-naped Crane	-6229243.896	5906858.167
White-naped Crane	-6178370.888	5932670.424
White-naped Crane	-5870654.873	6249804.831
White-naped Crane	-6174264.312	5936013.566
White-naped Crane	-5870649.307	6249806.524
White-naped Crane	-5330558.307	5585141.251
White-naped Crane	-6178370.888	5932654.111
White-naped Crane	-6229240.556	5906846.782
White-naped Crane	-6174266.539	5936007.039
White-naped Crane	-6174273.218	5935998.879
White-naped Crane	-5330553.854	5585138.116
White-naped Crane	-5870654.873	6249799.751
White-naped Crane	-6178369.775	5932647.586
White-naped Crane	-6229241.669	5906858.167
White-naped Crane	-5869195.474	6251479.711
White-naped Crane	-6174277.671	5935998.879
White-naped Crane	-6178372.002	5932647.586
White-naped Crane	-6229114.765	5906854.914
White-naped Crane	-5329681.109	5585802.941
White-naped Crane	-6171771.869	5937781.11
White-naped Crane	-6177995.742	5932980.373
White-naped Crane	-5332363.909	5587498.157
White-naped Crane	-6229161.519	5906645.109
White-naped Crane	-5332167.987	5587560.891
White-naped Crane	-6228789.712	5906386.521
White-naped Crane	-5863936.742	6265864.625
White-naped Crane	-5863927.836	6265857.839
White-naped Crane	-6177504.823	5932497.51
White-naped Crane	-5332245.91	5587607.942
White-naped Crane	-6225497.995	5905553.884
White-naped Crane	-6172679.123	5937102.121
White-naped Crane	-6178364.209	5932641.061
White-naped Crane	-5863928.949	6265844.266
White-naped Crane	-5329703.373	5585853.119
White-naped Crane	-6228700.657	5906545.901
White-naped Crane	-5863823.196	6265730.595
White-naped Crane	-5328990.928	5586060.104
White-naped Crane	-6171717.322	5937725.613
White-naped Crane	-6228725.147	5906503.616
White-naped Crane	-6178321.908	5932892.281
White-naped Crane	-6178288.512	5932703.05
White-naped Crane	-5864489.999	6268706.902
White-naped Crane	-6227768.912	5904905.069
White-naped Crane	-6174088.428	5934732.609
White-naped Crane	-5329100.022	5586133.805
White-naped Crane	-5879667.299	6258936.912

White-naped Crane	-5329242.511	5586035.015
White-naped Crane	-6178319.681	5932908.594
White-naped Crane	-6178360.87	5932642.692
White-naped Crane	-6228302.133	5907035.447
White-naped Crane	-5329787.976	5585878.208
White-naped Crane	-5879378.981	6259275.957
White-naped Crane	-6174229.803	5934934.939
White-naped Crane	-6174154.106	5935158.486
White-naped Crane	-5899300.717	6238845.935
White-naped Crane	-6228380.056	5907095.626
White-naped Crane	-5330569.439	5585127.14
White-naped Crane	-5906715.709	6241251.061
White-naped Crane	-6173977.108	5935533.797
White-naped Crane	-6228456.867	5906994.786
White-naped Crane	-6174010.504	5935584.384
White-naped Crane	-5331275.204	5585677.499
White-naped Crane	-6228501.395	5907001.292
White-naped Crane	-6178355.304	5932650.849
White-naped Crane	-6178358.643	5932644.324
White-naped Crane	-6228508.074	5906980.148
White-naped Crane	-5330622.872	5583789.8
White-naped Crane	-6173955.957	5935654.553
White-naped Crane	-6171766.303	5937790.903
White-naped Crane	-6226330.665	5905083.935
White-naped Crane	-6178606.886	5933146.771
White-naped Crane	-5961996.968	6238957.55
White-naped Crane	-5332179.119	5584680.293
White-naped Crane	-6171806.378	5937817.019
White-naped Crane	-6226005.612	5905287.197
White-naped Crane	-5332176.892	5584708.514
White-naped Crane	-5961494.917	6238243.915
White-naped Crane	-6178503.359	5933050.521
White-naped Crane	-6173753.356	5936204.502
White-naped Crane	-6225588.164	5904948.972
White-naped Crane	-5329781.297	5585842.143
White-naped Crane	-6178363.096	5932649.217
White-naped Crane	-5946736.179	6239630.654
White-naped Crane	-6174079.522	5936261.621
White-naped Crane	-6228493.602	5906928.102
White-naped Crane	-5330418.044	5584689.7
White-naped Crane	-6178366.436	5932647.586
White-naped Crane	-5946730.613	6239622.197
White-naped Crane	-6178372.002	5932645.955
White-naped Crane	-5330424.724	5584694.403
White-naped Crane	-6228494.716	5906919.97
White-naped Crane	-6174115.144	5936282.836
White-naped Crane	-5330426.95	5584689.7
White-naped Crane	-5946735.066	6239632.345
White-naped Crane	-6178376.454	5932647.586
White-naped Crane	-6228493.602	5906919.97
White-naped Crane	-5946725.047	6239628.963
White-naped Crane	-6174095.107	5936284.468
White-naped Crane	-5330422.497	5584686.564
White-naped Crane	-6178372.002	5932641.061
White-naped Crane	-6228489.15	5906915.091
White-naped Crane	-6174095.107	5936286.1
White-naped Crane	-5330424.724	5584689.7
White-naped Crane	-5946733.952	6239630.654

White-naped Crane	-6228493.602	5906924.85
White-naped Crane	-6178359.756	5932644.324
White-naped Crane	-5946728.386	6239639.11
White-naped Crane	-5330420.271	5584700.675
White-naped Crane	-6228490.263	5906928.102
White-naped Crane	-6174085.088	5936290.996
White-naped Crane	-6178374.228	5932637.798
White-naped Crane	-5946733.952	6239639.11
White-naped Crane	-6174083.975	5936287.732
White-naped Crane	-6228493.602	5906931.355
White-naped Crane	-5330422.497	5584702.243
White-naped Crane	-6178372.002	5932650.849
White-naped Crane	-6174080.635	5936271.412
White-naped Crane	-6228486.923	5906926.476
White-naped Crane	-5330464.799	5584711.65
White-naped Crane	-5946733.952	6239635.728
White-naped Crane	-6178370.888	5932647.586
White-naped Crane	-5946741.745	6239652.641
White-naped Crane	-6174089.541	5936277.94
White-naped Crane	-6228488.036	5906976.895
White-naped Crane	-5332250.363	5580943.328
White-naped Crane	-6178367.549	5932634.536
White-naped Crane	-5944871.577	6244887.123
White-naped Crane	-6172199.336	5937595.035
White-naped Crane	-5333325.709	5584743.007
White-naped Crane	-6225674.993	5905329.476
White-naped Crane	-6178259.569	5932815.609
White-naped Crane	-6171286.516	5936465.618
White-naped Crane	-5945012.953	6244816.045
White-naped Crane	-5332844.809	5584601.901
White-naped Crane	-6228215.304	5906931.355
White-naped Crane	-6178180.532	5933079.885
White-naped Crane	-6171230.856	5936441.138
White-naped Crane	-6228106.21	5906931.355
White-naped Crane	-5330542.722	5584056.312
White-naped Crane	-6178363.096	5932645.955
White-naped Crane	-5330148.651	5584797.882
White-naped Crane	-6178360.87	5932634.536
White-naped Crane	-6227714.366	5907637.252
White-naped Crane	-6171327.704	5936584.755
White-naped Crane	-5929421.545	6253475.103
White-naped Crane	-6173879.147	5935427.729
White-naped Crane	-6227596.367	5908136.619
White-naped Crane	-5332181.345	5584738.303
White-naped Crane	-6178496.679	5932734.044
White-naped Crane	-5929145.473	6253603.854
White-naped Crane	-5331838.481	5585368.604
White-naped Crane	-6178366.436	5932649.217
White-naped Crane	-6173708.828	5935951.554
White-naped Crane	-5332018.819	5586320.411
White-naped Crane	-6172331.806	5936286.1
White-naped Crane	-6178368.662	5932647.586
White-naped Crane	-6229517.742	5908287.899
White-naped Crane	-6173665.414	5935951.554
White-naped Crane	-5331934.216	5586583.861
White-naped Crane	-6178364.209	5932644.324
White-naped Crane	-6229250.575	5908224.459
White-naped Crane	-5329779.07	5585878.208

White-naped Crane	-6011292.578	6322261.139
White-naped Crane	-6228363.359	5914824.741
White-naped Crane	-6178340.832	5932991.792
White-naped Crane	-6173698.809	5935931.971
White-naped Crane	-6173679.885	5935886.278
White-naped Crane	-6228490.263	5907009.424
White-naped Crane	-6178056.967	5933216.92
White-naped Crane	-6065726.696	6315593.667
White-naped Crane	-6173660.961	5935945.026
White-naped Crane	-5329663.298	5585719.835
White-naped Crane	-6228509.187	5907001.292
White-naped Crane	-6172532.181	5937692.969
White-naped Crane	-5329718.958	5585754.332
White-naped Crane	-6178292.965	5932704.681
White-naped Crane	-6065629.848	6315620.974
White-naped Crane	-6228526.998	5907014.303
White-naped Crane	-5329759.033	5585893.888
White-naped Crane	-6228460.206	5906853.287
White-naped Crane	-6065642.093	6315550.998
White-naped Crane	-6178361.983	5932644.324
White-naped Crane	-6171273.158	5936302.42
White-naped Crane	-6065594.225	6315607.321
White-naped Crane	-6172085.79	5938011.26
White-naped Crane	-6178394.265	5932696.524
White-naped Crane	-6228297.68	5907059.844
White-naped Crane	-5329663.298	5585713.563
White-naped Crane	-6173829.053	5935455.47
White-naped Crane	-5329678.883	5585737.084
White-naped Crane	-6066423.556	6311385.92
White-naped Crane	-6226161.459	5904929.459
White-naped Crane	-6178064.76	5932962.428
White-naped Crane	-6174100.673	5934726.083
White-naped Crane	-5329669.977	5585722.972
White-naped Crane	-6227289.125	5904705.068
White-naped Crane	-6066558.252	6311408.096
White-naped Crane	-6178365.322	5932647.586
White-naped Crane	-5329678.883	5585773.149
White-naped Crane	-6227291.352	5904698.564
White-naped Crane	-6178363.096	5932641.061
White-naped Crane	-6174304.387	5936356.275
White-naped Crane	-6066558.252	6311391.037
White-naped Crane	-6066544.894	6311375.684
White-naped Crane	-6174301.048	5936362.803
White-naped Crane	-5329672.204	5585773.149
White-naped Crane	-6227290.239	5904692.06
White-naped Crane	-6178367.549	5932647.586
White-naped Crane	-6066552.686	6311411.508
White-naped Crane	-6227286.899	5904696.938
White-naped Crane	-5329672.204	5585774.717
White-naped Crane	-6178366.436	5932649.217
White-naped Crane	-5329672.204	5585780.989
White-naped Crane	-6066550.46	6311401.273
White-naped Crane	-6227286.899	5904701.816
White-naped Crane	-6178359.756	5932642.692
White-naped Crane	-6174341.123	5936367.699
White-naped Crane	-6066558.252	6311384.214
White-naped Crane	-6174299.935	5936361.171
White-naped Crane	-5329678.883	5585771.581

White-naped Crane	-6227289.125	5904693.686
White-naped Crane	-6178353.077	5932652.48
White-naped Crane	-6174303.274	5936359.539
White-naped Crane	-6227280.22	5904672.548
White-naped Crane	-5329674.43	5585780.989
White-naped Crane	-6178363.096	5932652.48
White-naped Crane	-6227286.899	5904669.296
White-naped Crane	-5329676.657	5585780.989
White-naped Crane	-6174299.935	5936361.171
White-naped Crane	-6066546.007	6311401.273
White-naped Crane	-5329763.486	5585857.823
White-naped Crane	-6227264.635	5904732.71
White-naped Crane	-6178141.57	5932693.262
White-naped Crane	-6171681.7	5937601.564
White-naped Crane	-5331504.523	5586417.636
White-naped Crane	-6225599.296	5904914.825
White-naped Crane	-6177997.968	5933076.622
White-naped Crane	-5329767.939	5585931.522
White-naped Crane	-6065600.905	6315033.876
White-naped Crane	-6178102.608	5933119.038
White-naped Crane	-6171697.285	5937619.519
White-naped Crane	-6228156.304	5906937.861
White-naped Crane	-6173816.808	5935576.224
White-naped Crane	-6178113.74	5932946.115
White-naped Crane	-6065718.903	6313499.765
White-naped Crane	-6178364.209	5932641.061
White-naped Crane	-6228180.795	5907015.93
White-naped Crane	-6173958.184	5935535.429
White-naped Crane	-6173962.637	5935652.921
White-naped Crane	-6065685.507	6313359.849
White-naped Crane	-6228195.266	5906960.631
White-naped Crane	-6178360.87	5932641.061
White-naped Crane	-5330188.726	5586224.755
White-naped Crane	-6173952.618	5935636.602
White-naped Crane	-6228119.569	5907072.855
White-naped Crane	-5329736.769	5585595.962
White-naped Crane	-6177974.591	5932975.479
White-naped Crane	-6173951.505	5935633.339
White-naped Crane	-5329792.429	5585864.095
White-naped Crane	-6173943.712	5935665.976
White-naped Crane	-6228942.22	5915446.626
White-naped Crane	-6178269.588	5932866.18
White-naped Crane	-6173932.58	5935742.673
White-naped Crane	-6228078.381	5914614.744
White-naped Crane	-5330206.537	5586141.645
White-naped Crane	-6178359.756	5932642.692
White-naped Crane	-6077649.013	6355087.292
White-naped Crane	-6228117.342	5914665.208
White-naped Crane	-6178477.755	5932799.296
White-naped Crane	-5329725.637	5585995.813
White-naped Crane	-6173639.81	5936007.039
White-naped Crane	-6178354.19	5932649.217
White-naped Crane	-5329792.429	5586111.851
White-naped Crane	-6228401.207	5914570.791
White-naped Crane	-6173643.15	5935977.664
White-naped Crane	-6173516.245	5936044.573
White-naped Crane	-5329678.883	5585799.805
White-naped Crane	-6178364.209	5932645.955

White-naped Crane	-6069950.157	6328633.097
White-naped Crane	-6173535.17	5936073.947
White-naped Crane	-6069862.215	6328349.339
White-naped Crane	-6229241.669	5906760.583
White-naped Crane	-5330233.254	5586240.436
White-naped Crane	-6178467.736	5932854.761
White-naped Crane	-6069843.29	6328409.167
White-naped Crane	-6178360.87	5932645.955
White-naped Crane	-6171834.208	5937691.337
White-naped Crane	-5329767.939	5586044.423
White-naped Crane	-6229143.708	5906820.759
White-naped Crane	-6226370.74	5905075.805
White-naped Crane	-6069848.856	6328393.783
White-naped Crane	-6178361.983	5932642.692
White-naped Crane	-5330222.122	5586234.164
White-naped Crane	-6069552.747	6328521.986
White-naped Crane	-6173686.564	5935936.867
White-naped Crane	-6178593.527	5933246.285
White-naped Crane	-5330400.233	5585497.178
White-naped Crane	-6226006.725	5905259.553
White-naped Crane	-6069783.178	6323257.023
White-naped Crane	-6174140.748	5935768.783
White-naped Crane	-5330395.78	5585506.586
White-naped Crane	-6178367.549	5932644.324
White-naped Crane	-6227281.333	5904677.426
White-naped Crane	-5330400.233	5585492.474
White-naped Crane	-6227279.107	5904672.548
White-naped Crane	-6178365.322	5932628.011
White-naped Crane	-6069787.631	6323258.732
White-naped Crane	-6174112.918	5935703.508
White-naped Crane	-6069778.725	6323253.607
White-naped Crane	-5330393.554	5585500.314
White-naped Crane	-6227275.767	5904666.044
White-naped Crane	-6178367.549	5932637.798
White-naped Crane	-6174141.861	5935750.832
White-naped Crane	-5330398.007	5585495.61
White-naped Crane	-6174140.748	5935757.36
White-naped Crane	-6069774.272	6323257.023
White-naped Crane	-6178363.096	5932641.061
White-naped Crane	-6227272.428	5904669.296
White-naped Crane	-6069768.706	6323274.106
White-naped Crane	-6174137.408	5935763.887
White-naped Crane	-6227267.975	5904672.548
White-naped Crane	-5330393.554	5585503.45
White-naped Crane	-6178382.02	5932647.586
White-naped Crane	-6174140.748	5935760.623
White-naped Crane	-5330411.365	5585519.13
White-naped Crane	-6227274.654	5904669.296
White-naped Crane	-6178384.247	5932645.955
White-naped Crane	-6174142.974	5935744.305
White-naped Crane	-5330418.044	5585512.858
White-naped Crane	-6227272.428	5904670.922
White-naped Crane	-6178378.681	5932659.005
White-naped Crane	-6174140.748	5935745.937
White-naped Crane	-5330415.818	5585525.402
White-naped Crane	-6227293.578	5904672.548
White-naped Crane	-6069782.065	6323257.023
White-naped Crane	-6178390.926	5932634.536

White-naped Crane	-6069765.367	6323267.273
White-naped Crane	-6174148.54	5935762.255
White-naped Crane	-5331379.845	5586014.63
White-naped Crane	-6227286.899	5904825.393
White-naped Crane	-6178290.738	5932461.623
White-naped Crane	-6178062.533	5932525.241
White-naped Crane	-6227282.446	5904680.678
White-naped Crane	-6171690.606	5937684.808
White-naped Crane	-5330208.764	5586231.028
White-naped Crane	-6225660.521	5905360.373
White-naped Crane	-6178288.512	5932727.519
White-naped Crane	-6069183.166	6324203.48
White-naped Crane	-5329765.712	5585940.93
White-naped Crane	-6178326.361	5932875.968
White-naped Crane	-6225210.791	5905760.41
White-naped Crane	-6172611.218	5937160.878
White-naped Crane	-6172549.992	5937098.857
White-naped Crane	-5330233.254	5586191.825
White-naped Crane	-6226152.553	5904846.531
White-naped Crane	-6178367.549	5933037.47
White-naped Crane	-6227195.617	5904892.06
White-naped Crane	-6178006.874	5933287.07
White-naped Crane	-5329469.602	5581408.796
White-naped Crane	-6172505.464	5937040.101
White-naped Crane	-6174301.048	5934931.675
White-naped Crane	-6178091.476	5932978.742
White-naped Crane	-5329469.602	5581753.602
White-naped Crane	-6229222.745	5906736.187
White-naped Crane	-6174155.219	5934977.363
White-naped Crane	-6178171.627	5932977.11
White-naped Crane	-6229189.349	5906728.055
White-naped Crane	-6174146.314	5934894.146
White-naped Crane	-6229202.707	5906856.54
White-naped Crane	-6178183.872	5933030.945
White-naped Crane	-5329641.034	5581603.14
White-naped Crane	-5331477.806	5584779.068
White-naped Crane	-6174138.521	5934961.046
White-naped Crane	-5855310.594	6304849.871
White-naped Crane	-6178183.872	5932848.235
White-naped Crane	-6178183.872	5932900.438
White-naped Crane	-5331437.731	5584775.932
White-naped Crane	-6174226.464	5936341.587
White-naped Crane	-6174206.426	5936354.643
White-naped Crane	-5331549.05	5584852.757
White-naped Crane	-6226919.545	5916298.126
White-naped Crane	-6178358.643	5932644.324
White-naped Crane	-5331582.446	5584896.658
White-naped Crane	-6173498.434	5936163.704
White-naped Crane	-5856959.236	6302409.334
White-naped Crane	-6227065.373	5916325.805
White-naped Crane	-6178349.738	5932631.273
White-naped Crane	-5858332.918	6302792.751
White-naped Crane	-6178502.245	5932817.24
White-naped Crane	-6229296.216	5906741.066
White-naped Crane	-5331600.257	5585139.684
White-naped Crane	-6178373.115	5933153.297
White-naped Crane	-6228341.095	5906986.654
White-naped Crane	-6172183.751	5937169.038

White-naped Crane	-5329959.408	5585617.914
White-naped Crane	-6174524.8	5935145.432
White-naped Crane	-5860033.88	6301645.96
White-naped Crane	-6178011.326	5933008.106
White-naped Crane	-5859413.831	6301657.887
White-naped Crane	-5330141.972	5585661.819
White-naped Crane	-6178364.209	5932647.586
White-naped Crane	-6178366.436	5932634.536
White-naped Crane	-5859418.283	6301634.033
White-naped Crane	-5330146.425	5585668.091
White-naped Crane	-6229197.142	5906788.231
White-naped Crane	-5859397.133	6301635.737
White-naped Crane	-5330146.425	5585666.523
White-naped Crane	-6178361.983	5932645.955
White-naped Crane	-6229128.123	5906721.549
White-naped Crane	-6174535.932	5935189.489
White-naped Crane	-5859383.774	6301628.922
White-naped Crane	-5330141.972	5585666.523
White-naped Crane	-6229108.086	5906692.274
White-naped Crane	-6178374.228	5932639.43
White-naped Crane	-6174527.026	5935199.28
White-naped Crane	-6174609.403	5935191.121
White-naped Crane	-5330141.972	5585674.363
White-naped Crane	-5859383.774	6301620.402
White-naped Crane	-6229079.143	5906721.549
White-naped Crane	-6178359.756	5932644.324
White-naped Crane	-5859381.548	6301632.329
White-naped Crane	-5330144.198	5585672.795
White-naped Crane	-6229094.728	5906698.78
White-naped Crane	-6174613.856	5935184.594
White-naped Crane	-6178355.304	5932645.955
White-naped Crane	-6174610.516	5935182.962
White-naped Crane	-6229088.048	5906693.901
White-naped Crane	-5330141.972	5585672.795
White-naped Crane	-6178354.19	5932642.692
White-naped Crane	-5859448.34	6301632.329
White-naped Crane	-6174610.516	5935179.699
White-naped Crane	-6229083.596	5906697.153
White-naped Crane	-5330146.425	5585668.091
White-naped Crane	-6178364.209	5932647.586
White-naped Crane	-6229043.521	5906674.384
White-naped Crane	-5330431.403	5586260.822
White-naped Crane	-6178172.74	5932499.141
White-naped Crane	-6172246.09	5937438.344
White-naped Crane	-6171783.001	5937888.839
White-naped Crane	-6226034.555	5905205.892
White-naped Crane	-5329761.259	5585911.137
White-naped Crane	-5861207.188	6298370.042
White-naped Crane	-6178131.551	5932691.631
White-naped Crane	-6228210.851	5906924.85
White-naped Crane	-6178359.756	5932644.324
White-naped Crane	-5331333.091	5586077.353
White-naped Crane	-5864685.922	6279337.946
White-naped Crane	-5329663.298	5585473.658
White-naped Crane	-6228207.511	5906919.97
White-naped Crane	-6172361.862	5937883.942
White-naped Crane	-6178358.643	5932637.798
White-naped Crane	-6228208.624	5906926.476

White-naped Crane	-5329636.582	5585312.157
White-naped Crane	-6178429.888	5932761.776
White-naped Crane	-6172005.64	5937924.749
White-naped Crane	-6228524.772	5907100.505
White-naped Crane	-6173637.584	5935786.733
White-naped Crane	-6178472.189	5932805.821
White-naped Crane	-5867811.773	6259335.291
White-naped Crane	-6178437.68	5932618.223
White-naped Crane	-6173758.922	5935556.643
White-naped Crane	-6173833.506	5935439.152
White-naped Crane	-5871539.863	6253088.861
White-naped Crane	-6178358.643	5932644.324
White-naped Crane	-6228037.192	5907126.528
White-naped Crane	-5871595.522	6252519.695
White-naped Crane	-6174366.726	5934951.256
White-naped Crane	-6178365.322	5932642.692
White-naped Crane	-6226136.969	5904810.759
White-naped Crane	-6172532.181	5937606.461
White-naped Crane	-6178286.286	5932458.36
White-naped Crane	-6172396.371	5937123.339
White-naped Crane	-5402115.589	5763276.802
White-naped Crane	-6228201.945	5906937.861
White-naped Crane	-6178360.87	5932659.005
White-naped Crane	-5402133.4	5763332.795
White-naped Crane	-6228211.964	5906929.729
White-naped Crane	-5860048.352	6301606.771
White-naped Crane	-6174160.785	5935050.791
White-naped Crane	-6178148.249	5932479.566
White-naped Crane	-5859927.013	6301599.956
White-naped Crane	-5402271.436	5763423.985
White-naped Crane	-6228514.753	5907014.303
White-naped Crane	-6174254.294	5934983.89
White-naped Crane	-5402512.999	5763334.395
White-naped Crane	-6172145.902	5937606.461
White-naped Crane	-5859760.034	6301783.976
White-naped Crane	-6228551.488	5907077.735
White-naped Crane	-6178084.797	5933032.576
White-naped Crane	-5859439.434	6301579.509
White-naped Crane	-6172332.919	5936289.364
White-naped Crane	-5402582.017	5763289.6
White-naped Crane	-6226153.667	5905139.222
White-naped Crane	-6178365.322	5932634.536
White-naped Crane	-5859736.657	6301858.948
White-naped Crane	-6174496.97	5935714.931
White-naped Crane	-6178359.756	5932641.061
White-naped Crane	-5402990.56	5761835.497
White-naped Crane	-6226085.762	5905194.509
White-naped Crane	-6174604.95	5934683.659
White-naped Crane	-5402995.013	5761833.897
White-naped Crane	-6228215.304	5906934.608
White-naped Crane	-5859141.098	6301729.451
White-naped Crane	-5402997.239	5761829.099
White-naped Crane	-5859178.946	6301700.485
White-naped Crane	-6178361.983	5932641.061
White-naped Crane	-6228216.417	5906915.091
White-naped Crane	-6174613.856	5934721.188
White-naped Crane	-5859172.267	6301686.854
White-naped Crane	-6174607.176	5934685.291

White-naped Crane	-5403002.805	5761816.302
White-naped Crane	-6228215.304	5906911.838
White-naped Crane	-6178369.775	5932645.955
White-naped Crane	-6174601.61	5934695.081
White-naped Crane	-5402998.352	5761813.103
White-naped Crane	-5859176.72	6301693.669
White-naped Crane	-6228215.304	5906913.465
White-naped Crane	-6178364.209	5932645.955
White-naped Crane	-6174606.063	5934698.344
White-naped Crane	-5402997.239	5761819.502
White-naped Crane	-6228214.19	5906924.85
White-naped Crane	-6178363.096	5932649.217
White-naped Crane	-5859153.343	6301703.892
White-naped Crane	-6174606.063	5934695.081
White-naped Crane	-5403000.579	5761817.902
White-naped Crane	-6228214.19	5906937.861
White-naped Crane	-6178361.983	5932650.849
White-naped Crane	-5403003.918	5761814.703
White-naped Crane	-6228216.417	5906916.718
White-naped Crane	-5859138.871	6301715.82
White-naped Crane	-6174597.158	5934696.713
White-naped Crane	-6178358.643	5932626.38
White-naped Crane	-5859145.551	6301707.3
White-naped Crane	-6174313.293	5935075.267
White-naped Crane	-5403005.032	5761817.902
White-naped Crane	-6228237.567	5907041.953
White-naped Crane	-6178361.983	5932641.061
White-naped Crane	-5402359.378	5763080.028
White-naped Crane	-6228236.454	5907035.447
White-naped Crane	-5402450.66	5763121.622
White-naped Crane	-6228168.549	5907063.097
White-naped Crane	-6177796.48	5932556.235
White-naped Crane	-6168905.392	5940130.235
White-naped Crane	-6168927.656	5940042.07
White-naped Crane	-5402521.905	5762984.043
White-naped Crane	-6228020.494	5907129.781
White-naped Crane	-5859418.283	6301606.771
White-naped Crane	-6173773.393	5934629.815
White-naped Crane	-5402534.15	5762963.246
White-naped Crane	-6227987.099	5907103.758
White-naped Crane	-6168846.393	5939849.416
White-naped Crane	-5859423.849	6301615.29
White-naped Crane	-6171318.799	5936454.194
White-naped Crane	-6227989.325	5907103.758
White-naped Crane	-5859701.035	6301768.641
White-naped Crane	-6172557.785	5935693.717
White-naped Crane	-6227984.872	5907098.879
White-naped Crane	-6178365.322	5932641.061
White-naped Crane	-6174830.929	5934672.238
White-naped Crane	-6227989.325	5907105.384
White-naped Crane	-5857246.44	6303264.804
White-naped Crane	-6178368.662	5932645.955
White-naped Crane	-6228214.19	5906928.102
White-naped Crane	-6178363.096	5932639.43
White-naped Crane	-6178482.208	5932424.104
White-naped Crane	-6178336.379	5932494.247
White-naped Crane	-5879902.183	6262428.033
White-naped Crane	-6224844.549	5929519.366

White-naped Crane	-6228661.695	5907624.24
White-naped Crane	-5879905.522	6262397.507
White-naped Crane	-6224821.172	5929405.218
White-naped Crane	-6228646.11	5907596.588
White-naped Crane	-6178485.547	5932727.519
White-naped Crane	-6224816.72	5929408.48
White-naped Crane	-6228632.752	5907596.588
White-naped Crane	-6178367.549	5932642.692
White-naped Crane	-6224574.043	5929558.502
White-naped Crane	-6228216.417	5906916.718
White-naped Crane	-6228089.513	5907027.315
White-naped Crane	-5884106.72	6259235.271
White-naped Crane	-6178363.096	5932644.324
White-naped Crane	-6224886.851	5929501.428
White-naped Crane	-5884172.399	6259526.858
White-naped Crane	-6224935.831	5929463.923
White-naped Crane	-6178364.209	5932645.955
White-naped Crane	-6228024.947	5907142.793
White-naped Crane	-5517856.69	5888939.067
White-naped Crane	-5884122.305	6259282.738
White-naped Crane	-5517836.652	5888950.429
White-naped Crane	-6228232.002	5907072.855
White-naped Crane	-6224944.737	5929452.508
White-naped Crane	-6178172.74	5932517.085
White-naped Crane	-6224942.511	5929431.309
White-naped Crane	-5517836.652	5888921.214
White-naped Crane	-5884106.72	6259228.49
White-naped Crane	-6228238.681	5907030.568
White-naped Crane	-6178245.097	5932667.161
White-naped Crane	-5884101.154	6259223.404
White-naped Crane	-5517833.313	5888942.314
White-naped Crane	-6224936.945	5929432.94
White-naped Crane	-6228240.907	5907040.326
White-naped Crane	-6178368.662	5932632.905
White-naped Crane	-5884097.814	6259230.185
White-naped Crane	-6224934.718	5929439.462
White-naped Crane	-6228245.36	5907020.809
White-naped Crane	-6178359.756	5932637.798
White-naped Crane	-5523179.988	5887110.089
White-naped Crane	-5523194.459	5887124.694
White-naped Crane	-6224925.813	5929431.309
White-naped Crane	-6228242.02	5907040.326
White-naped Crane	-5884105.607	6259230.185
White-naped Crane	-6178363.096	5932639.43
White-naped Crane	-5884105.607	6259231.88
White-naped Crane	-6228242.02	5907040.326
White-naped Crane	-5523207.818	5887119.826
White-naped Crane	-6178369.775	5932645.955
White-naped Crane	-6224934.718	5929431.309
White-naped Crane	-6224946.963	5929436.201
White-naped Crane	-6228240.907	5907040.326
White-naped Crane	-5884106.72	6259233.575
White-naped Crane	-5523215.61	5887116.58
White-naped Crane	-6178383.134	5932637.798
White-naped Crane	-5884103.38	6259223.404
White-naped Crane	-6224942.511	5929432.94
White-naped Crane	-5523186.667	5887129.562
White-naped Crane	-6228242.02	5907038.7

White-naped Crane	-6178384.247	5932647.586
White-naped Crane	-5883854.025	6259194.585
White-naped Crane	-6224955.869	5929496.536
White-naped Crane	-5523187.78	5887090.617
White-naped Crane	-6223296.095	5929600.9
White-naped Crane	-5523099.838	5886994.878
White-naped Crane	-5883404.294	6259086.09
White-naped Crane	-6225934.367	5905064.422
White-naped Crane	-6178600.207	5932885.756
White-naped Crane	-5883608.009	6259508.21
White-naped Crane	-6230623.144	5933210.395
White-naped Crane	-6225685.012	5904843.279
White-naped Crane	-6178624.697	5933274.019
White-naped Crane	-5521978.851	5888161.664
White-naped Crane	-6230706.634	5932955.903
White-naped Crane	-6225739.558	5905088.814
White-naped Crane	-5517833.313	5888596.612
White-naped Crane	-6178355.304	5933424.109
White-naped Crane	-6230593.088	5933238.128
White-naped Crane	-6225650.503	5904974.989
White-naped Crane	-5513781.283	5889583.44
White-naped Crane	-6178039.156	5933305.015
White-naped Crane	-5877426.437	6253151.539
White-naped Crane	-6225129.527	5905301.832
White-naped Crane	-6230509.598	5935473.42
White-naped Crane	-6178197.23	5933433.897
White-naped Crane	-6178443.246	5933212.026
White-naped Crane	-5471781.553	5898229.489
White-naped Crane	-6225113.943	5905048.162
White-naped Crane	-6230489.561	5935543.588
White-naped Crane	-6230468.41	5935546.852
White-naped Crane	-5471812.722	5898203.493
White-naped Crane	-6225090.565	5905184.753
White-naped Crane	-6178363.096	5932639.43
White-naped Crane	-5471856.137	5898180.746
White-naped Crane	-6225165.15	5905150.605
White-naped Crane	-6225258.658	5905116.457
White-naped Crane	-5471998.626	5898414.717
White-naped Crane	-6178365.322	5932662.268
White-naped Crane	-6189122.125	5979092.745
White-naped Crane	-6189747.74	5979691.405
White-naped Crane	-5472038.701	5898434.215
White-naped Crane	-6178697.055	5932778.089
White-naped Crane	-6189706.552	5979722.569
White-naped Crane	-6225787.425	5905423.792
White-naped Crane	-5472038.701	5898429.341
White-naped Crane	-6178670.338	5932822.134
White-naped Crane	-5861186.037	6292567.518
White-naped Crane	-5860492.516	6301647.664
White-naped Crane	-5472006.418	5898382.221
White-naped Crane	-6225731.766	5905283.945
White-naped Crane	-6178487.774	5932825.397
White-naped Crane	-6189696.533	5979809.502
White-naped Crane	-5860028.314	6301513.059
White-naped Crane	-6189716.571	5979752.094
White-naped Crane	-5472049.833	5898354.599
White-naped Crane	-6225896.519	5905370.129
White-naped Crane	-6178117.08	5932921.645

White-naped Crane	-5472014.21	5898422.841
White-naped Crane	-6228001.57	5907121.649
White-naped Crane	-6189735.495	5979679.924
White-naped Crane	-6178360.87	5932637.798
White-naped Crane	-6185169.17	5975086.896
White-naped Crane	-5472060.964	5898443.964
White-naped Crane	-6228013.815	5907120.023
White-naped Crane	-6178357.53	5932642.692
White-naped Crane	-6185207.018	5975163.945
White-naped Crane	-6228528.111	5906919.97
White-naped Crane	-5472047.606	5898422.841
White-naped Crane	-6178360.87	5932642.692
White-naped Crane	-6185245.98	5975191.815
White-naped Crane	-5472057.625	5898437.465
White-naped Crane	-5855292.783	6304826.008
White-naped Crane	-6228520.319	5906911.838
White-naped Crane	-6178364.209	5932639.43
White-naped Crane	-6185234.848	5975186.896
White-naped Crane	-6228493.602	5906913.465
White-naped Crane	-5472052.059	5898447.214
White-naped Crane	-6178364.209	5932644.324
White-naped Crane	-5855291.67	6304839.644
White-naped Crane	-6185238.188	5975196.733
White-naped Crane	-6178364.209	5932644.324
White-naped Crane	-5472045.38	5898447.214
White-naped Crane	-6228498.055	5906923.223
White-naped Crane	-5472045.38	5898437.465
White-naped Crane	-5855297.236	6304839.644
White-naped Crane	-6228495.829	5906915.091
White-naped Crane	-6178359.756	5932657.374
White-naped Crane	-6185232.622	5975193.454
White-naped Crane	-5855295.009	6304829.417
White-naped Crane	-6185235.961	5975191.815
White-naped Crane	-5472048.719	5898440.714
White-naped Crane	-6228508.074	5906924.85
White-naped Crane	-6178354.19	5932644.324
White-naped Crane	-6185245.98	5975193.454
White-naped Crane	-6228502.508	5906921.597
White-naped Crane	-5855305.028	6304831.122
White-naped Crane	-5472049.833	5898440.714
White-naped Crane	-6178356.417	5932610.067
White-naped Crane	-5855336.198	6304844.758
White-naped Crane	-5472049.833	5898443.964
White-naped Crane	-6228499.168	5906916.718
White-naped Crane	-6185243.754	5975196.733
White-naped Crane	-6178375.341	5932626.38
White-naped Crane	-6228491.376	5907006.171
White-naped Crane	-5472063.191	5898435.84
White-naped Crane	-5855396.31	6304853.28
White-naped Crane	-6178510.038	5932872.705
White-naped Crane	-6188128.042	5977998.863
White-naped Crane	-5472050.946	5898484.585
White-naped Crane	-6228004.91	5907115.143
White-naped Crane	-6178591.301	5933425.74
White-naped Crane	-5854658.262	6307702.048
White-naped Crane	-6178540.094	5933440.423
White-naped Crane	-5471527.744	5898135.252
White-naped Crane	-6189982.624	5979791.459

White-naped Crane	-6228006.023	5907113.517
White-naped Crane	-5471584.517	5897782.684
White-naped Crane	-6228209.738	5906928.102
White-naped Crane	-6178079.231	5933645.985
White-naped Crane	-6189939.21	5979804.581
White-naped Crane	-6188097.986	5977993.943
White-naped Crane	-6228214.19	5906923.223
White-naped Crane	-6178147.136	5933645.985
White-naped Crane	-5858711.405	6304476.586
White-naped Crane	-5857862.037	6303499.988
White-naped Crane	-6188012.269	5978000.502
White-naped Crane	-5422803.203	5923347.802
White-naped Crane	-6228111.776	5907082.614
White-naped Crane	-6178270.701	5933828.711
White-naped Crane	-6189380.386	5971667.908
White-naped Crane	-6228213.077	5906924.85
White-naped Crane	-6190147.377	5971602.36
White-naped Crane	-6178661.432	5933324.592
White-naped Crane	-5382700.356	5958663.276
White-naped Crane	-6228216.417	5906931.355
White-naped Crane	-6190101.736	5971522.066
White-naped Crane	-5382435.416	5958643.641
White-naped Crane	-6178616.904	5932853.129
White-naped Crane	-6228142.946	5907076.108
White-naped Crane	-6189110.993	5979094.385
White-naped Crane	-5382351.926	5958445.662
White-naped Crane	-6178363.096	5932650.849
White-naped Crane	-6228210.851	5906929.729
White-naped Crane	-5382256.192	5957976.094
White-naped Crane	-6178356.417	5932650.849
White-naped Crane	-6189109.88	5979081.264
White-naped Crane	-6228214.19	5906926.476
White-naped Crane	-6186570.682	5978520.366
White-naped Crane	-5382160.457	5958105.346
White-naped Crane	-6228208.624	5906926.476
White-naped Crane	-6178360.87	5932647.586
White-naped Crane	-6178455.491	5932738.938
White-naped Crane	-6228207.511	5906919.97
White-naped Crane	-5382354.153	5958483.294
White-naped Crane	-5859618.658	6301669.815
White-naped Crane	-6189444.951	5979863.63
White-naped Crane	-5382412.039	5958326.223
White-naped Crane	-6178959.769	5933265.862
White-naped Crane	-6228213.077	5906908.585
White-naped Crane	-6189443.838	5979860.35
White-naped Crane	-5860211.991	6301879.395
White-naped Crane	-6178973.127	5933319.698
White-naped Crane	-5382400.907	5958336.04
White-naped Crane	-6228213.077	5906921.597
White-naped Crane	-5858895.082	6301918.586
White-naped Crane	-5382334.115	5958826.899
White-naped Crane	-6185889.407	5976409.948
White-naped Crane	-6228211.964	5906924.85
White-naped Crane	-6178722.658	5933164.716
White-naped Crane	-5382245.06	5958270.594
White-naped Crane	-5855354.009	6304693.056
White-naped Crane	-6185888.294	5976418.146
White-naped Crane	-6228226.436	5906942.74

White-naped Crane	-6178363.096	5932639.43
White-naped Crane	-5382262.871	5958252.596
White-naped Crane	-6185888.294	5976419.785
White-naped Crane	-5855335.084	6304640.216
White-naped Crane	-6178359.756	5932644.324
White-naped Crane	-6228225.322	5906924.85
White-naped Crane	-5855349.556	6304653.852
White-naped Crane	-6185884.954	5976434.542
White-naped Crane	-5382263.984	5958255.869
White-naped Crane	-6178360.87	5932632.905
White-naped Crane	-6228210.851	5906923.223
White-naped Crane	-6185880.501	5976434.542
White-naped Crane	-5382261.758	5958273.866
White-naped Crane	-6228210.851	5906921.597
White-naped Crane	-6178363.096	5932644.324
White-naped Crane	-6185890.52	5976441.1
White-naped Crane	-6228214.19	5906929.729
White-naped Crane	-5381901.082	5958123.343
White-naped Crane	-6178363.096	5932641.061
White-naped Crane	-5381940.044	5958126.615
White-naped Crane	-6228213.077	5906924.85
White-naped Crane	-5855346.216	6304660.67
White-naped Crane	-6185883.841	5976462.415
White-naped Crane	-6178364.209	5932637.798
White-naped Crane	-5381944.497	5958124.979
White-naped Crane	-6228211.964	5906924.85
White-naped Crane	-6185888.294	5976437.821
White-naped Crane	-6185909.444	5976457.496
White-naped Crane	-5855343.99	6304653.852
White-naped Crane	-5381951.176	5958146.248
White-naped Crane	-6228209.738	5906924.85
White-naped Crane	-6178351.964	5932650.849
White-naped Crane	-6178599.093	5933311.541
White-naped Crane	-5855202.614	6304740.782
White-naped Crane	-5382269.55	5957949.917
White-naped Crane	-6228205.285	5907015.93
White-naped Crane	-6178631.376	5933453.474
White-naped Crane	-6189944.776	5979778.337
White-naped Crane	-5854492.396	6305969.827
White-naped Crane	-6178410.963	5933667.194
White-naped Crane	-5381451.352	5958192.06
White-naped Crane	-6189961.474	5979771.776
White-naped Crane	-6228021.608	5907133.034
White-naped Crane	-6189777.797	5979686.484
White-naped Crane	-6228011.589	5907120.023
White-naped Crane	-6178474.416	5933570.938
White-naped Crane	-5858011.205	6303428.41
White-naped Crane	-6228002.683	5907129.781
White-naped Crane	-6179038.805	5933435.529
White-naped Crane	-6189782.249	5979678.283
White-naped Crane	-6188067.929	5977867.672
White-naped Crane	-6178880.732	5933412.689
White-naped Crane	-6228001.57	5907100.505
White-naped Crane	-5859156.683	6301707.3
White-naped Crane	-6228008.249	5907100.505
White-naped Crane	-6190053.869	5971612.192
White-naped Crane	-6178911.901	5933349.063
White-naped Crane	-6229149.274	5906703.659

White-naped Crane	-6178360.87	5932641.061
White-naped Crane	-6190027.152	5971502.402
White-naped Crane	-6229158.18	5906882.563
White-naped Crane	-6178366.436	5932639.43
White-naped Crane	-5869256.7	6292276.476
White-naped Crane	-6189989.304	5971433.578
White-naped Crane	-5869268.945	6292296.9
White-naped Crane	-6178158.268	5932567.654
White-naped Crane	-6189875.758	5971364.755
White-naped Crane	-6189873.531	5971254.968
White-naped Crane	-5168063.02	6031396.003
White-naped Crane	-6228486.923	5906954.125
White-naped Crane	-6178425.435	5932654.111
White-naped Crane	-5870919.813	6251391.641
White-naped Crane	-6190268.716	5971569.587
White-naped Crane	-5168194.377	6031384.452
White-naped Crane	-6178364.209	5932642.692
White-naped Crane	-6228417.905	5906924.85
White-naped Crane	-5167100.106	6031298.644
White-naped Crane	-5871033.359	6246902.949
White-naped Crane	-6228189.7	5907004.545
White-naped Crane	-6178359.756	5932636.167
White-naped Crane	-6190300.998	5971587.612
White-naped Crane	-5871085.679	6246821.697
White-naped Crane	-6189843.475	5979686.484
White-naped Crane	-6228111.776	5907056.591
White-naped Crane	-6178493.34	5932539.923
White-naped Crane	-5167243.708	6031300.294
White-naped Crane	-5870767.305	6247058.684
White-naped Crane	-6189726.59	5979694.686
White-naped Crane	-5166907.524	6031476.861
White-naped Crane	-6228211.964	5906928.102
White-naped Crane	-6189728.816	5979699.606
White-naped Crane	-5164783.548	6032117.153
White-naped Crane	-5869678.601	6246936.804
White-naped Crane	-6228164.097	5907093.999
White-naped Crane	-6178338.606	5933349.063
White-naped Crane	-5164865.924	6032136.956
White-naped Crane	-6185655.636	5976837.893
White-naped Crane	-6178363.096	5932645.955
White-naped Crane	-6229088.048	5906715.044
White-naped Crane	-5164865.924	6032128.705
White-naped Crane	-6185674.56	5976841.172
White-naped Crane	-5870713.872	6249764.191
White-naped Crane	-6178360.87	5932637.798
White-naped Crane	-6229060.219	5906664.626
White-naped Crane	-5870716.098	6249760.804
White-naped Crane	-5164863.698	6032136.956
White-naped Crane	-6178364.209	5932641.061
White-naped Crane	-6229061.332	5906671.131
White-naped Crane	-6185587.731	5976842.812
White-naped Crane	-5164727.888	6032032.988
White-naped Crane	-5870717.212	6249770.964
White-naped Crane	-6178365.322	5932639.43
White-naped Crane	-6229063.558	5906672.758
White-naped Crane	-6185510.921	5976688.683
White-naped Crane	-5870716.098	6249772.657
White-naped Crane	-6185963.991	5976613.26

White-naped Crane	-5164729.001	6032031.338
White-naped Crane	-6229061.332	5906676.011
White-naped Crane	-6178365.322	5932631.273
White-naped Crane	-5870690.495	6249760.804
White-naped Crane	-5164735.68	6032028.037
White-naped Crane	-6229064.671	5906674.384
White-naped Crane	-6178359.756	5932637.798
White-naped Crane	-6185975.123	5976608.341
White-naped Crane	-6185968.444	5976600.143
White-naped Crane	-5870716.098	6249767.577
White-naped Crane	-6229078.03	5906667.879
White-naped Crane	-5164753.491	6032024.736
White-naped Crane	-6178364.209	5932629.642
White-naped Crane	-5870716.098	6249770.964
White-naped Crane	-6185965.104	5976605.062
White-naped Crane	-5164736.793	6032024.736
White-naped Crane	-6178363.096	5932639.43
White-naped Crane	-6229060.219	5906671.131
White-naped Crane	-5870887.531	6249708.311
White-naped Crane	-6185889.407	5976557.512
White-naped Crane	-6229218.292	5906728.055
White-naped Crane	-6178255.116	5932944.483
White-naped Crane	-5871161.376	6248912.488
White-naped Crane	-6188038.986	5977874.231
White-naped Crane	-6178142.683	5933350.695
White-naped Crane	-5167783.608	6031476.861
White-naped Crane	-6177817.631	5933433.897
White-naped Crane	-6225848.651	5905043.284
White-naped Crane	-6189938.097	5979666.802
White-naped Crane	-6227950.363	5907120.023
White-naped Crane	-6177684.047	5933078.254
White-naped Crane	-5871070.094	6248882.011
White-naped Crane	-6189162.2	5972379.129
White-naped Crane	-6189036.409	5972229.997
White-naped Crane	-6228000.457	5907110.264
White-naped Crane	-5871231.508	6250477.114
White-naped Crane	-6178361.983	5932645.955
White-naped Crane	-5151690.149	6032174.913
White-naped Crane	-5870995.51	6250367.039
White-naped Crane	-6228206.398	5907002.918
White-naped Crane	-6178361.983	5932649.217
White-naped Crane	-6188874.996	5971949.767
White-naped Crane	-5870943.19	6251511.891
White-naped Crane	-6228147.399	5907077.735
White-naped Crane	-6189257.935	5972198.86
White-naped Crane	-6178297.418	5932479.566
White-naped Crane	-5870958.775	6251488.18
White-naped Crane	-6189538.46	5972395.517
White-naped Crane	-6178498.906	5932445.31
White-naped Crane	-5151622.244	6029376.448
White-naped Crane	-6228018.268	5907126.528
White-naped Crane	-6228134.04	5907111.89
White-naped Crane	-5155259.052	6027688.902
White-naped Crane	-5870907.568	6251503.423
White-naped Crane	-6178358.643	5932530.135
White-naped Crane	-6189312.481	5972157.89
White-naped Crane	-6189203.388	5972298.827
White-naped Crane	-5167809.211	6031628.678

White-naped Crane	-5870928.719	6251500.036
White-naped Crane	-6178366.436	5932634.536
White-naped Crane	-6228136.267	5907108.637
White-naped Crane	-5870938.737	6251488.18
White-naped Crane	-5167769.136	6030902.62
White-naped Crane	-6189250.142	5972388.962
White-naped Crane	-6228268.737	5906985.027
White-naped Crane	-6178359.756	5932647.586
White-naped Crane	-5870958.775	6251472.937
White-naped Crane	-5167252.614	6028780.903
White-naped Crane	-6228173.002	5907095.626
White-naped Crane	-6178370.888	5932659.005
White-naped Crane	-6187383.314	5975229.52
White-naped Crane	-6187250.844	5975239.356
White-naped Crane	-5871035.585	6248951.431
White-naped Crane	-6228012.702	5907155.805
White-naped Crane	-5168242.244	6033678.477
White-naped Crane	-6178927.486	5933353.958
White-naped Crane	-5167506.422	6031674.884
White-naped Crane	-6228148.512	5907058.217
White-naped Crane	-6179035.466	5933114.144
White-naped Crane	-5870702.74	6249745.564
White-naped Crane	-6187246.391	5975616.42
White-naped Crane	-6187282.014	5975518.054
White-naped Crane	-5167697.892	6031361.35
White-naped Crane	-6179055.503	5933502.417
White-naped Crane	-6228210.851	5907074.482
White-naped Crane	-5870236.311	6250202.775
White-naped Crane	-6187265.316	5975375.425
White-naped Crane	-5167977.304	6031382.802
White-naped Crane	-6228176.342	5906991.533
White-naped Crane	-6178237.305	5933559.518
White-naped Crane	-5167975.077	6031480.161
White-naped Crane	-6178363.096	5932644.324
White-naped Crane	-6186184.403	5975796.761
White-naped Crane	-5870739.476	6249754.031
White-naped Crane	-6228223.096	5907063.097
White-naped Crane	-5167973.964	6031490.063
White-naped Crane	-5870732.796	6249770.964
White-naped Crane	-6228139.606	5907097.252
White-naped Crane	-6178359.756	5932642.692
White-naped Crane	-6186174.385	5975767.25
White-naped Crane	-5870730.57	6249765.884
White-naped Crane	-6186140.989	5975706.59
White-naped Crane	-5167956.153	6031485.112
White-naped Crane	-6228130.701	5907084.241
White-naped Crane	-6178367.549	5932644.324
White-naped Crane	-5167963.945	6031486.762
White-naped Crane	-5870729.457	6249769.271
White-naped Crane	-6228128.474	5907089.12
White-naped Crane	-6186149.894	5975703.311
White-naped Crane	-6178355.304	5932636.167
White-naped Crane	-5870736.136	6249776.044
White-naped Crane	-6186154.347	5975704.95
White-naped Crane	-5167962.832	6031485.112
White-naped Crane	-6228139.606	5907089.12
White-naped Crane	-6178365.322	5932634.536
White-naped Crane	-6186130.97	5975785.284

White-naped Crane	-5167956.153	6031523.066
White-naped Crane	-5870749.494	6249781.124
White-naped Crane	-6228127.361	5907093.999
White-naped Crane	-6178373.115	5932650.849
White-naped Crane	-6186155.46	5975709.869
White-naped Crane	-5870736.136	6249776.044
White-naped Crane	-6228130.701	5907089.12
White-naped Crane	-5167960.606	6031486.762
White-naped Crane	-6178390.926	5932642.692
White-naped Crane	-6186151.008	5975708.229
White-naped Crane	-5870704.967	6249750.644
White-naped Crane	-5167946.134	6031514.815
White-naped Crane	-6228116.229	5907098.879
White-naped Crane	-6178364.209	5932637.798
White-naped Crane	-5870856.361	6249726.938
White-naped Crane	-6187171.807	5975460.674
White-naped Crane	-5167109.012	6031100.63
White-naped Crane	-6178441.02	5932996.686
White-naped Crane	-6189778.91	5979684.844
White-naped Crane	-5167093.427	6031917.469
White-naped Crane	-5870305.33	6250243.417
White-naped Crane	-6225996.706	5905332.729
White-naped Crane	-6178434.341	5933629.67
White-naped Crane	-6226081.309	5905342.485
White-naped Crane	-5167504.196	6031953.775
White-naped Crane	-6178376.454	5933645.985
White-naped Crane	-5870885.304	6251733.767
White-naped Crane	-6189098.748	5979082.904
White-naped Crane	-5167524.233	6031950.474
White-naped Crane	-6225849.764	5905324.598
White-naped Crane	-6178009.1	5933507.312
White-naped Crane	-6189804.513	5979737.331
White-naped Crane	-6225755.143	5905155.483
White-naped Crane	-5871196.999	6252619.635
White-naped Crane	-6226721.396	5904804.255
White-naped Crane	-5149958.018	5954094.548
White-naped Crane	-6178163.834	5932645.955
White-naped Crane	-6189796.721	5971721.985
White-naped Crane	-5904204.341	6221371.148
White-naped Crane	-6228240.907	5906955.752
White-naped Crane	-6189683.175	5971613.831
White-naped Crane	-6178357.53	5932637.798
White-naped Crane	-5150932.063	5953438.8
White-naped Crane	-5151191.438	5953662.828
White-naped Crane	-6228312.152	5906941.114
White-naped Crane	-6178373.115	5932639.43
White-naped Crane	-6228158.531	5907025.688
White-naped Crane	-6190139.585	5971594.167
White-naped Crane	-6189734.382	5971251.69
White-naped Crane	-6228177.455	5906993.16
White-naped Crane	-5161928.203	5946101.431
White-naped Crane	-6177945.648	5932740.569
White-naped Crane	-5161933.769	5946065.487
White-naped Crane	-6228208.624	5907040.326
White-naped Crane	-6189743.288	5979724.21
White-naped Crane	-6177046.186	5931510.661
White-naped Crane	-6189737.722	5979714.368
White-naped Crane	-5162029.503	5946219.067

White-naped Crane	-6177031.715	5931491.089
White-naped Crane	-6227954.816	5907110.264
White-naped Crane	-6189734.382	5979702.887
White-naped Crane	-6228179.681	5906991.533
White-naped Crane	-6055230.381	6294259.504
White-naped Crane	-5161913.731	5946060.585
White-naped Crane	-6178366.436	5932634.536
White-naped Crane	-6055346.153	6294205.028
White-naped Crane	-6178366.436	5932636.167
White-naped Crane	-5161920.41	5946045.881
White-naped Crane	-6228472.452	5907090.746
White-naped Crane	-6189745.514	5979684.844
White-naped Crane	-5161879.222	5946060.585
White-naped Crane	-6189738.835	5979679.924
White-naped Crane	-6228178.568	5906988.28
White-naped Crane	-6178369.775	5932655.742
White-naped Crane	-6054588.067	6293874.776
White-naped Crane	-5161899.26	5946073.656
White-naped Crane	-6228180.795	5906983.401
White-naped Crane	-6189133.257	5979091.105
White-naped Crane	-6178367.549	5932649.217
White-naped Crane	-6055149.118	6294433.147
White-naped Crane	-6186069.744	5976903.48
White-naped Crane	-5161899.26	5946065.487
White-naped Crane	-6178363.096	5932641.061
White-naped Crane	-6228199.719	5907009.424
White-naped Crane	-6055146.891	6294412.718
White-naped Crane	-6186067.518	5976898.561
White-naped Crane	-5161904.826	5946065.487
White-naped Crane	-6178363.096	5932639.43
White-naped Crane	-6228190.813	5906999.665
White-naped Crane	-6186051.933	5976655.89
White-naped Crane	-5161904.826	5946081.825
White-naped Crane	-6055158.023	6294414.421
White-naped Crane	-6228134.04	5907092.373
White-naped Crane	-6055149.118	6294419.528
White-naped Crane	-6185912.784	5976542.756
White-naped Crane	-5161904.826	5946075.29
White-naped Crane	-6228129.588	5907097.252
White-naped Crane	-6178357.53	5932659.005
White-naped Crane	-6185911.671	5976542.756
White-naped Crane	-5161902.599	5946080.191
White-naped Crane	-6228132.927	5907098.879
White-naped Crane	-6055159.136	6294404.206
White-naped Crane	-6178361.983	5932645.955
White-naped Crane	-6185915.01	5976509.963
White-naped Crane	-5161902.599	5946080.191
White-naped Crane	-6228134.04	5907097.252
White-naped Crane	-6055151.344	6294404.206
White-naped Crane	-6178367.549	5932644.324
White-naped Crane	-6055160.25	6294402.504
White-naped Crane	-6186010.745	5975796.761
White-naped Crane	-5161914.844	5946076.924
White-naped Crane	-6228137.38	5907051.712
White-naped Crane	-6178384.247	5932632.905
White-naped Crane	-6055148.004	6294402.504
White-naped Crane	-6186005.179	5975795.121
White-naped Crane	-6228132.927	5907095.626

White-naped Crane	-6178364.209	5932639.43
White-naped Crane	-6186008.519	5975668.882
White-naped Crane	-6228127.361	5907084.241
White-naped Crane	-6178310.776	5932479.566
White-naped Crane	-5162030.617	5946364.481
White-naped Crane	-6228215.304	5907056.591
White-naped Crane	-6054515.71	6294342.92
White-naped Crane	-6188080.174	5977982.464
White-naped Crane	-6177728.575	5932611.698
White-naped Crane	-6226032.328	5905205.892
White-naped Crane	-5162570.516	5949399.122
White-naped Crane	-6055476.397	6294594.878
White-naped Crane	-6188069.042	5977982.464
White-naped Crane	-6055390.681	6294691.917
White-naped Crane	-6189944.776	5979781.618
White-naped Crane	-5162343.424	5949538.052
White-naped Crane	-6226175.931	5905205.892
White-naped Crane	-5162019.485	5949779.958
White-naped Crane	-6227145.523	5904692.06
White-naped Crane	-6177583.86	5933314.804
White-naped Crane	-6055219.249	6294215.242
White-naped Crane	-6189775.57	5979671.722
White-naped Crane	-6055200.325	6294254.396
White-naped Crane	-6227038.657	5904661.166
White-naped Crane	-6178346.398	5933526.889
White-naped Crane	-6189102.087	5979099.305
White-naped Crane	-6226908.413	5904659.54
White-naped Crane	-6054686.029	6293983.724
White-naped Crane	-6178423.209	5933371.903
White-naped Crane	-6054868.593	6293871.372
White-naped Crane	-6189121.012	5979097.665
White-naped Crane	-6227097.656	5904563.607
White-naped Crane	-6177995.742	5933608.461
White-naped Crane	-6054856.347	6293881.586
White-naped Crane	-6178035.817	5933641.091
White-naped Crane	-6189995.983	5979712.728
White-naped Crane	-5277427.74	5876199.425
White-naped Crane	-6189227.878	5979962.046
White-naped Crane	-6226498.757	5904693.686
White-naped Crane	-6177960.119	5933699.824
White-naped Crane	-6054740.575	6293789.662
White-naped Crane	-6189280.198	5979924.32
White-naped Crane	-5276993.594	5875919.056
White-naped Crane	-6178118.193	5933711.244
White-naped Crane	-6054870.819	6293888.395
White-naped Crane	-6189232.331	5979970.247
White-naped Crane	-5276502.675	5875990.363
White-naped Crane	-6228136.267	5907103.758
White-naped Crane	-6054857.461	6293878.181
White-naped Crane	-6189475.008	5979794.74
White-naped Crane	-5276473.732	5875975.777
White-naped Crane	-6177788.687	5933393.112
White-naped Crane	-6189578.535	5979789.819
White-naped Crane	-5274142.702	5877757.019
White-naped Crane	-6054878.611	6293830.517
White-naped Crane	-5274127.117	5877758.64
White-naped Crane	-6226045.687	5905204.266
White-naped Crane	-5273124.129	5877132.976

White-naped Crane	-6055712.394	6293243.25
White-naped Crane	-6178383.134	5931575.904
White-naped Crane	-6228191.926	5907012.677
White-naped Crane	-6185651.183	5976942.833
White-naped Crane	-6055185.853	6294237.373
White-naped Crane	-6178808.374	5930279.298
White-naped Crane	-5273100.752	5877108.663
White-naped Crane	-6228229.775	5907037.074
White-naped Crane	-6185622.24	5976926.436
White-naped Crane	-5272940.452	5876998.448
White-naped Crane	-6178806.148	5930276.036
White-naped Crane	-6185628.919	5976911.678
White-naped Crane	-6055190.306	6294249.289
White-naped Crane	-6228129.588	5907087.493
White-naped Crane	-6185626.693	5976918.237
White-naped Crane	-5272939.338	5877008.172
White-naped Crane	-6055196.985	6294233.968
White-naped Crane	-6228130.701	5907090.746
White-naped Crane	-5272938.225	5877011.414
White-naped Crane	-6055199.211	6294245.885
White-naped Crane	-6185618.9	5976916.597
White-naped Crane	-6228136.267	5907093.999
White-naped Crane	-6178791.676	5930285.821
White-naped Crane	-6055199.211	6294266.313
White-naped Crane	-6185613.334	5976926.436
White-naped Crane	-5272850.283	5877056.797
White-naped Crane	-6228136.267	5907095.626
White-naped Crane	-6178806.148	5930284.191
White-naped Crane	-6185575.486	5976852.65
White-naped Crane	-5272845.83	5877056.797
White-naped Crane	-6228138.493	5907092.373
White-naped Crane	-6055201.438	6294216.944
White-naped Crane	-6178799.468	5930277.667
White-naped Crane	-6185594.41	5976839.532
White-naped Crane	-6055202.551	6294244.182
White-naped Crane	-5272864.754	5877058.418
White-naped Crane	-6228138.493	5907087.493
White-naped Crane	-6178815.053	5930271.144
White-naped Crane	-6055240.4	6294240.777
White-naped Crane	-6228135.154	5907087.493
White-naped Crane	-5272853.622	5877056.797
White-naped Crane	-6185574.373	5976847.731
White-naped Crane	-6178802.808	5930284.191
White-naped Crane	-6228034.966	5907129.781
White-naped Crane	-5274364.228	5877672.73
White-naped Crane	-6054601.426	6294007.556
White-naped Crane	-6178929.712	5929408.48
White-naped Crane	-6187180.713	5978120.216
White-naped Crane	-6055909.43	6295985.887
White-naped Crane	-5274595.773	5877734.326
White-naped Crane	-6225840.859	5905290.449
White-naped Crane	-6050661.829	6296239.597
White-naped Crane	-5274217.286	5877429.593
White-naped Crane	-6225928.801	5905251.423
White-naped Crane	-6178056.967	5932632.905
White-naped Crane	-6189940.323	5979788.179
White-naped Crane	-6049390.56	6297785.861
White-naped Crane	-6226158.119	5904895.313

White-naped Crane	-6188047.892	5977872.591
White-naped Crane	-6188043.439	5977864.392
White-naped Crane	-5274433.246	5877089.213
White-naped Crane	-6226316.193	5905041.657
White-naped Crane	-6178367.549	5932637.798
White-naped Crane	-6049125.62	6298949.154
White-naped Crane	-6048792.775	6300761.693
White-naped Crane	-5274394.284	5877077.868
White-naped Crane	-6227164.448	5904653.036
White-naped Crane	-6177891.101	5932089.708
White-naped Crane	-6187897.61	5977900.469
White-naped Crane	-6188063.476	5977877.511
White-naped Crane	-6046573.064	6304814.076
White-naped Crane	-6178366.436	5932644.324
White-naped Crane	-5273373.485	5877186.463
White-naped Crane	-6227154.429	5904653.036
White-naped Crane	-6227170.014	5904649.784
White-naped Crane	-6178364.209	5932654.111
White-naped Crane	-6046706.647	6304877.144
White-naped Crane	-6190366.677	5971792.449
White-naped Crane	-6227180.032	5904877.426
White-naped Crane	-6047357.866	6304667.488
White-naped Crane	-5274693.734	5877852.656
White-naped Crane	-6177862.158	5932019.568
White-naped Crane	-6190272.055	5971744.926
White-naped Crane	-5491254.671	5877462.011
White-naped Crane	-5273944.553	5877791.059
White-naped Crane	-6050078.515	6302112.837
White-naped Crane	-6190296.545	5971797.365
White-naped Crane	-6178358.643	5932645.955
White-naped Crane	-6227177.806	5904890.434
White-naped Crane	-6227377.068	5904913.199
White-naped Crane	-6178149.363	5932882.493
White-naped Crane	-5273706.33	5877068.143
White-naped Crane	-6050080.741	6302085.573
White-naped Crane	-5492200.887	5877970.988
White-naped Crane	-5273584.992	5876348.527
White-naped Crane	-6227736.63	5904987.997
White-naped Crane	-6189108.766	5979114.066
White-naped Crane	-6177904.46	5933163.085
White-naped Crane	-6050035.1	6302133.284
White-naped Crane	-6225433.43	5905015.64
White-naped Crane	-5272536.362	5875914.194
White-naped Crane	-6050009.497	6302180.996
White-naped Crane	-6189162.2	5979097.665
White-naped Crane	-6177679.594	5933091.305
White-naped Crane	-6048825.057	6304057.3
White-naped Crane	-6225349.94	5905257.927
White-naped Crane	-6189941.436	5979748.813
White-naped Crane	-5274330.832	5877836.446
White-naped Crane	-5492167.491	5877938.568
White-naped Crane	-6048761.605	6303980.604
White-naped Crane	-6190049.416	5979722.569
White-naped Crane	-5492163.038	5877927.221
White-naped Crane	-5272924.867	5876604.6
White-naped Crane	-6225225.262	5905322.972
White-naped Crane	-6177744.16	5933125.563
White-naped Crane	-6185555.448	5976851.01

White-naped Crane	-5492194.208	5877962.883
White-naped Crane	-6048840.642	6304091.388
White-naped Crane	-6178369.775	5932647.586
White-naped Crane	-6226567.775	5904682.304
White-naped Crane	-5492189.755	5877956.399
White-naped Crane	-6185553.222	5976854.289
White-naped Crane	-5272872.547	5876622.428
White-naped Crane	-6178368.662	5932645.955
White-naped Crane	-6185543.203	5976849.37
White-naped Crane	-5492189.755	5877954.778
White-naped Crane	-6178359.756	5932632.905
White-naped Crane	-5272667.719	5876606.221
White-naped Crane	-6048559.004	6304074.344
White-naped Crane	-6227154.429	5904487.186
White-naped Crane	-6048567.909	6304094.796
White-naped Crane	-6185542.09	5976849.37
White-naped Crane	-5272526.343	5876487.908
White-naped Crane	-5492191.981	5877953.157
White-naped Crane	-6227121.033	5904480.682
White-naped Crane	-6178361.983	5932642.692
White-naped Crane	-6185546.543	5976844.451
White-naped Crane	-5272519.664	5876491.149
White-naped Crane	-5492189.755	5877956.399
White-naped Crane	-6048554.551	6304087.979
White-naped Crane	-6178363.096	5932649.217
White-naped Crane	-6227277.993	5904708.32
White-naped Crane	-6048565.683	6304122.066
White-naped Crane	-6185543.203	5976859.208
White-naped Crane	-5492183.076	5877954.778
White-naped Crane	-5272525.23	5876491.149
White-naped Crane	-6227361.483	5904892.06
White-naped Crane	-6178365.322	5932642.692
White-naped Crane	-6048533.4	6304106.727
White-naped Crane	-6185547.656	5976846.091
White-naped Crane	-5492180.849	5877946.673
White-naped Crane	-5272536.362	5876496.011
White-naped Crane	-6227363.709	5904892.06
White-naped Crane	-6178367.549	5932641.061
White-naped Crane	-6185543.203	5976852.65
White-naped Crane	-5492183.076	5877951.536
White-naped Crane	-6227362.596	5904882.304
White-naped Crane	-5272528.57	5876494.391
White-naped Crane	-6048533.4	6304087.979
White-naped Crane	-6178367.549	5932649.217
White-naped Crane	-6048526.721	6304091.388
White-naped Crane	-6185546.543	5976851.01
White-naped Crane	-5272530.796	5876491.149
White-naped Crane	-5492180.849	5877946.673
White-naped Crane	-6227363.709	5904883.93
White-naped Crane	-6178356.417	5932655.742
White-naped Crane	-5492169.717	5877911.011
White-naped Crane	-5277785.076	5876633.773
White-naped Crane	-6227171.127	5904879.052
White-naped Crane	-6185599.976	5976860.848
White-naped Crane	-6178355.304	5932637.798
White-naped Crane	-5490758.186	5876779.641
White-naped Crane	-6225531.391	5905256.301
White-naped Crane	-5277937.584	5875957.951

White-naped Crane	-6177453.616	5931911.912
White-naped Crane	-6048186.083	6304832.826
White-naped Crane	-5490802.714	5876842.851
White-naped Crane	-6225276.469	5905279.067
White-naped Crane	-6177829.876	5931969.002
White-naped Crane	-5277932.018	5875867.197
White-naped Crane	-6048351.95	6303799.944
White-naped Crane	-5491492.895	5877395.555
White-naped Crane	-6225067.188	5905410.783
White-naped Crane	-5277893.056	5875841.267
White-naped Crane	-6178368.662	5932657.374
White-naped Crane	-6185595.523	5976862.488
White-naped Crane	-5491490.668	5877392.313
White-naped Crane	-5279253.38	5875708.379
White-naped Crane	-6227065.373	5904500.194
White-naped Crane	-6055465.265	6294749.801
White-naped Crane	-6178364.209	5932639.43
White-naped Crane	-6055474.171	6294700.429
White-naped Crane	-5491490.668	5877390.692
White-naped Crane	-5281069.001	5874047.456
White-naped Crane	-6227171.127	5904501.82
White-naped Crane	-5281035.605	5874049.077
White-naped Crane	-6054947.629	6294097.78
White-naped Crane	-5491499.574	5877387.45
White-naped Crane	-5490386.379	5876862.301
White-naped Crane	-6227079.845	5905093.692
White-naped Crane	-6054968.78	6294097.78
White-naped Crane	-6177891.101	5932071.765
White-naped Crane	-5281102.397	5873844.927
White-naped Crane	-6185587.731	5976855.929
White-naped Crane	-5490326.267	5876713.19
White-naped Crane	-5281070.114	5873885.432
White-naped Crane	-6227943.684	5907107.011
White-naped Crane	-6177488.125	5933247.917
White-naped Crane	-5490341.851	5876675.913
White-naped Crane	-6001856.025	6303605.653
White-naped Crane	-5281022.247	5874023.153
White-naped Crane	-6177690.726	5933112.512
White-naped Crane	-6185594.41	5976849.37
White-naped Crane	-6227165.561	5904518.079
White-naped Crane	-5491909.23	5876812.056
White-naped Crane	-6227050.902	5904470.926
White-naped Crane	-5281018.907	5874039.355
White-naped Crane	-6185587.731	5976867.407
White-naped Crane	-6226535.492	5904911.573
White-naped Crane	-5281011.115	5874055.558
White-naped Crane	-5491884.739	5876813.677
White-naped Crane	-6177331.164	5933185.924
White-naped Crane	-5942123.099	6301438.089
White-naped Crane	-5492895.52	5881030.337
White-naped Crane	-6178363.096	5932624.748
White-naped Crane	-6185613.334	5976906.759
White-naped Crane	-5493111.48	5880954.124
White-naped Crane	-6227324.748	5904662.792
White-naped Crane	-6178359.756	5932641.061
White-naped Crane	-5278044.45	5875726.206
White-naped Crane	-5277053.707	5876870.405
White-naped Crane	-5894763.335	6265686.485

White-naped Crane	-5493164.914	5880929.801
White-naped Crane	-6178359.756	5932641.061
White-naped Crane	-6227259.069	5904812.385
White-naped Crane	-5277044.801	5876980.619
White-naped Crane	-5492876.596	5880952.503
White-naped Crane	-6178330.813	5932632.905
White-naped Crane	-6226199.308	5905109.953
White-naped Crane	-5870708.306	6249606.712
White-naped Crane	-5277060.386	5876962.79
White-naped Crane	-6228140.72	5907095.626
White-naped Crane	-6178369.775	5932645.955
White-naped Crane	-5870798.475	6248876.932
White-naped Crane	-5277052.594	5876974.135
White-naped Crane	-6178363.096	5932637.798
White-naped Crane	-6228134.04	5907087.493
White-naped Crane	-6185614.448	5976887.083
White-naped Crane	-5870802.928	6248854.921
White-naped Crane	-6185589.957	5976865.767
White-naped Crane	-5277048.141	5876967.652
White-naped Crane	-6228144.059	5907097.252
White-naped Crane	-6178364.209	5932641.061
White-naped Crane	-6185586.618	5976877.245
White-naped Crane	-5277041.462	5876987.102
White-naped Crane	-5870787.343	6248887.091
White-naped Crane	-6178361.983	5932644.324
White-naped Crane	-6228010.476	5907100.505
White-naped Crane	-5277042.575	5876985.481
White-naped Crane	-6178361.983	5932642.692
White-naped Crane	-6228129.588	5907095.626
White-naped Crane	-6185587.731	5976864.127
White-naped Crane	-6228140.72	5907107.011
White-naped Crane	-6178363.096	5932642.692
White-naped Crane	-5277044.801	5876991.964
White-naped Crane	-5870746.155	6249000.533
White-naped Crane	-5870740.589	6249002.226
White-naped Crane	-6178364.209	5932641.061
White-naped Crane	-5277044.801	5876982.239
White-naped Crane	-6228141.833	5907095.626
White-naped Crane	-5492911.105	5880876.29
White-naped Crane	-5870746.155	6248988.681
White-naped Crane	-6178365.322	5932639.43
White-naped Crane	-5277088.216	5877021.139
White-naped Crane	-5492909.992	5880873.047
White-naped Crane	-6228138.493	5907095.626
White-naped Crane	-5277906.414	5875991.984
White-naped Crane	-6228166.323	5907053.338
White-naped Crane	-6177802.046	5932104.388
White-naped Crane	-5496915.267	5876620.807
White-naped Crane	-5870857.474	6249732.018
White-naped Crane	-5278013.281	5876421.459
White-naped Crane	-6177289.976	5933412.689
White-naped Crane	-6177377.919	5933432.266
White-naped Crane	-5870643.741	6246176.79
White-naped Crane	-6225679.446	5904841.653
White-naped Crane	-5280228.539	5880736.839
White-naped Crane	-5492908.879	5880869.804
White-naped Crane	-5870635.948	6246159.864
White-naped Crane	-5276931.255	5877102.18

White-naped Crane	-6177327.825	5933208.763
White-naped Crane	-5493292.931	5880936.287
White-naped Crane	-5492950.067	5880887.641
White-naped Crane	-5870908.681	6244374.358
White-naped Crane	-6227961.495	5907110.264
White-naped Crane	-5871119.075	6244323.591
White-naped Crane	-5281882.746	5872631.467
White-naped Crane	-6178366.436	5932639.43
White-naped Crane	-6229197.142	5906837.023
White-naped Crane	-5492993.482	5879820.736
White-naped Crane	-5493076.971	5879890.453
White-naped Crane	-5871160.263	6244325.283
White-naped Crane	-5870709.419	6245980.45
White-naped Crane	-5280998.87	5874068.52
White-naped Crane	-5870642.628	6246154.786
White-naped Crane	-5278120.148	5876082.739
White-naped Crane	-6227450.539	5905067.675
White-naped Crane	-6178285.172	5932712.837
White-naped Crane	-5278104.563	5875520.396
White-naped Crane	-6177866.611	5933561.149
White-naped Crane	-6227273.541	5904659.54
White-naped Crane	-5871087.906	6248089.653
White-naped Crane	-5871792.558	6248959.897
White-naped Crane	-6226000.046	5905095.318
White-naped Crane	-6177422.446	5933340.906
White-naped Crane	-5278057.809	5875476.641
White-naped Crane	-5384868.86	5891976.317
White-naped Crane	-5871913.896	6249146.148
White-naped Crane	-6226042.347	5905082.309
White-naped Crane	-5385334.176	5891896.76
White-naped Crane	-5278095.657	5875513.914
White-naped Crane	-6178363.096	5932641.061
White-naped Crane	-6226045.687	5905070.927
White-naped Crane	-5385341.968	5891848.052
White-naped Crane	-6178365.322	5932645.955
White-naped Crane	-5870861.927	6249743.871
White-naped Crane	-5283690.575	5877914.253
White-naped Crane	-6176744.511	5931763.48
White-naped Crane	-5871115.735	6248837.99
White-naped Crane	-5288973.798	5878643.725
White-naped Crane	-6226193.742	5905292.076
White-naped Crane	-5378815.306	5890580.121
White-naped Crane	-5378808.627	5890570.381
White-naped Crane	-5280169.539	5874350.45
White-naped Crane	-5871131.32	6248824.444
White-naped Crane	-6227303.597	5904947.346
White-naped Crane	-6177147.487	5932264.245
White-naped Crane	-5871062.302	6249107.204
White-naped Crane	-5360400.836	5900008.815
White-naped Crane	-5280178.445	5874138.191
White-naped Crane	-6228007.136	5907118.396
White-naped Crane	-6177460.295	5932331.124
White-naped Crane	-6178365.322	5932641.061
White-naped Crane	-5277034.783	5876881.75
White-naped Crane	-6228166.323	5907084.241
White-naped Crane	-5360436.458	5900064.069
White-naped Crane	-5360425.326	5900052.694
White-naped Crane	-5277031.443	5876876.888

White-naped Crane	-5871770.294	6248882.011
White-naped Crane	-6178365.322	5932644.324
White-naped Crane	-6228149.625	5907102.132
White-naped Crane	-5360420.874	5900051.068
White-naped Crane	-5871776.973	6248910.795
White-naped Crane	-5277034.783	5876872.025
White-naped Crane	-6228131.814	5907095.626
White-naped Crane	-6178364.209	5932639.43
White-naped Crane	-5277030.33	5876876.888
White-naped Crane	-6228136.267	5907100.505
White-naped Crane	-6178373.115	5932650.849
White-naped Crane	-5871839.312	6248966.67
White-naped Crane	-6228139.606	5907118.396
White-naped Crane	-5277045.915	5876899.579
White-naped Crane	-6178364.209	5932595.385
White-naped Crane	-5871867.142	6248986.988
White-naped Crane	-5360425.326	5900060.819
White-naped Crane	-5277057.046	5876889.854
White-naped Crane	-6228141.833	5907090.746
White-naped Crane	-5871859.35	6249039.476
White-naped Crane	-6178363.096	5932641.061
White-naped Crane	-5871843.765	6249017.465
White-naped Crane	-5277060.386	5876883.371
White-naped Crane	-6228134.04	5907089.12
White-naped Crane	-6178374.228	5932628.011
White-naped Crane	-5277052.594	5876886.612
White-naped Crane	-5360452.043	5900062.444
White-naped Crane	-5871853.784	6249036.09
White-naped Crane	-6228129.588	5907095.626
White-naped Crane	-6178314.115	5932616.592
White-naped Crane	-5871742.464	6248837.99
White-naped Crane	-6228134.04	5907084.241
White-naped Crane	-5277670.417	5876082.739
White-naped Crane	-6177532.653	5933101.093
White-naped Crane	-5360376.346	5899989.314
White-naped Crane	-5870847.455	6247923.742
White-naped Crane	-6225357.732	5905347.364
White-naped Crane	-5276954.633	5876920.649
White-naped Crane	-6177301.108	5933234.866
White-naped Crane	-5362325.55	5900642.636
White-naped Crane	-5362449.115	5900733.65
White-naped Crane	-5870895.323	6246794.614
White-naped Crane	-6177323.372	5933190.818
White-naped Crane	-5871123.528	6248807.513
White-naped Crane	-5351557.616	5904122.978
White-naped Crane	-6227934.778	5907108.637
White-naped Crane	-6177342.296	5933298.49
White-naped Crane	-5276954.633	5877082.73
White-naped Crane	-6227944.797	5907103.758
White-naped Crane	-5351457.428	5904059.568
White-naped Crane	-5277919.773	5875977.398
White-naped Crane	-5871339.488	6249842.084
White-naped Crane	-6177229.864	5933309.91
White-naped Crane	-6228008.249	5907116.77
White-naped Crane	-6178363.096	5932652.48
White-naped Crane	-5871032.246	6251496.648
White-naped Crane	-5281023.36	5874047.456
White-naped Crane	-5303872.799	5873423.68

White-naped Crane	-5870900.889	6251669.406
White-naped Crane	-6229186.01	5906721.549
White-naped Crane	-6178365.322	5932647.586
White-naped Crane	-6177618.369	5933166.348
White-naped Crane	-5281012.228	5874040.975
White-naped Crane	-5281020.02	5874045.836
White-naped Crane	-6226733.641	5915137.306
White-naped Crane	-6177927.837	5933694.929
White-naped Crane	-5267972.263	5876171.874
White-naped Crane	-5267958.904	5876152.426
White-naped Crane	-5867226.232	6258857.239
White-naped Crane	-5280999.983	5874050.697
White-naped Crane	-6231100.705	5915720.139
White-naped Crane	-6230947.084	5916221.601
White-naped Crane	-5278085.639	5875484.744
White-naped Crane	-5867416.589	6258972.511
White-naped Crane	-5276930.142	5877050.314
White-naped Crane	-5274894.109	5877851.035
White-naped Crane	-6231818.716	5917771.762
White-naped Crane	-6178367.549	5932647.586
White-naped Crane	-6177062.884	5932037.51
White-naped Crane	-6231883.281	5917851.557
White-naped Crane	-5276945.727	5877102.18
White-naped Crane	-5278065.601	5875868.817
White-naped Crane	-5274867.392	5877859.14
White-naped Crane	-6177460.295	5932355.593
White-naped Crane	-5859717.733	6301737.97
White-naped Crane	-6228518.093	5907024.062
White-naped Crane	-5274903.014	5877857.519
White-naped Crane	-5277944.263	5875839.646
White-naped Crane	-6228501.395	5906996.413
White-naped Crane	-6177167.525	5932272.401
White-naped Crane	-5855372.933	6304723.737
White-naped Crane	-5275123.427	5877823.479
White-naped Crane	-5277011.405	5876936.857
White-naped Crane	-6228352.227	5907001.292
White-naped Crane	-6177140.808	5931854.822
White-naped Crane	-5855340.65	6304677.715
White-naped Crane	-5277075.971	5876991.964
White-naped Crane	-6228187.474	5907063.097
White-naped Crane	-6178356.417	5932644.324
White-naped Crane	-5277043.688	5876988.723
White-naped Crane	-5855367.367	6304643.625
White-naped Crane	-6178361.983	5932645.955
White-naped Crane	-6228147.399	5907102.132
White-naped Crane	-5277042.575	5876990.343
White-naped Crane	-5855355.122	6304638.512
White-naped Crane	-6178369.775	5932642.692
White-naped Crane	-6228138.493	5907095.626
White-naped Crane	-5277043.688	5876987.102
White-naped Crane	-6228132.927	5907100.505
White-naped Crane	-5855368.48	6304648.739
White-naped Crane	-6178351.964	5932641.061
White-naped Crane	-5855380.725	6304631.694
White-naped Crane	-5277050.367	5876990.343
White-naped Crane	-6228132.927	5907095.626
White-naped Crane	-6178367.549	5932644.324
White-naped Crane	-6228135.154	5907085.867

White-naped Crane	-5277053.707	5876991.964
White-naped Crane	-5855359.575	6304636.807
White-naped Crane	-6178370.888	5932639.43
White-naped Crane	-5855356.235	6304645.33
White-naped Crane	-6228141.833	5907095.626
White-naped Crane	-5277050.367	5876987.102
White-naped Crane	-6178375.341	5932637.798
White-naped Crane	-5277032.556	5876873.646
White-naped Crane	-6228140.72	5907095.626
White-naped Crane	-5855337.311	6304665.784
White-naped Crane	-6178370.888	5932636.167
White-naped Crane	-5855341.764	6304664.079
White-naped Crane	-5276955.746	5877076.247
White-naped Crane	-6226028.989	5905217.275
White-naped Crane	-6177188.675	5932208.784
White-naped Crane	-5857774.094	6303609.062
White-naped Crane	-6225371.091	5905035.153
White-naped Crane	-6177001.659	5932202.259
White-naped Crane	-5278473.03	5878929.046
White-naped Crane	-5275444.027	5877719.738
White-naped Crane	-5280958.795	5877888.317
White-naped Crane	-6225993.367	5905070.927
White-naped Crane	-6176952.678	5931787.946
White-naped Crane	-5275448.48	5877684.077
White-naped Crane	-5858263.9	6302545.658
White-naped Crane	-6226003.385	5905079.057
White-naped Crane	-6177538.219	5933125.563
White-naped Crane	-5858313.994	6302480.904
White-naped Crane	-5306814.973	5861667.805
White-naped Crane	-5275403.952	5877619.24
White-naped Crane	-6177391.277	5933411.057
White-naped Crane	-6178376.454	5932649.217
White-naped Crane	-5858748.14	6303447.157
White-naped Crane	-5306953.009	5866767.455
White-naped Crane	-6228516.979	5906999.665
White-naped Crane	-5275410.631	5877669.488
White-naped Crane	-5307060.989	5866684.892
White-naped Crane	-6228501.395	5906994.786
White-naped Crane	-6178367.549	5932644.324
White-naped Crane	-5305911.058	5865368.84
White-naped Crane	-5275412.858	5877659.763
White-naped Crane	-6228485.81	5906991.533
White-naped Crane	-6178374.228	5932636.167
White-naped Crane	-5275397.273	5877687.319
White-naped Crane	-6227947.024	5907105.384
White-naped Crane	-6177148.6	5932208.784
White-naped Crane	-5823080.262	6348984.617
White-naped Crane	-5275415.084	5877656.521
White-naped Crane	-5277943.15	5875980.639
White-naped Crane	-5823407.541	6349164.547
White-naped Crane	-6177745.273	5932934.695
White-naped Crane	-6228238.681	5906949.246
White-naped Crane	-5816948.784	6357910.44
White-naped Crane	-5277946.489	5875982.26
White-naped Crane	-6229149.274	5906702.033
White-naped Crane	-5275421.763	5877674.351
White-naped Crane	-5275455.159	5877658.142
White-naped Crane	-5277940.923	5875868.817

White-naped Crane	-6229282.858	5906679.263
White-naped Crane	-6177441.371	5933167.979
White-naped Crane	-5275386.141	5877671.109
White-naped Crane	-5817655.663	6358462.833
White-naped Crane	-5277940.923	5875842.888
White-naped Crane	-6178364.209	5932628.011
White-naped Crane	-6228880.994	5906552.407
White-naped Crane	-5817348.421	6358310.149
White-naped Crane	-5275419.537	5877674.351
White-naped Crane	-5277958.734	5875860.714
White-naped Crane	-6228512.527	5906978.522
White-naped Crane	-6178369.775	5932639.43
White-naped Crane	-5817156.952	6357913.871
White-naped Crane	-6177462.521	5933225.077
White-naped Crane	-6228509.187	5907001.292
White-naped Crane	-5277819.585	5875859.094
White-naped Crane	-5275390.594	5877679.214
White-naped Crane	-5816848.597	6358042.53
White-naped Crane	-6226750.339	5904705.068
White-naped Crane	-5279090.854	5876210.77
White-naped Crane	-6177709.651	5933070.097
White-naped Crane	-6228138.493	5907090.746
White-naped Crane	-5280404.424	5876985.481
White-naped Crane	-5816869.748	6358193.494
White-naped Crane	-6178364.209	5932637.798
White-naped Crane	-5816840.805	6358162.615
White-naped Crane	-5280447.838	5877009.793
White-naped Crane	-6228131.814	5907082.614
White-naped Crane	-6178369.775	5932660.636
White-naped Crane	-5816838.578	6358160.899
White-naped Crane	-5280441.159	5877009.793
White-naped Crane	-6228138.493	5907092.373
White-naped Crane	-6178363.096	5932639.43
White-naped Crane	-5816827.446	6358147.175
White-naped Crane	-6228137.38	5907102.132
White-naped Crane	-5280450.065	5877009.793
White-naped Crane	-6178360.87	5932628.011
White-naped Crane	-5816833.012	6358155.753
White-naped Crane	-6228146.286	5907108.637
White-naped Crane	-5280448.951	5877006.552
White-naped Crane	-6178358.643	5932644.324
White-naped Crane	-5280448.951	5877008.172
White-naped Crane	-6178376.454	5932639.43
White-naped Crane	-5816830.786	6358150.606
White-naped Crane	-5816834.125	6358150.606
White-naped Crane	-5280446.725	5877013.035
White-naped Crane	-6228135.154	5907093.999
White-naped Crane	-6178367.549	5932632.905
White-naped Crane	-5816840.805	6358145.46
White-naped Crane	-5280441.159	5877003.31
White-naped Crane	-6228043.872	5907133.034
White-naped Crane	-6178363.096	5932641.061
White-naped Crane	-5272700.002	5876901.2
White-naped Crane	-5275383.915	5877650.037
White-naped Crane	-5816903.143	6358159.184
White-naped Crane	-6228146.286	5907090.746
White-naped Crane	-5816010.361	6361167
White-naped Crane	-5275426.216	5877658.142

White-naped Crane	-6224887.964	5905077.431
White-naped Crane	-5277014.745	5877098.938
White-naped Crane	-6177140.808	5933464.894
White-naped Crane	-5275417.31	5877612.756
White-naped Crane	-5277939.81	5875983.88
White-naped Crane	-6223328.378	5902432.212
White-naped Crane	-6177240.996	5933251.179
White-naped Crane	-5273454.748	5877507.397
White-naped Crane	-5279583.999	5879679.68
White-naped Crane	-6223706.864	5902170.498
White-naped Crane	-6177330.051	5933195.712
White-naped Crane	-5270830.947	5880012.055
White-naped Crane	-5814779.168	6376043.676
White-naped Crane	-6227565.198	5908338.326
White-naped Crane	-5280005.9	5879760.746
White-naped Crane	-6177328.938	5933097.83
White-naped Crane	-5813885.272	6375680.932
White-naped Crane	-5281310.564	5881886.565
White-naped Crane	-5270853.211	5880021.784
White-naped Crane	-6230053.188	5913703.184
White-naped Crane	-5820347.368	6376427.068
White-naped Crane	-5284069.061	5881743.855
White-naped Crane	-5270846.532	5880005.57
White-naped Crane	-6229725.909	5913669.002
White-naped Crane	-6178367.549	5932647.586
White-naped Crane	-5814144.646	6376437.383
White-naped Crane	-5281573.278	5873943.761
White-naped Crane	-5270797.551	5879995.842
White-naped Crane	-6178364.209	5932637.798
White-naped Crane	-5814445.209	6376547.419
White-naped Crane	-5281204.811	5874133.331
White-naped Crane	-6177678.481	5933120.669
White-naped Crane	-5275317.123	5877494.429
White-naped Crane	-5275194.671	5877465.253
White-naped Crane	-5281463.072	5873710.449
White-naped Crane	-6177726.349	5933039.101
White-naped Crane	-6226546.624	5917081.32
White-naped Crane	-5815248.936	6374842.039
White-naped Crane	-5815125.371	6374775
White-naped Crane	-5275221.388	5877460.39
White-naped Crane	-6230848.01	5922642.249
White-naped Crane	-6177414.654	5933130.457
White-naped Crane	-5281524.298	5873804.421
White-naped Crane	-5275214.709	5877450.665
White-naped Crane	-6177303.334	5933283.807
White-naped Crane	-5281613.353	5873963.203
White-naped Crane	-5815097.541	6374986.432
White-naped Crane	-5815474.914	6375407.594
White-naped Crane	-5275232.52	5877445.802
White-naped Crane	-5277946.489	5875961.192
White-naped Crane	-6230718.879	5924322.309
White-naped Crane	-6177076.243	5933406.163
White-naped Crane	-5275199.124	5877473.358
White-naped Crane	-6178366.436	5932639.43
White-naped Crane	-5277889.716	5875996.845
White-naped Crane	-6230723.332	5924301.123
White-naped Crane	-5275408.405	5877659.763
White-naped Crane	-6229848.361	5924183.785

White-naped Crane	-5812123.085	6376444.261
White-naped Crane	-6178358.643	5932650.849
White-naped Crane	-5814320.531	6366794.358
White-naped Crane	-5277766.152	5875948.227
White-naped Crane	-6178364.209	5932649.217
White-naped Crane	-6229964.133	5924252.232
White-naped Crane	-5275297.085	5877593.305
White-naped Crane	-5277453.344	5876789.366
White-naped Crane	-5817231.536	6358030.522
White-naped Crane	-5275326.028	5877536.573
White-naped Crane	-6229906.247	5923540.087
White-naped Crane	-5275328.255	5877512.259
White-naped Crane	-6178369.775	5932636.167
White-naped Crane	-5277493.419	5876810.436
White-naped Crane	-6229966.359	5923575.938
White-naped Crane	-5816839.691	6358047.677
White-naped Crane	-5816840.805	6358037.384
White-naped Crane	-5277495.645	5876808.815
White-naped Crane	-6178366.436	5932639.43
White-naped Crane	-6230064.32	5923525.421
White-naped Crane	-5277492.306	5876825.023
White-naped Crane	-6230108.848	5923378.763
White-naped Crane	-6178364.209	5932642.692
White-naped Crane	-5816833.012	6358106.003
White-naped Crane	-5277491.192	5876815.298
White-naped Crane	-6230117.754	5923370.615
White-naped Crane	-6178358.643	5932647.586
White-naped Crane	-5277491.192	5876820.16
White-naped Crane	-6230124.433	5923351.061
White-naped Crane	-5816860.842	6358064.832
White-naped Crane	-6178363.096	5932645.955
White-naped Crane	-5277493.419	5876818.54
White-naped Crane	-5816847.484	6358054.539
White-naped Crane	-6230106.622	5923351.061
White-naped Crane	-6178347.511	5932660.636
White-naped Crane	-5816815.201	6358030.522
White-naped Crane	-5277490.079	5876807.194
White-naped Crane	-6178363.096	5932637.798
White-naped Crane	-5816995.539	6357855.546
White-naped Crane	-6177952.327	5932071.765
White-naped Crane	-5277190.63	5876816.919
White-naped Crane	-6229892.888	5923647.639
White-naped Crane	-5275361.651	5877659.763
White-naped Crane	-5277118.272	5875919.056
White-naped Crane	-6233083.305	5917516.097
White-naped Crane	-6177686.274	5933107.618
White-naped Crane	-5816912.049	6357907.009
White-naped Crane	-5817249.347	6357929.31
White-naped Crane	-6233194.624	5916356.741
White-naped Crane	-5277280.799	5876596.496
White-naped Crane	-6177129.676	5933226.709
White-naped Crane	-5277343.138	5876677.533
White-naped Crane	-6228501.395	5906989.907
White-naped Crane	-6177529.313	5933172.873
White-naped Crane	-5816523.544	6358164.33
White-naped Crane	-5277334.232	5876669.43
White-naped Crane	-6178177.192	5932805.821
White-naped Crane	-5816477.903	6358078.555

White-naped Crane	-5279323.511	5876345.286
White-naped Crane	-6228472.452	5906993.16
White-naped Crane	-5280315.368	5875024.527
White-naped Crane	-6228508.074	5906985.027
White-naped Crane	-5820260.539	6371550.892
White-naped Crane	-6178365.322	5932644.324
White-naped Crane	-5820167.031	6371477.006
White-naped Crane	-5280313.142	5875019.665
White-naped Crane	-6228495.829	5906996.413
White-naped Crane	-6178373.115	5932637.798
White-naped Crane	-5280322.047	5874996.979
White-naped Crane	-5275328.255	5877406.901
White-naped Crane	-6178360.87	5932644.324
White-naped Crane	-5815385.859	6375854.566
White-naped Crane	-6178420.982	5932851.498
White-naped Crane	-5280327.613	5875000.22
White-naped Crane	-6228559.281	5907020.809
White-naped Crane	-5815572.876	6375589.818
White-naped Crane	-6228506.961	5906994.786
White-naped Crane	-6177809.838	5933066.834
White-naped Crane	-5275221.388	5877423.11
White-naped Crane	-5277411.042	5876834.747
White-naped Crane	-6228511.413	5906998.039
White-naped Crane	-6177690.726	5933084.779
White-naped Crane	-5275214.709	5877408.522
White-naped Crane	-5277874.132	5876659.705
White-naped Crane	-6228508.074	5906988.28
White-naped Crane	-6177373.466	5933288.701
White-naped Crane	-5815578.442	6375770.327
White-naped Crane	-6228434.603	5907079.361
White-naped Crane	-6177000.545	5933311.541
White-naped Crane	-5815553.951	6376026.484
White-naped Crane	-6228470.225	5907102.132
White-naped Crane	-6177016.13	5933455.106
White-naped Crane	-5277835.17	5876886.612
White-naped Crane	-5815252.275	6375959.435
White-naped Crane	-6177449.163	5933223.446
White-naped Crane	-6228463.546	5907067.976
White-naped Crane	-5815168.786	6375902.702
White-naped Crane	-6178367.549	5932619.854
White-naped Crane	-5277487.853	5876803.953
White-naped Crane	-5814112.364	6376452.857
White-naped Crane	-5277483.4	5876815.298
White-naped Crane	-6228512.527	5906898.827
White-naped Crane	-6178363.096	5932645.955
White-naped Crane	-5277484.513	5876815.298
White-naped Crane	-6228474.678	5906858.167
White-naped Crane	-5814093.44	6376458.015
White-naped Crane	-6178366.436	5932641.061
White-naped Crane	-5277476.721	5876823.402
White-naped Crane	-6178366.436	5932629.642
White-naped Crane	-5814081.194	6376471.769
White-naped Crane	-6228469.112	5906897.2
White-naped Crane	-5277480.061	5876825.023
White-naped Crane	-6228472.452	5906897.2
White-naped Crane	-5814107.911	6376451.138
White-naped Crane	-6178365.322	5932644.324
White-naped Crane	-5814096.779	6376470.05

White-naped Crane	-5277483.4	5876820.16
White-naped Crane	-6228482.47	5906890.695
White-naped Crane	-6178348.624	5932644.324
White-naped Crane	-5277477.834	5876823.402
White-naped Crane	-6228479.131	5906893.948
White-naped Crane	-5814091.213	6376459.734
White-naped Crane	-6178351.964	5932634.536
White-naped Crane	-5814055.591	6376411.594
White-naped Crane	-5277442.212	5876820.16
White-naped Crane	-6228472.452	5906885.816
White-naped Crane	-6178365.322	5932639.43
White-naped Crane	-6226024.536	5905201.014
White-naped Crane	-5811144.586	6375744.54
White-naped Crane	-6178167.174	5932500.772
White-naped Crane	-5277748.34	5876923.891
White-naped Crane	-5806029.456	6374625.453
White-naped Crane	-6225962.197	5905236.788
White-naped Crane	-5276913.444	5877056.797
White-naped Crane	-6176980.508	5933318.067
White-naped Crane	-5277812.906	5876818.54
White-naped Crane	-6227165.561	5904737.588
White-naped Crane	-6176951.565	5933401.269
White-naped Crane	-5277907.527	5876800.711
White-naped Crane	-6227298.031	5904687.182
White-naped Crane	-6177008.338	5933437.16
White-naped Crane	-5280745.061	5877465.253
White-naped Crane	-5815319.067	6358371.908
White-naped Crane	-5279840.034	5879391.092
White-naped Crane	-6227170.014	5904664.418
White-naped Crane	-5279551.716	5879699.136
White-naped Crane	-6177387.937	5933166.348
White-naped Crane	-6226969.638	5904731.084
White-naped Crane	-5815181.031	6358329.02
White-naped Crane	-5277311.968	5876873.646
White-naped Crane	-5815163.22	6358329.02
White-naped Crane	-6178360.87	5932641.061
White-naped Crane	-5280099.408	5873932.419
White-naped Crane	-6226713.604	5904948.972
White-naped Crane	-6227301.371	5904646.532
White-naped Crane	-5815163.22	6358317.011
White-naped Crane	-5280030.39	5873896.774
White-naped Crane	-6178360.87	5932642.692
White-naped Crane	-5280010.353	5873912.976
White-naped Crane	-6178363.096	5932650.849
White-naped Crane	-5815365.821	6358387.348
White-naped Crane	-6226065.724	5905040.031
White-naped Crane	-5815345.784	6358394.21
White-naped Crane	-6178354.19	5932660.636
White-naped Crane	-5280030.39	5873934.039
White-naped Crane	-6227089.864	5904493.69
White-naped Crane	-5279999.221	5873874.091
White-naped Crane	-6178357.53	5932645.955
White-naped Crane	-5280160.634	5873846.547
White-naped Crane	-6227102.109	5904623.768
White-naped Crane	-5815808.873	6358514.3
White-naped Crane	-5279996.994	5873865.99
White-naped Crane	-6178349.738	5932133.749
White-naped Crane	-6227158.882	5904654.662

White-naped Crane	-5815307.935	6358377.055
White-naped Crane	-6226969.638	5904836.775
White-naped Crane	-6177059.545	5931505.768
White-naped Crane	-5278001.036	5876593.255
White-naped Crane	-5815305.709	6358368.477
White-naped Crane	-6176949.338	5931474.778
White-naped Crane	-5277894.169	5876731.018
White-naped Crane	-6227244.598	5905100.196
White-naped Crane	-6177243.222	5932244.67
White-naped Crane	-6227044.223	5904698.564
White-naped Crane	-5277817.359	5876839.61
White-naped Crane	-5815164.333	6358332.451
White-naped Crane	-5815075.277	6358294.709
White-naped Crane	-6177341.183	5932252.826
White-naped Crane	-5277480.061	5876821.781
White-naped Crane	-5277481.174	5876812.056
White-naped Crane	-6178365.322	5932632.905
White-naped Crane	-5815101.994	6358095.71
White-naped Crane	-5277478.947	5876820.16
White-naped Crane	-6178363.096	5932644.324
White-naped Crane	-6228141.833	5907097.252
White-naped Crane	-5277480.061	5876818.54
White-naped Crane	-5815112.013	6358106.003
White-naped Crane	-6178365.322	5932639.43
White-naped Crane	-6228136.267	5907100.505
White-naped Crane	-5815105.334	6358116.296
White-naped Crane	-6228131.814	5907095.626
White-naped Crane	-5277483.4	5876823.402
White-naped Crane	-6178367.549	5932645.955
White-naped Crane	-5277485.626	5876820.16
White-naped Crane	-6228140.72	5907113.517
White-naped Crane	-5815179.918	6358119.727
White-naped Crane	-6178354.19	5932650.849
White-naped Crane	-5277478.947	5876826.643
White-naped Crane	-6228145.172	5907097.252
White-naped Crane	-6178360.87	5932642.692
White-naped Crane	-5815177.691	6358114.581
White-naped Crane	-6228130.701	5907098.879
White-naped Crane	-5277474.495	5876820.16
White-naped Crane	-6178361.983	5932652.48
White-naped Crane	-5815179.918	6358123.158
White-naped Crane	-5815113.126	6358119.727
White-naped Crane	-6228132.927	5907100.505
White-naped Crane	-5277494.532	5876803.953
White-naped Crane	-6178364.209	5932636.167
White-naped Crane	-6227981.533	5907107.011
White-naped Crane	-5278042.224	5876384.183
White-naped Crane	-5815201.068	6358066.547
White-naped Crane	-5814870.45	6359212.568
White-naped Crane	-5278041.111	5876405.252
White-naped Crane	-6177256.58	5933270.756
White-naped Crane	-6226185.949	5905087.187
White-naped Crane	-6176923.735	5933562.781
White-naped Crane	-5814680.093	6359706.709
White-naped Crane	-5279263.399	5875781.305
White-naped Crane	-5280127.238	5880195.273
White-naped Crane	-5814851.525	6360588.687
White-naped Crane	-5279246.701	5875513.914

White-naped Crane	-6177330.051	5933215.289
White-naped Crane	-6226790.414	5904830.271
White-naped Crane	-5279965.825	5874859.243
White-naped Crane	-5814550.963	6364145.007
White-naped Crane	-5277900.848	5876755.33
White-naped Crane	-6177602.784	5933287.07
White-naped Crane	-6227171.127	5904669.296
White-naped Crane	-5815208.861	6371178.035
White-naped Crane	-5279961.372	5874844.66
White-naped Crane	-6226526.587	5904924.581
White-naped Crane	-6177566.048	5933088.042
White-naped Crane	-5278183.6	5876619.186
White-naped Crane	-5279956.919	5874846.28
White-naped Crane	-5815169.899	6371222.708
White-naped Crane	-6178359.756	5932639.43
White-naped Crane	-6226980.77	5904820.515
White-naped Crane	-5814121.269	6372398.044
White-naped Crane	-5279970.278	5874860.864
White-naped Crane	-6226882.809	5904796.125
White-naped Crane	-5280170.653	5873841.686
White-naped Crane	-6178366.436	5932637.798
White-naped Crane	-5280010.353	5874807.391
White-naped Crane	-6178365.322	5932647.586
White-naped Crane	-5280181.785	5873848.167
White-naped Crane	-6225679.446	5905202.64
White-naped Crane	-5280175.105	5873869.23
White-naped Crane	-6227657.593	5905023.771
White-naped Crane	-6178360.87	5932652.48
White-naped Crane	-5280526.875	5873951.862
White-naped Crane	-5280676.043	5873901.635
White-naped Crane	-6178341.945	5932562.76
White-naped Crane	-6228751.864	5906308.457
White-naped Crane	-6228503.621	5906993.16
White-naped Crane	-5280662.685	5873895.154
White-naped Crane	-5280204.048	5873854.648
White-naped Crane	-6177867.724	5932086.445
White-naped Crane	-5281171.415	5873177.422
White-naped Crane	-5280246.35	5873846.547
White-naped Crane	-6228503.621	5906993.16
White-naped Crane	-5842996.432	6318594.649
White-naped Crane	-6177670.689	5932352.33
White-naped Crane	-5841817.559	6319327.135
White-naped Crane	-5281368.45	5873678.046
White-naped Crane	-6228521.432	5907095.626
White-naped Crane	-6177330.051	5932461.623
White-naped Crane	-5860817.569	6298162.253
White-naped Crane	-6228341.095	5906986.654
White-naped Crane	-6177264.373	5932564.391
White-naped Crane	-5281352.866	5873691.007
White-naped Crane	-6228508.074	5907009.424
White-naped Crane	-6177405.748	5932497.51
White-naped Crane	-5281492.015	5873697.488
White-naped Crane	-5859133.305	6301250.669
White-naped Crane	-5858869.478	6301824.87
White-naped Crane	-5281624.485	5873577.595
White-naped Crane	-6178363.096	5932642.692
White-naped Crane	-5858878.384	6301833.389
White-naped Crane	-6178356.417	5932639.43

White-naped Crane	-5281611.127	5873563.013
White-naped Crane	-6228136.267	5907098.879
White-naped Crane	-6228134.04	5907092.373
White-naped Crane	-5281893.878	5873527.369
White-naped Crane	-6178358.643	5932637.798
White-naped Crane	-5858872.818	6301833.389
White-naped Crane	-6228138.493	5907103.758
White-naped Crane	-5858871.705	6301838.501
White-naped Crane	-5281899.444	5873533.85
White-naped Crane	-6178363.096	5932644.324
White-naped Crane	-5858869.478	6301835.093
White-naped Crane	-5281898.331	5873537.09
White-naped Crane	-6228132.927	5907098.879
White-naped Crane	-6178364.209	5932639.43
White-naped Crane	-5858882.837	6301831.685
White-naped Crane	-6228136.267	5907103.758
White-naped Crane	-5281897.218	5873532.23
White-naped Crane	-6178363.096	5932642.692
White-naped Crane	-5281899.444	5873533.85
White-naped Crane	-6228132.927	5907100.505
White-naped Crane	-5858875.044	6301826.574
White-naped Crane	-6178357.53	5932662.268
White-naped Crane	-6228135.154	5907103.758
White-naped Crane	-6178359.756	5932641.061
White-naped Crane	-5858868.365	6301828.277
White-naped Crane	-5859463.924	6302504.76
White-naped Crane	-5270787.533	5873535.47
White-naped Crane	-6177618.369	5933099.461
White-naped Crane	-5859492.867	6302467.271
White-naped Crane	-6225246.413	5905415.661
White-naped Crane	-5270781.967	5873736.373
White-naped Crane	-6227942.571	5907115.143
White-naped Crane	-5859318.096	6302201.444
White-naped Crane	-6177380.145	5933223.446
White-naped Crane	-6227942.571	5907123.275
White-naped Crane	-5277964.3	5876802.332
White-naped Crane	-6227989.325	5907108.637
White-naped Crane	-6177006.111	5933393.112
White-naped Crane	-6228131.814	5907111.89
White-naped Crane	-5813483.409	6360574.959
White-naped Crane	-5280181.785	5874353.691
White-naped Crane	-5815570.649	6368091.012
White-naped Crane	-5280517.969	5873768.777
White-naped Crane	-6227633.103	5908112.22
White-naped Crane	-6178366.436	5932642.692
White-naped Crane	-5815569.536	6368233.57
White-naped Crane	-5280477.894	5873715.31
White-naped Crane	-6227521.783	5908156.139
White-naped Crane	-6178364.209	5932642.692
White-naped Crane	-5815644.12	6368257.616
White-naped Crane	-6227581.896	5908213.072
White-naped Crane	-5280421.121	5873807.662
White-naped Crane	-6178351.964	5932636.167
White-naped Crane	-5280499.045	5873938.9
White-naped Crane	-5815768.798	6368185.478
White-naped Crane	-6227606.386	5908092.7
White-naped Crane	-6178360.87	5932644.324
White-naped Crane	-6227108.788	5904573.362

White-naped Crane	-5280748.401	5874031.254
White-naped Crane	-6178370.888	5932655.742
White-naped Crane	-5815560.63	6368224.982
White-naped Crane	-5280690.515	5874050.697
White-naped Crane	-6178360.87	5932644.324
White-naped Crane	-6227149.976	5904631.898
White-naped Crane	-6178365.322	5932639.43
White-naped Crane	-5277982.111	5876742.363
White-naped Crane	-5815572.876	6368223.265
White-naped Crane	-6227165.561	5904718.076
White-naped Crane	-5814247.06	6359375.562
White-naped Crane	-5277958.734	5876966.031
White-naped Crane	-6178373.115	5932649.217
White-naped Crane	-6227159.995	5904739.214
White-naped Crane	-5815032.976	6359320.658
White-naped Crane	-5277865.226	5876966.031
White-naped Crane	-6177333.391	5932561.129
White-naped Crane	-6225949.952	5905209.144
White-naped Crane	-5814516.454	6359427.035
White-naped Crane	-5276972.444	5877141.08
White-naped Crane	-6177261.033	5932637.798
White-naped Crane	-6225899.858	5905235.162
White-naped Crane	-5277059.273	5876993.585
White-naped Crane	-5814514.227	6359415.025
White-naped Crane	-6178365.322	5932641.061
White-naped Crane	-5814516.454	6359415.025
White-naped Crane	-5272727.831	5877073.005
White-naped Crane	-6228139.606	5907093.999
White-naped Crane	-6178360.87	5932641.061
White-naped Crane	-5814530.925	6359428.751
White-naped Crane	-5272722.265	5877077.868
White-naped Crane	-6228137.38	5907102.132
White-naped Crane	-6178307.436	5932649.217
White-naped Crane	-5272734.511	5877074.626
White-naped Crane	-5814499.756	6359416.74
White-naped Crane	-6228138.493	5907100.505
White-naped Crane	-6178356.417	5932639.43
White-naped Crane	-5814336.116	6359471.645
White-naped Crane	-5272744.529	5877066.522
White-naped Crane	-6227943.684	5907103.758
White-naped Crane	-6178380.907	5932642.692
White-naped Crane	-5272748.982	5877071.384
White-naped Crane	-6227955.929	5907118.396
White-naped Crane	-5814357.267	6359490.518
White-naped Crane	-6178369.775	5932644.324
White-naped Crane	-6227955.929	5907072.855
White-naped Crane	-6178359.756	5932642.692
White-naped Crane	-5814360.606	6359497.382
White-naped Crane	-5273157.525	5877100.559
White-naped Crane	-6227998.231	5907133.034
White-naped Crane	-6178363.096	5932637.798
White-naped Crane	-5814933.902	6360521.764
White-naped Crane	-6177415.767	5932422.473
White-naped Crane	-6228041.645	5907128.155
White-naped Crane	-5278778.046	5879368.394
White-naped Crane	-6227722.158	5907835.694
White-naped Crane	-6177284.41	5932513.822
White-naped Crane	-5810258.483	6373609.639

White-naped Crane	-6177460.295	5932309.918
White-naped Crane	-5281253.791	5877019.518
White-naped Crane	-6227668.725	5908043.901
White-naped Crane	-5281244.886	5876980.619
White-naped Crane	-6227187.825	5908387.127
White-naped Crane	-5811471.865	6379720.153
White-naped Crane	-5809768.677	6375036.283
White-naped Crane	-6226581.133	5905074.179
White-naped Crane	-5282075.329	5876752.088
White-naped Crane	-6226556.643	5910591.584
White-naped Crane	-5302272.024	5872292.895
White-naped Crane	-5803980.064	6374293.71
White-naped Crane	-6178367.549	5932636.167
White-naped Crane	-5803993.422	6374288.553
White-naped Crane	-5302266.458	5872313.954
White-naped Crane	-6178363.096	5932637.798
White-naped Crane	-6226629.001	5910650.16
White-naped Crane	-5803974.498	6374297.148
White-naped Crane	-6226740.32	5915075.443
White-naped Crane	-6178365.322	5932637.798
White-naped Crane	-5811354.98	6360914.734
White-naped Crane	-6178359.756	5932641.061
White-naped Crane	-5306519.976	5866956.868
White-naped Crane	-5811316.018	6360806.623
White-naped Crane	-6178300.757	5932486.091
White-naped Crane	-5306639.088	5866765.836
White-naped Crane	-6228866.523	5915197.541
White-naped Crane	-5811312.679	6360804.907
White-naped Crane	-6228742.958	5915199.169
White-naped Crane	-6177436.918	5933161.453
White-naped Crane	-5306615.711	5866772.312
White-naped Crane	-5307140.026	5866707.556
White-naped Crane	-6177196.468	5933229.971
White-naped Crane	-6177010.564	5933311.541
White-naped Crane	-5306949.669	5866663.846
White-naped Crane	-5811635.505	6360870.117
White-naped Crane	-5812537.193	6360834.079
White-naped Crane	-5306659.125	5866508.435
White-naped Crane	-6177740.82	5933043.995
White-naped Crane	-6227699.894	5907814.549
White-naped Crane	-5813523.484	6360571.527
White-naped Crane	-6226034.555	5905210.77
White-naped Crane	-5307190.119	5863434.784
White-naped Crane	-6177893.328	5933305.015
White-naped Crane	-6226027.876	5905212.396
White-naped Crane	-5813629.237	6359679.256
White-naped Crane	-6177857.706	5933215.289
White-naped Crane	-5307563.04	5861484.979
White-naped Crane	-6227233.466	5904638.402
White-naped Crane	-5813721.632	6359780.49
White-naped Crane	-6178369.775	5932636.167
White-naped Crane	-5813622.558	6359663.814
White-naped Crane	-5306587.881	5866966.582
White-naped Crane	-6227301.371	5904646.532
White-naped Crane	-6178350.851	5932629.642
White-naped Crane	-5306581.202	5866961.725
White-naped Crane	-5813616.992	6359665.529
White-naped Crane	-6227294.691	5904734.336

White-naped Crane	-6178356.417	5932647.586
White-naped Crane	-5306575.636	5866960.106
White-naped Crane	-6227293.578	5904750.596
White-naped Crane	-5813616.992	6359677.54
White-naped Crane	-6178356.417	5932642.692
White-naped Crane	-5813620.332	6359665.529
White-naped Crane	-6227298.031	5904748.97
White-naped Crane	-5306584.541	5866953.63
White-naped Crane	-6178368.662	5932662.268
White-naped Crane	-5813630.35	6359662.098
White-naped Crane	-5306583.428	5866953.63
White-naped Crane	-6227300.257	5904740.84
White-naped Crane	-6178363.096	5932642.692
White-naped Crane	-5813640.369	6359651.803
White-naped Crane	-5306581.202	5866811.165
White-naped Crane	-6178383.134	5932639.43
White-naped Crane	-6227298.031	5904748.97
White-naped Crane	-6227298.031	5904745.718
White-naped Crane	-6178386.473	5932634.536
White-naped Crane	-5306590.107	5866812.784
White-naped Crane	-6225733.992	5905183.127
White-naped Crane	-6178400.945	5932500.772
White-naped Crane	-5813418.843	6360384.486
White-naped Crane	-6227973.74	5907113.517
White-naped Crane	-5307830.206	5861588.526
White-naped Crane	-6178365.322	5932251.195
White-naped Crane	-5814007.723	6362320.315
White-naped Crane	-6178259.569	5932169.635
White-naped Crane	-6228030.513	5907137.914
White-naped Crane	-6227987.099	5907108.637
White-naped Crane	-5307150.044	5861954.187
White-naped Crane	-5814587.698	6362279.122
White-naped Crane	-6227713.253	5907780.39
White-naped Crane	-5307152.271	5861946.097
White-naped Crane	-5813838.518	6362277.406
White-naped Crane	-5307187.893	5861949.333
White-naped Crane	-6227744.422	5907705.568
White-naped Crane	-6177155.28	5932404.529
White-naped Crane	-6178356.417	5932639.43
White-naped Crane	-6227471.689	5908405.021
White-naped Crane	-5810186.125	6375627.639
White-naped Crane	-6178364.209	5932644.324
White-naped Crane	-5306558.938	5866723.745
White-naped Crane	-6226528.813	5910576.94
White-naped Crane	-5306529.995	5867023.245
White-naped Crane	-5810190.578	6375632.796
White-naped Crane	-6178364.209	5932650.849
White-naped Crane	-6226534.379	5910585.075
White-naped Crane	-5808947.139	6373726.511
White-naped Crane	-5306533.334	5867054.005
White-naped Crane	-6178448.812	5932590.492
White-naped Crane	-5809033.969	6373767.761
White-naped Crane	-5306578.975	5866963.344
White-naped Crane	-6227945.91	5912555.732
White-naped Crane	-6178451.038	5932521.979
White-naped Crane	-6228532.564	5914880.091
White-naped Crane	-5306869.519	5867073.432
White-naped Crane	-6178492.227	5932624.748

White-naped Crane	-6178407.624	5932322.968
White-naped Crane	-6228455.754	5914617.999
White-naped Crane	-5808597.596	6372123.096
White-naped Crane	-5307853.584	5861599.852
White-naped Crane	-5809300.022	6371191.78
White-naped Crane	-5307880.3	5861594.998
White-naped Crane	-6225836.406	5905407.531
White-naped Crane	-6178356.417	5932644.324
White-naped Crane	-6225860.896	5905392.895
White-naped Crane	-6178370.888	5932647.586
White-naped Crane	-6178514.491	5932500.772
White-naped Crane	-6226020.083	5905205.892
White-naped Crane	-5303529.935	5863501.133
White-naped Crane	-5812547.212	6360209.46
White-naped Crane	-5303593.387	5863467.149
White-naped Crane	-5813632.577	6359667.245
White-naped Crane	-6228164.097	5907076.108
White-naped Crane	-5813627.011	6359670.677
White-naped Crane	-5303596.726	5863460.676
White-naped Crane	-6228135.154	5907095.626
White-naped Crane	-5303588.934	5863470.386
White-naped Crane	-6228132.927	5907110.264
White-naped Crane	-6178350.851	5932650.849
White-naped Crane	-5813630.35	6359674.108
White-naped Crane	-5303591.16	5863467.149
White-naped Crane	-6228140.72	5907100.505
White-naped Crane	-6178365.322	5932642.692
White-naped Crane	-5813618.105	6359632.929
White-naped Crane	-6228139.606	5907110.264
White-naped Crane	-5303574.462	5863496.278
White-naped Crane	-6178360.87	5932645.955
White-naped Crane	-5303584.481	5863483.332
White-naped Crane	-6228134.04	5907102.132
White-naped Crane	-6178370.888	5932634.536
White-naped Crane	-5813631.464	6359674.108
White-naped Crane	-5813627.011	6359679.256
White-naped Crane	-5303574.462	5863486.569
White-naped Crane	-6228129.588	5907089.12
White-naped Crane	-6178360.87	5932645.955
White-naped Crane	-6228128.474	5907085.867
White-naped Crane	-5303575.576	5863481.714
White-naped Crane	-5813641.482	6359687.835
White-naped Crane	-6178365.322	5932650.849
White-naped Crane	-5307820.188	5861454.239
White-naped Crane	-6228179.681	5906980.148
White-naped Crane	-6178024.685	5932538.291
White-naped Crane	-5308035.034	5861533.517
White-naped Crane	-6226023.423	5905228.657
White-naped Crane	-5811992.841	6353473.761
White-naped Crane	-5308379.012	5861209.938
White-naped Crane	-6225830.84	5905412.409
White-naped Crane	-5307993.846	5861572.347
White-naped Crane	-6177310.014	5932396.373
White-naped Crane	-5819823.054	6359862.85
White-naped Crane	-6228509.187	5914971.255
White-naped Crane	-5307260.251	5863031.842
White-naped Crane	-6177193.128	5932564.391
White-naped Crane	-6228528.111	5915024.977

White-naped Crane	-5307239.1	5863133.789
White-naped Crane	-6178347.511	5932670.424
White-naped Crane	-6228564.847	5914982.65
White-naped Crane	-5306572.296	5866900.206
White-naped Crane	-6178366.436	5932639.43
White-naped Crane	-5820180.389	6370731.316
White-naped Crane	-5306635.748	5866929.346
White-naped Crane	-6228589.337	5914937.068
White-naped Crane	-6178365.322	5932637.798
White-naped Crane	-6178359.756	5932634.536
White-naped Crane	-6228514.753	5914972.883
White-naped Crane	-6228516.979	5914972.883
White-naped Crane	-5301685.371	5867484.657
White-naped Crane	-6178467.736	5932732.413
White-naped Crane	-6178589.075	5932882.493
White-naped Crane	-5872816.697	6255809.874
White-naped Crane	-6228597.129	5915024.977
White-naped Crane	-5302180.742	5866702.699
White-naped Crane	-5302190.761	5866688.129
White-naped Crane	-6228508.074	5914891.486
White-naped Crane	-6178324.134	5932884.124
White-naped Crane	-5872302.401	6256264.027
White-naped Crane	-5871859.35	6255913.242
White-naped Crane	-6228395.641	5914645.673
White-naped Crane	-5306469.882	5861834.455
White-naped Crane	-5871034.472	6248871.853
White-naped Crane	-5307207.931	5863093.334
White-naped Crane	-6228274.303	5914505.677
White-naped Crane	-6177109.639	5932216.94
White-naped Crane	-6177013.904	5932205.522
White-naped Crane	-5307286.967	5862942.841
White-naped Crane	-5871070.094	6248836.296
White-naped Crane	-5871221.489	6248924.34
White-naped Crane	-5305911.058	5865661.818
White-naped Crane	-6178363.096	5932649.217
White-naped Crane	-6228854.277	5915490.582
White-naped Crane	-5305938.888	5865669.912
White-naped Crane	-5871191.433	6249113.977
White-naped Crane	-6228781.92	5915288.708
White-naped Crane	-6178377.568	5932644.324
White-naped Crane	-5871191.433	6249120.749
White-naped Crane	-5305937.775	5865666.674
White-naped Crane	-6228784.146	5915290.336
White-naped Crane	-6178364.209	5932645.955
White-naped Crane	-5305893.247	5865655.343
White-naped Crane	-6178364.209	5932642.692
White-naped Crane	-6228758.543	5915275.684
White-naped Crane	-5871184.754	6249120.749
White-naped Crane	-5305838.701	5865635.919
White-naped Crane	-6228768.561	5915283.824
White-naped Crane	-5871225.942	6249091.965
White-naped Crane	-6178365.322	5932642.692
White-naped Crane	-5871185.867	6249117.363
White-naped Crane	-5305837.588	5865635.919
White-naped Crane	-6228764.109	5915274.056
White-naped Crane	-6178356.417	5932636.167
White-naped Crane	-5305834.248	5865635.919
White-naped Crane	-6228790.825	5915296.848

White-naped Crane	-6178360.87	5932644.324
White-naped Crane	-5305838.701	5865631.063
White-naped Crane	-6228766.335	5915278.94
White-naped Crane	-5871189.206	6249119.056
White-naped Crane	-6178355.304	5932644.324
White-naped Crane	-5872537.285	6250993.64
White-naped Crane	-6228787.486	5915288.708
White-naped Crane	-5305816.437	5865619.732
White-naped Crane	-6178356.417	5932654.111
White-naped Crane	-5277955.395	5876786.124
White-naped Crane	-6228783.033	5915288.708
White-naped Crane	-6178364.209	5932652.48
White-naped Crane	-6228784.146	5915301.732
White-naped Crane	-5871095.698	6248704.232
White-naped Crane	-6178476.642	5932551.341
White-naped Crane	-5870900.889	6246740.446
White-naped Crane	-6226028.989	5905214.022
White-naped Crane	-5304729.959	5867418.277
White-naped Crane	-6177384.598	5931926.592
White-naped Crane	-6225641.597	5905453.063
White-naped Crane	-5304804.543	5867468.466
White-naped Crane	-5870994.397	6246708.285
White-naped Crane	-5870965.454	6246716.748
White-naped Crane	-5304536.263	5867559.132
White-naped Crane	-6227161.108	5904740.84
White-naped Crane	-6177369.013	5932590.492
White-naped Crane	-6227168.9	5904742.466
White-naped Crane	-5304721.053	5867405.324
White-naped Crane	-6177405.748	5933411.057
White-naped Crane	-5871754.709	6248780.423
White-naped Crane	-6227156.655	5904905.069
White-naped Crane	-5302305.42	5866176.577
White-naped Crane	-6177200.921	5933500.786
White-naped Crane	-5279271.191	5876066.532
White-naped Crane	-6225551.428	5905072.553
White-naped Crane	-5881209.074	6254927.057
White-naped Crane	-5302326.571	5866199.24
White-naped Crane	-5302338.816	5866194.384
White-naped Crane	-6225453.467	5905022.145
White-naped Crane	-5879435.754	6253503.902
White-naped Crane	-6177294.429	5933257.705
White-naped Crane	-6227526.236	5904979.867
White-naped Crane	-5300054.54	5866692.986
White-naped Crane	-5878483.973	6254113.796
White-naped Crane	-6177312.24	5933123.932
White-naped Crane	-6225473.505	5905105.074
White-naped Crane	-6177268.825	5933148.402
White-naped Crane	-5307379.363	5862567.431
White-naped Crane	-5875077.596	6257741.89
White-naped Crane	-6178358.643	5932639.43
White-naped Crane	-5872212.232	6257507.99
White-naped Crane	-5306909.594	5866152.296
White-naped Crane	-6225255.318	5905148.979
White-naped Crane	-6178361.983	5932637.798
White-naped Crane	-5306741.502	5866607.186
White-naped Crane	-6225074.981	5905178.248
White-naped Crane	-5870857.474	6246503.469
White-naped Crane	-6178354.19	5932632.905

White-naped Crane	-5870969.907	6246708.285
White-naped Crane	-5306690.295	5866498.722
White-naped Crane	-6225141.772	5905223.779
White-naped Crane	-6178398.718	5932634.536
White-naped Crane	-5278001.036	5876666.188
White-naped Crane	-6227301.371	5904659.54
White-naped Crane	-5870933.172	6246691.358
White-naped Crane	-5306741.502	5866613.661
White-naped Crane	-6177258.807	5932236.514
White-naped Crane	-5306738.162	5866599.091
White-naped Crane	-5871131.32	6248150.601
White-naped Crane	-6227282.446	5904670.922
White-naped Crane	-6178366.436	5932642.692
White-naped Crane	-5306737.049	5866605.567
White-naped Crane	-6227299.144	5904648.158
White-naped Crane	-6178367.549	5932641.061
White-naped Crane	-5871804.803	6248841.376
White-naped Crane	-5306739.275	5866605.567
White-naped Crane	-6227223.447	5904636.776
White-naped Crane	-5306740.389	5866608.805
White-naped Crane	-6227216.768	5904644.906
White-naped Crane	-6178364.209	5932647.586
White-naped Crane	-5306742.615	5866612.042
White-naped Crane	-6227220.107	5904640.028
White-naped Crane	-5871789.218	6248849.842
White-naped Crane	-6178376.454	5932641.061
White-naped Crane	-5871790.332	6248844.762
White-naped Crane	-5306739.275	5866607.186
White-naped Crane	-6227217.881	5904635.15
White-naped Crane	-6178367.549	5932631.273
White-naped Crane	-5871801.464	6248858.307
White-naped Crane	-5306743.728	5866605.567
White-naped Crane	-6227294.691	5904748.97
White-naped Crane	-6178374.228	5932634.536
White-naped Crane	-5871788.105	6248851.535
White-naped Crane	-6227293.578	5904748.97
White-naped Crane	-6178356.417	5932634.536
White-naped Crane	-6225191.866	5905225.405
White-naped Crane	-5307362.665	5862538.305
White-naped Crane	-6178429.888	5932468.148
White-naped Crane	-5870834.097	6246845.396
White-naped Crane	-6225168.489	5905199.388
White-naped Crane	-6177185.336	5932137.012
White-naped Crane	-5307681.038	5862429.892
White-naped Crane	-5307341.514	5862541.541
White-naped Crane	-6225310.978	5905233.536
White-naped Crane	-6177001.659	5932327.862
White-naped Crane	-5307122.215	5861870.051
White-naped Crane	-6177291.089	5933353.958
White-naped Crane	-6226305.061	5905033.527
White-naped Crane	-5307288.081	5861646.772
White-naped Crane	-6225798.557	5905251.423
White-naped Crane	-5870986.605	6246913.106
White-naped Crane	-6177186.449	5933274.019
White-naped Crane	-5871137.999	6248118.434
White-naped Crane	-5307192.346	5861282.742
White-naped Crane	-6227133.278	5904581.492
White-naped Crane	-6177101.846	5933760.188

White-naped Crane	-5307156.724	5861242.296
White-naped Crane	-6177049.526	5933592.147
White-naped Crane	-5873309.843	6263242.103
White-naped Crane	-6227084.298	5904591.248
White-naped Crane	-5280159.521	5875056.936
White-naped Crane	-5873532.482	6263415.103
White-naped Crane	-6177434.691	5933158.191
White-naped Crane	-5307537.436	5861434.824
White-naped Crane	-6226345.136	5905018.893
White-naped Crane	-5307151.158	5861269.799
White-naped Crane	-6228503.621	5907001.292
White-naped Crane	-6178361.983	5932626.38
White-naped Crane	-6228504.734	5906996.413
White-naped Crane	-6178361.983	5932649.217
White-naped Crane	-5881784.595	6323954.042
White-naped Crane	-5307209.044	5861292.45
White-naped Crane	-6228506.961	5906999.665
White-naped Crane	-6178366.436	5932644.324
White-naped Crane	-5307837.999	5861389.523
White-naped Crane	-6178365.322	5932649.217
White-naped Crane	-5278077.846	5876601.358
White-naped Crane	-5881747.86	6323918.165
White-naped Crane	-5307785.679	5861357.165
White-naped Crane	-6228539.243	5906864.672
White-naped Crane	-6178364.209	5932632.905
White-naped Crane	-5881273.639	6323943.791
White-naped Crane	-5307770.094	5861501.158
White-naped Crane	-6228502.508	5906996.413
White-naped Crane	-6178366.436	5932652.48
White-naped Crane	-5306968.594	5866263.992
White-naped Crane	-6177151.94	5933691.666
White-naped Crane	-6228441.282	5907090.746
White-naped Crane	-5306916.273	5866573.189
White-naped Crane	-6176811.302	5933714.507
White-naped Crane	-6228493.602	5907093.999
White-naped Crane	-5881861.406	6324205.188
White-naped Crane	-5306841.689	5866628.231
White-naped Crane	-5881869.198	6324198.354
White-naped Crane	-6228434.603	5906898.827
White-naped Crane	-6178363.096	5932642.692
White-naped Crane	-5881861.406	6324206.897
White-naped Crane	-5306838.35	5866634.706
White-naped Crane	-6228534.791	5906869.551
White-naped Crane	-6178360.87	5932641.061
White-naped Crane	-5306836.123	5866634.706
White-naped Crane	-6228535.904	5906877.684
White-naped Crane	-6178343.059	5932650.849
White-naped Crane	-5881856.953	6324210.314
White-naped Crane	-6228541.47	5906884.189
White-naped Crane	-5306837.237	5866637.944
White-naped Crane	-6178363.096	5932642.692
White-naped Crane	-6228545.922	5906880.936
White-naped Crane	-5306839.463	5866642.801
White-naped Crane	-6178364.209	5932642.692
White-naped Crane	-5881859.18	6324191.52
White-naped Crane	-5306839.463	5866636.325
White-naped Crane	-6228542.583	5906877.684
White-naped Crane	-6178357.53	5932642.692

White-naped Crane	-6228540.357	5906872.804
White-naped Crane	-5306797.162	5866620.137
White-naped Crane	-5881861.406	6324188.103
White-naped Crane	-6178364.209	5932634.536
White-naped Crane	-5306788.256	5866590.997
White-naped Crane	-5881854.727	6324191.52
White-naped Crane	-6228540.357	5906876.057
White-naped Crane	-6178364.209	5932636.167
White-naped Crane	-6228553.715	5907085.867
White-naped Crane	-6177560.483	5933221.814
White-naped Crane	-5307150.044	5862811.769
White-naped Crane	-5882206.496	6324085.594
White-naped Crane	-6176792.378	5933787.924
White-naped Crane	-5307115.535	5862755.134
White-naped Crane	-6225369.977	5905197.761
White-naped Crane	-5307606.454	5862614.356
White-naped Crane	-5881723.37	6323936.958
White-naped Crane	-6225217.47	5905179.874
White-naped Crane	-6176776.793	5933647.616
White-naped Crane	-6225247.526	5905174.996
White-naped Crane	-5881694.427	6323960.876
White-naped Crane	-6176639.87	5933660.668
White-naped Crane	-5307071.008	5863124.08
White-naped Crane	-5307193.459	5863460.676
White-naped Crane	-6228486.923	5906994.786
White-naped Crane	-6176825.774	5933902.129
White-naped Crane	-6228604.922	5907105.384
White-naped Crane	-5306811.633	5863459.058
White-naped Crane	-6177004.998	5933823.817
White-naped Crane	-5883642.518	6324259.861
White-naped Crane	-5883482.218	6324324.784
White-naped Crane	-5306993.084	5863331.215
White-naped Crane	-6176883.66	5933562.781
White-naped Crane	-6228462.433	5907107.011
White-naped Crane	-5307164.516	5863480.095
White-naped Crane	-5881520.768	6323909.623
White-naped Crane	-6176950.452	5933673.72
White-naped Crane	-6228294.34	5907077.735
White-naped Crane	-6228180.795	5907048.459
White-naped Crane	-6177350.089	5933287.07
White-naped Crane	-5882271.062	6324051.424
White-naped Crane	-6228512.527	5906952.499
White-naped Crane	-6177082.922	5931613.419
White-naped Crane	-5306422.015	5863465.531
White-naped Crane	-5882275.514	6324070.218
White-naped Crane	-6177790.914	5931911.912
White-naped Crane	-5306466.543	5863447.73
White-naped Crane	-6229062.445	5906796.363
White-naped Crane	-5882253.251	6324051.424
White-naped Crane	-5306174.886	5865739.516
White-naped Crane	-6229204.934	5906680.89
White-naped Crane	-6178360.87	5932637.798
White-naped Crane	-5306192.697	5865627.826
White-naped Crane	-6228503.621	5907082.614
White-naped Crane	-6178363.096	5932636.167
White-naped Crane	-5303521.029	5866935.822
White-naped Crane	-6228167.436	5907092.373
White-naped Crane	-5881950.462	6324007.004

White-naped Crane	-6178360.87	5932642.692
White-naped Crane	-5881707.785	6324147.099
White-naped Crane	-6228269.85	5907056.591
White-naped Crane	-6178360.87	5932636.167
White-naped Crane	-5300346.197	5860603.261
White-naped Crane	-5879009.401	6323516.69
White-naped Crane	-6178358.643	5932644.324
White-naped Crane	-6226034.555	5905218.901
White-naped Crane	-5879554.866	6323504.732
White-naped Crane	-5300731.363	5863703.422
White-naped Crane	-6227987.099	5907107.011
White-naped Crane	-6178490	5932504.035
White-naped Crane	-5883488.897	6324358.955
White-naped Crane	-6228138.493	5907097.252
White-naped Crane	-6178503.359	5932433.892
White-naped Crane	-5300735.815	5863708.277
White-naped Crane	-5883488.897	6324343.578
White-naped Crane	-5300733.589	5863727.697
White-naped Crane	-6178343.059	5932650.849
White-naped Crane	-6228136.267	5907108.637
White-naped Crane	-5883488.897	6324364.081
White-naped Crane	-5300733.589	5863727.697
White-naped Crane	-6228145.172	5907103.758
White-naped Crane	-6178369.775	5932647.586
White-naped Crane	-6228140.72	5907100.505
White-naped Crane	-5300735.815	5863734.17
White-naped Crane	-5883493.35	6324358.955
White-naped Crane	-6178354.19	5932626.38
White-naped Crane	-5300749.174	5863732.552
White-naped Crane	-6228140.72	5907098.879
White-naped Crane	-6178356.417	5932634.536
White-naped Crane	-5883477.765	6324379.457
White-naped Crane	-6228142.946	5907102.132
White-naped Crane	-5300742.495	5863730.933
White-naped Crane	-5883486.67	6324360.663
White-naped Crane	-6178363.096	5932642.692
White-naped Crane	-5883484.444	6324353.829
White-naped Crane	-5300731.363	5863680.765
White-naped Crane	-6228138.493	5907102.132
White-naped Crane	-6178358.643	5932634.536
White-naped Crane	-5300771.438	5863643.544
White-naped Crane	-6228178.568	5907056.591
White-naped Crane	-6178456.604	5932572.548
White-naped Crane	-5878911.439	6323328.773
White-naped Crane	-6225992.253	5905161.987
White-naped Crane	-5304380.415	5866686.511
White-naped Crane	-5879018.306	6323438.106
White-naped Crane	-6177826.536	5931996.731
White-naped Crane	-5278003.262	5876562.461
White-naped Crane	-6177942.308	5932388.217
White-naped Crane	-5307016.461	5862719.535
White-naped Crane	-5879232.04	6323653.36
White-naped Crane	-6228002.683	5907118.396
White-naped Crane	-6177285.523	5932282.188
White-naped Crane	-5879579.356	6323518.399
White-naped Crane	-5306807.18	5862971.969
White-naped Crane	-5306680.276	5866846.781
White-naped Crane	-5879445.773	6323566.233

White-naped Crane	-6228009.363	5907111.89
White-naped Crane	-6178171.627	5932505.666
White-naped Crane	-6178222.833	5932616.592
White-naped Crane	-5306949.669	5866581.284
White-naped Crane	-6228009.363	5907108.637
White-naped Crane	-6228505.847	5906991.533
White-naped Crane	-6178033.59	5932771.564
White-naped Crane	-6178051.401	5933083.148
White-naped Crane	-5981536.878	6343050.812
White-naped Crane	-6228525.885	5914984.278
White-naped Crane	-5307105.517	5863031.842
White-naped Crane	-5307141.139	5862685.554
White-naped Crane	-6228548.149	5915023.349
White-naped Crane	-6178251.777	5933198.975
White-naped Crane	-6046062.108	6340133.428
White-naped Crane	-5307437.249	5866519.767
White-naped Crane	-6045988.637	6340177.934
White-naped Crane	-6228545.922	5915021.721
White-naped Crane	-6178038.043	5933205.501
White-naped Crane	-6046017.58	6340143.698
White-naped Crane	-5307383.815	5866586.14
White-naped Crane	-6178176.079	5933158.191
White-naped Crane	-6228545.922	5915002.185
White-naped Crane	-5307372.683	5866539.193
White-naped Crane	-6058908.377	6332251.001
White-naped Crane	-6228550.375	5914982.65
White-naped Crane	-6178156.042	5933197.344
White-naped Crane	-6059721.009	6331914.107
White-naped Crane	-5307530.757	5866471.202
White-naped Crane	-6228543.696	5915003.813
White-naped Crane	-6178369.775	5932637.798
White-naped Crane	-6059700.972	6331917.527
White-naped Crane	-6228530.338	5914981.022
White-naped Crane	-6178360.87	5932641.061
White-naped Crane	-5307352.646	5866531.099
White-naped Crane	-6059647.538	6332050.915
White-naped Crane	-5307348.193	5866542.431
White-naped Crane	-6178360.87	5932650.849
White-naped Crane	-5306849.482	5866649.276
White-naped Crane	-6059700.972	6332071.436
White-naped Crane	-6178365.322	5932644.324
White-naped Crane	-5306740.389	5866613.661
White-naped Crane	-6178374.228	5932642.692
White-naped Crane	-6228145.172	5914741.718
White-naped Crane	-6059594.105	6331837.153
White-naped Crane	-6059594.105	6331838.863
White-naped Crane	-5306738.162	5866608.805
White-naped Crane	-6228145.172	5914733.579
White-naped Crane	-6178360.87	5932637.798
White-naped Crane	-5306738.162	5866603.948
White-naped Crane	-6059562.935	6331828.603
White-naped Crane	-6178365.322	5932639.43
White-naped Crane	-6228140.72	5914762.881
White-naped Crane	-6059574.067	6331818.342
White-naped Crane	-5306738.162	5866610.423
White-naped Crane	-6228149.625	5914722.184
White-naped Crane	-6178363.096	5932644.324
White-naped Crane	-6059589.652	6331826.893

White-naped Crane	-5306747.068	5866610.423
White-naped Crane	-6228156.304	5914733.579
White-naped Crane	-6178365.322	5932650.849
White-naped Crane	-5306744.841	5866610.423
White-naped Crane	-6228145.172	5914733.579
White-naped Crane	-6059578.52	6331826.893
White-naped Crane	-6178363.096	5932649.217
White-naped Crane	-5306747.068	5866613.661
White-naped Crane	-6228154.078	5914731.951
White-naped Crane	-6059580.747	6331818.342
White-naped Crane	-6178372.002	5932650.849
White-naped Crane	-6059560.709	6331828.603
White-naped Crane	-6228154.078	5914723.812
White-naped Crane	-5306744.841	5866607.186
White-naped Crane	-6178363.096	5932641.061
White-naped Crane	-5307081.026	5862641.864
White-naped Crane	-6226018.97	5916892.435
White-naped Crane	-6059618.595	6332015.002
White-naped Crane	-6178291.852	5932482.829
White-naped Crane	-6059647.538	6332081.697
White-naped Crane	-6226030.102	5905205.892
White-naped Crane	-6178176.079	5932508.929
White-naped Crane	-6059616.369	6331826.893
White-naped Crane	-5307063.215	5862905.623
White-naped Crane	-6225927.688	5905280.693
White-naped Crane	-6177419.107	5932282.188
White-naped Crane	-6059591.879	6332095.378
White-naped Crane	-5306898.462	5866560.239
White-naped Crane	-6177374.579	5932278.925
White-naped Crane	-6225756.256	5905448.184
White-naped Crane	-6059843.461	6331236.94
White-naped Crane	-6228585.997	5915031.488
White-naped Crane	-5306787.143	5866597.472
White-naped Crane	-6177232.09	5932228.358
White-naped Crane	-5306902.915	5866586.14
White-naped Crane	-6177719.669	5932659.005
White-naped Crane	-6228543.696	5915007.069
White-naped Crane	-5307902.564	5861266.564
White-naped Crane	-6062570.788	6328395.492
White-naped Crane	-6228585.997	5915021.721
White-naped Crane	-6177744.16	5932655.742
White-naped Crane	-5307635.397	5861273.035
White-naped Crane	-6228528.111	5914925.672
White-naped Crane	-6177726.349	5932662.268
White-naped Crane	-5307263.59	5861358.783
White-naped Crane	-6178367.549	5932634.536
White-naped Crane	-5307043.178	5866498.722
White-naped Crane	-6228223.096	5916228.114
White-naped Crane	-6178369.775	5932634.536
White-naped Crane	-6178367.549	5932641.061
White-naped Crane	-5306967.48	5866696.224
White-naped Crane	-6228334.415	5917177.392
White-naped Crane	-5961519.407	6159925.988
White-naped Crane	-6178365.322	5932641.061
White-naped Crane	-5306894.01	5866667.084
White-naped Crane	-6228826.448	5916176.013
White-naped Crane	-5306867.293	5866688.129
White-naped Crane	-6178363.096	5932639.43

White-naped Crane	-6228630.525	5915734.791
White-naped Crane	-5961212.165	6160572.69
White-naped Crane	-6178449.925	5932673.687
White-naped Crane	-5306891.783	5866702.699
White-naped Crane	-6228365.585	5915459.65
White-naped Crane	-5307844.678	5861035.211
White-naped Crane	-5959317.508	6160815.636
White-naped Crane	-6178514.491	5932787.877
White-naped Crane	-6228053.89	5914593.581
White-naped Crane	-5303253.862	5863232.501
White-naped Crane	-6228318.831	5907082.614
White-naped Crane	-6178349.738	5932637.798
White-naped Crane	-5306853.935	5866644.42
White-naped Crane	-6178363.096	5932631.273
White-naped Crane	-5955871.056	6159006.281
White-naped Crane	-5955864.377	6158999.581
White-naped Crane	-5306851.708	5866636.325
White-naped Crane	-6178363.096	5932632.905
White-naped Crane	-6228162.983	5907074.482
White-naped Crane	-5955864.377	6159021.358
White-naped Crane	-5306851.708	5866644.42
White-naped Crane	-6178363.096	5932637.798
White-naped Crane	-6228165.21	5907084.241
White-naped Crane	-5306851.708	5866647.657
White-naped Crane	-6228142.946	5907110.264
White-naped Crane	-5955871.056	6159009.632
White-naped Crane	-6178372.002	5932642.692
White-naped Crane	-5955868.83	6159011.307
White-naped Crane	-5306856.161	5866652.514
White-naped Crane	-6228142.946	5907108.637
White-naped Crane	-6178360.87	5932645.955
White-naped Crane	-5955857.698	6159012.982
White-naped Crane	-5306853.935	5866649.276
White-naped Crane	-6228138.493	5907102.132
White-naped Crane	-6178367.549	5932631.273
White-naped Crane	-6228136.267	5907102.132
White-naped Crane	-5306856.161	5866646.039
White-naped Crane	-5955868.83	6159019.682
White-naped Crane	-6178354.19	5932637.798
White-naped Crane	-5306862.84	5866646.039
White-naped Crane	-6228140.72	5907093.999
White-naped Crane	-5955866.603	6158994.555
White-naped Crane	-6178356.417	5932647.586
White-naped Crane	-5952705.13	6159807.04
White-naped Crane	-6226023.423	5905197.761
White-naped Crane	-5307860.263	5861404.084
White-naped Crane	-6178423.209	5932520.347
White-naped Crane	-6178443.246	5932207.153
White-naped Crane	-6226132.516	5905163.613
White-naped Crane	-5307504.04	5861103.16
White-naped Crane	-5307630.945	5861284.36
White-naped Crane	-6226210.44	5905049.788
White-naped Crane	-6177546.011	5931615.05
White-naped Crane	-5953027.956	6158215.648
White-naped Crane	-5306212.734	5861064.332
White-naped Crane	-6226174.817	5905041.657
White-naped Crane	-5953388.632	6157394.942
White-naped Crane	-5306157.075	5861148.46

White-naped Crane	-6227192.277	5904674.174
White-naped Crane	-6177764.197	5932543.185
White-naped Crane	-5953388.632	6157389.918
White-naped Crane	-6227216.768	5904879.052
White-naped Crane	-5306045.755	5861007.708
White-naped Crane	-6228552.602	5914984.278
White-naped Crane	-6177717.443	5932659.005
White-naped Crane	-6178354.19	5932647.586
White-naped Crane	-6228514.753	5914981.022
White-naped Crane	-5307274.722	5866658.99
White-naped Crane	-5280157.294	5873768.777
White-naped Crane	-6228572.639	5915021.721
White-naped Crane	-6178369.775	5932641.061
White-naped Crane	-5279549.49	5875928.78
White-naped Crane	-5280157.294	5873827.104
White-naped Crane	-5928232.653	6254977.889
White-naped Crane	-6228516.979	5914982.65
White-naped Crane	-6178358.643	5932650.849
White-naped Crane	-5928457.518	6254840.644
White-naped Crane	-5279549.49	5875941.744
White-naped Crane	-5280146.162	5873784.979
White-naped Crane	-6228534.791	5915028.233
White-naped Crane	-6178360.87	5932642.692
White-naped Crane	-5928372.916	6254803.369
White-naped Crane	-5280108.314	5874028.013
White-naped Crane	-6226479.833	5915090.095
White-naped Crane	-6178367.549	5932623.117
White-naped Crane	-5277993.243	5875018.045
White-naped Crane	-6226586.699	5915143.818
White-naped Crane	-6178367.549	5932637.798
White-naped Crane	-5928343.972	6262957.17
White-naped Crane	-5276969.104	5876841.23
White-naped Crane	-6178363.096	5932641.061
White-naped Crane	-6226684.661	5914762.881
White-naped Crane	-5277997.696	5876991.964
White-naped Crane	-6228131.814	5914746.602
White-naped Crane	-6178374.228	5932659.005
White-naped Crane	-5927159.533	6265477.811
White-naped Crane	-6228476.904	5906898.827
White-naped Crane	-5278516.445	5878184.961
White-naped Crane	-6178369.775	5932645.955
White-naped Crane	-5278509.766	5878184.961
White-naped Crane	-5926990.327	6265430.308
White-naped Crane	-6178211.702	5932784.614
White-naped Crane	-6228414.565	5906867.925
White-naped Crane	-5927337.644	6265340.394
White-naped Crane	-5278509.766	5878189.824
White-naped Crane	-6228421.245	5906906.959
White-naped Crane	-6178356.417	5932632.905
White-naped Crane	-5278511.992	5878196.308
White-naped Crane	-6178349.738	5932645.955
White-naped Crane	-6228425.697	5906908.585
White-naped Crane	-5927326.512	6265348.877
White-naped Crane	-5278507.539	5878202.792
White-naped Crane	-6228430.15	5906911.838
White-naped Crane	-6178365.322	5932644.324
White-naped Crane	-5927326.512	6265357.359
White-naped Crane	-5278511.992	5878206.034

White-naped Crane	-6228479.131	5906887.442
White-naped Crane	-6178369.775	5932641.061
White-naped Crane	-5927339.871	6265352.27
White-naped Crane	-5278518.671	5878191.445
White-naped Crane	-6228467.999	5906887.442
White-naped Crane	-6178363.096	5932632.905
White-naped Crane	-6228474.678	5906887.442
White-naped Crane	-5927328.739	6265374.324
White-naped Crane	-5278498.634	5878199.55
White-naped Crane	-6178427.661	5932678.58
White-naped Crane	-5927339.871	6265372.627
White-naped Crane	-5278505.313	5878194.687
White-naped Crane	-6228474.678	5906882.563
White-naped Crane	-6178378.681	5932642.692
White-naped Crane	-6226032.328	5905217.275
White-naped Crane	-6178385.36	5932513.822
White-naped Crane	-5277890.829	5876828.264
White-naped Crane	-6225197.432	5905201.014
White-naped Crane	-6178345.285	5932425.735
White-naped Crane	-5925988.452	6268939.417
White-naped Crane	-5277053.707	5877006.552
White-naped Crane	-6228191.926	5907072.855
White-naped Crane	-5919407.244	6272770.915
White-naped Crane	-6227984.872	5907120.023
White-naped Crane	-5277129.404	5877121.63
White-naped Crane	-6227982.646	5907110.264
White-naped Crane	-5277187.29	5877189.705
White-naped Crane	-6228027.174	5907139.54
White-naped Crane	-5277844.075	5876889.854
White-naped Crane	-5277723.85	5876936.857
White-naped Crane	-6227991.551	5907108.637
White-naped Crane	-5277832.943	5877030.864
White-naped Crane	-5278425.163	5878196.308
White-naped Crane	-6228058.343	5907136.287
White-naped Crane	-5807092.557	6343073.073
White-naped Crane	-5278431.842	5878191.445
White-naped Crane	-6228214.19	5907074.482
White-naped Crane	-5278391.767	5878363.276
White-naped Crane	-6228499.168	5906991.533
White-naped Crane	-5278645.576	5878191.445
White-naped Crane	-5803419.014	6346130.242
White-naped Crane	-6228503.621	5906985.027
White-naped Crane	-6228439.056	5906869.551
White-naped Crane	-5807415.383	6352967.989
White-naped Crane	-6177855.479	5932838.448
White-naped Crane	-5278124.6	5876833.127
White-naped Crane	-5807232.819	6353232.015
White-naped Crane	-6177786.461	5933061.94
White-naped Crane	-5278064.488	5876875.267
White-naped Crane	-6228501.395	5907076.108
White-naped Crane	-5278017.734	5876841.23
White-naped Crane	-5807321.875	6353202.869
White-naped Crane	-6228140.72	5907105.384
White-naped Crane	-6177802.046	5933107.618
White-naped Crane	-5277950.942	5876831.506
White-naped Crane	-5278525.351	5877405.28
White-naped Crane	-6226014.517	5905227.031
White-naped Crane	-5807402.025	6352978.276

White-naped Crane	-6177786.461	5933241.391
White-naped Crane	-5278560.973	5877552.782
White-naped Crane	-5807413.157	6353226.872
White-naped Crane	-6178360.87	5932637.798
White-naped Crane	-6226580.02	5904666.044
White-naped Crane	-5807410.93	6353204.584
White-naped Crane	-6226582.247	5904662.792
White-naped Crane	-5278558.746	5877526.847
White-naped Crane	-6178363.096	5932647.586
White-naped Crane	-5807393.119	6353204.584
White-naped Crane	-5278567.652	5877546.298
White-naped Crane	-6178356.417	5932641.061
White-naped Crane	-6226517.681	5904779.864
White-naped Crane	-5278572.105	5877547.919
White-naped Crane	-6226515.455	5904799.377
White-naped Crane	-5807422.062	6353216.585
White-naped Crane	-6178369.775	5932644.324
White-naped Crane	-5278572.105	5877549.54
White-naped Crane	-6226519.908	5904796.125
White-naped Crane	-5807415.383	6353211.442
White-naped Crane	-6178376.454	5932637.798
White-naped Crane	-5278567.652	5877549.54
White-naped Crane	-6226513.229	5904784.742
White-naped Crane	-6178360.87	5932636.167
White-naped Crane	-5807406.478	6353228.586
White-naped Crane	-5807413.157	6353216.585
White-naped Crane	-5278569.878	5877544.677
White-naped Crane	-6226515.455	5904787.995
White-naped Crane	-6178358.643	5932645.955
White-naped Crane	-5278565.426	5877536.573
White-naped Crane	-5807430.968	6353199.44
White-naped Crane	-6226515.455	5904783.116
White-naped Crane	-6178376.454	5932678.58
White-naped Crane	-6225994.48	5905103.448
White-naped Crane	-5808887.027	6353533.77
White-naped Crane	-6178363.096	5932636.167
White-naped Crane	-5809835.469	6354075.583
White-naped Crane	-6178365.322	5932645.955
White-naped Crane	-5278039.998	5876995.206
White-naped Crane	-5811456.281	6353484.048
White-naped Crane	-5277942.036	5876841.23
White-naped Crane	-6178365.322	5932631.273
White-naped Crane	-6225816.369	5905033.527
White-naped Crane	-5812821.058	6355166.176
White-naped Crane	-6225480.184	5905009.136
White-naped Crane	-5278082.299	5877006.552
White-naped Crane	-6178380.907	5932628.011
White-naped Crane	-5811387.263	6356354.674
White-naped Crane	-5278594.369	5877397.176
White-naped Crane	-6225580.371	5905181.5
White-naped Crane	-6178193.89	5932512.191
White-naped Crane	-5278632.217	5877504.155
White-naped Crane	-6178265.135	5932597.017
White-naped Crane	-6226535.492	5910746.161
White-naped Crane	-5278629.991	5877337.203
White-naped Crane	-5810806.175	6354953.533
White-naped Crane	-5278035.545	5876953.065
White-naped Crane	-6178383.134	5932637.798

White-naped Crane	-5811211.378	6354679.164
White-naped Crane	-5278100.11	5876602.979
White-naped Crane	-5278111.242	5877079.488
White-naped Crane	-6226526.587	5911037.424
White-naped Crane	-6178363.096	5932644.324
White-naped Crane	-5278616.633	5878204.413
White-naped Crane	-6226635.68	5911065.087
White-naped Crane	-6178354.19	5932641.061
White-naped Crane	-5278614.406	5878188.203
White-naped Crane	-5810852.929	6354845.499
White-naped Crane	-6228105.097	5914681.486
White-naped Crane	-6178360.87	5932652.48
White-naped Crane	-5279046.326	5876502.494
White-naped Crane	-5810870.74	6354864.362
White-naped Crane	-5278908.29	5877496.05
White-naped Crane	-6228125.135	5914705.905
White-naped Crane	-6178229.513	5932504.035
White-naped Crane	-5810910.815	6355064.999
White-naped Crane	-5278122.374	5876792.607
White-naped Crane	-6178213.928	5932484.46
White-naped Crane	-6227973.74	5914653.812
White-naped Crane	-5278914.969	5877675.972
White-naped Crane	-6227978.193	5914663.58
White-naped Crane	-6178260.682	5932564.391
White-naped Crane	-5810930.853	6354886.655
White-naped Crane	-5810973.154	6354878.081
White-naped Crane	-5278398.446	5878144.435
White-naped Crane	-6228608.261	5915054.28
White-naped Crane	-6178365.322	5932642.692
White-naped Crane	-5278407.352	5878165.509
White-naped Crane	-5811200.246	6354638.009
White-naped Crane	-6178360.87	5932645.955
White-naped Crane	-6228864.296	5915363.597
White-naped Crane	-5810821.76	6355152.457
White-naped Crane	-6228937.767	5915396.157
White-naped Crane	-5278538.709	5878240.076
White-naped Crane	-6178367.549	5932644.324
White-naped Crane	-5811451.828	6354543.697
White-naped Crane	-5278420.71	5878212.519
White-naped Crane	-6178356.417	5932645.955
White-naped Crane	-6228926.635	5915485.698
White-naped Crane	-5811182.435	6354715.174
White-naped Crane	-5278532.03	5878199.55
White-naped Crane	-6228939.993	5915466.162
White-naped Crane	-6178358.643	5932623.117
White-naped Crane	-5811313.792	6354643.154
White-naped Crane	-5278534.256	5878202.792
White-naped Crane	-6228933.314	5915472.674
White-naped Crane	-6178363.096	5932649.217
White-naped Crane	-6228904.371	5915427.089
White-naped Crane	-6178358.643	5932639.43
White-naped Crane	-5278538.709	5878201.171
White-naped Crane	-5811200.246	6354584.852
